# Enantioselective Narasaka–Heck cyclizations: synthesis of tetrasubstituted nitrogen-bearing stereocenters†
†Electronic supplementary information (ESI) available: Experimental procedures and characterisation data for all compounds are provided. CCDC 1438659 and 1438660. For ESI and crystallographic data in CIF or other electronic format see DOI: 10.1039/c6sc04466b



**DOI:** 10.1039/c6sc04466b

**Published:** 2016-11-24

**Authors:** Nicholas J. Race, Adele Faulkner, Gabriele Fumagalli, Takayuki Yamauchi, James S. Scott, Marie Rydén-Landergren, Hazel A. Sparkes, John F. Bower

**Affiliations:** a School of Chemistry , University of Bristol , Bristol , BS8 1TS , UK . Email: john.bower@bris.ac.uk; b AstraZeneca , Alderley Park , Macclesfield , Cheshire SK10 4TG , UK; c AstraZeneca , Pepparedsleden 1 , Mölndal , 43183 , Sweden

## Abstract

The first examples of highly enantioselective Narasaka–Heck cyclizations are described. A SPINOL-derived P,N-ligand system enables Pd-catalyzed 5-*exo* cyclization of a range of oxime esters with sterically diverse trisubstituted alkenes to generate dihydropyrroles containing tetrasubstituted nitrogen-bearing stereocenters in 56 to 86% yield and 90 : 10 to 95 : 5 e.r. These processes are rare examples of reactions that proceed *via* enantioselective migratory insertion of alkenes into Pd–N bonds, and the first where trisubstituted alkenes are used to generate tetrasubstituted stereocenters with high enantioselectivity.

## Abstract 2

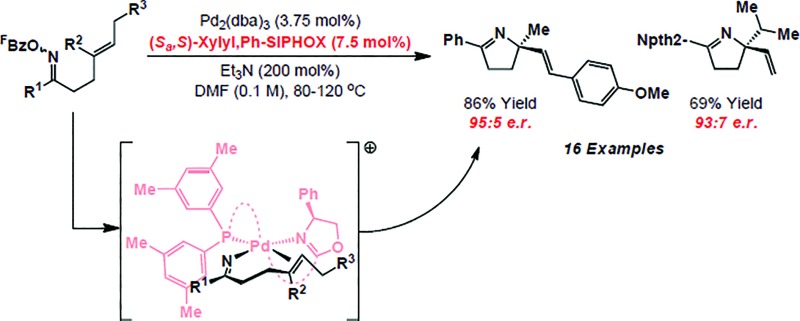
A SPINOL-derived P,N-ligand system enables Pd-catalyzed 5-*exo* cyclization of oxime esters with trisubstituted alkenes to generate dihydropyrroles in up to 86% yield and 95 : 5 e.r.

## Introduction

**Scheme 1:**
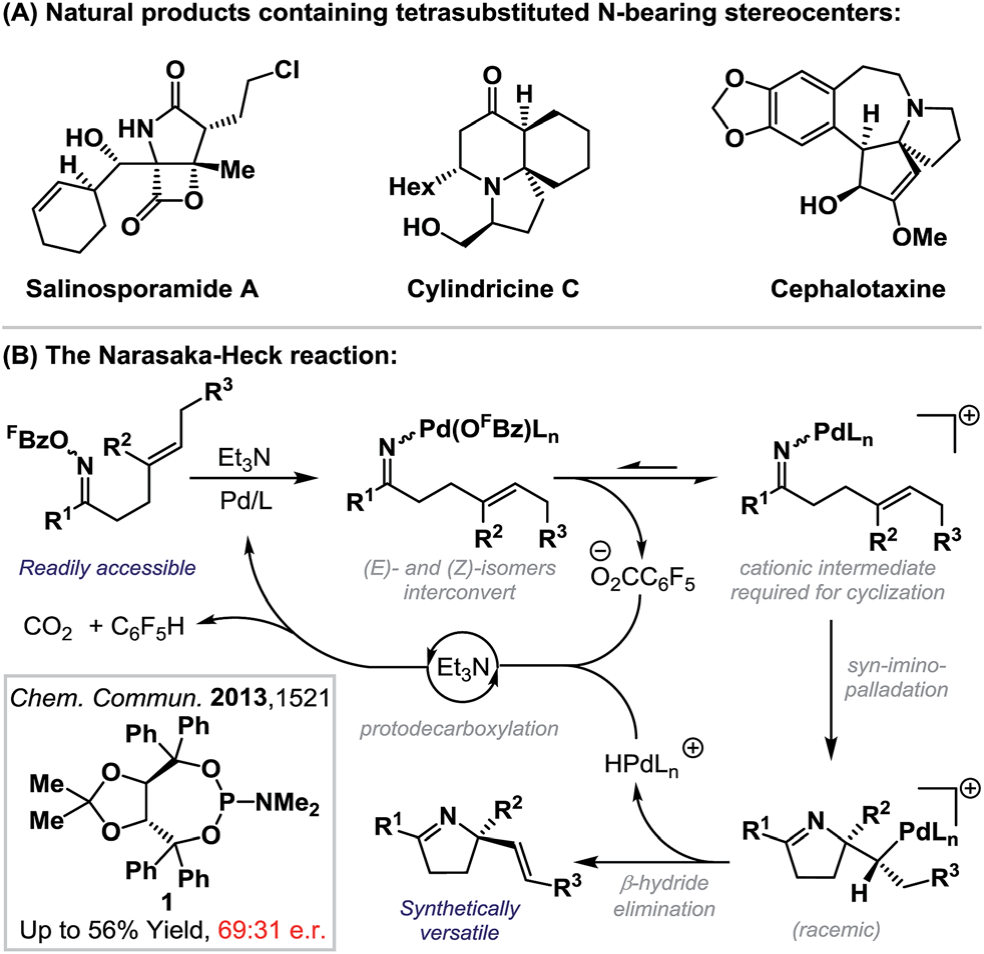


The intramolecular Heck reaction is a powerful method for the enantioselective construction of all-carbon quaternary stereocenters.^1^ Pioneering contributions from the Overman and Shibasaki groups^2^ have resulted in its establishment as a lynchpin reaction in the synthesis of a wide range of challenging natural products, most notably alkaloids.^3^ Within this class of compound, fully substituted nitrogen-bearing stereocenters are ubiquitous (Scheme 1A),^4^ and so related C–N bond formations *via* enantioselective aza-variants of the Heck reaction become appealing. First reported in 1999, the Narasaka–Heck cyclization of oxime esters with alkenes is the prototype aza-variant of the conventional Heck reaction, in so much as it incorporates the key steps of (a) N–O oxidative addition, (b) imino-palladation and (c) β-hydride elimination (Scheme 1B).^5,6^ Recently, related processes that use other classes of redox active N-donors have started to emerge.^7^

We have shown that the Narasaka reaction and related cascades are effective at generating tetrasubstituted stereocenters *via* cyclization onto a wide range of sterically encumbered alkenes.^8c,d^ However, to date, highly enantioselective variants have remained elusive. In this report, we disclose the first examples of highly enantioselective Narasaka–Heck cyclizations, which provide efficient access to sterically congested tetrasubstituted stereocenters. These studies serve as proof-of-concept for enantioselective processes of this type and, in broader terms, provide rare examples of reactions that involve enantioselective migratory insertion of alkenes into N–Pd bonds.^6,9–12^ Indeed, to the best of our knowledge, this is only the third class of process where this step is used to generate tetrasubstituted nitrogen-bearing stereocenters with high enantioselectivity,^9c,d^ and the first that achieves this using trisubstituted alkenes.

## Results and discussion

**Scheme 2:**
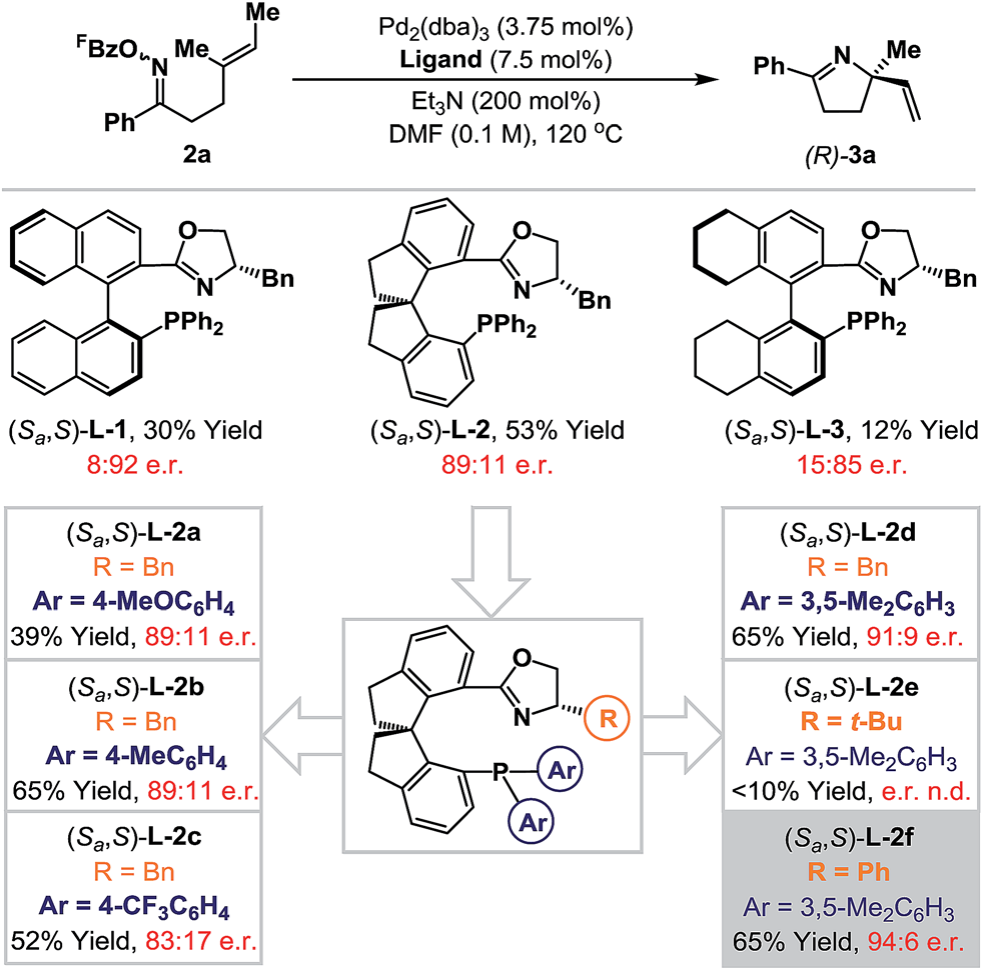
Development of an enantioselective protocol.

Rendering Narasaka–Heck cyclizations enantioselective has proven especially challenging because processes that deliver racemic products exhibit prescriptive ligand requirements, with P(3,5-(CF_3_)_2_C_6_H_3_)_3_ emerging as by far the most efficient and general system to date.^8a–d^ In earlier work we assayed a wide range of commercial mono- and bi-dentate chiral P-based systems, and established that TADDOL-phosphoramidate **1** can promote cyclization of **2a** to **3a** in 56% yield and 66 : 34 e.r. (Scheme 1B, box and Scheme 2).^8*c*^ Evaluation of a range of non-commercial variants of **1** failed to deliver a system that offered appreciable additional benefits to yield or selectivity. Our attention therefore turned to P,N-based systems, inspired, in part, by their success in conventional cationic Heck reactions.^1^ Note that in the current scenario, entry to a cationic manifold is driven by facile, triethylammonium-mediated protodecarboxylation of the pentafluorobenzoate leaving group.^8*d*^ The most promising early results were obtained using BINOL, SPINOL and H_8_-BINOL derived systems, as outlined in Scheme 2. We found that non-commercial ligands (*S*_a_,*S*)-**L-1** ^13^ and (*S*_a_,*S*)-**L-3** ^14^ provided (*S*)-**3a** with appreciable levels of enantioselectivity (92 : 8 and 85 : 15 e.r. respectively), but in low yield. However, (*S*_a_,*S*)-**L-2** ^15^ offered the best balance between cyclization efficiency and selectivity, delivering the opposite (*R*)-enantiomer of **3a** in 53% yield and 89 : 11 e.r.; this latter ligand system can be considered pseudo-diastereomeric with respect to (*S*_a_,*S*)-**L-1** and (*S*_a_,*S*)-**L-2**, which accounts for the observed switch in enantioinduction.^16^

(*S*_a_,*S*)-**L-2** is commercially available, but optimization of the oxazoline and phosphine aryl groups of this system necessitated the “in house” synthesis of a range of known non-commercial or novel analogues.^17^ Initially the phosphine aryl groups were varied ((*S*_a_,*S*)-**L-2a–d**) and these studies revealed that replacement of the phenyl groups with 3,5-dimethylated variants ((*S*_a_,*S*)-**L-2d**) offered a significant improvement in yield and a marginal enhancement in enantioselectivity for **3a**. Notably, strongly electron-donating or -withdrawing groups at the *para*-position of the arene resulted either in lower yields or lower enantioselectivities (*cf.* (*S*_a_,*S*)-**L-2a** *vs.* (*S*_a_,*S*)-**L-2c**). With a suitable phosphine aryl group established, attention turned to variations at the oxazoline portion. (*S*_a_,*S*)-**L-2e**, in which a bulky *tert*-butyl group has replaced the benzyl moiety present in (*S*_a_,*S*)-**L-2d**, was ineffective and generated **3a** in low yield (<10%). However, replacement of the benzyl group with a phenyl substituent ((*S*_a_,*S*)-**L-2f**) provided **3a** in an increased e.r. of 94 : 6 and maintained cyclization efficiency at 65% yield. Although the improvements on moving from (*S*_a_,*S*)-**L-2a** to (*S*_a_,*S*)-**L-2f** may appear modest, they are significant, and this ligand confers approximately 10–20% enhancements for both yield and e.r. (*vs.* (*S*_a_,*S*)-**L-2**) for additional examples discussed later. During the course of this work, the synthesis and application of (*S*_a_,*S*)-**L-2f** to highly enantioselective reductions of 2-pyridyl cyclic imines was reported by Zhou and co-workers.^18^

With an optimal ligand system established, we evaluated initially its scope with respect to the alkene component (Table 1). A range of systems **2b–j**, where Ar = phenyl or 2-naphthyl,^19^ cyclized to provide the targets in good to excellent yield and high enantioselectivity (91 : 9 to 95 : 5 e.r.). Notably, the system tolerates significant steric variation at R^1^ and R^2^, whilst maintaining cyclization efficiency and enantioselectivity. For example, cyclization of **2j**, which possesses an iso-propyl substituent at R^1^, afforded **3j** in 69% yield and 93 : 7 e.r. To achieve an optimal balance between cyclization efficiency and enantioselectivity, fine tuning of reaction temperature was required on a case-by-case basis. Control of substrate alkene geometry is crucial, as the alternate (*Z*)-isomer of **2c** cyclized with considerably lower levels of enantioinduction.^20^ The absolute stereochemistry of cyclization products **3a–j** was assigned on the basis of an X-ray structure of **3b** and supporting VCD analysis of **3a** and **3h** (see the ESI†).^21^

**Table 1:** Enantioselective Narasaka–Heck cyclizations: scope of the alkene component

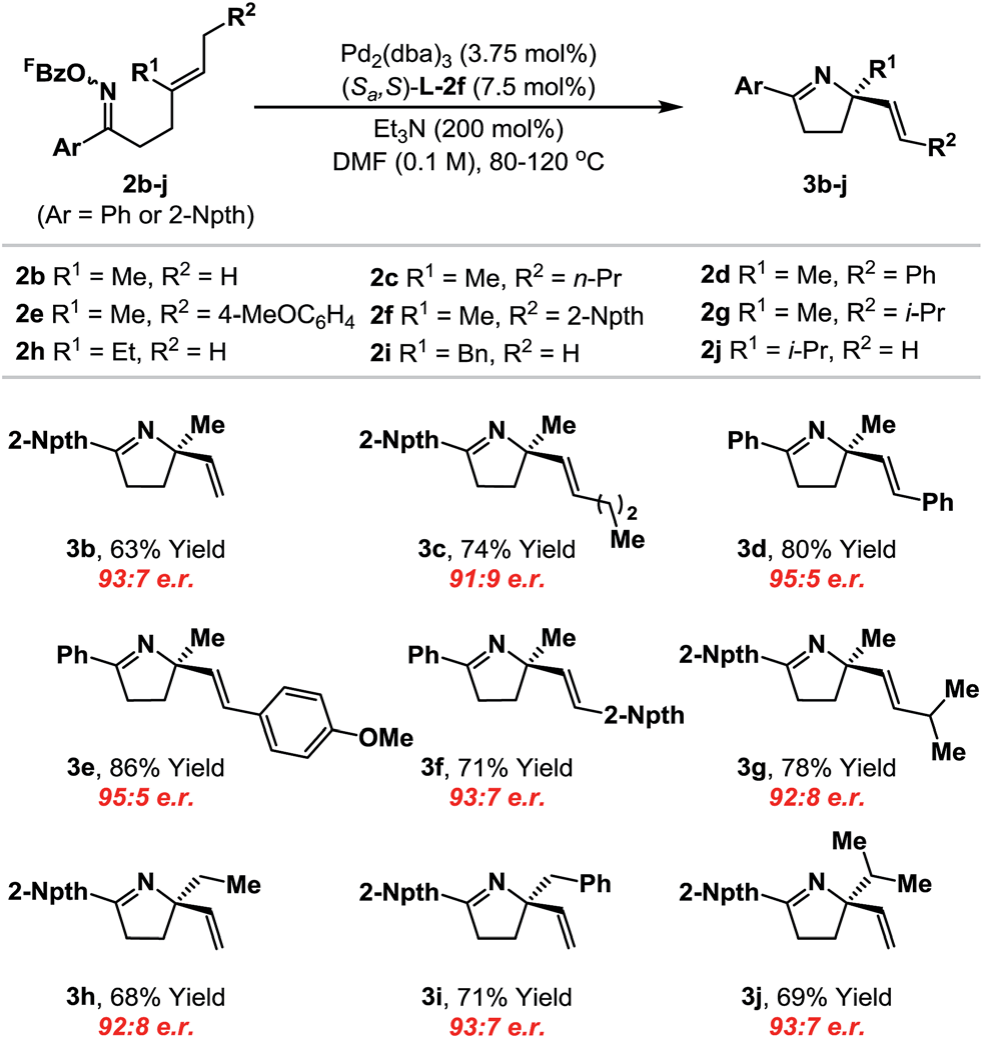

We have also conducted a preliminary evaluation of the scope of oxime ester moiety (Table 2). Systems **2k–n**, which possess electron rich or poor aryl groups at R^1^, cyclized efficiently and with minimal variation in enantioinduction. The system can be extended to other, distinct classes of oxime ester. For example, cyclization of cyclopropyl and cyclohexyl derivatives **2o** and **2p** occurred efficiently to deliver the targets **3o** and **3p** with satisfactory levels of enantioselectivity. The stereochemical assignments of the products were made by analogy to **3b** and were supported by VCD analysis of **3p** (see the ESI†). Pertinent limitations of the oxime ester moiety in non-enantioselective Narasaka–Heck cyclizations have already been delineated in our earlier work.^8a,22^

**Table 2:** Enantioselective Narasaka–Heck cyclizations: scope of the oxime ester component

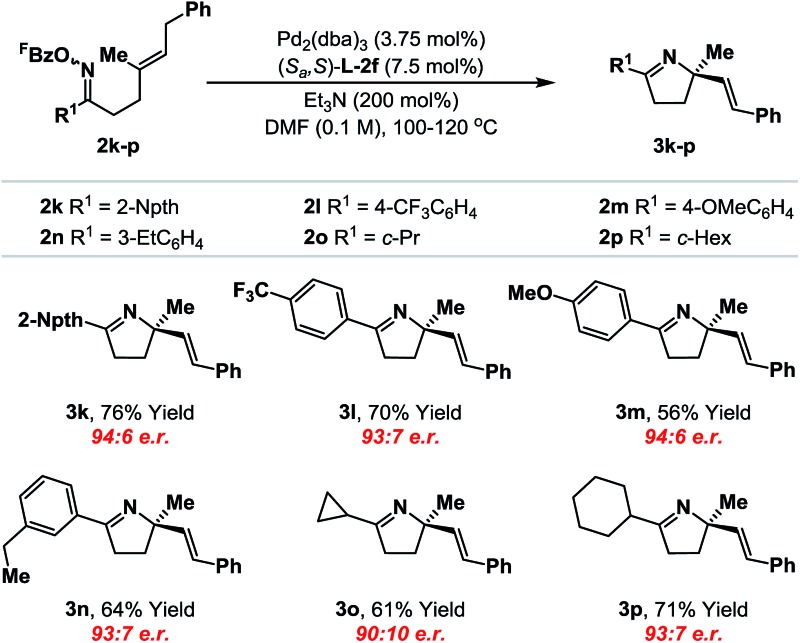

**Scheme 3:**
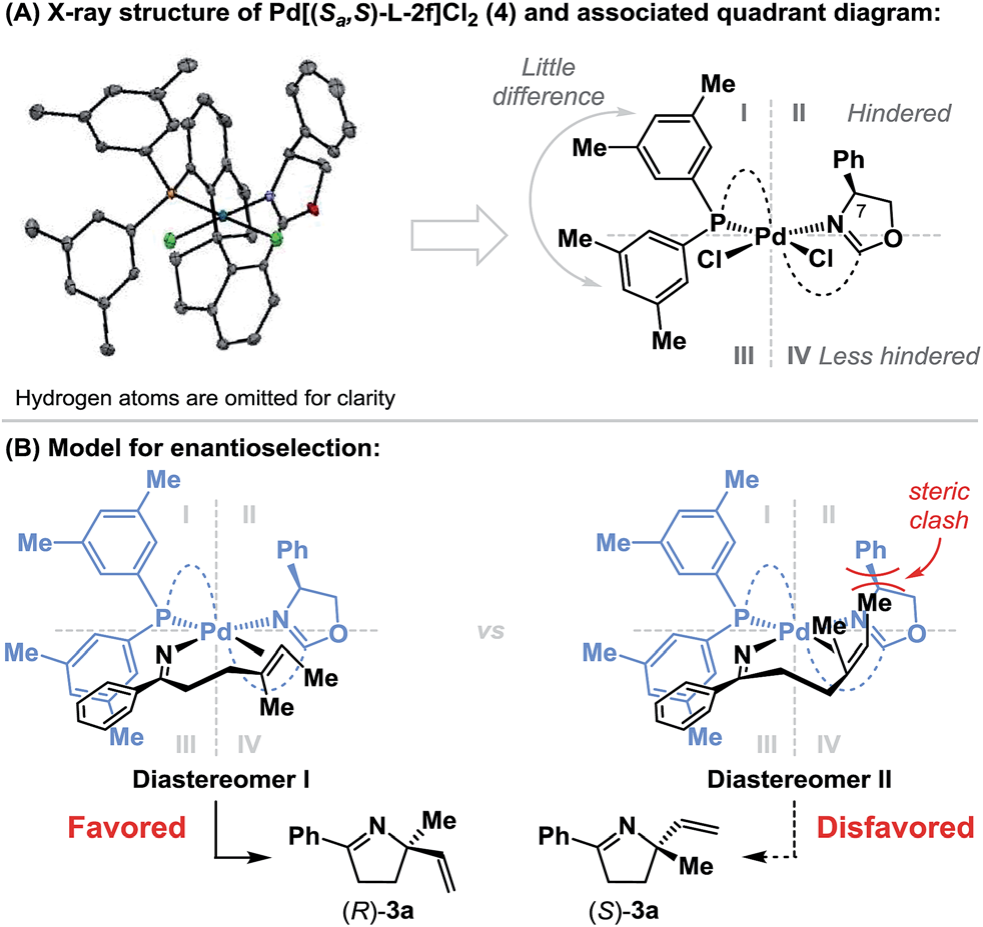


To rationalize the sense of enantioinduction in the processes described here, (*S*_a_,*S*)-**L-2f**-ligated palladium complex **4** was synthesized and characterized by single crystal X-ray diffraction (see the ESI†), and this enabled the construction of a quadrant diagram (Scheme 3).^21^ The two xylyl groups of the phosphine provide little steric difference between quadrants I and III due to the similarity of the two N–Pd–P-aryl torsion angles (–115.5° and 124.6°). The oxazoline resides approximately perpendicular to the square plane of the complex (P–Pd–N–C(7) torsion angle = 80.7°), such that the phenyl substituent occupies quadrant II, and quadrant IV remains relatively unimpeded. Scheme 3B shows the conformations of the two diastereomeric complexes that lead to enantiodivergent iminopalladation during the conversion of **2a** to **3a**. The alkene likely coordinates *trans* to the phosphine, such that differentiation of its enantiotopic faces by the phenyl substituent of the oxazoline is facilitated. For diastereomer I, which leads to the major enantiomer (*R*)-**3a**, the terminal methyl group of the alkene occupies “free” quadrant IV and steric clashes are minimized. Minor enantiomer (*S*)-**3a** requires access to the indicated conformer of diastereomer II, where the alkene methyl substituent is placed in quadrant II and suffers unfavorable interactions with the oxazoline phenyl group. The increased enantioselectivity obtained with (*S*_a_,*S*)-**L-2f** *vs.* (*S*_a_,*S*)-**L-2d** is consistent with this model (*A*_Ph_ = 3 *vs. A*_Bn_ = 1.8), as is the insensitivity of the system to increased substitution at R^2^ (*cf.* **3b** *vs.* **3j**). A key factor in the chemical efficiency of (*S*_a_,*S*)-**L-2f** likely resides in the weak donor ability of the oxazoline nitrogen, which, in turn, should enhance σ-donation from the *trans*-imino group.^23^ This lowers the basicity of this moiety, such that competing protodepalladation is suppressed and cyclization efficiency is enhanced. A similar rationale was invoked for the success of P(3,5-(CF_3_)_2_C_6_H_3_)_3_ in our earlier work.^8a–d^ In the present case, the structural features of the ligand backbone also play a key role, as highlighted by the studies outlined in Scheme 2.

**Scheme 4:**
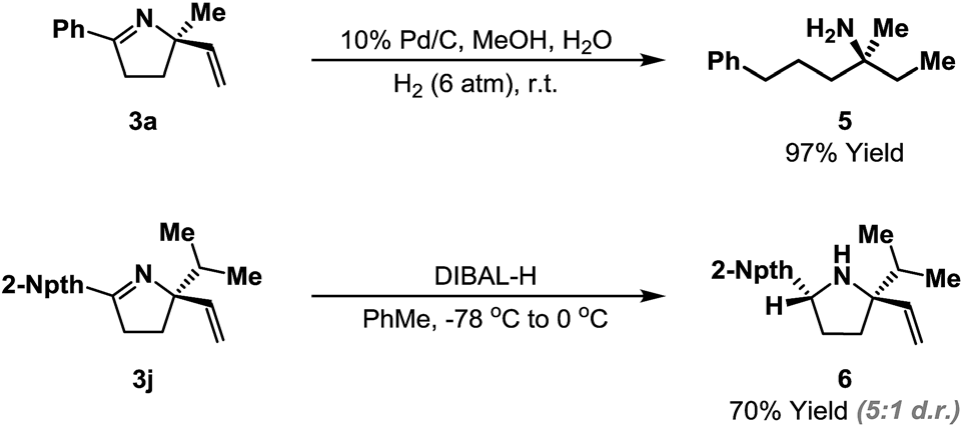
Reductive manipulations of the cyclization products.

The heterocyclic products described here retain synthetically flexible imine and alkene moieties and this provides many opportunities for derivatization. Our preliminary focus has been upon reductive manipulations of the cyclization products (Scheme 4). Exhaustive hydrogenation of both the alkene and imine moieties of **3a** (H_2_ (6 atm.), Pd/C, 4–6 days) generated efficiently acyclic target **5**, which possesses a remote, tetrasubstituted stereocenter; this defines a flexible approach to this challenging class of substrates. Chemoselective reduction of the imine of **3j** was achieved using DIBAL-H, and this occurred from the less hindered face to generate pyrrolidine **6** in 5 : 1 d.r. Related reductions of less sterically biased substrates proceeded with lower levels of diastereocontrol; efforts to address this issue will be a focus of future studies.^24^

## Conclusions

To conclude, we have outlined the identification and development of a P,N-based ligand system that promotes, for the first time, highly enantioselective Narasaka–Heck cyclizations. This provides access to challenging pyrrolidine derivatives that contain fully substituted nitrogen-bearing stereocenters and are key motifs in a wide range of alkaloid targets. The processes described here add to an emerging, yet rare class of reactions that proceed *via* enantioselective migratory insertion of alkenes into Pd–N bonds.^9–12^ Within this context, the current proof-of-concept study is unique in harnessing trisubstituted alkenes to generate tetrasubstituted stereocenters. Stereocontrolled manipulations of the cyclization products and the development of related enantioselective cyclizations and cascades are the focus of ongoing investigations in our laboratory.
