# Supplementary material for: Enantioselective Narasaka–Heck cyclizations: synthesis of tetrasubstituted nitrogen-bearing stereocenters
Source: Chem Sci. 2016 Nov 24;8(3):1981–5. doi: 10.1039/c6sc04466b (PMC5390761; doi:10.1039/c6sc04466b)

Electronic Supplementary Material (ESI) for Chemical Science.
This journal is © The Royal Society of Chemistry 2016

Enantioselective Narasaka-Heck Cyclizations: Synthesis of Tetrasubstituted Nitrogen-Bearing Stereocenters

Nicholas J. Race,[†] Adele Faulkner,[†] Gabriele Fumagalli,[†] Takayuki Yamauchi,[†] James S. Scott,[‡] Marie Rydén-Landergren,[§] Hazel A. Sparkes,[†] and John F. Bower[*,†]

[†] *School of Chemistry, University of Bristol, Bristol, BS8 1TS, United Kingdom*
[‡] *AstraZeneca, Alderley Park, Macclesfield, Cheshire, SK10 4TG, United Kingdom*
[§] *AstraZeneca, Pepparedsleden 1, Mölndal, 43183, Sweden*

Electronic Supplementary Information

Table of Contents

General Experimental Details ---------- 2
Experimental Procedures and Data ---------- 3
X-ray Crystallography Experimental Details---------- 68
References ---------- 69
Copies of ^{1}H and ^{13}C NMR spectra for novel compounds ---------- 70

General experimental details

Starting materials sourced from commercial suppliers were used as received unless otherwise stated. Dry solvents, where necessary, were obtained by distillation using standard procedures or by passage through a column of anhydrous alumina using equipment from Anhydrous Engineering based on the Grubbs' design. Petrol refers to the fraction of petroleum ether boiling in the range of 40-60 °C. The removal of solvents *in vacuo* was achieved using both a Büchi rotary evaporator (bath temperatures up to 40 °C) at a pressure of either 15 mmHg (diaphragm pump) or 0.1 mmHg (oil pump), as appropriate, and a high vacuum line at room temperature. Reactions requiring anhydrous conditions were run under an atmosphere of dry nitrogen; glassware, syringes and needles were either flame-dried immediately prior to use or placed in an oven (150 °C) for at least 2 h and allowed to cool either in a desiccator or under high vacuum on a Schlenk line; liquid reagents, solutions or solvents were added *via* syringe through rubber septa; solid reagents were added *via* Schlenk type adapters. Commercially available Merck Kieselgel $60F_{254}$ aluminium-backed plates were used for TLC analysis. Visualisation was achieved by UV fluorescence and/or basic $KMnO_4$ solution and heat. Flash column chromatography (FCC) was performed using Merck Kieselgel 60 (40–63 μm). The crude material was applied to the column as a solution of either the appropriate eluent or minimum amount of CH_2Cl_2, or by pre-adsorption onto silica, as appropriate. Melting points were determined using a Gallenkamkp metling point apparatus and temperature controller and are uncorrected. Optical rotations were measured using an ADP220 polarimeter (Bellingham & Stanley Ltd.) and are reported in $deg.cm^2.g^{-1}$. Infra-red spectra were recorded in the range 4000-600 cm^{-1} on a Perkin Elmer Spectrum One spectrometer either as neat films or solids compressed onto a diamond window. Abbreviations used are: w (weak), m (medium), s (strong) and br. (broad). NMR spectra were recorded on a JEOL Eclipse 300, JEOL ECS 400, Varian 400-MR, Varian VNMR S500a or Varian VNMR S500b spectrometer. Chemical shifts are quoted in parts per million (ppm); ^1H NMR spectra are referenced to residual protons of the deuterated solvent as an internal standard; ^{13}C NMR are referenced to the deuterated solvent as an internal standard; ^{19}F NMR spectra are referenced to CCl_3F as an external standard; ^{31}P NMR spectra are referenced to H_3PO_4 as an external standard. Coupling constants (*J*) are quoted to the nearest 0.5 Hz for ^1H NMR and nearest 0.1 Hz for ^{13}C NMR. Other abbreviations used are: s (singlet), d (doublet), t (triplet), q (quartet), hep (heptet), m (multiplet), and br (broad). Assignments of ^1H NMR and ^{13}C NMR signals were made, where possible, using COSY, DEPT, HMQC, HMBC and nOe experiments. The proton and carbon assignments are

listed based on the ‘non-IUPAC’ numbering system associated with each structure. Mass spectra were determined by the University of Bristol mass spectrometry service by either electron impact (EI^+) or chemical ionisation (CI^+) using a VG Micromass Autosepec spectrometer, or by electrospray ionisation (ESI^+) using a Brüker Daltonics MicrOTOF II spectrometer. Chiral HPLC was performed using either the racemate or the antipode as a standard on an Agilent 1290 Infinity system equipped with a quaternary pump, diode array detector and column thermostat under the conditions specified in each case.

General Procedures

General Procedure A for the preparation of alcohol precursors from the corresponding aldehyde/esters: To a solution of the appropriate ester/aldehyde (100 mol%) in anhydrous THF (1 mL/mmol) at 0 °C was slowly added 1M $LiAlH_4$ (110 mol%) in THF (2 mL/mmol). The mixture was stirred at room temperature until complete consumption of ester/aldehyde was observed by TLC (*approx*. 1-5 hours). The mixture was cooled to 0 °C and water (0.5 mL/mmol), NaOH (1 mL/mmol) and water (1 mL/mmol) were added sequentially. The suspension was filtered through celite®, washing with CH_2Cl_2 (150 mL/mmol), and the resulting solution was concentrated *in vacuo*. Purification of the residue by FCC under the conditions noted, afforded the corresponding alcohol.

General Procedure B for the preparation of bromide precursors from the corresponding alcohol: To a solution of the appropriate alcohol (100 mol%) in Et_2O (2 mL/mmol) at 0 °C was added PBr_3 (50 mol%). The solution was warmed to room temperature and stirred for 16 hours. The reaction was cooled to 0 °C and water (1 mL/mmol) and 5% aq. K_2CO_3 (1 mL/mmol) were added. The organic portion was isolated, washed with brine (1 mL/mmol), dried (Na_2SO_4) and concentrated *in vacuo* to afford the corresponding bromide, which was used in the next stage without further purification unless otherwise stated.

General Procedure C for the preparation of ketone precursors *via* the corresponding β-keto ester. Part A: To NaH (100 mol%, 60% dispersion in mineral oil), the appropriate β-keto ester (100 mol%) was added at 0 °C. Anhydrous DMF (5 mL/mmol) was added to the reaction *via* cannula. The mixture was stirred at room temperature until gas evolution ceased (*approx*. 15 minutes). The appropriate chloride/bromide (110 mol%) was then added *via* syringe and the mixture was heated at 70 °C for 16 hours. The mixture was cooled to room temperature and the solvent removed by concentration *in vacuo* to afford the alkylated product which was used without further purification unless otherwise stated. Part B: To the residue in THF (2 mL/mmol), MeOH (1 mL/mmol), water (1 mL/mmol) and KOH (500 mol%) were added. The mixture was then heated at 75 °C until complete consumption of intermediate

ester was observed by TLC (*approx.* 6-16 hours). After cooling to room temperature, the mixture was acidified with aq. 1 M HCl (8 mL/mmol) and extracted with Et_2O (20 mL/mmol). The organic extracts were washed with brine (10 mL/mmol), dried (Na_2SO_4) and concentrated *in vacuo*. Purification of the residue by FCC, under the conditions noted, afforded the corresponding alkylated ketone.

General Procedure D for oxime ester formation: Part A: $H_2NOH.HCl$ (120 mol%) and NaOAc (120 mol%) were added to a solution of the appropriate ketone (100 mol%) in MeOH (3 mL/mmol). The mixture was heated at 75 °C for the specified time. After cooling to room temperature, the mixture was diluted with EtOAc (10 mL/mmol), washed with brine (10 mL/mmol), dried (Na_2SO_4) and concentrated *in vacuo*. The oxime obtained in this way was used in the next stage without further purification, unless otherwise stated. Part B: To a solution of the appropriate oxime (100 mol%) in anhydrous CH_2Cl_2 (3 mL/mmol) at 0 °C was added, *via* syringe, Et_3N (200 mol%) and then $ClC(O)C_6F_5$ (120 mol%). The mixture was then warmed to room temperature and stirred for the specified time. MeOH (0.5 mL/mmol) and then EtOAc (15 mL/mmol) were added. The mixture was then washed with saturated aq. Na_2CO_3 (2 × 15 mL/mmol) and brine (15 mL/mmol), dried (Na_2SO_4) and concentrated *in vacuo*. The residue was purified by FCC, under the conditions noted, to afford the corresponding oxime ester.

General Procedure E for asymmetric Narasaka-Heck cyclizations: An oven-dried reaction tube, fitted with magnetic stirrer, was charged with $Pd_2(dba)_3$ (3.75 mol%), (S_a,*S*)-**L-2f** (7.5 mol%) and oxime ester substrate (100 mol%). The tube was fitted with a rubber septum and purged with argon. Anhydrous DMF (10 mL/mmol) and then reagent grade Et_3N (200 mol%) were added *via* syringe. The mixture was then placed in a preheated oil bath at the specified temperature until complete consumption of starting material was observed (*approx.* 1-7 hours, as noted). The mixture was then cooled to room temperature and concentrated *in vacuo* (*approx.* 1.0 mmHg). The residue was purified by flash column chromatography, under the conditions noted, to afford the target heterocycle.

Experimental Procedures

The synthesis of binaphthyl *P*,*N*-ligand (S_a,*S*)-**L-1** was carried out following reported literature procedures.[1,2]

The synthesis of H8-binaphthyl *P*,*N*-ligand (S_a,*S*)-**L-3** was carried out following reported literature procedures.[3]

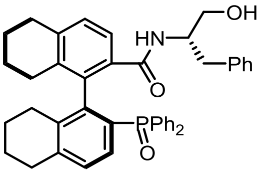

(*S*)-2'-(Diphenylphosphoryl)-*N*-((*S*)-1-hydroxy-3-phenylpropan-2-yl)-5,5',6,6',7,7',8,8'-octahydro-[1,1'-binaphthalene]-2-carboxamide

$[\alpha]_D^{21}$ -53.0 (c = 1.0, $CHCl_3$).

<u>^{1}H NMR</u> (400 MHz, $CDCl_3$): 8.31 (d, *J* = 8.5 Hz, 1H, NH), 7.78-7.68 (m, 3H, ArH), 7.54-7.25 (m, 10H, ArH) 7.21-7.08 (m, 4H, ArH), 7.03 (dd, *J* = 8.0, 3.0 Hz, 1H, ArH), 6.86 (d, *J* = 8.0 Hz, 1H, ArH), 4.13-4.06 (m, 1H, CH), 3.38 (dd, *J* = 11.5, 3.0 Hz, 1H, CH_2), 3.26 (dd, *J* = 11.5, 4.0 Hz, 1H, CH_2), 2.96-2.86 (m, 2H, CH_2), 2.84-2.72 (m, 2H, CH_2), 2.54-2.43 (m, 2H, CH_2), 2.29-2.21 (m, 1H, CH_2), 1.88 (dt, *J* = 18.0, 6.5 Hz, 1H, CH_2), 1.75-1.50 (m, 5H, CH_2), 1.38-1.20 (m, 4H, CH_2), 0.87-0.78 (m, 1H, CH_2).

<u>^{13}C NMR</u> (100 MHz, $CDCl_3$): 170.7 (CO), 144.2 (d, *J* = 8.2, ArC), 143.2 (d, *J* = 2.6, ArC), 141.5 (ArC), 138.9 (d, *J* = 10.4, ArC), 138.6 (d, *J* = 14.9, ArC), 135.4 (ArC), 134.7 (d, *J* = 4.2, ArC), 134.6 (ArC), 132.0 (d, *J* = 9.2, ArCH), 131.9 (d, *J* = 105.1, ArC), 131.9 (d, *J* = 2.8, ArCH), 131.4 (d, *J* = 2.9, ArCH), 131.3 (d, *J* = 9.8, ArCH), 130.7 (d, *J* = 13.6, ArCH), 130.4 (d, *J* = 104.6, ArC), 129.2 (ArCH), 129.2 (ArCH), 128.5 (d, *J* = 11.9, ArCH), 128.3 (ArCH), 128.1 (d, *J* = 14.0, ArCH), 128.0 (d, *J* = 12.5, ArCH),126.3 (ArCH), 126.2 (ArCH), 126.0 (d, *J* = 104.4, ArC), 125.3 (ArCH), 124.2 (ArCH), 117.9 (ArCH), 110.6 (ArCH), 63.0 (CH_2), 53.3 (CH), 36.9 (CH_2), 30.3 (CH_2), 29.6 (CH_2), 27.0 (2C, 2 × CH_2), 22.9 (CH_2), 22.3 (CH_2), 22.2 (CH_2), 22.0 (CH_2).

<u>^{31}P NMR</u> (162 MHz, $CDCl_3$): 31.5 (s).

<u>MS</u>: (ESI^+) Found $[M+H]^+$: 640.2975, $C_{42}H_{43}NO_3PN$ requires 640.2975.

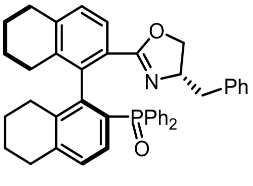

((*S*)-2'-((*S*)-4-Benzyl-4,5-dihydrooxazol-2-yl)-5,5',6,6',7,7',8,8'-octahydro-[1,1'-binaphthalen]-2-yl)diphenylphosphine oxide

$[\alpha]_D^{21}$ -77.6 (c = 1.16, $CHCl_3$).

^{1}H NMR (400 MHz, $CDCl_3$): 7.66-7.60 (m, 2H, ArH), 7.43-7.06 (m, 16H, ArH), 6.89 (d, *J* = 8.0 Hz, 1H, ArH), 4.17 (dddd, *J* = 9.5, 7.5, 7.5, 7.0 Hz, 1H, CH), 3.95 (dd, *J* = 9.5, 8.0 Hz, 1H, CH_2), 3.69 (t, *J* = 8.0 Hz, 1H, CH_2), 2.83-2.70 (m, 5H, CH_2), 2.51-2.43 (m, 2H, CH_2), 2.19 (dt, *J* = 17.0, 6.5 Hz, 1H, CH_2), 1.94-1.81 (m, 3H, CH_2), 1.76-1.49 (m, 7H, CH_2).

^{13}C NMR (100 MHz, $CDCl_3$): 162.9 (CN), 145.1 (d, *J* = 8.5, ArC), 141.2 (d, *J* = 2.8, ArC), 140.4 (ArC), 138.9 (ArC), 138.6 (d, J = 4.2, ArC), 136.4 (d, *J* = 10.5, ArC), 134.8 (d, *J* = 102.7, ArC), 133.4 (d, *J* = 102.7, ArC), 132.1 (d, *J* = 9.3, ArCH), 131.8 (d, *J* = 9.7, ArCH), 131.1 (d, *J* = 11.9, ArCH), 130.9 (d, *J* = 2.8, ArCH), 130.3 (d, *J* = 2.8, ArCH), 129.1 (ArCH), 128.6 (ArCH), 128.2 (ArCH), 127.8 (d, *J* = 11.8, ArCH), 127.3 (ArCH), 127.3 (d, *J* = 105.4, ArC), 127.2 (ArCH), 126.5 (ArCH), 126.1 9 ArCH), 124.0 (ArC), 70.8 (CH_2), 68.1 (CH), 42.0 (CH_2), 30.4 (CH_2), 30.2 (CH_2), 27.9 (CH_2), 27.1 (CH_2), 23.3 (CH_2), 23.1 (CH_2), 22.6 (CH_2), 22.4 (CH_2), 1 × ArC not observed.

^{31}P NMR (162 MHz, $CDCl_3$): 28.1 (s).

FTIR: 2927, 1642, 1436, 1177, 1115, 1071 cm^{-1}.

MS: (ESI^+) Found $[M+H]^+$: 622.2876, $C_{42}H_{41}NO_2P$ requires 622.2869.

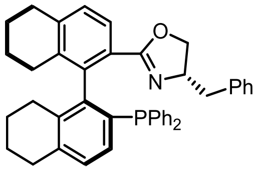

(*S*)-4-Benzyl-2-((*S*)-2'-(diphenylphosphanyl)-5,5',6,6',7,7',8,8'-octahydro-[1,1'-binaphthalen]-2-yl)-4,5-dihydrooxazole (S_a,*S*)**-L-3**

$[\alpha]_D^{21}$ -70.1 (c = 1.0, $CHCl_3$).

^{1}H NMR (400 MHz, $CDCl_3$): 7.71 (d, *J* = 8.0 Hz, 1H, ArH), 7.27-7.02 (m, 18H, ArH), 4.13 (dtd, *J* = 9.5, 7.5, 6.5 Hz, 1H, CH), 3.83 (dd, *J* = 9.5, 8.0 Hz, 1H, CH_2), 3.61 (dd, *J* = 8.0, 8.0 Hz, 1H, CH_2), 2.89-2.77 (m, 5H, CH_2), 2.40-2.33 (m, 2H, CH_2), 2.10-2.02 (m, 1H, CH_2), 1.94-1.90 (m, 2H, CH_2), 1.74-1.55 (m, 6H, CH_2), 1.51-1.41 (m, 1H, CH_2), 1.32-1.23 (m, 1H, CH_2).

^{13}C NMR (100 MHz, $CDCl_3$): 163.6 (CN), 147.1 (ArC), 146.8 (ArC), 140.7 (d, *J* = 8.7, ArC), 140.5 (ArC), 138.9 (d, *J* = 13.0, ArC), 138.4 (d, *J* = 13.4, ArC), 138.1 (d, *J* = 1.1, ArC), 136.0 (d, *J* = 1.9, ArC), 135.2 (d, *J* = 6.9, ArC), 134.0 (d, *J* = 20.5, ArCH), 133.4 (d, *J* = 18.8, ArCH), 132.8 (d, *J* = 6.8, ArC), 131.3 (d, *J* = 2.3, ArCH), 129.1 (ArCH), 128.3 (2C, 2 × ArCH), 128.2 (ArCH), 128.1 (d, *J* = 7.0, ArCH), 128.0 (ArCH), 127.7 (d, *J* = 5.8, ArCH), 127.5 (ArCH), 126.7 (ArCH), 126.1 (ArCH), 124.8 (d, *J* = 3.0, ArC), 70.8 (CH_2), 68.1 (CH), 42.0, 30.4, 30.2, 27.9, 27.1, 23.3, 23.1, 22.6, 22.4 (9 × CH_2).

^{31}P NMR (162 MHz, $CDCl_3$): -15.5 (s).

*The synthesis of enantioenriched triflate **(S)-S1** was carried out following procedures which were, in some cases, different to the original report.*

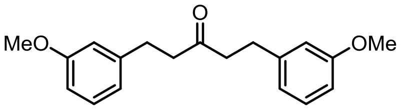

1,5-Bis(3-methoxyphenyl)pentan-3-one

To a solution of NaOH (22.7 g, 568 mmol) in H_2O (200 mL) and EtOH (200 mL) at 0 °C was added a solution of acetone (8.14 mL, 111 mmol) and *m*-anisaldehyde (27.0 mL, 222 mmol) in EtOH (50 mL) dropwise over 30 minutes. During this time the reaction mixture turned from colorless to yellow. After the addition was complete the reaction mixture was stirred for a further 2 hours after which time it was diluted with CH_2Cl_2 (150 mL) and the phases were separated. The organic layer was washed with H_2O/brine (1:1, 140 mL), dried (Na_2SO_4), filtered and concentrated *in vacuo* to give crude 1,5-bis(3-methoxyphenyl)-1,4-pentadien-3-one as a viscous, yellow oil (32.6 g). This material was used in the next step without further purification.

^{1}H NMR (400 MHz, $CDCl_3$): 7.70 (d, *J* = 16.0 Hz, 2H, CH), 7.33 (t, *J* = 8.0 Hz, 2H, ArCH), 7.21 (d, *J* = 7.5 Hz, 2H, ArCH), 7.13 (t, *J* = 2.0 Hz, 2H, ArCH), 7.06 (d, *J* = 16.0 Hz, 2H, CH), 6.96 (ddd, *J* = 8.0, 2.5, 1.0 Hz, 2H, ArCH), 3.85 (s, 6H, OCH_3).

The crude 1,5-bis(3-methoxyphenyl)-1,4-pentadien-3-one (assuming 111 mmol) was dissolved in EtOAc (300 mL) and Pd/C (760 mg, 10% Pd, 7.20 mmol) was added under nitrogen. The atmosphere was saturated with hydrogen gas (1 atm) and stirred vigorously under a balloon of hydrogen until consumption of starting material was confirmed by TLC. The reaction mixture was then degassed with argon, filtered through a pad of celite® and concentrated *in vacuo*. Purification of the residue (4:1 hexane:EtOAc) afforded the title compound (15.5 g, 47% over 2 steps) as a colorless oil.

^{1}H NMR (400 MHz, $CDCl_3$): 7.20-7.15 (2H, m, ArH), 6.74-6.70 (6H, m, ArH), 3.77 (6H, s, OCH_3), 2.85 (t, *J* = 8.0 Hz, 4H, CH_2), 2.69 (t, *J* = 8.0 Hz, 4H, CH_2).

^{13}C NMR (100 MHz, $CDCl_3$): 209.4 (CO), 160.1 (ArC), 143.1 (ArC), 129.9, 121.0, 114.6, 111.8 (4 × ArCH), 55.6 (OCH_3), 44.9 (CH_2), 30.3 (CH_2). *The spectroscopic properties of this compound were consistent with the data available in the literature.*[4]

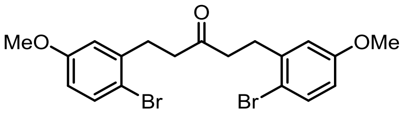

1,5-Bis(2-bromo-5-methoxyphenyl)pentan-3-one

To a solution of 1,5-bis(3-methoxyphenyl)pentan-3-one (15.5 g, 52.0 mmol) in acetone (100 mL) at 0 °C was added *N*-bromosuccinimide (19.4 g, 109 mmol) portionwise under nitrogen, followed by a few drops of aq. 1 M HCl. After 1 minutes, the cloudy reaction mixture turned clear which indicated reaction

completion. The mixture was then concentrated *in vacuo* and the residue was redissolved in Et_2O (300 mL), washed with H_2O (100 mL), brine (100 mL), dried (Na_2SO_4), filtered and concentrated *in vacuo* to give the title compound (23.3 g, 99%) as a colorless oil that solidified on standing. This material was used in the next step without further purification.

^{1}H NMR (400 MHz, $CDCl_3$): 7.39 (d, J = 8.5 Hz, 2H, ArH), 6.77 (d, J = 3.0 Hz, 2H, ArH), 6.63 (dd, J = 8.5, 3.0 Hz, 2H, ArH), 3.76 (s, 6H, OCH_3), 2.96 (dd, J = 8.5, 7.0 Hz, 4H, CH_2), 2.73 (dd, J = 8.5, 7.0 Hz, 4H, CH_2). *The spectroscopic properties of this compound were consistent with the data available in the literature.*[5]

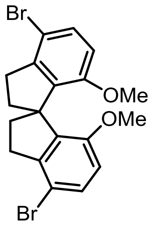

4,4'-Dibromo-7,7'-dimethoxy-2,2',3,3'-tetrahydro-1,1'-spirobi[indene]

A heterogeneous solution of 1,5-bis(2-bromo-5-methoxyphenyl)pentan-3-one (4.54 g, 10.0 mmol) and phosphotungstic acid hydrate (4.32 g, 1.50 mmol) in toluene (60 mL) was heated at 140 °C under Dean-Stark conditions for 18 hours. The reaction mixture was then filtered through a pad of celite®, washing with $CHCl_3$ (150 mL). The filtrate was concentrated *in vacuo* and purification of the residue by FCC (20:1 hexane:EtOAc), followed by trituration of the resulting solid with ice-cold Et_2O, afforded the title compound (2.83 g, 65%) as a colorless solid.

^{1}H NMR (400 MHz, $CDCl_3$): 7.26 (d, J = 8.5 Hz, 2H, ArH), 6.52 (dd, J = 8.6, 0.5 Hz, 2H, ArH), 3.52 (s, 6H, OCH_3), 3.07 (dddd, J = 16.5, 9.0, 4.0, 0.5 Hz, 2H, CH_2), 2.95 (dddd, J = 16.5, 8.0, 8.0, 1.0 Hz, 2H, CH_2), 2.32 (dddd, J = 13.0, 9.0, 8.0, 0.5 Hz, 2H, CH_2), 2.16 (ddd, J = 12.5, 8.4, 4.0 Hz, 2H, CH_2).
^{13}C NMR (100 MHz, $CDCl_3$): 155.5, 144.9, 138.0 (3 × ArC), 130.2, 100.7, 110.4 (3 × ArCH), 61.9 (C), 55.2 (OCH_3), 37.8 (CH_2), 33.1 (CH_2). *The spectroscopic properties of this compound were consistent with the data available in the literature.*[6]

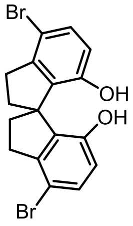

4,4'-Dibromo-2,2',3,3'-tetrahydro-1,1'-spirobi[indene]-7,7'-diol

To a Schlenk tube containing CH_2Cl_2 (60 mL) at -78 °C was added BBr_3 (6.00 mL, 62.3 mmol) in one portion *via* syringe. Separately, a -78 °C solution of 4,4'-dibromo-7,7'-dimethoxy-2,2',3,3'-tetrahydro-1,1'-spirobi[indene] (11.5 g, 26.4 mmol) in CH_2Cl_2 (110 mL) was prepared. The BBr_3 solution was cannula-transferred to the solution of 4,4'-dibromo-7,7'-dimethoxy-2,2',3,3'-tetrahydro-1,1'-spirobi[indene] over 10 minutes. The reaction mixture was warmed to room temperature and stirred

until complete conversion to product was observed by TLC analysis (*approx*. 48 hours). The mixture was diluted with CH_2Cl_2 (125 mL), cooled to 0 °C and carefully quenched with saturated aq. $NaHCO_3$ (250 mL). The layers were separated and the organic layer washed with brine (100 mL), dried (Na_2SO_4), filtered and concentrated *in vacuo* to give 4,4'-dibromo-2,2',3,3'-tetrahydro-1,1'-spirobi[indene]-7,7'-diol (10.2 g, 95%) as a colorless foam. This material was used in the next step without further purification.

^{1}H NMR (400 MHz, $CDCl_3$): 7.30 (d, *J* = 8.5 Hz, 2H, ArH), 6.59 (dt, *J* = 8.5, 0.5, 2H, ArH), 3.11-2.94 (m, 4H, CH_2), 2.31 (ddd, *J* = 13.0, 7.5, 2.0, 2H, CH_2), 2.24-2.15 (m, 2H, CH_2). *The spectroscopic properties of this compound were consistent with the data available in the literature.*[7]

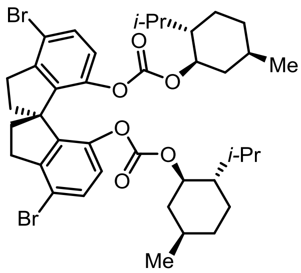

(*S*)-4,4'-Dibromo-2,2',3,3'-tetrahydro-1,1'-spirobi[indene]-7,7'-diyl bis((1*R*,2*S*,5*R*)-2-isopropyl-5-methylcyclohexyl) bis(carbonate)

To a solution of NaOH (4.44 g, 111 mmol) in H_2O (75 mL) was added 4,4'-dibromo-2,2',3,3'-tetrahydro-1,1'-spirobi[indene]-7,7'-diol (10.2 g, 25.0 mmol) and a solution of tetrabutylammonium bromide (3.72 g, 11.5 mmol) in $CHCl_3$ (75 mL). The mixture was cooled to 0 °C and (1*R*)-(−)-menthyl chloroformate (16.0 mL, 74.7 mmol) was added dropwise over 10 minutes. The reaction mixture was warmed to room temperature and stirred for 10 minutes. After this time, the phases were separated and the aqueous phase was extracted with CH_2Cl_2 (2 × 50 mL). The combined organic phases were washed with brine (50 mL), dried (Na_2SO_4), filtered and concentrated *in vacuo*. Recrystallisation of the residue from hot hexane (*approx.* 700 mL) afforded the title compound (6.97 g, 36%) as a colorless solid.

$[\alpha]^{21}_{D}$ -83.8 (c = 0.8, $CHCl_3$) [Lit.[7] $[\alpha]^{25}_{D}$ -91.0 (c = 1.0, $CHCl_3$)].

M.P. 218-220 °C (hexane) [Lit.[7] 220-222 °C (hexane)].

^{1}H NMR (500 MHz, $CDCl_3$): 7.35 (d, *J* = 8.5 Hz, 2H, ArH), 6.87 (d, *J* = 8.5 Hz, 2H, ArH), 4.36 (ddd, *J* = 11.0, 11.0, 4.5 Hz, 2H, CH), 3.10-2.96 (m, 4H, CH), 2.31-2.22 (m, 4H, CH), 1.87-1.83 (m, 2H, CH), 1.67-1.60 (m, 6H, CH), 1.44-1.35 (m, 2H, CH), 1.30 (ddt, *J* = 12.5, 11.0, 3.0 Hz, 2H, CH), 1.01-0.78 (m, 18H, CH), 0.71 (d, *J* = 7.0 Hz, 6H, CH). *The spectroscopic properties of this compound were consistent with the data available in the literature*.[7]

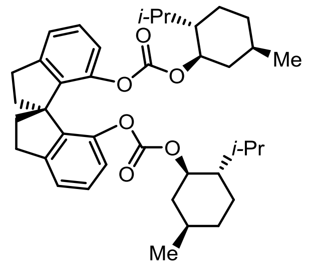

Bis((1*R*,2*S*,5*R*)-2-isopropyl-5-methylcyclohexyl) ((*S*)-2,2',3,3'-tetrahydro-1,1'-spirobi[indene]-7,7'-diyl) bis(carbonate)[8]

To a solution of (*S*)-4,4'-dibromo-2,2',3,3'-tetrahydro-1,1'-spirobi[indene]-7,7'-diyl bis((1*R*,2*S*,5*R*)-2-isopropyl-5-methylcyclohexyl) bis(carbonate) (3.16 g, 4.09 mmol) in THF (80 mL) at -78 °C was added *n*-BuLi (1.58 M in hexane, 6.46 mL, 10.2 mmol). The reaction was stirred at this temperature for 1 hour followed by addition of AcOH (2.05 mL, 35.9 mmol). The reaction mixture was then warmed to room temperature and concentrated *in vacuo*. The residue was taken up in Et_2O (300 mL), washed with saturated aq. $NaHCO_3$ (50 mL), H_2O (50 mL), brine (50 mL), dried (Na_2SO_4), filtered and concentrated *in vacuo* to yield bis((1*R*,2*S*,5*R*)-2-isopropyl-5-methylcyclohexyl) ((*S*)-2,2',3,3'-tetrahydro-1,1'-spirobi[indene]-7,7'-diyl) bis(carbonate) (2.52 g, quant.) as a colorless solid. This material was used in the next step without further purification.

^{1}H NMR (400 MHz, $CDCl_3$): 7.19 (t, *J* = 7.5 Hz, 2H, ArH), 7.10 (d, *J* = 7.5 Hz, 2H, ArH), 6.92 (d, *J* = 8.0 Hz, 2H, ArH), 4.34 (td, *J* = 11.0, 4.5 Hz, 2H, CH), 3.07-2.91 (m, 4H, CH), 1.91-1.86 (m, 2H, CH), 1.64-1.17 (m, 10H, CH), 1.00-0.75 (m, 18H, CH), 0.67 (d, *J* = 7.0 Hz, 6H, CH). *The spectroscopic properties of this compound were consistent with the data available in the literature*.[9]

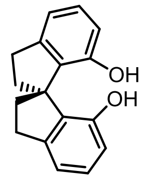

(*S*)-2,2',3,3'-Tetrahydro-1,1'-spirobi[indene]-7,7'-diol

To a solution of KOH (22.0 g, 396 mmol) in 10% degassed H_2O/EtOH (460 mL) was added bis((1*R*,2*S*,5*R*)-2-isopropyl-5-methylcyclohexyl) ((*S*)-2,2',3,3'-tetrahydro-1,1'-spirobi[indene]-7,7'-diyl) bis(carbonate) (2.52 g, 4.09 mmol). The resulting reaction mixture was heated at reflux for 1 hour after which time consumption of starting material was evidenced by TLC analysis. The mixture was cooled to room temperature and the EtOH removed *in vacuo*. The aqueous phase was extracted with hexane (2 × 90 mL) and then acidified to pH 2 through dropwise addition of 2 M HCl, during which time a white precipitate was formed. The suspension was extracted with Et_2O (2 × 150 mL) and the combined organic phases were dried (Na_2SO_4) and concentrated *in vacuo* to yield the title compound (948 mg, 92%) as a colorless solid. This material was used the next step without further purification.

^{1}H NMR (400 MHz, $CDCl_3$): 7.17 (d, *J* = 8.0 Hz, 2H, ArH), 6.90 (dd, *J* = 7.5, 1.0 Hz, 2H, ArH), 6.69 (d, *J* = 8.0 Hz, 2H, ArH), 4.61 (br.s, 2H, OH), 3.11-2.98 (m, 4H, CH_2), 2.32 (ddd, *J* = 13.0, 7.0, 2.0 Hz,

2H, C$\underline{H}_2$), 2.25-2.16 (m, 2H, C$\underline{H}_2$). *The spectroscopic properties of this compound were consistent with the data available in the literature*.[9]

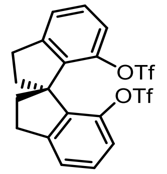

(*S*)-2,2',3,3'-Tetrahydro-1,1'-spirobi[indene]-7,7'-diyl bis(trifluoromethanesulfonate) (*S*)-S1

To a solution of (*S*)-2,2',3,3'-tetrahydro-1,1'-spirobi[indene]-7,7'-diol (2.00 g, 7.94 mmol) and pyridine (2.80 mL, 34.8 mmol) in CH_2Cl_2 (40 mL) at 0 °C was added trifluoromethanesulfonic anhydride (2.94 mL, 17.5 mmol). The reaction stirred at this temperature until consumption of starting material was observed by TLC. The mixture was then concentrated *in vacuo* and purified by FCC (40:1 hexane:EtOAc) to afford the title compound (3.48 g, 85%) as a colorless oil that solidified to a waxy solid on standing.

$[\alpha]_D^{21}$ -60.2 (c = 1.0, $CHCl_3$), [Lit.[285] $[\alpha]_D$ -64 (c = 0.5, CH_2Cl_2)].

<u>^{1}H NMR</u> (400 MHz, $CDCl_3$): 7.30-7.23 (m, 4H, Ar$\underline{H}$), 7.14-7.10 (m, 2H, Ar$\underline{H}$), 3.14-3.03 (m, 4H, C$\underline{H}_2$), 2.39-2.26 (m, 4H, C$\underline{H}_2$).

<u>^{19}F NMR</u> (376 MHz, $CDCl_3$): -74.9 (s). *The spectroscopic properties of this compound were consistent with the data available in the literature*.[10]

The majority of SIPHOX ligands employed in this study were synthesized from enantioenriched triflate **(*S*)-S1** by reported procedures.[11]

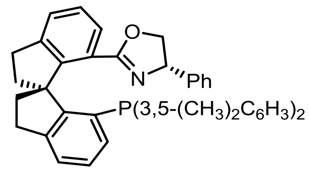

(*S*)-2-((*S*)-7'-(Bis(3,5-dimethylphenyl)phosphanyl)-2,2',3,3'-tetrahydro-1,1'-spirobi[inden]-7-yl)-4-phenyl-4,5-dihydrooxazole (S_a,*S*)-**L-2**

$[\alpha]_D^{21}$ -180 (c = 1.12, $CHCl_3$) [Lit.[11] $[\alpha]_D^{17}$ -180.9 (c = 0.5, CH_2Cl_2)].

<u>^{1}H NMR</u> (500 MHz, $CDCl_3$) 7.77 (d, *J* = 7.5 Hz, 1H, Ar$\underline{H}$), 7.40 (d, *J* = 7.5, 7.5, 1.0 Hz, 1H, Ar$\underline{H}$), 7.31-7.17 (m, 5H, Ar$\underline{H}$), 7.10 (t, *J* = 7.5 Hz, 1H, Ar$\underline{H}$), 7.00-6.94 (m, 3H, Ar$\underline{H}$), 6.86 (d, *J* = 9.5 Hz, 2H, Ar$\underline{H}$), 6.81 (d, *J* = 8.0 Hz, 2H, Ar$\underline{H}$), 6.63 (d, *J* = 7.5 Hz, 2H, Ar$\underline{H}$), 4.84 (dd, *J* = 10.0, 7.5 Hz, 1H, C$\underline{H}$), 3.62 (dd, *J* = 10.0, 7.5 Hz, 1H, C$\underline{H}_2$), 3.52 (dd, *J* = 10.0, 8.0 Hz, 1H, C$\underline{H}_2$), 3.11-2.97 (m, 3H, C$\underline{H}_2$), 2.91-2.85 (m, 1H, C$\underline{H}_2$), 2.71-2.65 (m, 1H, C$\underline{H}_2$), 2.27-2.22 (m, 1H, C$\underline{H}_2$), 2.20 (s, 6H, C$\underline{H}_3$), 2.16-2.11 (m, 8H, C$\underline{H}_2$ & C$\underline{H}_3$).

<u>^{13}C NMR</u> (125 MHz, $CDCl_3$): 165.2 ($\underline{C}$N), 154.9 (d, *J* = 25.7, Ar$\underline{C}$), 149.6 (d, *J* = 2.9, Ar$\underline{C}$), 145.2 (d, *J* = 2.6, Ar$\underline{C}$), 144.2 (d, *J* = 7.7, Ar$\underline{C}$), 142.7 (Ar$\underline{C}$), 138.4 (d, *J* = 12.8, Ar$\underline{C}$), 137.2 (d, *J* = 13.9, Ar$\underline{C}$),

137.2 (d, *J* = 6.9, ArC), 137.0 (d, *J* = 7.1, ArC), 133.5 (d, *J* = 2.5, ArC), 132.7 (d, *J* = 21.5, ArC), 131.7 (d, *J* = 20.5, ArCH), 131.3 (d, *J* = 20.2, ArCH), 129.7 (d, *J* = 7.5, ArCH), 129.0 (ArCH), 128.4 (ArCH), 127.1 (ArCH), 127.0 (ArCH), 126.5 (ArCH), 126.4 (ArCH), 126.2 (ArCH), 125.0 (d, *J* = 4.3, ArC), 124.7 (ArCH), 73.6 (CH_2), 69.2 (CH), 63.6 (d, *J* = 3.4, C), 40.6 (d, *J* = 4.4, CH_2), 38.3 (CH_2), 30.9 (CH_2), 30.7 (CH_2), 21.3 (CH_3), 21.2 (CH_3); *1 × ArC and 1 × ArCH not observed*.

^{31}P NMR (202 MHz, $CDCl_3$): -20.4 (s).

FTIR: 2863, 1641, 1579, 1452, 1353 cm^{-1}.

MS: (ESI$^+$) Found $[M+H]^+$: 606.2924, $C_{42}H_{21}NOP$ requires 606.2920.

The spectroscopic properties of this compound were consistent with the data available in the literature.[11]

(*R*)-2-Methyl-5-phenyl-2-vinyl-3,4-dihydro-2*H*-pyrrole 3a

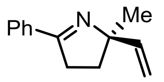

General Procedure E: Oxime ester **2a**[12] (50 mg, 0.13 mmol) was employed and the reaction was heated at 120 °C for 1.5 hours. Purification of the residue by FCC (x2, toluene:EtOAc 10:1 – 5:1 then hexane:EtOAc 5:1) afforded the title compound (15 mg, 65%, 93:7 e.r.) as a colorless oil.

$[\alpha]_D^{19}$ +31.3 (c = 0.26, $CHCl_3$).

^{1}H NMR (400 MHz, $CDCl_3$): 7.91-7.85 (m, 2H, ArH), 7.47-7.37 (m, 3H, ArH), 6.05 (dd, *J* = 17.5, 10.5 Hz, 1H, $HC=CH_2$), 5.13 (dd, *J* = 17.5, 1.0 Hz, 1H, $HC=CH_aH_b$), 5.02 (dd, *J* = 10.5, 1.0 Hz, 1H, $HC=CH_aH_b$), 3.11-2.91 (m, 2H, $Ar(CN)CH_2CH_2$), 2.15-1.84 (m, 2H, $Ar(CN)CH_2CH_2$), 1.45 (d, *J* = 1.0 Hz, 3H, $(NC)CH_3$).

^{13}C NMR (100 MHz, $CDCl_3$): 171.4 (C=N), 144.3 ($HC=CH_2$), 134.6 (ArC), 130.4, 128.4, 127.8 (3 × ArCH), 111.3 ($HC=CH_2$), 77.0 (quaternary C), 35.3 ($Ar(CN)CH_2CH_2$), 34.9 ($Ar(CN)CH_2CH_2$), 26.9 ($(NC)CH_3$).

The spectroscopic properties of this compound were consistent with the data available in the literature.[12] The enantiomeric purity of this compound was determined by chiral HPLC (Chiralpak IB, isocratic hexanes-*i*-PrOH 99:1 + 0.1% diethylamine, 0.7 mL/min, 20 °C) against a racemic standard prepared under similar conditions using $P(3,5-(CF_3)_2C_6H_3)_3$ (15 mol%) as ligand; t_R (minor) = 9.6 min and t_R (major) = 13.1 min.

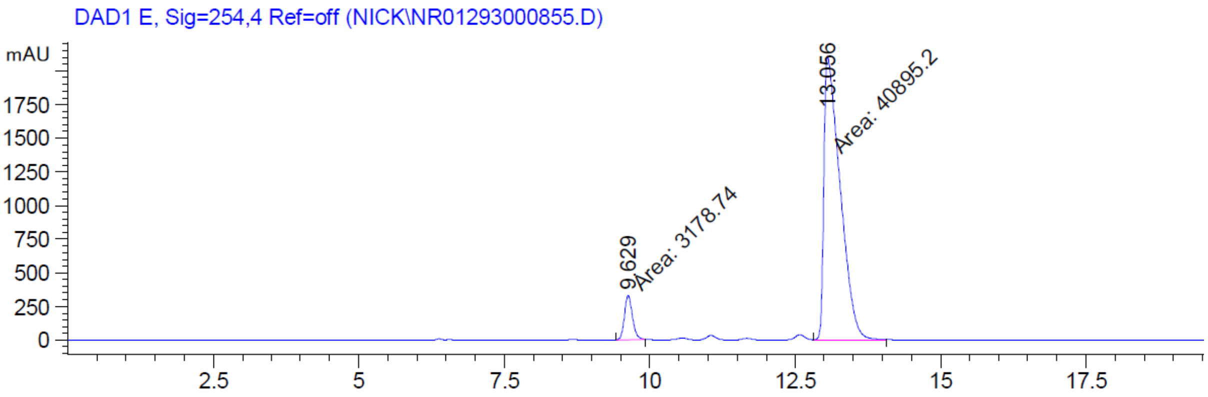

Peak #	RetTime [min]	Type	Width [min]	Area [mAU*s]	Height [mAU]	Area %
1	9.629	MM	0.1607	3178.73779	329.63705	7.2123
2	13.056	MM	0.3229	4.08952e4	2110.92603	92.7877

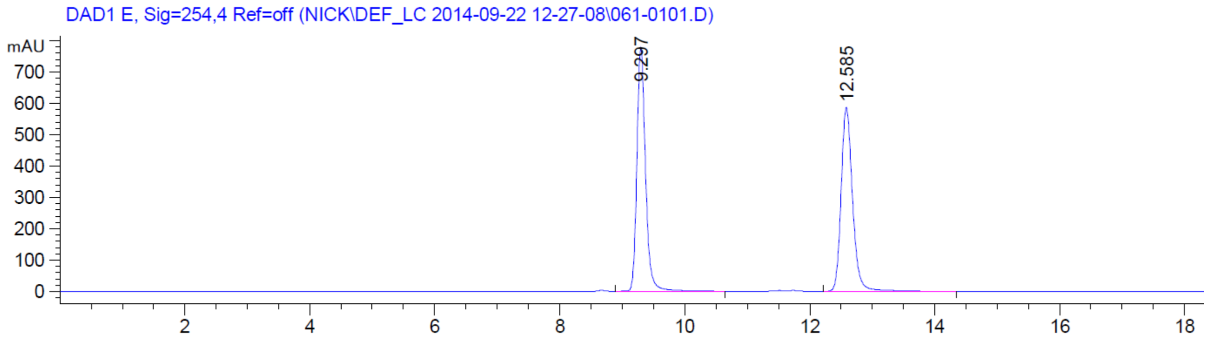

Peak #	RetTime [min]	Type	Width [min]	Area [mAU*s]	Height [mAU]	Area %
1	9.297	BB	0.1478	7440.51074	776.63190	49.9720
2	12.585	BB	0.1942	7448.84326	586.23016	50.0280

(*E*)-4-Methyl-1-(naphthalen-2-yl)hex-4-en-1-one

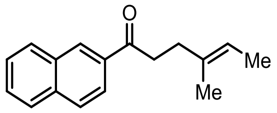

To a solution of 2-acetonaphthone (2.00 g, 11.8 mmol) and *N*,*N’*-dimethylhydrazine (1.07 mL, 14.1 mmol) in benzene (10 mL) was added a few drops of trifluoroacetic acid. The reaction mixture was then heated at reflux under Dean-Stark conditions until consumption of starting material was observed by TLC analysis. The mixture was then concentrated *in vacuo* to afford the title compound (2.38 g, 95%) as a yellow oil that solidified on standing. This material was used without further purification. **^{1}H NMR** (400 MHz, $CDCl_3$): 7.98 (1H, br.s, ArH), 7.94 (J = 6.5, 2.0 Hz, 1H, dd, ArH), 7.78-7.69 (3H, m, ArH), 7.39-7.35 (2H, m, ArH), 2.57 (6H, s, $N(CH_3)_2$), 2.36 (3H, s, $(CN)CH_3$). To a solution of 1,1-dimethyl-

2-(1-(naphthalen-2-yl)ethylidene)hydrazine (500 mg, 2.36 mmol) in THF (11 mL) at -78 °C was added *n*-BuLi (1.52 M in hexane, 1.68 mL, 2.55 mmol) dropwise *via* syringe over 2 minutes. After stirring at this temperature for 30 minutes, (*E*)-1-bromo-2-methylbut-2-ene[12] (419 mg, 2.83 mmol) was added *via* syringe in one portion. The reaction was stirred at this temperature for a further 90 minutes, after which time it was quenched by addition of 1 M HCl (10 mL). It was warmed to room temperature and hydrolysis of the intermediate hydrazone was monitored by TLC. After stirring for 90 minutes, the phases were separated and the aqueous phase with extracted with EtOAc (50 mL). The combined organic phases were washed with brine (50 mL), dried (Na_2SO_4), filtered and concentrated *in vacuo*. Purification of the residue by FCC (hexane:EtOAc 30:1) afforded the title compound (427 mg, 76%) as a pale yellow oil.

^{1}H NMR (400 MHz, $CDCl_3$): 8.48 (br.s, 1H, Ar$\underline{H}$), 8.04 (dd, *J* = 8.5, 2.0 Hz, 1H, Ar$\underline{H}$), 7.97 (d, *J* = 8.0 Hz, 1H, Ar$\underline{H}$), 7.91-7.87 (m, 2H, Ar$\underline{H}$), 7.62-7.53 (m, 2H, Ar$\underline{H}$), 5.34-5.28 (m, 1H, C=C$\underline{H}$), 3.22-3.18 (m, 2H, Ar(CN)C$\underline{H_2}$), 2.50-2.46 (m, 2H, Ar(CN)CH_2C$\underline{H_2}$), 1.70 (t, *J* = 1.0 Hz, 3H, C(C$\underline{H_3}$)=$CHCH_3$), 1.60 (dq, *J* = 6.5, 1.0 Hz, 3H, C(CH_3)=CHC$\underline{H_3}$).

^{13}C NMR (100 MHz, $CDCl_3$): 200.1 ($\underline{C}$=O), 135.5 (Ar$\underline{C}$), 134.6 (quaternary $\underline{C}$), 134.3 (Ar$\underline{C}$), 132.5 (Ar$\underline{C}$), 129.6, 129.5, 128.4, 128.3, 127.7, 126.7, 123.9 (7 × Ar$\underline{C}$H), 119.1 (C=$\underline{C}$H), 37.5 (Ar(CN)$\underline{C}H_2$), 34.1 (Ar(CN)$CH_2$$\underline{C}H_2$), 15.9 (C($\underline{C}H_3$)=$CHCH_3$), 13.4 (C=CH$\underline{C}H_3$).

FTIR: 2920, 1680, 1627, 1468, 1357, 1294, 1123 cm^{-1}.

MS: (ESI^+) Found $[M+Na]^+$: 261.1251, $C_{17}H_{18}ONa$ requires 261.1250.

(4*E*)-4-Methyl-1-(naphthalen-2-yl)hex-4-en-1-one *O*-perfluorobenzoyl oxime 2b

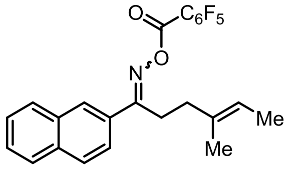

General Procedure D: Part A: (*E*)-4-Methyl-1-(naphthalen-2-yl)hex-4-en-1-one (415 mg, 1.74 mmol) was employed affording the corresponding oxime (430 mg, 97%) as a colorless solid. Part B: The corresponding oxime (430 mg, 1.70 mmol) was employed. Purification of the residue by FCC (hexane:EtOAc 30:1) afforded the title compound (477 mg, 63%, 13:1 mixture of oxime isomers) as a colorless solid.

^{1}H NMR (400 MHz, $CDCl_3$): *Signals for the major isomer only* 8.18-8.17 (m, 1H, Ar$\underline{H}$), 7.92-7.86 (m, 4H, Ar$\underline{H}$), 7.58-7.52 (m, 2H, Ar$\underline{H}$), 5.27-5.22 (m, 1H, C=C$\underline{H}$), 3.12-3.08 (m, 2H, Ar(CN)C$\underline{H_2}$), 2.32-2.28 (m, 2H, Ar(CN)CH_2C$\underline{H_2}$), 1.62 (tq, *J* = 1.0, 1.0 Hz, 3H, C(C$\underline{H_3}$)=$CHCH_3$), 1.56-1.54 (m, 3H, C(CH_3)=CHC$\underline{H_3}$).

^{13}C NMR (100 MHz, $CDCl_3$): 168.5 ($\underline{C}$=N), 156.6 ($\underline{C}$O), 134.5 (Ar$\underline{C}$), 133.7 (quaternary $\underline{C}$), 132.9 (Ar$\underline{C}$), 130.5 (Ar$\underline{C}$), 128.8, 128.6, 128.0, 127.7, 127.6, 126.7, 124.0 (7 × Ar$\underline{C}$H), 120.3 (C=$\underline{C}$H), 36.5 (Ar(CN)$CH_2$$\underline{C}H_2$), 27.8 (Ar(CN)$\underline{C}H_2$), 15.5 (C($\underline{C}H_3$)=$CHCH_3$), 13.4 (C=CH$\underline{C}H_3$); *Signals*

corresponding to the perfluorinated benzoyl ester moiety were not resolvable due to their anticipated weak intensity.

δ_F (376 MHz, $CDCl_3$) -136.9 (2F), -137.4 (0.15F), -147.5 (1F), -148.1 (0.07F), -159.7 (2F), -160.0 (0.15F).

FTIR: 2992, 2903, 1756, 1650, 1526, 1496, 1323 cm^{-1}.

MS: (ESI^+) Found $[M+H]^+$: 448.1321, $C_{24}H_{19}F_5NO_2$ requires 448.1330.

(*R*)-2-Methyl-5-(naphthalen-2-yl)-2-vinyl-3,4-dihydro-2*H*-pyrrole 3b

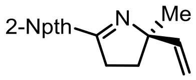

General Procedure E: Oxime ester **2b** (50 mg, 0.11 mmol) was employed and the reaction was heated at 80 °C for 5.5 hours. Purification of the residue by FCC (x2, toluene:EtOAc 15:1 – 5:1 then hexane:EtOAc 5:1) afforded the title compound (15.5 mg, 60%, 93:7 e.r.) as a colorless solid.

$[\alpha]_D^{21}$ +32.4 (c = 0.68, $CHCl_3$).

M.P. 73-74° (CH_2Cl_2-pentane).

^{1}H NMR (400 MHz, $CDCl_3$): 8.20 (br.s, 1H, Ar$\underline{H}$), 8.12 (dd, *J* = 8.5, 1.5 Hz, 1H, Ar$\underline{H}$), 7.91-7.84 (m, 3H, Ar$\underline{H}$), 7.54-7.48 (m, 2H, Ar$\underline{H}$), 6.11 (dd, *J* = 17.5, 10.5 Hz, 1H, $\underline{H}C=CH_2$), 5.16 (dd, *J* = 17.5, 1.5 Hz, 1H, $HC=CH_a\underline{H_b}$), 5.03 (dd, *J* = 10.5, 1.5 Hz, 1H, $HC=C\underline{H_a}H_b$), 3.21-3.05 (m, 2H, Ar(CN)$C\underline{H_2}CH_2$), 2.14 (ddd, *J* = 12.5, 9.0, 6.0 Hz, 1H, Ar(CN)$CH_2C\underline{H_2}$), 1.94 (ddd, *J* = 12.5, 9.0, 7.0 Hz, 1H, Ar(CN)$CH_2C\underline{H_2}$), 1.48 (s, 3H, (NC)$C\underline{H_3}$).

^{13}C NMR (100 MHz, $CDCl_3$): 171.3 ($\underline{C}$=N), 144.3 ($H\underline{C}=CH_2$), 134.4, 132.9, 132.1 (3 × Ar$\underline{C}$), 128.7, 128.1, 128.0, 127.7, 127.0, 126.3, 124.8 (7 × Ar$\underline{C}$H), 111.4 ($HC=\underline{C}H_2$), 76.8 (quaternary $\underline{C}$), 35.4 (Ar(CN)$CH_2\underline{C}H_2$), 34.9 (Ar(CN)$\underline{C}H_2CH_2$), 27.0 ((NC)$\underline{C}H_3$).

FTIR: 2961, 1612, 1447, 1405, 1351, 1296 cm^{-1}.

MS: (ESI^+) Found $[M+H]^+$: 236.1429, $C_{17}H_{18}N$ requires 236.1434.

The enantiomeric purity of this compound was determined by chiral HPLC (Chiralpak IB, isocratic hexanes-*i*-PrOH-diethylamine 99:1:0.1, 0.7 mL/min, 20 °C) against a racemic standard prepared under similar conditions using $P(3,5-(CF_3)_2C_6H_3)_3$ (15 mol%) as ligand; t_R (minor) = 8.2 min and t_R (major) = 10.9 min. Crystals suitable for X-ray diffraction were grown by vapour diffusion of pentane into a concentrated CH_2Cl_2 solution of **3b** at 5 °C. The absolute structure of this compound was determined by X-ray crystallography.

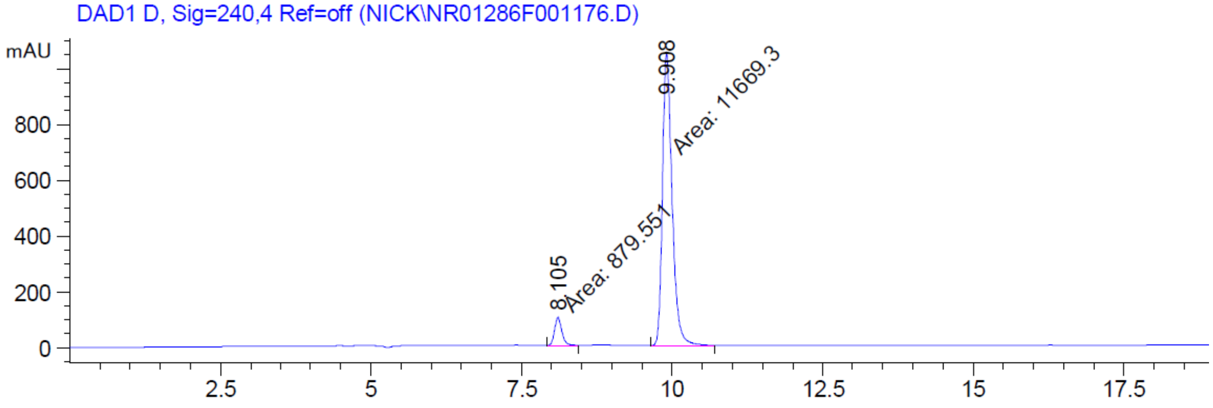

Peak #	RetTime [min]	Type	Width [min]	Area [mAU*s]	Height [mAU]	Area %
1	8.105	MM	0.1449	879.55078	101.19036	7.0090
2	9.908	MM	0.1856	1.16693e4	1048.00830	92.9910

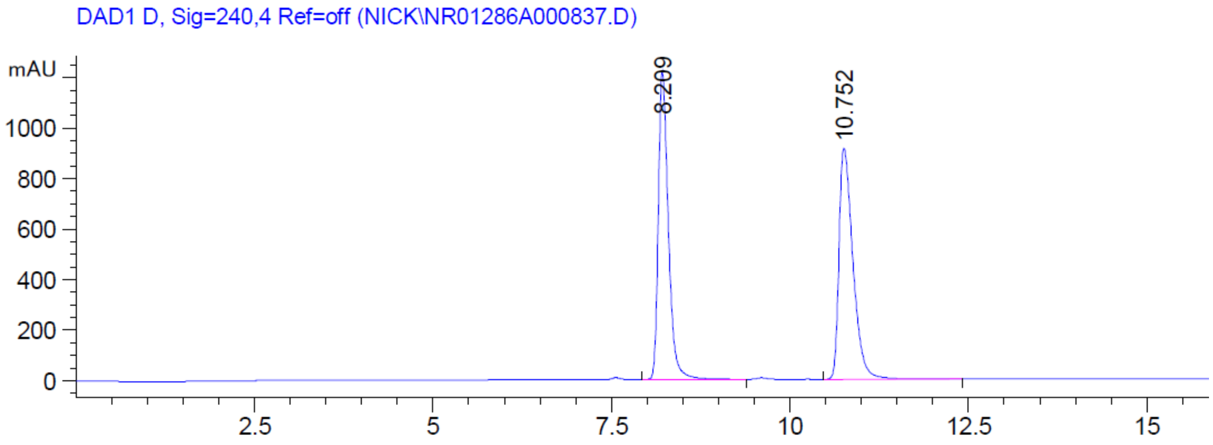

Peak #	RetTime [min]	Type	Width [min]	Area [mAU*s]	Height [mAU]	Area %
1	8.209	BV	0.1501	1.19238e4	1219.00391	49.0368
2	10.752	BB	0.2062	1.23923e4	914.08649	50.9632

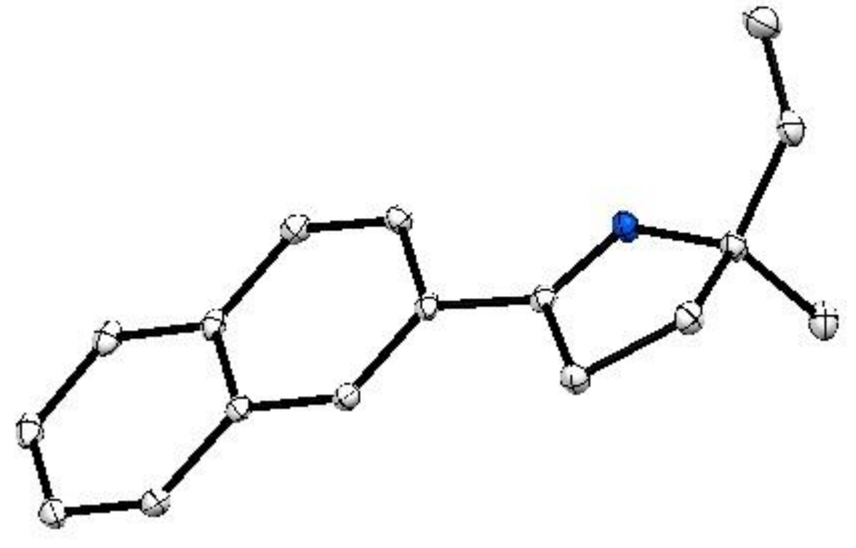

ORTEP view of dihydropyrrole (*R*)-**3b**, ellipsoids at 30%, hydrogen atoms omitted for clarity; Crystal data for dihydropyrrole (*R*)-**3b**: $C_{17}H_{17}NO$, MW, 235.31, orthorhombic, space group $P2_12_12_1$, a = 6.0770(11) Å, b = 7.7665(14) Å, c = 27.619(5) Å, V = 1303.5(4) $Å^3$, α = 90°, β = 90°, γ = 90°, Z = 4, D_c = 1.199 g/cm^3, CuKα radiation, λ = 1.54178 Å, μ = 0.525 mm^{-1}, T = 100 K, crystal size 0.456 × 0.288 × 0.281 mm, Microstar, 12133 reflections collected, 2295 were unique, R_{int} = 0.0359; R_1 = 0.0273 and wR_2 = 0.0711, GOF = 1.076 for 164 refined parameters. Flack parameter = -0.04(10).

(*R*,*E*)-2-Methyl-5-(naphthalen-2-yl)-2-(pent-1-en-1-yl)-3,4-dihydro-2*H*-pyrrole 3c

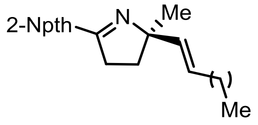

General Procedure E: Oxime ester **2c**[12] (50.0 mg, 0.102 mmol) was employed and the reaction was heated at 120 °C for 2 hours. FCC (toluene:EtOAc 16:1) afforded the title compound (21.0 mg, 74%, 20:1 r.r., 91:9 e.r.) as a pale yellow oil.

$[\alpha]_D^{23}$ +25.9 (c = 0.46, $CHCl_3$).

^{1}H NMR (400 MHz, $CDCl_3$): 8.24-8.06 (m, 2H, ArH), 7.91-7.79 (m, 3H, ArH), 7.58-7.44 (m, 2H, ArH), 5.71 (dt, *J* = 15.5 and 1.0 Hz, 1H, HC=CHCH$_2$), 5.56 (dt, *J* = 15.5 and 6.5 Hz, 1H, HC=CHCH$_2$), 3.20-3.03 (m, 2H, Ar(CN)CH$_2$), 2.15-2.07 (m, 1H, Ar(CN)CH$_2$CH$_2$), 2.03 (m, 2H, C=CHCH$_2$), 1.98-1.88 (m, 1H, Ar(CN)CH$_2$CH$_2$), 1.47 (s, 3H, (NC)CH$_3$), 1.41 (tq, *J* = 7.5 and 7.5 Hz, 2H, C=CHCH$_2$CH$_2$), 0.90 (t, *J* = 7.5 Hz, 3H, C=CH(CH$_2$)$_2$CH$_3$).

^{13}C NMR (100 MHz, $CDCl_3$): 170.8 (C=N), 136.3 (C=CH), 134.3, 133.0, 132.3 (3 × ArC), 128.7, 128.0 (2 signals), 127.7, 127.3 (5 × ArCH), 126.9 (C=CH), 126.3, 124.8 (2 × ArCH), 76.3 (quaternary C), 36.1 (Ar(CN)CH$_2$), 34.9 (Ar(CN)CH$_2$CH$_2$), 34.6 (C=CHCH$_2$), 27.4 ((NC)CH$_3$), 22.5 (C=CHCH$_2$CH$_2$), 13.7 (C=CH(CH$_2$)$_2$CH$_3$).

Spectroscopic properties were identical to that described previously for the racemic material.[12]

The enantiomeric purity of this compound was determined by chiral HPLC (Chiralpak IB, isocratic hexane-*i*-PrOH (95:5, 0.5 mL/min, 20 °C) against a racemic standard prepared under similar conditions using $P(3,5\text{-}(CF_3)_2C_6H_3)_3$ (15 mol%) as ligand; t_R (minor) = 8.6 min and t_R (major) = 10.0 min.

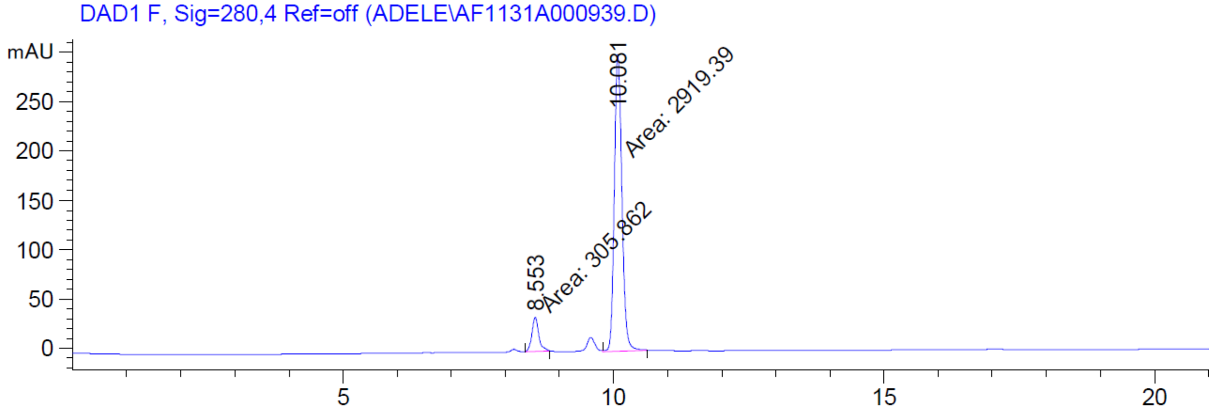

Peak #	RetTime [min]	Type	Width [min]	Area [mAU*s]	Height [mAU]	Area %
1	8.553	MM	0.1475	305.86169	34.56162	9.4833
2	10.081	MM	0.1615	2919.39478	301.20853	90.5167

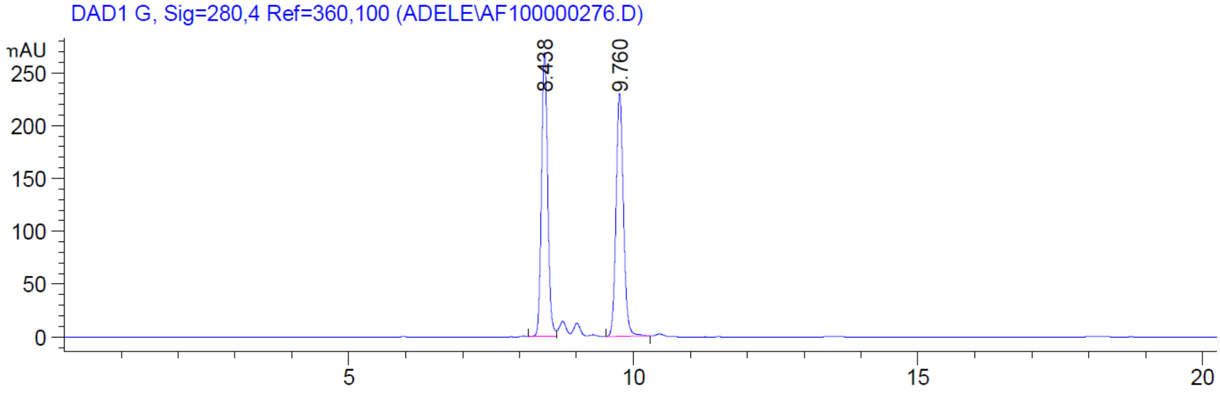

Peak #	RetTime [min]	Type	Width [min]	Area [mAU*s]	Height [mAU]	Area %
1	8.438	BV	0.1172	2055.91431	268.80472	49.6845
2	9.760	BB	0.1393	2082.02515	230.67490	50.3155

(*E*)-4-Methyl-1,6-diphenylhex-4-en-1-one

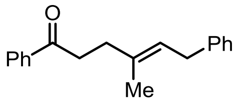

General Procedure C: Ethyl benzoylacetate (0.42 mL, 2.44 mmol) and (*E*)-(4-bromo-3-methylbut-2-en-1-yl)benzene[13] (600 mg, 2.68 mmol) were employed. Purification of the residue by FCC (hexane:EtOAc 40:1) afforded the title compound (444 mg, 69%) as a colorless oil.

^{1}H NMR (400 MHz, $CDCl_3$): 7.98-7.95 (m, 2H, ArH), 7.58-7.54 (m, 1H, ArH), 7.48-7.44 (m, 2H, ArH), 7.31-7.26 (m, 2H, ArH), 7.21-7.16 (m, 3H, ArH), 5.44-5.39 (m, 1H, C=CH), 3.38 (d, *J* = 7.5 Hz, 2H, C=CHCH_2Ar), 3.13-3.09 (m, 2H, Ar(CO)CH_2), 2.51-2.47 (m, 2H, Ar(CO)CH_2CH_2), 1.79 (d, *J* = 1.5 Hz, 3H, CH_3C=CH).

^{13}C NMR (100 MHz, $CDCl_3$): 200.0 (C=O), 141.4 (ArC), 137.0 (ArC), 135.0 (C=CH), 132.9, 128.5, 128.3 (2C), 128.0, 125.7 (6 × ArCH), 123.6 (C=CH), 37.3 (Ar(CO)CH_2), 34.2 (C=CHCH_2Ar), 34.0 (Ar(CO)CH_2CH_2), 16.4 (CH_3C=CH).

FTIR: 2991, 1681, 1597, 1491, 1446, 1202 cm^{-1}.

MS: (ESI$^+$) Found [M+H]$^+$: 265.1588, $C_{19}H_{21}O$ requires 265.1587.

(4*E*)-4-Methyl-1,6-diphenylhex-4-en-1-one *O*-perfluorobenzoyl oxime 2d

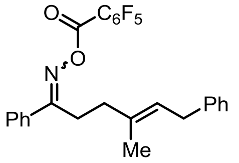

General Procedure D: Part A: (*E*)-4-Methyl-1,6-diphenylhex-4-en-1-one (419 mg, 1.59 mmol) was employed affording the corresponding oxime (436 mg, 98%) as a colorless solid. Part B: The corresponding oxime (420 mg, 1.51 mmol) was employed. Purification of the residue by FCC (hexane:EtOAc 50:1 – 25:1) afforded the title compound (481 mg, 68%, 10:1 mixture of oxime isomers) as a yellow oil that solidified on standing.

^{1}H NMR (400 MHz, $CDCl_3$): *Data for major isomer* 7.71-7.68 (m, 2H, ArH), 7.49-7.35 (m, 3H, ArH), 7.29-7.24 (m, 2H, ArH), 7.20-7.11 (m, 3H, ArH), 5.33 (tq, *J* = 7.5, 1.5 Hz, 1H, C=CH), 3.31 (d, *J* = 7.5 Hz, 2H, C=CHCH_2Ar), 3.05-3.01 (m, 2H, Ar(CN)CH_2), 2.32-2.27 (m, 2H, Ar(CN)CH_2CH_2), 1.71 (br.s, 3H, CH_3C=CH); *Diagnostic signals for minor isomer* 2.91-2.87 (m, 0.2H, Ar(CN)CH_2’), 2.25-2.21 (m, 0.2H, Ar(CN)CH_2CH_2’).

^{13}C NMR (100 MHz, $CDCl_3$): *Data for major isomer* 168.6 (C=N), 156.5 (C=O), 141.1 (ArC), 134.0 (C=CH), 133.1 (ArC), 131.0, 128.8, 128.4, 128.2, 127.5, 125.9 (6 × ArCH), 124.9 (C=CH), 36.3 (Ar(CN)CH_2CH_2), 34.2 (C=CHCH_2Ar), 27.8 (Ar(CN)CH_2), 16.0 (CH_3C=CH). *Signals corresponding to the perfluorinated benzoyl ester moiety were not resolvable due to their anticipated weak intensity.*

^{19}F NMR (376 MHz, $CDCl_3$): -137.0 (2F), -137.2 (0.2F), -147.4 (1F), -148.0 (0.1F), -159.7 (2F), -160.0 (0.2F).

FTIR: 2992, 2915, 1756, 1654, 1526, 1497, 1324, 1197 cm^{-1}.

MS: (ESI$^+$) Found $[M+Na]^+$: 496.1301, $C_{26}H_{20}F_5NO_2Na$ requires 496.1306.

(*R*,*E*)-2-Methyl-5-phenyl-2-styryl-3,4-dihydro-2*H*-pyrrole 3d

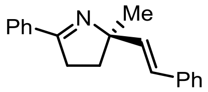

General Procedure E: Oxime ester **2d** (50 mg, 0.11 mmol) was employed and the reaction was heated at 100 °C for 2.5 hours. Purification of the residue by FCC (x2, toluene:EtOAc 20:1 – 10:1 then hexane:EtOAc 5:1) afforded the title compound (22 mg, 80%, 95: 5 e.r.) as a colorless oil.

$[\alpha]^{21}_{D}$ +65.5 (c = 0.84, $CHCl_3$).

^{1}H NMR (400 MHz, $CDCl_3$): 7.93-7.90 (m, 2H, ArH), 7.46-7.37 (m, 5H, ArH), 7.32-7.27 (m, 2H, ArH), 7.22-7.18 (m, 1H, ArH), 6.50 (d, *J* = 16.0 Hz, 1H, CH=CHAr), 6.45 (d, *J* = 16.0 Hz, 1H, CH=CHAr), 3.13-2.98 (m, 2H, Ar(CN)CH_2), 2.18 (ddd, *J* = 12.5, 9.0, 6.0 Hz, 1H, Ar(CN)CH_2CH_2), 2.00 (ddd, *J* = 12.5, 9.0, 7.0 Hz, 1H, Ar(CN)CH_2CH_2), 1.54 (s, 3H, (NC)CH_3).

^{13}C NMR (100 MHz, $CDCl_3$): 171.4 (C=N), 137.4 (ArC), 136.4 (CH=CHAr), 134.6 (ArC), 130.5, 128.4 (2C), 127.8, 127.1 (5 × ArCH), 126.5 (CH=CHAr), 126.3 (ArCH), 76.4 (quaternary C), 36.0 (Ar(CN)CH_2CH_2), 35.0 (Ar(CN)CH_2CH_2), 27.5 ((NC)CH_3).

FTIR: 2962, 1610, 1450, 1447, 1339 cm^{-1}.

MS: (ESI$^+$) Found $[M+H]^+$: 262.1588, $C_{19}H_{20}N$ requires 262.1590. The enantiomeric purity of this compound was determined by chiral HPLC (Chiralpak IB, isocratic hexanes-*i*-PrOH 90:10, 0.5 mL/min, 20 °C) against a racemic standard prepared under similar conditions using $P(3,5-(CF_3)_2C_6H_3)_3$; t_R (minor) = 8.6 min and t_R (major) = 13.0 min. The absolute structure of this molecule was confirmed by Vibrational Circular Dichroism (VCD).

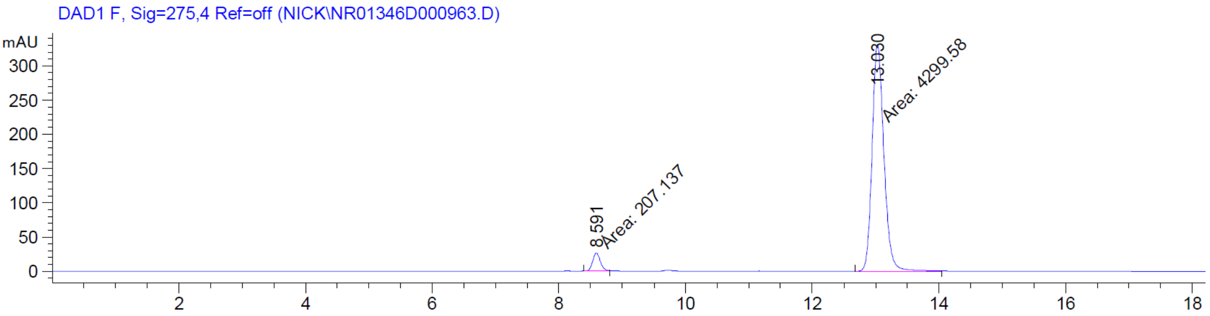

Peak #	RetTime [min]	Type	Width [min]	Area [mAU*s]	Height [mAU]	Area %
1	8.591	MM	0.1344	207.13673	25.69152	4.5962
2	13.030	MM	0.2161	4299.57617	331.62375	95.4038

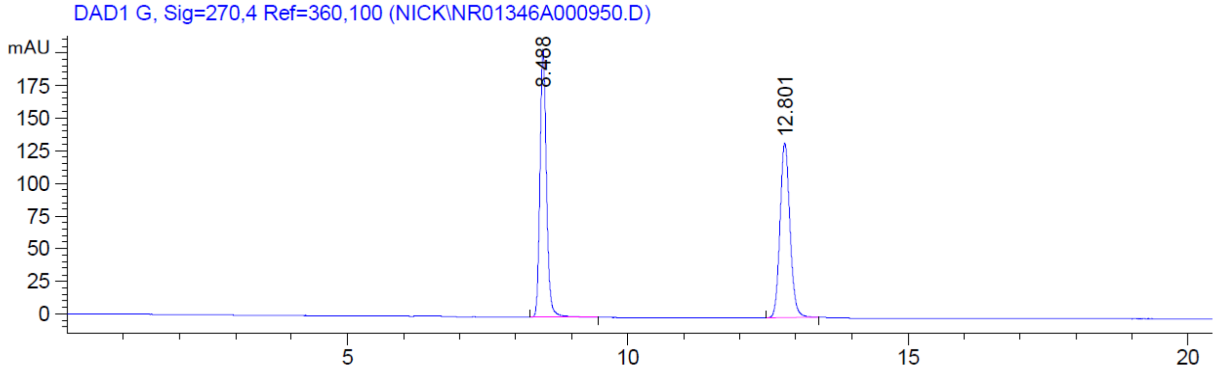

Peak #	RetTime [min]	Type	Width [min]	Area [mAU*s]	Height [mAU]	Area %
1	8.488	BB	0.1250	1671.07813	205.22136	50.3005
2	12.801	BB	0.1908	1651.10999	133.97676	49.6995

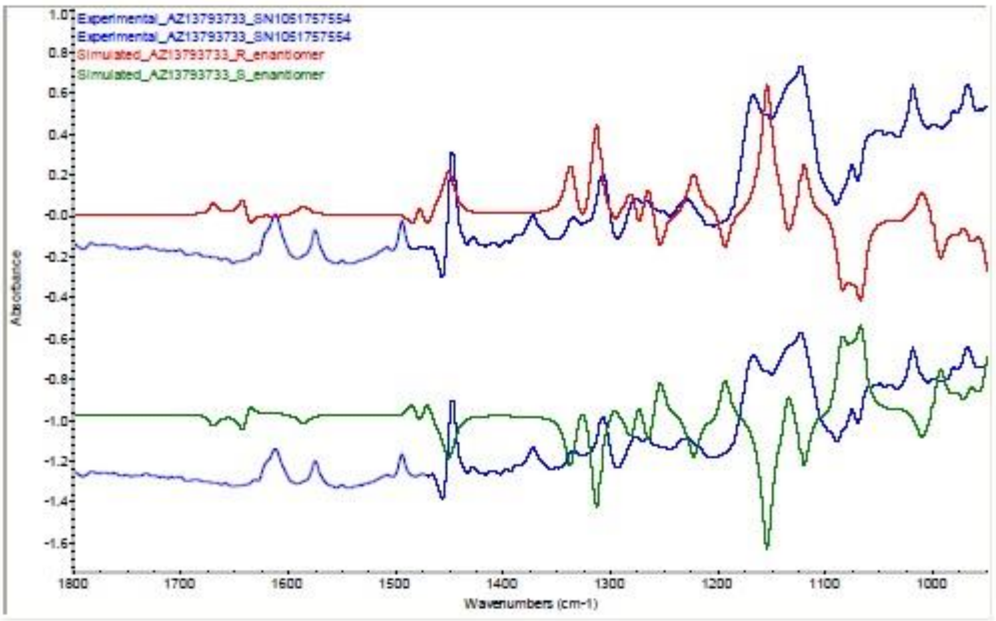

Comparison of experimental VCD spectrum of dihydropyrrole **3d** *(in duplicate, blue) and simulated spectra of the R (red) and S (green) enantiomers. The agreement between the simulated spectrum of the R enantiomer and the experimental spectrum is good, therefore dihydropyrrole* **3d** *has R configuration.*

Experimental: A 13.1 mg sample of dihydropyrrole **3d** was dissolved in $CDCl_3$. A VCD spectrum was acquired in 0.100 mm BaF_2 cells for 7 hours each in a BioTools ChiralIR instrument equipped with dual source and dual photoelastic modulator. The resolution was 4 cm^{-1}. The experimental VCD spectrum shows weak characteristic bands.

Computational Spectral Simulations: A Monte Carlo molecular mechanics search for low energy geometries was conducted for full structures of the two enantiomers, *R* and *S*. *MacroModel* within the *Maestro* graphical interface (Schrödinger Inc.) was used to generate starting coordinates for conformers. All conformers within 5 kcal/mole of the lowest energy conformer were used as starting points for density functional theory (DFT) minimizations within *Gaussian09*. Optimized structures, harmonic

vibrational frequencies/intensities, VCD rotational strengths, and free energies at STP (including zero-point energies) were determined for each conformer. In these calculations, the functional B3LYP and the basis set 6-31G* were used. Simulations of infrared and VCD spectra for each conformation were generated using an in-house built program to fit Lorentzian line shapes (12 cm^{-1} line width) to the computed spectra thereby allowing direct comparisons between simulated and experimental spectra.

Results: The experimental spectrum was compared with simulated spectra of the two enantiomers based on DFT calculations starting with full structures. The comparison is presented above. The agreement between the simulated spectrum of the *R* enantiomer and the experimental spectrum is good. It is therefore concluded that dihydropyrrole **3d** has *R* configuration.

2-(4-Methoxyphenyl)acetaldehyde

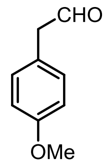

To a solution of methyl 4-methoxyphenylacetate (4.40 mL, 27.8 mmol) in anhydrous toluene (50 mL) at -78 °C was added dropwise diisobutylaluminium hydride (1 M in toluene, 33.4 mL, 33.4 mmol). The reaction was stirred at this temperature for 1 hour. The reaction was quenched with MeOH (5 mL) and then poured into CH_2Cl_2 (150 mL), then washed sequentially with 1 M HCl (20 mL), brine (20 mL). The organic phase was concentrated *in vacuo*. FCC (hexane:EtOAc 15:1) afforded the title compound (3.46 g, 83%) as a colorless oil.

^{1}H NMR (400 MHz, $CDCl_3$): 9.73 (t, *J* = 2.5 Hz, 1H, (CO)H), 7.14 (d, *J* = 8.0 Hz, 2H, ArH), 6.92 (d, *J* = 8.0 Hz, 2H, ArH), 3.82 (s, 3H, OCH_3), 3.64 (d, *J* = 2.5 Hz, 2H, CH_2).

^{13}C NMR (100 MHz, $CDCl_3$): 199.6 (C=O), 159.0 (ArC), 130.7 (ArCH), 123.7 (ArC), 114.4 (ArCH), 55.3 (OCH_3), 49.7 (CH_2).

The spectroscopic properties of this compound were consistent with the data in the literature.[14]

Ethyl (*E*)-4-(4-methoxyphenyl)-2-methylbut-2-enoate

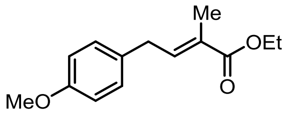

To a solution of 2-(4-methoxyphenyl)acetaldehyde (1.80 g, 12.00 mmol) in CH_2Cl_2 (40 mL) at 0 °C was added a solution of ethyl 2-(triphenylphosphoranylidene)butanoate (5.20 g, 14.40 mmol) in CH_2Cl_2 (30 mL). The reaction was stirred at room temperature for 16 hours and then concentrated *in vacuo*. The oil was taken up in hexane (200 mL) and the precipitate filtered off. The filtrate was concentrated *in vacuo*. FCC (hexane:EtOAc 70:1 to 40:1) afforded the title compound (2.12 g, 75%) as a colorless oil.

^{1}H NMR (400 MHz, $CDCl_3$): 7.13 (d, *J* = 8.0 Hz, 2H, ArH), 6.93-6.83 (m, 3H, ArH and HC=C), 4.19 (q, *J* = 7.0 Hz, 2H, $C(O)OCH_2CH_3$), 3.80 (s, 3H, OCH_3), 3.47 (d, *J* = 7.5 Hz, 2H, $ArCH_2CH$), 1.97-1.94 (m, 3H, $HC{=}CCH_3$), 1.29 (t, *J* = 7.0 Hz, 3H, $C(O)OCH_2CH_3$).
^{13}C NMR (100 MHz, $CDCl_3$): 168.1 (C=O), 158.2 (ArC), 140.4 (HC=C), 131.0 (ArC), 129.4 (ArCH), 128.2 (HC=C), 114.1 (ArCH), 60.5 ($C(O)OCH_2CH_3$), 55.3 (OCH_3), 34.0 ($ArCH_2CH$), 14.3 ($C(O)OCH_2CH_3$), 12.5 ($HC{=}CCH_3$),
FTIR: 1708, 1511, 1247 cm^{-1}.
MS: (ESI^+) Found $[M+H]^+$: 235.1325, $C_{14}H_{19}O_3$ requires 235.1329.

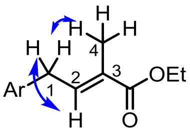

To confirm the olefin geometry, nOe analysis was carried out. An nOe enhancement between C1-H_2 and C2-H was observed and nOe was observed between C1-H_2 and C4-H_2, which is consistent with an (E)-olefin geometry.

(*E*)-4-(4-Methoxyphenyl)-2-methylbut-2-en-1-ol

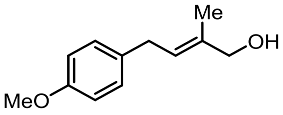

General Procedure A: In a modification to the general procedure $LiAlH_4$ (1M in Et_2O) was employed with ethyl (*E*)-4-(4-methoxyphenyl)-2-methylbut-2-enoate (2.00 g, 8.55 mmol). FCC (hexane:EtOAc 5:1 to 4:1) afforded the title compound (1.50 g, 91%) as a colorless oil.
^{1}H NMR (400 MHz, $CDCl_3$): 7.11 (d, *J* = 8.0 Hz, 2H, ArH), 6.84 (d, *J* = 8.0 Hz, 2H, ArH), 5.61 (tq, *J* = 7.5 and 1.5 Hz, 1H, HC=C), 4.06 (d, *J* = 3.5 Hz, 2H, CH_2OH), 3.80 (s, 3H, OCH_3), 3.35 (d, *J* = 7.5 Hz, 2H, $ArCH_2CH$), 1.79 (s, 3H, $HC{=}CCH_3$), 1.37 (t, *J* = 3.5 Hz, 1H, OH).
^{13}C NMR (100 MHz, $CDCl_3$): 157.9 (ArC), 135.4 (HC=C), 133.0 (ArC), 129.2 (ArCH), 125.1 (HC=C), 113.9 (ArCH), 68.8 (CH_2OH), 55.3 (OCH_3), 33.0 ($ArCH_2CH$), 13.8 ($HC{=}CCH_3$).
FTIR: 3324, 2910, 1509, 1124 cm^{-1}.
MS: (ESI^+) Found $[M+H-H_2O]^+$: 175.1120, $C_{12}H_{15}O$ requires 175.1117.

(*E*)-1-(4-Bromo-3-methylbut-2-en-1-yl)-4-methoxybenzene

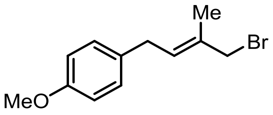

General Procedure B: (*E*)-4-(4-Methoxyphenyl)-2-methylbut-2-en-1-ol (1.45 g, 7.56 mmol) was employed to afford the title compound (1.26 g, 66%) as a colorless oil.

^{1}H NMR (400 MHz, $CDCl_3$): 7.08 (d, J = 8.0 Hz, 2H, ArH), 6.85 (d, J = 8.0 Hz, 2H, ArH), 5.78 (t, J = 7.5 Hz, 1H, HC=C), 4.02 (s, 2H, CH_2Br), 3.80 (s, 3H, OCH_3), 3.34 (d, J = 7.5 Hz, 2H, $ArCH_2CH$), 1.88 (s, 3H, $HC{=}CCH_3$).

^{13}C NMR (100 MHz, $CDCl_3$): 158.0 (ArC), 132.6 (HC=C), 132.1 (ArC), 130.1 (HC=C), 129.2, 114.0 (2 × ArCH), 55.3 (OCH_3), 41.4 (CH_2Br), 33.6 ($ArCH_2CH$), 14.8 ($HC{=}CCH_3$).

FTIR: 1510, 1246 cm^{-1}.

MS: (CI^+) Found $[M+H]^+$: 255.0378, $C_{12}H_{16}O^{79}Br$ requires 255.0385.

(*E*)-6-(4-Methoxyphenyl)-4-methyl-1-phenylhex-4-en-1-one

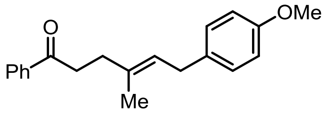

General Procedure C: Ethyl benzoylacetate (0.71 mL, 4.10 mmol) and (*E*)-1-(4-bromo-3-methylbut-2-en-1-yl)-4-methoxybenzene (1.20 g, 4.72 mmol) were employed. FCC (hexane:EtOAc 40:1) afforded the title compound (1.09 g, 90%) as a colorless oil.

^{1}H NMR (400 MHz, $CDCl_3$): 7.97 (d, J = 7.5 Hz, 2H, ArH), 7.57 (t, J = 7.5 Hz, 1H, ArH), 7.47 (t, J = 7.5 Hz, 2H, ArH), 7.08 (d, J = 8.0 Hz, 2H, ArH), 6.83 (d, J = 8.0 Hz, 2H, ArH), 5.39 (tq, J = 7.5 and 1.0 Hz, 1H, C=CH), 3.80 (s, 3H, OCH_3), 3.31 (d, J = 7.5 Hz, 2H, $C{=}CHCH_2Ar$), 3.10 (t, J = 7.5 Hz, 2H, $ArC(O)CH_2$), 2.48 (t, J = 7.5 Hz, 2H, $ArC(O)CH_2CH_2$), 1.79 (s, 3H, $CH_3C{=}CH$).

^{13}C NMR (100 MHz, $CDCl_3$): 200.0 (C=O), 157.8, 137.0 (2 × ArC), 134.7 (C=CH), 133.5 (ArC), 132.9, 129.1, 128.6, 128.1 (4 × ArCH), 124.1 (C=CH), 113.8 (ArCH), 55.3 (OCH_3), 37.3 ($ArC(O)CH_2$), 34.0 ($ArC(O)CH_2CH_2$), 33.3 ($C{=}CHCH_2Ar$), 16.4 ($CH_3C{=}CH$).

FTIR: 1684, 1510, 1244 cm^{-1}.

MS: (ESI^+) Found $[M+H]^+$: 295.1680, $C_{20}H_{23}O$ requires 295.1693.

(4*E*)-6-(4-Methoxyphenyl)-4-methyl-1-phenylhex-4-en-1-one *O*-perfluorobenzoyl oxime 2e

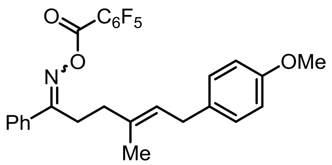

General Procedure D: Part A: (*E*)-6-(4-Methoxyphenyl)-4-methyl-1-phenylhex-4-en-1-one (0.60 g, 2.04 mmol) was used. The reaction was heated at 75 °C for 2 hours to afford the corresponding oxime (0.56 g, 89%) as a colorless solid. Part B: The corresponding oxime (0.56 g, 1.81 mmol) was employed and the reaction was stirred for 1 hour. FCC (× 2; hexane: EtOAc 30:1; toluene:hexane 2:1) afforded oxime ester **X** (0.70 g, 77%) as a colorless oil.

^{1}H NMR (400 MHz, $CDCl_3$): 7.71 (d, J = 7.5 Hz, 2H, ArH), 7.52-7.39 (m, 3H, ArH), 7.05 (d, J = 8.0 Hz, 2H, ArH), 6.82 (d, J = 8.0 Hz, 2H, ArH), 5.32 (t, J = 7.5 Hz, 1H, C=CH), 3.80 (s, 3H, OCH_3), 3.26

(d, J = 7.5 Hz, 2H, C=CHCH_2Ar), 3.04 (t, J = 7.5 Hz, 2H, Ar(CN)CH_2), 2.29 (t, J = 7.5 Hz, 2H, Ar(CN)CH_2CH_2), 1.71 (s, 3H, CH_3C=CH).

^{13}C NMR (100 MHz, $CDCl_3$): 168.6 (C=N), 157.8 (C=O), 133.6 (C=CH), 133.1 (2 signals) (2 × ArC), 131.0, 129.1, 128.8, 127.5 (4 × ArCH), 125.3 (C=CH), 133.8 (ArCH), 55.2 (OCH_3), 36.3 (Ar(CN)$CH_2$$CH_2$), 33.3 (C=CH$CH_2$Ar), 27.8 (Ar(CN)$CH_2$), 15.9 ($CH_3$C=CH). *Signals corresponding to the perfluorinated benzoyl ester moiety were not resolvable due to their anticipated weak intensity.*

^{19}F NMR (376 MHz, $CDCl_3$): *Signals for the major isomer:* -137.0 (2F), -147.5 (1F), -159.7 (2F).

FTIR: 1764, 1507, 1326, 1193 cm^{-1}.

MS: (ESI^+) Found $[M+Na]^+$: 526.1412, $C_{27}H_{22}NO_2F_5Na$ requires 526.1412.

(*R*,*E*)-2-(4-Methoxystyryl)-2-methyl-5-phenyl-3,4-dihydro-2*H*-pyrrole 3e

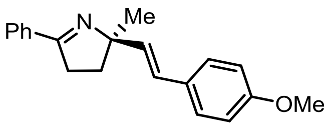

General Procedure E: Oxime ester **2e** (55.0 mg, 0.109 mmol) was employed and the reaction was heated at 120 °C for 1.5 hours. FCC (toluene:EtOAc 60:1 to 30:1) afforded the title compound (27.5 mg, 86%, 95:5 e.r.) as a pale yellow oil.

$[\alpha]_D^{23}$ +46.1 (c = 0.93, $CHCl_3$).

^{1}H NMR (400 MHz, $CDCl_3$): 7.95-7.87 (m, 2H, ArH), 7.48-7.39 (m, 3H, ArH), 7.32 (d, J = 8.0 Hz, 2H, ArH), 6.84 (d, J = 8.0 Hz, 2H, ArH), 6.44 (d, J = 16.0 Hz, 1H, CH=CHAr), 6.33 (d, J = 16.0 Hz, 1H, CH=CHAr), 3.80 (s, 3H, OCH_3), 3.15-2.90 (m, 2H, Ar(CN)CH_2), 2.23-2.11 (m, 1H, Ar(CN)CH_2CH_2), 2.08-1.92 (m, 1H, Ar(CN)CH_2CH_2), 1.53 (s, 3H, (NC)CH_3).

^{13}C NMR (100 MHz, $CDCl_3$): 171.3 (C=N), 158.9, 134.7 (2 × ArC), 134.3 (CH=CHAr), 130.4 (ArCH), 130.2 (ArC), 128.4, 127.8, 127.5 (3 × ArCH), 126.0 (CH=CHAr), 113.9 (ArC), 76.4 (quaternary C), 55.3 (OCH_3), 36.0 (Ar(CN)$CH_2$$CH_2$), 35.0 (Ar(CN)$CH_2$), 27.6 ((NC)$CH_3$).

FTIR: 2926, 1608, 1511, 1248 cm^{-1}.

MS: (ESI^+) Found $[M+H]^+$: 292.1688, $C_{20}H_{22}NO$ requires 292.1696.

The enantiomeric purity of this compound was determined by chiral HPLC (Chiralpak IB, isocratic hexane-*i*-PrOH (90:10, 0.5 mL/min, 20 °C) against a racemic standard prepared under similar conditions using $P(3,5-(CF_3)_2C_6H_3)_3$ (15 mol%) as ligand; t_R (minor) = 9.9 min and t_R (major) = 15.2 min.

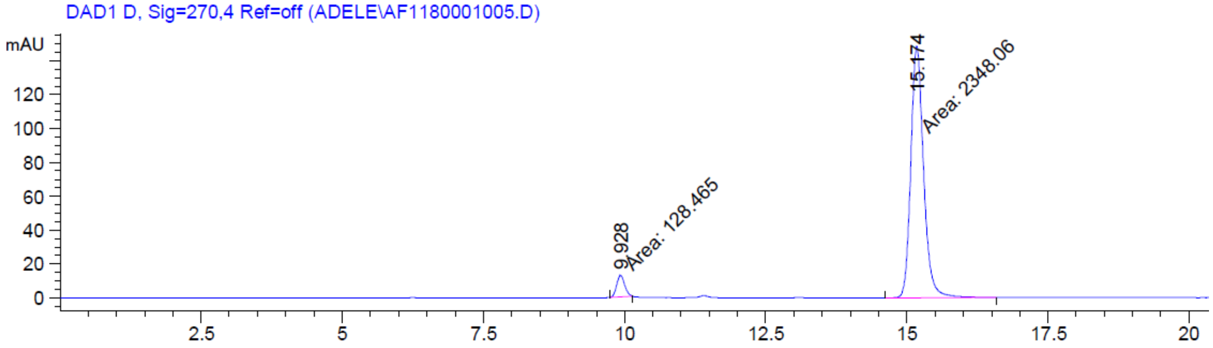

Peak #	RetTime [min]	Type	Width [min]	Area [mAU*s]	Height [mAU]	Area %
1	9.928	MM	0.1662	128.46539	12.87989	5.1873
2	15.174	MM	0.2630	2348.06177	148.77753	94.8127

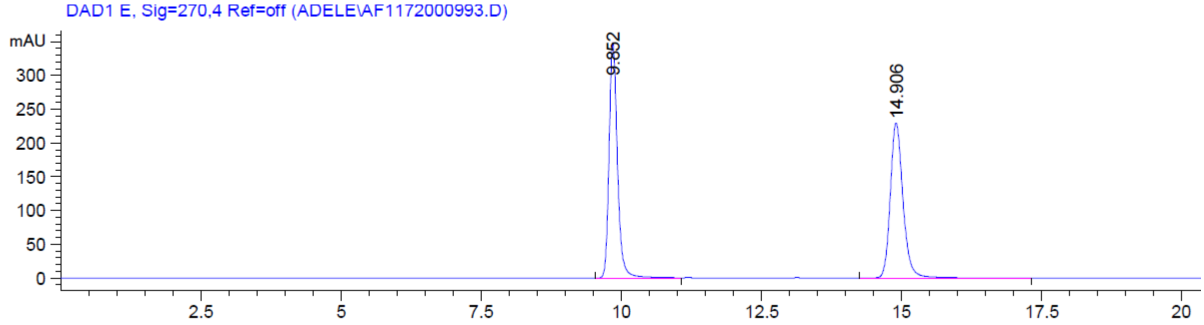

Peak #	RetTime [min]	Type	Width [min]	Area [mAU*s]	Height [mAU]	Area %
1	9.852	BB	0.1569	3616.51147	349.02615	49.8904
2	14.906	BB	0.2406	3632.39648	229.58746	50.1096

Methyl 2-(Naphthalen-2-yl)acetate

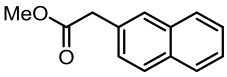

To a solution of 2-(naphthalene-2-yl)acetic acid (3.00 g, 16.10 mmol) in anhydrous MeOH (20 mL) was added concentrated H_2SO_4 (1 mL). The reaction was heated to 75 °C for 14 hours. The reaction mixture was concentrated *in vacuo*. FCC (hexane:EtOAc 5:1) afforded the title compound (2.87 g, 89%) as a colorless oil.

^{1}H NMR (400 MHz, $CDCl_3$): 7.87-7.78 (m, 3H, ArH), 7.72 (s, 1H, ArH), 7.52-7.40 (m, 3H, ArH), 3.81 (s, 2H, CH_2), 3.72 (s, 3H, CH_3).

^{13}C NMR (100 MHz, $CDCl_3$): 172.0 (CO), 133.4, 132.5, 131.4 (3 × ArC), 128.2, 128.0, 127.7, 127.6, 127.3, 126.2, 125.8 (7 × ArCH), 52.1 (CH_3), 41.4 (CH_2).

The spectroscopic properties of this compound were consistent with the data in the literature.[15]

2-(Naphthalen-2-yl)acetaldehyde

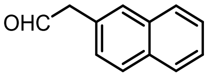

To a solution of methyl 2-(naphthalen-2-yl)acetate (2.70 g, 13.50 mmol) in anhydrous toluene (35 mL), at -78 °C was added dropwise diisobutylaluminium hydride (1.0 M solution in toluene, 14.90 mL, 14.90 mmol). The reaction was stirred for 1 hour. The reaction was quenched with MeOH (5 mL) and then poured into CH_2Cl_2 (100 mL), then washed sequentially with 1M HCl (15 mL), brine (15 mL). The organic phase was concentrated *in vacuo*. FCC (hexane:EtOAc 15:1) afforded the title compound (0.92 g, 40%) as a pale yellow oil.

^{1}H NMR (400 MHz, $CDCl_3$): 9.84 (t, *J* = 2.5 Hz, 1H, (CO)H), 7.90-7.80 (m, 3H, ArH), 7.71 (s, 1H, ArH), 7.55-7.46 (m, 2H, ArH), 7.34 (dd, *J* = 8.0 and 1.5 Hz, 1H, ArH), 3.87 (d, *J* = 2.5 Hz, 2H, CH_2).

^{13}C NMR (100 MHz, $CDCl_3$): 199.3 (C=O), 133.5, 132.4, 129.1 (3 × ArC), 128.7, 128.5, 127.7, 127.6, 127.4, 126.4, 126.1 (7 × ArCH), 50.7 (CH_2).

The spectroscopic properties of this compound were consistent with the data in the literature.[16]

Ethyl (*E*)-2-methyl-4-(naphthalene-2-yl)but-2-enoate

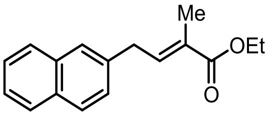

To a solution of 2-(naphthalen-2-yl)acetaldehyde (0.82 g, 4.80 mmol) in CH_2Cl_2 (20 mL) at 0 °C was added a solution of ethyl 2-(triphenylphosphoranylidene)butanoate (2.09 g, 5.76 mmol) in CH_2Cl_2 (20 mL). The reaction was stirred at room temperature for 16 hours. The reaction was cooled to room temperature and concentrated *in vacuo*. The oil was taken up in hexane (80 mL) and the precipitate filtered off. The filtrate was concentrated *in vacuo*. FCC (hexane:EtOAc 70:1) afforded the title compound (1.09 g, 89%) as a colorless oil.

^{1}H NMR (400 MHz, $CDCl_3$): 7.85-7.76 (m, 3H, ArH), 7.62 (s, 1H, ArH), 7.51-7.41 (m, 2H, ArH), 7.33 (d, *J* = 8.0 Hz, 1H, ArH), 7.01 (tq, *J* = 7.5 and 1.5 Hz, 1H, HC=C), 4.21 (q, *J* = 7.0 Hz, 2H, (CO)OCH_2CH_3), 3.70 (d, *J* = 7.5 Hz, 2H, ArCH_2CH), 2.02 (s, 3H, HC=CCH_3), 1.30 (t, *J* = 7.0 Hz, 3H, (CO)OCH_2CH_3).

^{13}C NMR (100 MHz, $CDCl_3$): 168.1 (C=O), 137.8 (HC=C), 136.5, 133.6, 132.2 (3 × ArC), 128.7 (HC=C), 128.3, 127.6, 127.5, 127.1, 126.6, 126.1, 125.5 (7 × ArCH), 60.6 ((CO)OCH_2CH_3), 35.0 (ArCH_2CH), 14.3 ((CO)OCH_2CH_3), 12.6 (HC=CCH_3).

FTIR: 1706, 1257 cm^{-1}.

MS: (ESI^+) Found $[M+H]^+$: 255.1379, $C_{17}H_{19}O_2$ requires 255.1380.

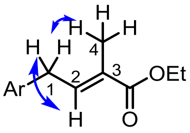

To confirm the olefin geometry, nOe analysis was carried out. An nOe enhancement between C1-H_2 and C2-H was observed and nOe was observed between C1-H_2 and C4-H_2, which is consistent with an (E)-olefin geometry.

(*E*)-2-Methyl-4-(naphthalen-2-yl)but-2-en-1-ol

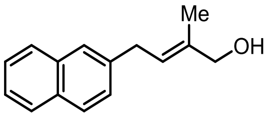

General Procedure A: In a modification to the General Procedure, $LiAlH_4$ (1M in Et_2O) was employed with ethyl (*E*)-2-methyl-4-(naphthalene-2-yl)but-2-enoate (1.19 g, 4.69 mmol). FCC (hexane:EtOAc 5:1) afforded the title compound (0.95 g, 96%) as a pale yellow oil.

^{1}H NMR (400 MHz, $CDCl_3$): 7.84-7.75 (m, 3H, ArH), 7.62 (s, 1H, ArH), 7.49-7.40 (m, 2H, ArH), 7.33 (dd, *J* = 8.5 and 1.5 Hz, 1H, ArH), 5.75-5.68 (m, 1H, HC=C), 4.10 (s, 2H, CH_2OH), 3.58 (d, *J* = 7.5 Hz, 2H, $ArCH_2CH$), 1.85 (s, 3H, $HC=CCH_3$).

^{13}C NMR (100 MHz, $CDCl_3$): 138.4 (ArC), 136.0 (HC=C), 133.6, 132.0 (2 × ArC), 128.0, 127.6, 127.4, 127.2, 126.2, 126.0, 125.2 (7 × ArCH), 124.4 (HC=C), 68.8 (CH_2OH), 34.1 ($ArCH_2CH$), 13.9 ($HC=CCH_3$).

The spectroscopic properties of this compound were consistent with the data in the literature.[17]

(*E*)-2-(4-Bromo-3-methylbut-2-en-1-yl)naphthalene

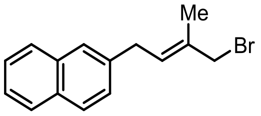

General Procedure B: (*E*)-2-Methyl-4-(naphthalen-2-yl)but-2-en-1-ol (0.90 g, 4.24 mmol) was employed to afford the title compound (1.05 g, 90%) as a colorless oil.

^{1}H NMR (400 MHz, $CDCl_3$): 7.84-7.75 (m, 3H, ArH), 7.60 (s, 1H, ArH), 7.49-7.40 (m, 2H, ArH), 7.30 (dd, *J* = 8.5 and 1.5 Hz, 1H, ArH), 5.88 (t, *J* = 7.5 Hz, 1H, HC=C), 4.04 (s, 2H, CH_2Br), 3.56 (d, *J* = 7.5 Hz, 2H, $ArCH_2CH$), 1.93 (s, 3H, $HC=CCH_3$).

^{13}C NMR (100 MHz, $CDCl_3$): 132.8, 128.9 (2 × ArC), 128.5 (HC=C), 127.3 (ArC), 124.7 (HC=C), 123.4, 122.9, 122.7, 122.3, 121.6, 121.3, 120.6 (7 × ArCH), 36.5 (CH_2Br), 29.9 ($ArCH_2CH$), 10.2 ($HC=CCH_3$).

FTIR: 1507, 1206 cm^{-1}.

MS: (EI^+) Found $[M]^+$: 274.0366, $C_{15}H_{15}{}^{79}Br$ requires 274.0357.

(*E*)-4-Methyl-6-(naphthalen-2-yl)-1-phenylhex-4-en-1-one

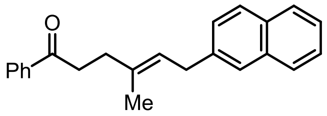

General Procedure C: Ethyl benzoylacetate (0.57 mL, 3.31 mmol) and (*E*)-2-(4-bromo-3-methylbut-2-en-1-yl)naphthalene (1.00 g, 3.65 mmol) were employed. FCC (hexane:EtOAc 100:1) afforded the title compound (0.88 g, 85%) as a colorless oil.

^{1}H NMR (400 MHz, $CDCl_3$): 8.00-7.94 (m, 2H, ArH), 7.83-7.74 (m, 3H, ArH), 7.60 (s, 1H, ArH), 7.56 (t, *J* = 7.5 Hz, 1H, ArH), 7.49-7.39 (m, 4H, ArH), 7.31 (dd, *J* = 8.5 and 1.5 Hz, 1H, ArH), 5.49 (tq, *J* = 7.5 and 1.5 Hz, 1H, C=CH), 3.54 (d, *J* = 7.5 Hz, 2H, ArCH_2CH), 3.13 (t, *J* = 7.5 Hz, 2H, Ar(CO)CH_2), 2.53 (t, *J* = 7.5 Hz, 2H, Ar(CO)CH_2CH_2), 1.84 (s, 3H, CH_3C=CH).

^{13}C NMR (100 MHz, $CDCl_3$): 200.0 (C=O), 138.9, 137.0 (2 × ArC), 135.3 (C=CH), 133.6 (ArC), 132.9 (ArCH), 132.0 (ArC), 128.6, 128.0, 127.9, 127.6, 127.4, 127.3, 126.1, 125.9, 125.1 (9 × ArCH), 123.5 (C=CH), 37.3 (Ar(CO)CH_2), 34.4 (C=CHCH_2Ar), 34.0 (Ar(CO)CH_2CH_2), 16.5 (CH_3C=CH).

FTIR: 1684, 1448, 1203 cm^{-1}.

MS: (ESI^+) Found $[M+H]^+$: 315.1746, $C_{23}H_{23}O$ requires 315.1743.

(4*E*)-4-Methyl-6-(naphthalen-2-yl)-1-phenylhex-4-en-1-one *O*-perfluorobenzoyl oxime 2f

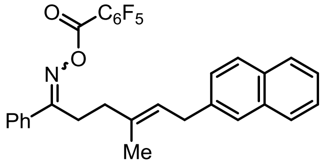

General Procedure D: Part A: (*E*)-4-Methyl-6-(naphthalen-2-yl)-1-phenylhex-4-en-1-one (0.86 g, 2.73 mmol) was used. The reaction was heated at 75 °C for 2 hours to afford the corresponding oxime (0.84 g, 94%) as a colorless solid. Part B: The corresponding oxime (0.84 g, 2.55 mmol) was employed and the reaction was stirred for 1 hour. FCC (hexane:EtOAc 40:1) afforded the title compound (1.10 g, 82%) as colorless oil.

^{1}H NMR (400 MHz, $CDCl_3$): 7.83-7.69 (m, 5H, ArH), 7.57 (s, 1H, ArH), 7.50-7.38 (m, 5H, ArH), 7.30-7.25 (m, 1H, ArH), 5.46-5.39 (m, 1H, C=CH), 3.48 (d, *J* = 7.5 Hz, 2H, ArCH_2CH), 3.07 (t, *J* = 7.5 Hz, 2H, Ar(CN)CH_2), 2.34 (t, *J* = 7.5 Hz, 2H, Ar(CN)CH_2CH_2), 1.77 (s, 3H, CH_3C=CH).

^{13}C NMR (100 MHz, $CDCl_3$): 168.6 (C=O), 138.6 (ArC), 134.2 (C=CH), 133.6, 133.1, 132.0 (3 × ArC), 131.0, 128.8, 128.0, 127.6, 127.5, 127.3, 127.2, 126.1, 125.9, 125.2 (10 × ArCH), 124.8 (C=CH), 36.3 (Ar(CN)CH_2CH_2), 34.4 (C=CHCH_2Ar), 27.8 (Ar(CN)CH_2), 16.1 (CH_3C=CH). *Signals corresponding to the perfluorinated benzoyl ester moiety were not resolvable due to their anticipated weak intensity.*

^{19}F NMR (376 MHz, $CDCl_3$): *Signals for the major isomer:* -136.9 (2F), -147.5 (1F), -159.7 (2F).

FTIR: 1763, 1498, 1325, 1193 cm^{-1}.

MS: (ESI^+) Found $[M+Na]^+$: 546.1465, $C_{30}H_{22}NO_2F_5Na$ requires 546.1463.

(*R*,*E*)-2-Methyl-2-(2-(naphthalen-2-yl)vinyl)-5-phenyl-3,4-dihydro-2*H*-pyrrole 3f

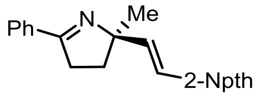

General Procedure E: Oxime ester **2f** (55.0 mg, 0.105 mmol) was employed and the reaction was heated at 100 °C for 3.75 hours. FCC (toluene:hexane 3:1 to toluene) afforded the title compound (23.0 mg, 71%, 93:7 e.r.) as a pale yellow oil.

$[\alpha]_D^{22}$ +29.4 (c = 1.02, $CHCl_3$).

^{1}H NMR (400 MHz, $CDCl_3$): 7.98-7.92 (m, 2H, ArH), 7.82-7.72 (m, 4H, ArH), 7.63 (dd, *J* = 8.5 and 1.5 Hz, 1H, ArH), 7.50-7.39 (m, 5H, ArH), 6.68 (d, *J* = 16.0 Hz, 1H, (NC)CH=CHAr), 6.60 (d, *J* = 16.0 Hz, 1H, (NC)CH=CHAr), 3.18-3.01 (m, 2H, Ar(CN)CH_2), 2.29-2.18 (m, 1H, Ar(CN)CH_2CH_2), 2.09-1.99 (m, 1H, Ar(CN)CH_2CH_2), 1.60 (s, 3H, (NC)CH_3).

^{13}C NMR (100 MHz, $CDCl_3$): 171.5 (C=N), 136.9 ((NC)CH=CHAr), 134.9, 134.7, 133.7, 132.8 (4 × ArC), 130.5, 128.4, 128.0, 127.9, 127.8, 127.6 (6 × ArCH), 126.7 ((NC)CH=CHAr), 126.1 (2 signals), 125.6, 123.7 (4 × ArCH), 76.5 (quaternary C), 36.1 (Ar(CN)CH_2CH_2), 35.0 (Ar(CN)CH_2), 27.6 ((NC)CH_3).

FTIR: 1613, 1448, 1339, 1279 cm^{-1}.

MS: (ESI^+) Found $[M+H]^+$: 312.1742, $C_{23}H_{22}N$ requires 312.1747.

The enantiomeric purity of this compound was determined by chiral HPLC (Chiralpak IB, isocratic hexane-*i*-PrOH (90:10, 0.5 mL/min, 20 °C) against a racemic standard prepared under similar conditions using $P(3,5-(CF_3)_2C_6H_3)_3$ (15 mol%) as ligand; t_R (minor) = 9.9 min and t_R (major) = 14.7 min.

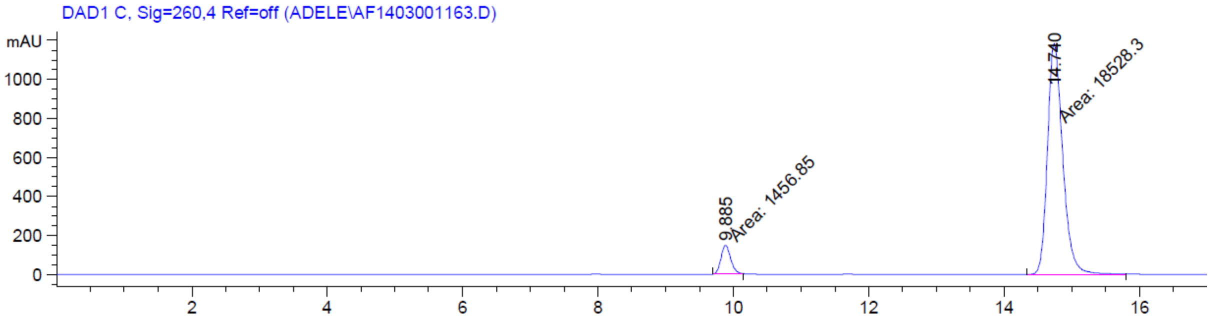

Peak #	RetTime [min]	Type	Width [min]	Area [mAU*s]	Height [mAU]	Area %
1	9.885	MM	0.1651	1456.85303	147.08362	7.2897
2	14.740	MM	0.2611	1.85283e4	1182.82361	92.7103

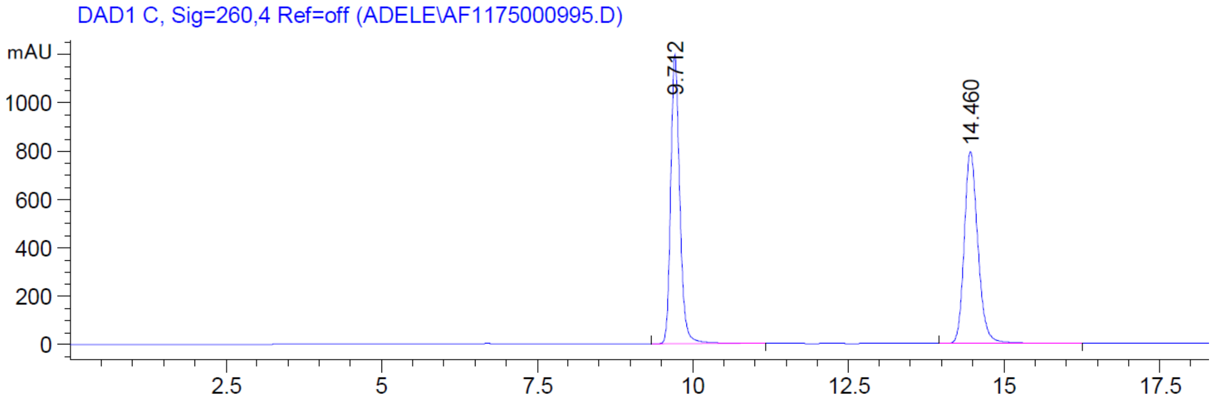

Peak #	RetTime [min]	Type	Width [min]	Area [mAU*s]	Height [mAU]	Area %
1	9.712	BB	0.1563	1.21183e4	1195.28064	49.9258
2	14.460	BB	0.2348	1.21543e4	793.14734	50.0742

Methyl (*E*)-2-(2-naphthoyl)-4,7-dimethyloct-4-enoate

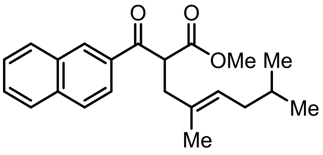

General Procedure C: Part A: Methyl 3-(naphthalene-2-yl)-3-oxopropanoate (0.98 g, 4.30 mmol) and (*E*)-1-bromo-2,5-dimethylhex-2-ene[12] (0.90 g, 4.74 mmol) were employed. FCC (hexane:EtOAc 30:1) afforded the title compound (1.40 g, 92%) as a pale yellow oil.

H NMR (400 MHz, $CDCl_3$): 8.54 (s, 1H, ArH), 8.08-7.96 (m, 2H, ArH), 7.90 (t, *J* = 8.0 Hz, 2H, ArH), 7.66-7.54 (m, 2H, ArH), 5.24 (t, *J* = 7.0 Hz, 1H, C=CH), 4.72 (t, *J* = 7.0 Hz, 1H, (CO)CH(CO)), 3.68 (s, 3H, (CO)OCH_3), 2.88-2.69 (m, 2H, (Ar(CO)$CHCH_2$), 1.83 (dd, *J* = 7.0 and 7.0 Hz, 2H, C=$CHCH_2$), 1.66 (s, 3H, CH_3C=CH), 1.57-1.46 (m, 1H, $CH(CH_3)_2$), 0.81 (t, *J* = 6.5 Hz, 6H, $CH(CH_3)_2$).

^{13}C NMR (100 MHz, $CDCl_3$): 194.8 (Ar(C=O), 170.2 ((CO)OCH_3), 135.7, 133.7, 132.5 (3 × ArC), 131.5 (C=CH), 130.5, 129.7, 128.8, 128.6, 127.8 (5 × ArCH), 126.9, 126.8 (ArCH and (C=CH), 124.1 (ArCH), 53.0 (Ar(CO)CH), 52.5 ((CO)OCH_3), 39.0 (Ar(CO)$CHCH_2$), 37.1 (C=$CHCH_2$), 28.7 ($CH(CH_3)_2$), 22.3, 22.2 ($CH(CH_3)_2$), 16.3 (CH_3C=CH).

FTIR: 1739, 1681, 1434, 1278, 1153 cm^{-1}.

MS: (ESI^+) Found $[M+H]^+$: 339.1943, $C_{22}H_{27}O_3$ requires 339.1955.

(*E*)-4,7-Dimethyl-1-(naphthalene-2-yl)oct-4-en-1-one

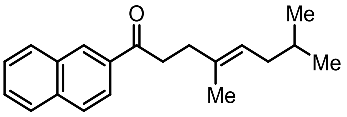

General Procedure C: Part B: Methyl (E)-2-(2-naphthoyl)-4,7-dimethyloct-4-enoate (1.40 g, 4.14 mmol) was employed. FCC (hexane:EtOAc 20:1) afforded the title compound (0.78 g, 67%) as a colorless oil.

^{1}H NMR (400 MHz, CDCl$_3$): 8.49 (s, 1H, ArH), 8.05 (dd, *J* = 8.0 and 1.5 Hz, 1H, ArH), 7.98 (d, *J* = 8.0 Hz, 1H, ArH), 7.93-7.87 (m, 2H, ArH), 7.64-7.52 (m, 2H, ArH), 5.29-5.22 (m, 1H, C=CH), 3.25-3.18 (m, 2H, Ar(CO)CH$_2$), 2.50 (t, *J* = 7.0 Hz, 2H, Ar(CO)CH$_2$CH$_2$), 1.90 (dd, *J* = 7.0 and 7.0 Hz, 2H, C=CHCH$_2$), 1.70 (s, 3H, CH$_3$C=CH), 1.65-1.55 (m, 1H, CH(CH$_3$)$_2$), 0.89 (d, *J* = 6.5 Hz, 6H, CH(CH$_3$)$_2$).

^{13}C NMR (100 MHz, CDCl$_3$): 200.2 (C=O), 135.5 (ArC), 134.4, 134.2 (ArC and C=CH), 132.5 (ArCH), 129.6, 129.5, 128.4, 128.3, 127.8, 126.7, (6 × ArCH), 124.4 (C=CH), 124.0 (ArCH), 37.6 (Ar(CO)CH$_2$), 37.1 (C=CHCH$_2$), 34.4 (Ar(CO)CH$_2$CH$_2$), 28.8 (CH(CH$_3$)$_2$), 22.4 (CH(CH$_3$)$_2$), 16.3 (CH$_3$C=CH).

FTIR: 1679, 1466, 1364, 1181, 1123 cm^{-1}.

MS: (ESI$^+$) Found [M+H]$^+$: 281.1893, $C_{20}H_{25}O$ requires 281.1900.

(*E*)-4,7-Dimethyl-1-(naphthalene-2-yl)oct-4-en-1-one *O*-perfluorobenzoyl oxime 2g

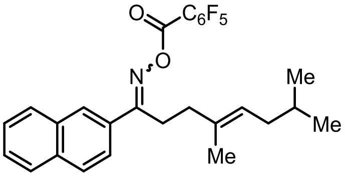

General Procedure D: Part A: (*E*)-4,7-Dimethyl-1-(naphthalene-2-yl)oct-4-en-1-one (0.77 g, 2.75 mmol) was used. The reaction was heated at 75 °C for 3 hours to afford the corresponding oxime (0.78 g, 96%) as a colorless oil. Part B: The corresponding oxime (0.78 g, 2.64 mmol) was employed and the reaction was stirred for 16 hours. FCC (hexane:EtOAc 60:1) afforded oxime ester (1.02 g, 79%, 1:0.1 mixture of oxime isomers) as a yellow solid.

^{1}H NMR (400 MHz, CDCl$_3$): *Signals for the major isomer:* 8.19 (m, 1H, ArH), 7.95-7.86 (m, 4H, ArH), 7.61-7.52 (m, 2H, ArH), 5.19 (t, *J* = 7.0 Hz, 1H, C=CH), 3.17-3.09 (m, 2H, Ar(CN)CH$_2$), 2.33 (t, *J* = 7.5 Hz, 2H, Ar(CN)CH$_2$CH$_2$), 1.85 (dd, *J* = 7.0 and 7.0 Hz, 2H, C=CHCH$_2$), 1.63 (s, 3H, CH$_3$C=CH), 1.61-1.51 (m, 1H, CH(CH$_3$)$_2$), 0.87 (d, *J* = 6.5 Hz, 6H, CH(CH$_3$)$_2$). *Characteristic signals only for the minor isomer:* 3.24-3.19 (m, 0.2H, Ar(CN)CH$_2$).

^{13}C NMR (100 MHz, CDCl$_3$): *Signals for the major isomer only:* 168.5 (C=N), 134.4 (ArC), 133.4 (C=CH), 132.9, 130.5 (2 × ArC), 128.8, 128.6, 128.0, 127.7, 127.6, 126.7 (6 × ArCH), 125.4 (C=CH), 124.0 (ArCH), 37.1 (C=CHCH$_2$), 36.7 (Ar(CN)CH$_2$CH$_2$), 28.7 (CH(CH$_3$)$_2$), 27.9 (Ar(CN)CH$_2$), 22.3

(CH(CH$_3$)$_2$), 15.9 (CH$_3$C=CH). *Signals corresponding to the perfluorinated benzoyl ester moiety were not resolvable due to their anticipated weak intensity.*

^{19}F NMR (376 MHz, CDCl$_3$): *Signals for the major isomer:* -136.9 (2F), -147.5 (1F), -159.7 (2F). *Signals for the minor isomer*: -137.0 (0.2F), -147.7 (0.1F), -160.0 (0.2F).

FTIR: 1759, 1525, 1494, 1324, 1191 cm^{-1}.

MS: (ESI$^+$) Found [M+Na]$^+$: 512.1612, $C_{27}H_{24}NO_2F_5Na$ requires 512.1619.

(*R*,*E*)-2-Methyl-2-(3-methylbut-1-en-1-yl)-5-(naphthalen-2-yl)-3,4-dihydro-2*H*-pyrrole 3g

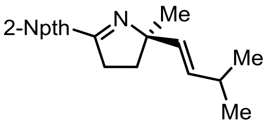

General Procedure E: Oxime ester **2g** (50.0 mg, 0.102 mmol) was employed and the reaction was heated at 100 °C for 2.5 hours. FCC (hexane:EtOAc 50:1) afforded the title compound (22.0 mg, 78%, 92:8 e.r.) as a pale yellow oil.

$[\alpha]_D^{23}$ +99.0 (c = 0.67, CHCl$_3$).

^{1}H NMR (400 MHz, CDCl$_3$): 8.21 (s, 1H, ArH), 8.12 (dd, *J* = 8.5 and 1.5 Hz, 1H, ArH), 7.94-7.82 (m, 3H, ArH), 7.56-7.48 (m, 2H, ArH), 5.66 (d, *J* = 15.5 Hz, 1H, HC=CHCH), 5.54 (dd, *J* = 15.5 and 6.5 Hz, 1H, HC=CHCH), 3.21-3.02 (m, 2H, Ar(CN)CH$_2$), 2.35-2.24 (m, 1H, CH(CH$_3$)$_2$), 2.16-2.05 (m, 1H, Ar(CN)CH$_2$CH$_2$), 1.98-1.88 (m, 1H, Ar(CN)CH$_2$CH$_2$), 1.46 (s, 3H, (NC)CH$_3$), 1.00 (d, *J* = 6.5 Hz, 6H, CH(CH$_3$)$_2$).

^{13}C NMR (100 MHz, CDCl$_3$): 170.7 (C=N), 134.3 (2 signals) (ArC and (HC=CHCH), 133.2 (HC=CHCH), 133.0, 132.3 (2 × ArC), 128.7, 128.0 (2 signals), 127.7, 126.9, 126.3, 124.8 (7 × ArCH), 76.2 (quaternary C), 36.1 (Ar(CN)CH$_2$CH$_2$), 34.9 (Ar(CN)CH$_2$), 30.9 (CH(CH$_3$)$_2$), 27.4 ((NC)CH$_3$), 22.6, 22.5 (CH(CH$_3$)$_2$).

FTIR: 1612, 1465, 1363, 1279, 1127 cm^{-1}.

MS: (ESI$^+$) Found [M+H]$^+$: 278.1899, $C_{20}H_{24}N$ requires 278.1903.

The enantiomeric purity of this compound was determined by chiral HPLC (Chiralpak IB, isocratic hexane-*i*-PrOH (95:5, 0.5 mL/min, 20 °C) against a racemic standard prepared under similar conditions using P(3,5-(CF$_3$)$_2$C$_6$H$_3$)$_3$ (15 mol%) as ligand; t_R (minor) = 8.4 min and t_R (major) = 9.4 min.

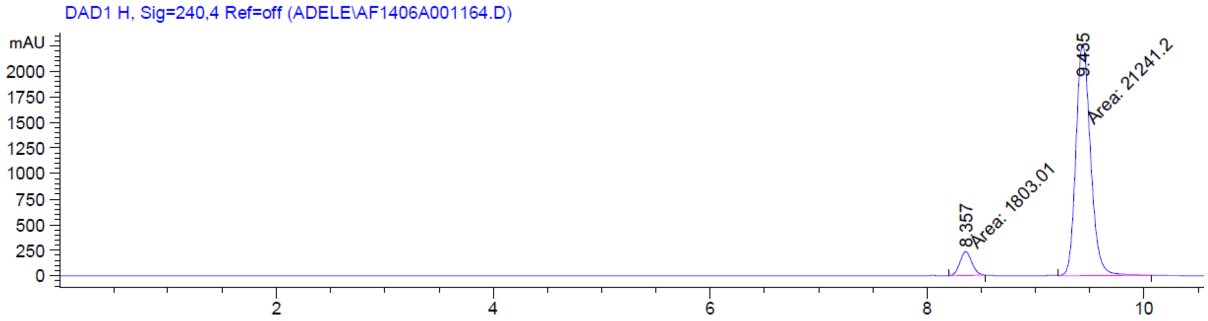

Peak #	RetTime [min]	Type	Width [min]	Area [mAU*s]	Height [mAU]	Area %
1	8.357	MM	0.1295	1803.01392	232.12125	7.8242
2	9.435	MM	0.1564	2.12412e4	2264.18335	92.1758

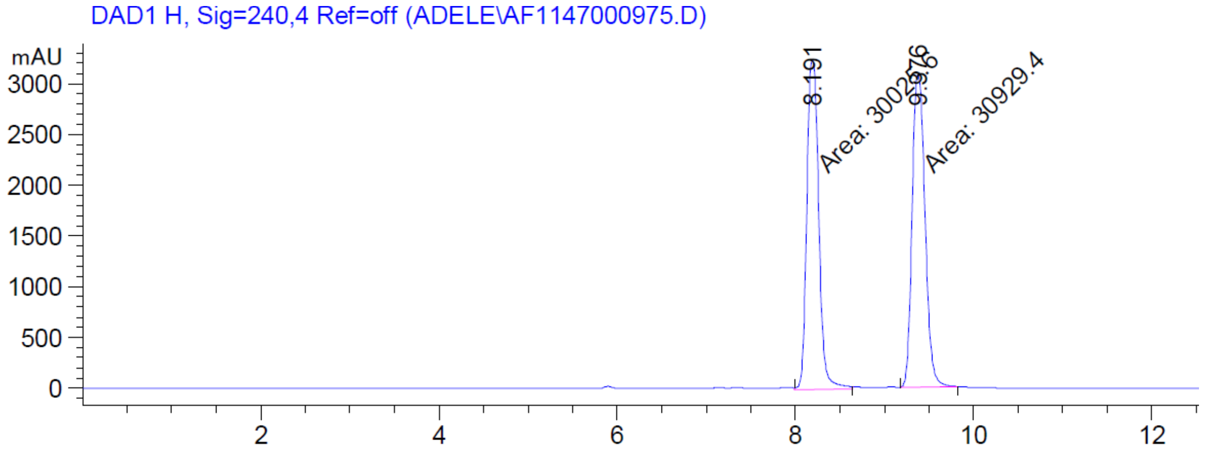

Peak #	RetTime [min]	Type	Width [min]	Area [mAU*s]	Height [mAU]	Area %
1	8.191	MM	0.1542	3.00256e4	3245.61353	49.2586
2	9.376	MM	0.1672	3.09294e4	3082.37939	50.7414

(*E*)-2-Ethylbut-2-en-1-ol

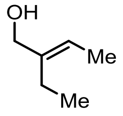

General Procedure A: (*E*)-2-Ethylbut-2-enal (5.80 mL, 51.02 mmol) was employed. The reaction was stirred at 0 °C for 30 minutes. Careful concentration *in vacuo* was performed at 0 °C to remove Et_2O. Kugelrohr distillation was performed to remove MeOH to afford the corresponding alcohol (4.04 g, 79%, 1:0.05 mixture of *E*:*Z* isomers) as a pale yellow oil.

^{1}H NMR (400 MHz, $CDCl_3$): *Data for the major isomer only:* 5.46 (q, *J* = 7.0 Hz, 1H, C=CH), 4.03 (s, 2H, CH_2OH), 2.12 (q, *J* = 7.5 Hz, 2H, CH_2CH_3), 1.63 (d, *J* = 7.0 Hz, 3H, C=CHCH_3), 0.99 (t, *J* = 7.5 Hz, 3H, CH_2CH_3).

^{13}C NMR (100 MHz, $CDCl_3$): *Data for the major isomer only:* 141.4 (C=CH), 120.5 (C=CH), 66.9 (CH_2OH), 20.7 (CH_2CH_3), 12.8, 12.7 (CH_2CH_3 and C=CHCH_3),

The spectroscopic properties of this compound were consistent with the data in the literature.[18]

(*E*)-3-(Bromomethyl)pent-2-ene

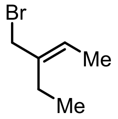

To a solution of *N*-bromosuccinimide (3.66 g, 20.56 mmol) in anhydrous CH_2Cl_2 (50 mL) at 0 °C was added dropwise dimethylsulfide (1.75 mL, 23.86 mmol). The reaction was stirred for 30 minutes and then the reaction was cooled to - 20 °C. (*E*)-2-Ethylbut-2-en-1-ol (1.35 g, 15.70 mmol) was added dropwise and the reaction was stirred for 10 minutes at - 20 °C. The reaction was warmed to room temperature and stirred for 1 hour. The reaction was poured into ice (25 g), the organic extract was washed with brine (2 × 30 mL) and the organic phase was carefully concentrated *in vacuo*. FCC (pentane) afforded the title compound (0.67 g, 26%, 1:0.05 mixture of *E*:*Z* isomers) as a colorless oil.

^{1}H NMR (400 MHz, $CDCl_3$): *Data for the major isomer only:* 5.68 (q, *J* = 7.0 Hz, 1H, C=C<u>H</u>), 4.03 (s, 2H, C<u>H₂</u>Br), 2.24 (q, *J* = 7.5 Hz, 2H, C<u>H₂</u>CH₃), 1.65 (d, *J* = 7.0 Hz, 3H, C=CHC<u>H₃</u>), 1.02 (t, *J* = 7.5 Hz, 3H, CH₂C<u>H₃</u>). *Characteristic signals for the minor isomer:* 5.50 (q, *J* = 7.0 Hz, 0.05H, C=C<u>H</u>).

^{13}C NMR (100 MHz, $CDCl_3$): *Data for the major isomer only:* 138.4 (<u>C</u>=CH), 125.8 (C=<u>C</u>H), 39.4 (<u>C</u>H₂Br), 21.0 (<u>C</u>H₂CH₃), 13.5 (C=CH<u>C</u>H₃), 12.7 (CH₂<u>C</u>H₃).

FTIR: 2927, 1660, 1609, 1249 cm^{-1}.

MS: *Mass spectrometry data was not obtained using either EI, CI and ESI techniques due to high volatility of this bromide.*

(*E*)-4-Ethyl-1-(naphthalen-2-yl)hex-4-en-1-one

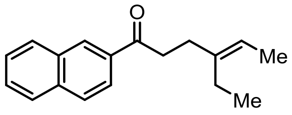

General Procedure C: Methyl 3-(naphthalen-2-yl)-3-oxopropanoate (0.83 g, 3.65 mmol) and (*E*)-3-(bromomethyl)pent-2-ene (0.65 g, 4.01 mmol) were employed. FCC (hexane:EtOAc 50:1) afforded the title compound (0.50 g, 54%, 1:0.05 mixture of *E*:*Z* isomers) as a pale yellow oil.

^{1}H NMR (400 MHz, $CDCl_3$): *Data for the major isomer:* 8.47 (s, 1H, Ar<u>H</u>), 8.03 (dd, *J* = 8.0 and 1.5 Hz, 1H, Ar<u>H</u>), 7.95 (d, *J* = 8.0 Hz, 1H, Ar<u>H</u>), 7.90-7.88 (m, 2H, Ar<u>H</u>), 7.62-7.51 (m, 2H, Ar<u>H</u>), 5.26 (q, *J* = 7.0 Hz, 1H, C=C<u>H</u>), 3.21-3.15 (m, 2H, Ar(CO)C<u>H₂</u>), 2.51-2.44 (m, 2H, Ar(CO)CH₂C<u>H₂</u>), 2.13 (q, *J* = 7.5 Hz, 2H, C<u>H₂</u>CH₃), 1.62 (d, *J* = 7.0 Hz, 3H, C=CHC<u>H₃</u>), 1.03 (t, *J* = 7.5 Hz, 3H, CH₂C<u>H₃</u>).

^{13}C NMR (100 MHz, $CDCl_3$): 200.1 (<u>C</u>=O), 140.6 (<u>C</u>=CH), 135.5, 134.4, 132.5 (3 × Ar<u>C</u>), 129.6, 129.5, 128.4, 128.3, 127.7, 126.7, 123.9 (7 × Ar<u>C</u>H), 118.4 (C=<u>C</u>H), 37.6 (Ar(CO)<u>C</u>H₂), 31.0 (Ar(CO)CH₂<u>C</u>H₂), 23.1 (<u>C</u>H₂CH₃), 13.0 (C=CH<u>C</u>H₃), 12.8 (CH₂<u>C</u>H₃).

FTIR: 1682, 1468 cm^{-1}.

MS: (ESI$^+$) Found $[M+H]^+$: 253.1584, $C_{18}H_{21}O$ requires 253.1587.

(4*E*)-4-Methyl-1-(naphthalen-2-yl)-6-phenylhex-4-en-1-one *O*-perfluorobenzoyl oxime 2h

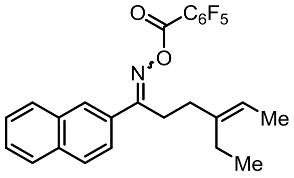

General Procedure D: Part A: (*E*)-4-Ethyl-1-(naphthalen-2-yl)hex-4-en-1-one (0.48 g, 1.90 mmol) was used. The reaction was heated at 75 °C for 2 hours to afford the corresponding oxime (0.45 g, 89%) as a colorless solid. Part B: The corresponding oxime (0.45 g, 1.69 mmol) was employed and the reaction was stirred for 2 hours. FCC (hexane: EtOAc 40:1 to EtOAc) afforded the title compound (0.61 g, 79%, 1:0.05 mixture of *E*:*Z* isomers) as a colorless solid.

^{1}H NMR (400 MHz, $CDCl_3$): *Signals for the major isomer:* 8.17 (s, 1H, ArH), 7.94-7.83 (m, 4H, ArH), 7.60-7.49 (m, 2H, ArH), 5.22 (q, *J* = 7.0 Hz, 1H, C=CH), 3.07 (t, *J* = 7.0 Hz, 2H, Ar(CN)CH_2), 2.30 (t, *J* = 7.0 Hz, 2H, Ar(CN)CH_2CH_2), 2.04 (q, *J* = 7.5 Hz, 2H, CH_2CH_3), 1.57 (d, *J* = 7.0 Hz, 3H, C=CHCH_3), 0.92 (t, *J* = 7.5 Hz, 3H, CH_2CH_3).

^{13}C NMR (100 MHz, $CDCl_3$): *Signals for the major isomer only:* 168.5 (C=N), 139.8 (C=CH), 134.5, 132.9, 130.4 (3 × ArC), 128.8, 128.6, 128.0, 127.7, 127.6, 126.7, 123.9 (7 × ArCH), 119.6 (C=CH), 33.3 (Ar(CN)CH_2CH_2), 28.0 (Ar(CN)CH_2), 22.6 (CH_2CH_3), 13.0 (C=CHCH_3), 12.6 (CH_2CH_3). *Signals corresponding to the perfluorinated benzoyl ester moiety were not resolvable due to their anticipated weak intensity.*

^{19}F NMR (376 MHz, $CDCl_3$): *Signals for the major isomers:* -136.9 (2F), -147.5 (1F), -159.7 (2F), *Signals for the minor isomer:* -137.4 (0.1F), -148.1 (0.05F), -160.0 (0.1F).

FTIR: 1755, 1524, 1491, 1324, 1189 cm^{-1}.

MS: (ESI^+) Found $[M+Na]^+$: 484.1300, $C_{25}H_{20}NO_2F_5Na$ requires 484.1306.

(*R*)-2-Ethyl-5-(naphthalen-2-yl)-2-vinyl-3,4-dihydro-2*H*-pyrrole 3h

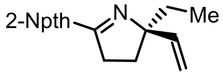

General Procedure E: Oxime ester **2h** (50.0 mg, 0.108 mmol) was employed and the reaction was heated at 80 °C for 6.5 hours. FCC (toluene:EtOAc 80:1) afforded the title compound (18.5 mg, 68%, 92:8 e.r.) as a pale yellow solid.

$[\alpha]_D^{22}$ +38.5 (c = 0.73, $CHCl_3$).

M.P. 75-77 °C (CH_2Cl_2-pentane)

^{1}H NMR (400 MHz, $CDCl_3$): 8.21 (s, 1H, ArH), 8.14 (dd, *J* = 8.0 and 1.5 Hz, 1H, ArH), 7.94-7.82 (m, 3H, ArH), 7.56-7.48 (m, 2H, ArH), 6.12 (dd, *J* = 17.0 and 11.0 Hz, 1H, HC=CH_2), 5.12 (dd, *J* = 17.0 and 1.5 Hz, 1H, HC=CH_2), 5.07 (dd, *J* = 11.0 and 1.5 Hz, 1H, HC=CH_2), 3.20-2.98 (m, 2H, Ar(CN)CH_2), 2.13-1.91 (m, 2H, Ar(CN)CH_2CH_2), 1.90-1.75 (m, 2H, (NC)CH_2CH_3), 0.98 (t, *J* = 7.5 Hz, 3H, (NC)CH_2CH_3).

^{13}C NMR (100 MHz, $CDCl_3$): 171.2 (C=N), 143.3 (CH=CH_2), 134.4, 123.0, 132.3 (3 × ArC), 128.7, 128.0 (2 signals), 127.7, 126.9, 126.3, 124.8 (7 × ArCH), 111.9 (CH=CH_2), 80.6 (quaternary C), 35.0 (Ar(CN)CH_2), 33.5 ((NC)CH_2CH_3), 31.8 (Ar(CN)CH_2CH_2), 8.8 ((NC)CH_2CH_3).

FTIR: 2965, 1613 cm^{-1}.

MS: (ESI^+) Found $[M+H]^+$: 250.1592, $C_{18}H_{20}N$ requires 250.1590.

The enantiomeric purity of this compound was determined by chiral HPLC (Chiralpak IB, isocratic hexane-*i*-PrOH (99:1, 0.5 mL/min, 20 °C) against a racemic standard prepared under similar conditions using $P(3,5-(CF_3)_2C_6H_3)_3$ (15 mol%) as ligand; t_R (major) = 12.5 min and t_R (minor) = 13.9 min. The absolute structure of this molecule was confirmed by Vibrational Circular Dichroism (VCD).

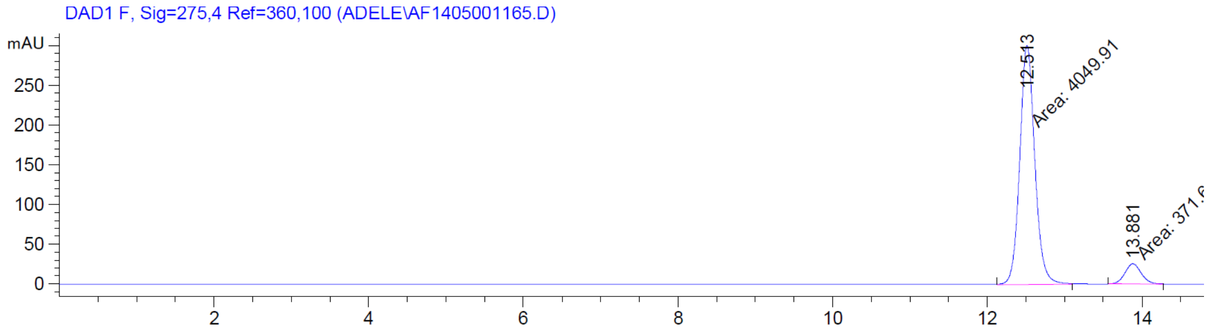

Peak #	RetTime [min]	Type	Width [min]	Area [mAU*s]	Height [mAU]	Area %
1	12.513	MM	0.2243	4049.91089	300.87048	91.5953
2	13.881	MM	0.2437	371.61670	25.41363	8.4047

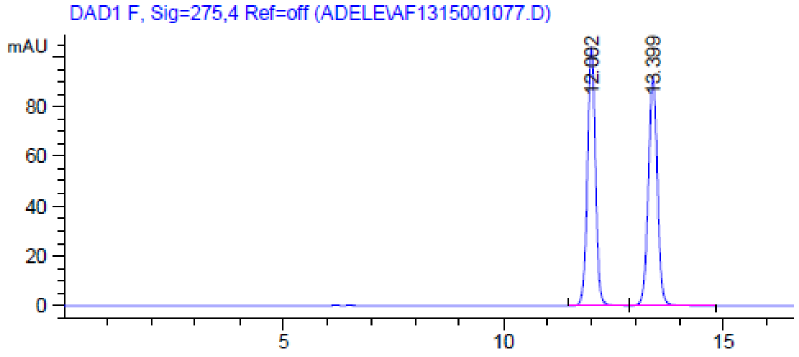

Peak #	RetTime [min]	Type	Width [min]	Area [mAU*s]	Height [mAU]	Area %
1	12.002	BB	0.1942	1315.42322	103.54400	49.9591
2	13.399	BB	0.2214	1317.57605	91.84719	50.0409

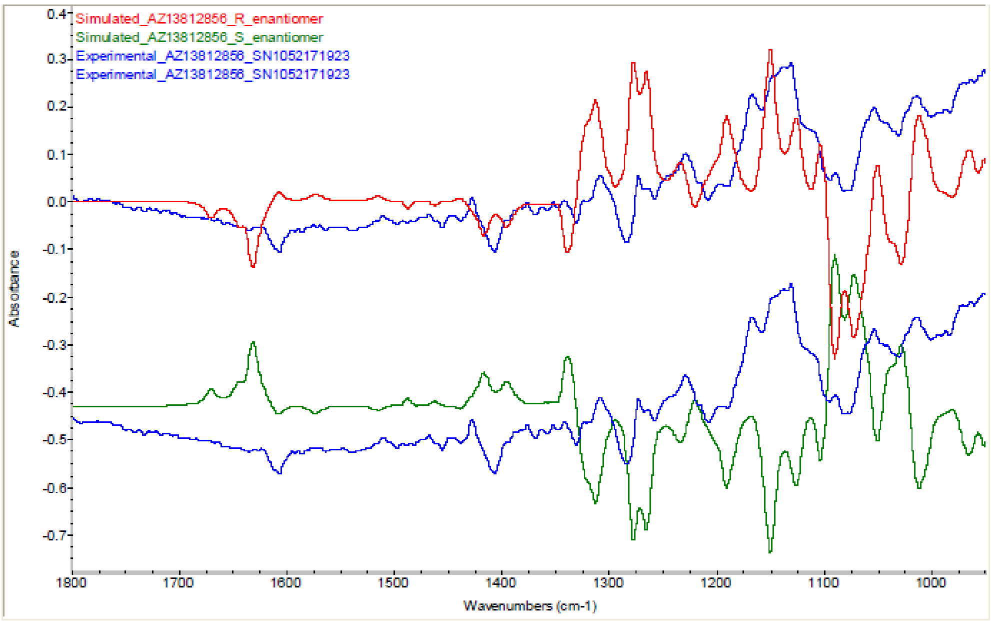

Comparison of experimental VCD spectrum of dihydropyrrole **3h** *(in duplicate, blue) and simulated spectra of the R (red) and S (green) enantiomers. The agreement between the simulated spectrum of the R enantiomer and the experimental spectrum is good, therefore dihydropyrrole* **3h** *has R configuration.*

Experimental: A 15.4 mg sample of dihydropyrrole **3h** was dissolved in $CDCl_3$ (150 μL). A VCD spectrum was acquired in 0.100 mm BaF_2 cells for 7 hours each in a BioTools ChiralIR instrument equipped with dual source and dual photoelastic modulator. The resolution was 4 cm^{-1}. The experimental VCD spectrum shows weak characteristic bands.

Computational Spectral Simulations: A Monte Carlo molecular mechanics search for low energy geometries was conducted for full structures of the two enantiomers, *R* and *S*. *MacroModel* within the *Maestro* graphical interface (Schrödinger Inc.) was used to generate starting coordinates for conformers. All conformers within 5 kcal/mole of the lowest energy conformer were used as starting points for density functional theory (DFT) minimizations within *Gaussian09*. Optimized structures, harmonic vibrational frequencies/intensities, VCD rotational strengths, and free energies at STP (including zero-point energies) were determined for each conformer. In these calculations, the functional B3LYP and the basis set 6-31G* were used. Simulations of infrared and VCD spectra for each conformation were generated using an in-house built program to fit Lorentzian line shapes (12 cm^{-1} line width) to the computed spectra thereby allowing direct comparisons between simulated and experimental spectra.

Results: The experimental spectrum was compared with simulated spectra of the two enantiomers based on DFT calculations starting with full structures. The comparison is presented above. The agreement between the simulated spectrum of the *R* enantiomer and the experimental spectrum is good. It is therefore concluded that dihydropyrrole **3h** has *R* configuration.

Methyl (*E*)-2-benzylbut-2-enoate

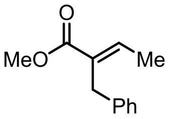

To a solution of methyl 3-hydroxy-2-methylenebutanoate (1.65 g, 12.5 mmol) and phenylboronic acid (3.10 g, 25.1 mmol) in methanol (50 mL), $[Rh(COD)Cl]_2$ (63.0 mg, 0.125 mmol) was added. The reaction was heated to 55 °C for 20 hours. The solvent was concentrated *in vacuo* and FCC (hexane:EtOAc 50:1) afforded the title compound (1.89 g, 79%) as a pale yellow oil.

^{1}H NMR (400 MHz, $CDCl_3$): 7.30-7.13 (m, 5H, ArH), 7.04 (q, *J* = 7.0 Hz, 1H, C=CH), 3.72-3.67 (m, 5H, CH_2Ar and OCH_3), 1.89 (d, *J* = 7.0 Hz, 3H, $C{=}CHCH_3$).

^{13}C NMR (100 MHz, $CDCl_3$): 168.0 (C=O), 139.6 (ArC), 138.9 (C=CH), 131.9 (C=CH), 128.3, 128.2, 125.9 (3 × ArCH), 51.7 (OCH_3), 32.0 (CH_2Ar), 14.7 ($C{=}CHCH_3$).

The spectroscopic properties of this compound were consistent with the data in the literature.[19]

(*E*)-2-Benzylbut-2-en-1-ol

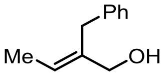

To a solution of methyl (*E*)-2-benzylbut-2-enoate (1.87 g, 9.84 mmol) in anhydrous THF (35 mL) at -78 °C, was added dropwise diisobutylaluminium hydride (1M in hexane, 20.7 mL, 20.7 mmol). The reaction was stirred for 1 hour. The reaction was quenched with sat. NH_4Cl (50 mL). The precipitate was filtered off and the organic phase was concentrated *in vacuo*. FCC (hexane:EtOAc 10:1 to 8:1) afforded the title compound (1.18 g, 74%) as a colorless oil.

^{1}H NMR (400 MHz, $CDCl_3$): 7.31-7.14 (m, 5H, ArH), 5.70 (q, *J* = 7.0 Hz, 1H, C=CH), 3.97 (m, 2H, CH_2OH), 3.49 (s, 2H, CH_2Ar), 1.77 (d, *J* = 7.0 Hz, 3H, CH_3).

^{13}C NMR (100 MHz, $CDCl_3$): 139.7 (ArC), 138.4 (HC=C), 128.5, 128.4, 126.0 (3 × ArCH), 122.5 (HC=C), 66.8 (CH_2OH), 33.4 (CH_2Ar), 13.4 (CH_3).

FTIR: 3310, 1494, 1452 cm^{-1}.

MS: (ESI^+) Found $[M+H]^+$: 145.1014, $C_{11}H_{13}$ requires 145.1012.

(*E*)-(2-(Bromomethyl)but-2-en-1-yl)benzene

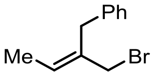

General Procedure B: (*E*)-2-Benzylbut-2-en-1-ol (0.65 g, 4.01 mmol) was employed to afford the title compound (0.75 g, 83%) as a colorless oil.

^{1}H NMR (400 MHz, $CDCl_3$): 7.32-7.14 (m, 5H, ArH), 5.86 (q, *J* = 7.0 Hz, 1H, C=CH), 3.88 (s, 2H, CH_2Br), 3.60 (s, 2H, CH_2Ar), 1.79 (d, *J* = 7.0 Hz, 3H, CH_3).

^{13}C NMR (100 MHz, $CDCl_3$): 139.0 (ArC), 135.9 (HC=C), 128.6, 128.5, 127.2 (3 × ArCH), 126.3 (HC=C), 39.1 (CH_2Br), 33.3 (CH_2Ar), 14.1 (CH_3).

FTIR: 1494, 1453, 1432, 1203 cm^{-1}.

MS: (EI$^+$) Found [M]$^+$: 224.0207, $C_{11}H_{13}{}^{79}Br$ requires 224.0201.

(*Z*)-4-Benzyl-1-(naphthalen-2-yl)hex-4-en-1-one

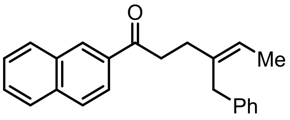

General Procedure C: Methyl 3-(naphthalen-2-yl)-3-oxopropanoate (0.60 g, 2.63 mmol) and (*E*)-(2-(bromomethyl)but-2-en-1-yl)benzene (0.65 g, 2.90 mmol) were employed. FCC (hexane:EtOAc 50:1) afforded the title compound (0.57 g, 69%) as a colorless oil.

^{1}H NMR (400 MHz, $CDCl_3$): 8.33 (s, 1H, ArH), 7.95 (dd, *J* = 8.0 and 2.0 Hz, 1H, ArH), 7.92 (d, *J* = 8.0 Hz, 1H, ArH), 7.87-7.83 (m, 2H, ArH), 7.62-7.50 (m, 2H, ArH), 7.31-7.14 (m, 5H, ArH), 5.52 (q, *J* = 7.0 Hz, 1H, C=CH), 3.49 (s, 2H, CH_2Ar), 3.15-3.08 (m, 2H, Ar(CO)CH_2), 2.48-2.40 (m, 2H, Ar(CO)CH_2CH_2), 1.75 (d, *J* = 7.0 Hz, 3H, CH_3).

^{13}C NMR (100 MHz, $CDCl_3$): 199.9 (C=O), 140.0 (ArC), 137.4 (C=CH), 135.5, 134.2, 132.5 (3 × ArC), 129.6, 129.5, 128.5, 128.4, 128.3 (2 signals), 127.7, 126.7, 126.0, 123.9 (10 × ArCH), 121.0 (C=CH), 37.6 (Ar(CO)CH_2), 36.1 (CH_2Ar), 31.5 (Ar(CO)CH_2CH_2), 13.7 (CH_3).

FTIR: 1679, 1182 cm^{-1}.

MS: (ESI$^+$) Found [M+H]$^+$: 315.1732, $C_{23}H_{23}O$ requires 315.1743.

(*Z*)-4-Benzyl-1-(Naphthalen-2-yl)hex-4-en-1-one *O*-perfluorobenzoyl oxime 2i

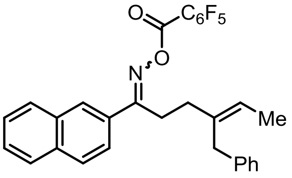

General Procedure D: Part A: (*Z*)-4-Benzyl-1-(naphthalen-2-yl)hex-4-en-1-one (0.57 g, 1.81 mmol) was used. The reaction was heated at 75 °C for 3 hours to afford the corresponding oxime (0.40 g, 67%) as a colorless solid. Part B: The corresponding oxime (0.40 g, 1.21 mmol) was employed and the reaction was stirred for 1 hour. FCC (hexane: EtOAc 40:1 to EtOAc) afforded the title compound (0.40 g, 63%, 1:0.05 mixture of oxime isomers) as a colorless solid.

^{1}H NMR (400 MHz, $CDCl_3$): *Signals for the major isomer:* 8.03 (s, 1H, ArH), 7.88-7.78 (m, 4H, ArH), 7.59-7.49 (m, 2H, ArH), 7.23-7.06 (m, 5H, ArH), 5.49 (q, *J* = 7.0 Hz, 1H, C=CH), 3.40 (s, 2H, CH_2Ar), 3.08-2.98 (m, 2H, Ar(CN)CH_2), 2.25 (t, *J* = 7.5 Hz, 2H, Ar(CN)CH_2CH_2), 1.72 (d, *J* = 7.0 Hz, 3H, CH_3).

^{13}C NMR (100 MHz, $CDCl_3$): *Signals for the major isomer only:* 168.2 (C=N), 139.5 (ArC), 136.7 (C=CH), 134.4, 132.8, 130.3 (3 × ArC), 128.8, 128.5, 128.4, 128.3, 128.0, 127.7, 127.5, 126.6, 126.0, 123.9 (10 × ArCH), 122.1 (C=CH), 35.5 (CH_2Ar), 33.5 (Ar(CN)CH_2CH_2), 27.9 (Ar(CN)CH_2), 13.6 (CH_3). *Signals corresponding to the perfluorinated benzoyl ester moiety were not resolvable due to their anticipated weak intensity.*

^{19}F NMR (376 MHz, $CDCl_3$): *Signals for the major isomers:* -136.9 (2F), -147.5 (1F), -159.6 (2F), *Signals for the minor isomer:* -137.4 (0.1F), -148.1 (0.05F), -160.0 (0.1F).

FTIR: 1756, 1526, 1492, 1323, 1189 cm^{-1}.

MS: (ESI^+) Found $[M+Na]^+$: 546.1457, $C_{30}H_{22}NO_2F_5Na$ requires 546.1463.

(*S*)-2-Benzyl-5-(naphthalen-2-yl)-2-vinyl-3,4-dihydro-2*H*-pyrrole 3i

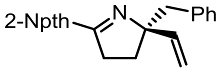

General Procedure E: Oxime ester **2i** (55.0 mg, 0.105 mmol) was employed and the reaction was heated at 80 °C for 5.5 hours. FCC (toluene:EtOAc 100:1) afforded the title compound (23 mg, 71%, 93:7 e.r.) as a pale yellow solid.

$[\alpha]_D^{23}$ -118.8 (c = 0.64, $CHCl_3$).

M.P. 89-91 °C (CH_2Cl_2-pentane).

^{1}H NMR (400 MHz, $CDCl_3$): 8.13-8.06 (m, 2H, ArH), 7.90-7.82 (m, 3H, ArH), 7.55-7.46 (m, 2H, ArH), 7.27-7.12 (m, 5H, ArH), 6.22 (dd, *J* = 17.0 and 10.0 Hz, 1H, H$C=CH_2$), 5.18 (dd, *J* = 17.0 and 1.0 Hz, 1H, HC=CH_2), 5.07 (dd, *J* = 10.0 and 1.0 Hz, 1H, HC=CH_2), 3.19 (d, *J* = 13.0 Hz, 1H, CH_2Ar), 3.01 (d, *J* = 13.0 Hz, 1H, CH_2Ar), 2.96-2.83 (m, 1H, Ar(CN)CH_2), 2.49-2.38 (m, 1H, Ar(CN)CH_2), 2.15-2.04 (m, 1H, Ar(CN)CH_2CH_2), 2.02-1.92 (m, 1H, Ar(CN)CH_2CH_2).

^{13}C NMR (100 MHz, $CDCl_3$): 172.2 (C=N), 143.8 (CH=CH_2), 137.7, 134.4, 132.9, 132.1 (4 × ArC), 130.8 (2 signals), 128.7, 128.0 (2 signals), 127.7 (2 signals), 127.0, 126.3, 126.1 (10 × ArCH), 112.1 (CH=CH_2), 80.6 (quaternary C), 46.6 (CH_2Ar), 35.1 (Ar(CN)CH_2), 31.7 (Ar(CN)CH_2CH_2).

FTIR: 1613, 1280, 1134 cm^{-1}.

MS: (ESI^+) Found $[M+H]^+$: 312.1733, $C_{23}H_{22}N$ requires 312.1747.

The enantiomeric purity of this compound was determined by chiral HPLC (Chiralpak IA, isocratic hexane-*i*-PrOH (95:5, 0.5 mL/min, 20 °C) against a racemic standard prepared under similar conditions using $P(3,5\text{-}(CF_3)_2C_6H_3)_3$ (15 mol%) as ligand; t_R (major) = 11.4 min and t_R (minor) = 13.0 min.

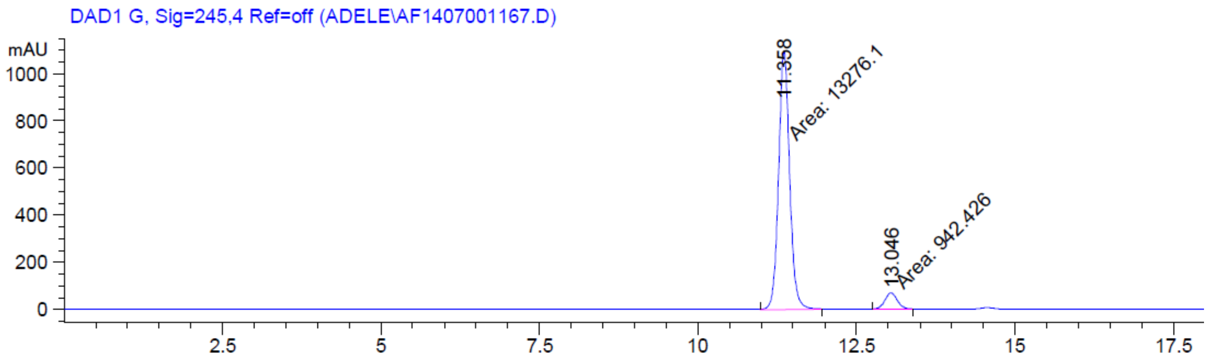

Peak #	RetTime [min]	Type	Width [min]	Area [mAU*s]	Height [mAU]	Area %
1	11.358	MM	0.2018	1.32761e4	1096.54993	93.3718
2	13.046	MM	0.2282	942.42639	68.82517	6.6282

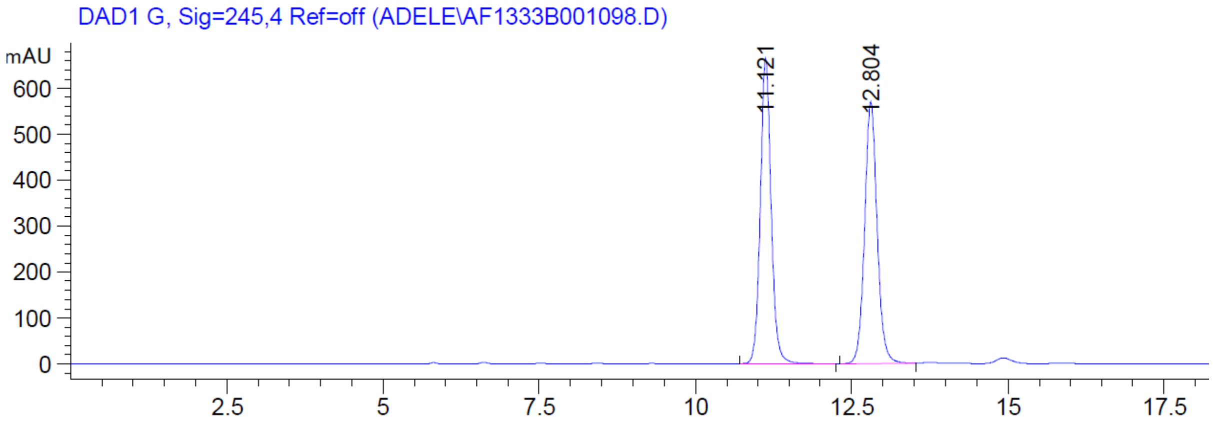

Peak #	RetTime [min]	Type	Width [min]	Area [mAU*s]	Height [mAU]	Area %
1	11.121	BB	0.1819	7970.15430	664.71588	50.0925
2	12.804	BB	0.2124	7940.73389	570.41974	49.9075

(*E*)-2-Isopropylbut-2-en-1-ol

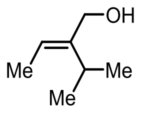

To a solution of 2-butyn-1-ol (2.13 mL, 28.5 mmol) in anhydrous Et_2O (50 mL) was added CuI (5.4 g, 28.5 mmol). The reaction was cooled to 0 °C. Isopropylmagnesium chloride (1.89 M in THF, 45.5 mL, 85.6 mmol) was added dropwise. The reaction was stirred at room temperature for 20 hours. The reaction was quenched with sat. NH_4Cl (100 mL) and extracted with Et_2O (2 × 150 mL). The solvent was carefully distilled off and purification of the residue by FCC (pentane:Et_2O 30:1 to 10:1) afforded the title compound (0.54 g, 17%) as a colorless oil.

^{1}H NMR (400 MHz, $CDCl_3$): 5.49 (q, *J* = 7.0 Hz, 1H, $\underline{H}C{=}C$), 4.09 (s, 2H, $C\underline{H_2}OH$), 2.86 (sept. *J* = 6.5 Hz, 1H, $C\underline{H}(CH_3)_2$), 1.66 (d, *J* = 7.0 Hz, 3H, $C\underline{H_3}CH{=}C$), 1.07 (d, *J* = 6.5 Hz, 6H, $CH(C\underline{H_3})_2$).
^{13}C NMR (100 MHz, $CDCl_3$): 144.7 ($HC{=}\underline{C}$), 120.5 ($H\underline{C}{=}C$), 64.6 ($\underline{C}H_2OH$), 27.6 ($\underline{C}H(CH_3)_2$), 21.2 ($CH(\underline{C}H_3)_2$), 12.7 ($\underline{C}H_3CH{=}C$).
The spectroscopic properties of this compound were consistent with the data in the literature.[20]

(*E*)-(3-(Bromomethyl)-4-methylpent-2-ene

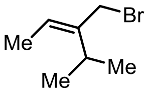

General Procedure B: (*E*)-2-Isopropylbut-2-en-1-ol (0.53 g, 4.65 mmol) was employed to afford the title compound (0.81 g, 98%) as a colorless oil.
^{1}H NMR (400 MHz, $CDCl_3$): 5.71 (q, *J* = 7.0 Hz, 1H, $\underline{H}C{=}C$), 4.02 (s, 2H, $C\underline{H_2}Br$), 2.86 (sept. *J* = 6.5 Hz, 1H, $C\underline{H}(CH_3)_2$), 1.69 (d, *J* = 7.0 Hz, 3H, $C\underline{H_3}CH{=}C$), 1.16 (d, *J* = 6.5 Hz, 6H, $CH(C\underline{H_3})_2$).
^{13}C NMR (100 MHz, $CDCl_3$): 141.4 ($HC{=}\underline{C}$), 127.6 ($H\underline{C}{=}C$), 36.0 ($\underline{C}H_2Br$), 28.4 ($\underline{C}H(CH_3)_2$), 21.1 ($CH(\underline{C}H_3)_2$), 13.5 ($\underline{C}H_3CH{=}C$).
FTIR: 1460, 1375, 1060 cm^{-1}.
MS: *Mass Spectrometry data was not obtained by either EI, CI or ESI techniques*.

(*Z*)-4-Isopropyl-1-(naphthalen-2-yl)hex-4-en-1-one

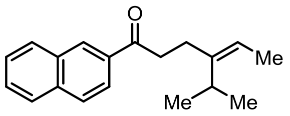

General Procedure C: Methyl 3-(naphthalene-2-yl)-3-oxopropanoate (0.86 g, 3.78 mmol) and (*E*)-(3-(bromomethyl)-4-methylpent-2-ene (0.80 g, 4.54 mmol) was employed. FCC (hexane:EtOAc 60:1) afforded the target ketone (0.82 g, 82%) as a colorless oil.
^{1}H NMR (400 MHz, $CDCl_3$): 8.49 (s, 1H, $Ar\underline{H}$), 8.06 (dd, *J* = 8.0 and 1.5 Hz, 1H, $Ar\underline{H}$), 7.98 (d, *J* = 8.0 Hz, 1H, $Ar\underline{H}$), 7.90 (t, *J* = 8.0 Hz, 2H, $Ar\underline{H}$), 7.64-7.52 (m, 2H, $Ar\underline{H}$), 5.23 (q, *J* = 7.0 Hz, 1H, $C{=}C\underline{H}$), 3.27-3.18 (m, 2H, $Ar(CO)C\underline{H_2}$), 2.94 (sept. *J* = 6.5 Hz, 1H, $C\underline{H}(CH_3)_2$), 2.45 (t, *J* = 7.0 Hz, 2H, $Ar(CO)CH_2C\underline{H_2}$), 1.65 (d, *J* = 7.0 Hz, 3H, $C{=}CHC\underline{H_3}$), 1.06 (d, *J* = 6.5 Hz, 6H, $CH(C\underline{H_3})_2$).
^{13}C NMR (100 MHz, $CDCl_3$): 200.2 ($\underline{C}{=}O$), 144.0 ($\underline{C}{=}CH$), 135.5, 134.4, 132.6 (3 × $Ar\underline{C}$), 129.6, 129.5, 128.4, 128.3, 127.8, 126.7, 124.0 (7 × $Ar\underline{C}H$), 117.0 ($C{=}\underline{C}H$), 38.2 ($Ar(CO)\underline{C}H_2$), 28.6 ($\underline{C}H(CH_3)_2$), 25.4 ($Ar(CO)CH_2\underline{C}H_2$), 20.9 ($CH(\underline{C}H_3)_2$), 12.8 ($C{=}CH\underline{C}H_3$).
FTIR: 2960, 1681, 1467, 1360, 1182, 1123 cm^{-1}.
MS: (ESI^+) Found $[M+H]^+$: 267.1741, $C_{19}H_{23}O$ requires 267.1743.

(4*Z*)-4-Isopropyl-1-(naphthalene-2-yl)hex-4-en-1-one *O*-perfluorobenzoyl oxime 2j

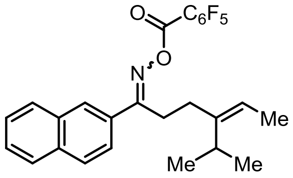

General Procedure D: Part A: (*Z*)-4-Isopropyl-1-(naphthalen-2-yl)hex-4-en-1-one (0.82 g, 3.08 mmol) was used. The reaction was heated at 75 °C for 2 hours to afford the corresponding oxime (0.81 g, 94%) as a colorless oil. Part B: The corresponding oxime (0.81 g, 2.88 mmol) was employed and the reaction was stirred for 2 hours. FCC (hexane:EtOAc 40:1) afforded the title compound (0.85 g, 62%, 1:0.1 mixture of oxime isomers) as a yellow solid.

^{1}H NMR (400 MHz, $CDCl_3$): *Signals for the major isomer:* 8.20 (s, 1H, ArH), 7.98-7.85 (m, 4H, ArH), 7.61-7.52 (m, 2H, ArH), 5.26 (q, *J* = 7.0 Hz, 1H, C=CH), 3.13-3.07 (m, 2H, Ar(CN)CH_2), 2.88 (sept. *J* = 6.5 Hz, 1H, $CH(CH_3)_2$), 2.24 (t, *J* = 7.0 Hz, 2H, Ar(CN)CH_2CH_2), 1.65 (d, *J* = 7.0 Hz, 3H, C=$CHCH_3$), 0.94 (d, *J* = 6.5 Hz, 6H, $CH(CH_3)_2$). *Characteristic signals only for the minor isomer:* 0.91 (d, *J* = 6.5 Hz, 0.6H, $CH(CH_3)_2$).

^{13}C NMR (100 MHz, $CDCl_3$): *Signals for the major isomer only:* 168.7 (C=N), 143.4 (C=CH), 134.5, 132.9, 130.4 (3 × ArC), 128.8, 128.6, 128.0, 127.7, 127.6, 126.7, 123.9 (7 × ArCH), 118.1 (C=CH), 28.8 (Ar(CN)CH_2), 28.4 ($CH(CH_3)_2$), 28.0 (Ar(CN)CH_2CH_2), 20.7 ($CH(CH_3)_2$), 12.8 (C=$CHCH_3$). *Signals corresponding to the perfluorinated benzoyl ester moiety were not resolvable due to their anticipated weak intensity.*

^{19}F NMR (376 MHz, $CDCl_3$): *Signals for the major isomer:* -136.9 (2F), -147.4 (1F), -159.7 (2F). *Signals for the minor isomer*: -137.4 (0.2F), -148.1 (0.1F), -160.0 (0.2F).

FTIR: 1755, 1524, 1491, 1324, 1190 cm^{-1}.

MS: (ESI^+) Found $[M+Na]^+$: 498.1461, $C_{26}H_{22}NO_2F_5Na$ requires 498.1463.

(*S*)-2-Isopropyl-5-(naphthalen-2-yl)-2-vinyl-3,4-dihydro-2*H*-pyrrole 3j

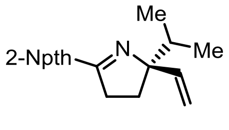

General Procedure E: Oxime ester **2j** (50.0 mg, 0.105 mmol) was employed and the reaction was heated at 120 °C for 1.75 hours. FCC (hexane:EtOAc 60:1) afforded the title compound (19 mg, 69%, 93:7 e.r.) as a pale yellow solid.

$[\alpha]^{22}_{D}$ +25.0 (c = 0.40, $CHCl_3$).

M.P. 71-72 °C (CH_2Cl_2-pentane).

^{1}H NMR (400 MHz, $CDCl_3$): 8.19 (s, 1H, ArH), 8.14 (dd, *J* = 8.5 and 1.5 Hz, 1H, ArH), 7.94-7.82 (m, 3H, ArH), 7.56-7.48 (m, 2H, ArH), 6.18-6.08 (m, 1H, $HC=CH_2$), 5.11-5.04 (m, 2H, $HC=CH_2$), 3.13-

2.94 (m, 2H, Ar(CN)CH_2), 2.13-1.90 (m, 3H, Ar(CN)CH_2CH_2 and CH(CH_3)$_2$), 1.05 (d, J = 6.5 Hz, 3H, CH(CH_3)$_2$), 0.96 (d, J = 6.5 Hz, 3H, CH(CH_3)$_2$).

^{13}C NMR (100 MHz, $CDCl_3$): 170.8 (C=N), 141.8 (HC=CH_2), 134.3, 133.0, 132.4 (3 × ArC), 128.6, 128.0, 127.9, 127.7, 126.9, 126.3, 124.9 (7 × ArCH), 112.4 (HC=CH_2), 83.7 (quaternary C), 36.9 (CH(CH_3)$_2$), 34.9 (Ar(CN)CH_2), 28.9 (Ar(CN)$CH_2$$CH_2$), 17.9, 17.6 (CH($CH_3$)$_2$).

FTIR: 1613, 1468, 1363, 1126 cm^{-1}.

MS: (ESI$^+$) Found [M+H]$^+$: 264.1755, $C_{19}H_{22}N$ requires 264.1747.

The enantiomeric purity of this compound was determined by chiral HPLC (Chiralpak IA, isocratic hexane-*i*-PrOH (99:1, 0.5 mL/min, 20 °C) against a racemic standard prepared under similar conditions using P(3,5-$(CF_3)_2C_6H_3)_3$ (15 mol%) as ligand; t_R (major) = 10.6 min and t_R (minor) = 11.6 min.

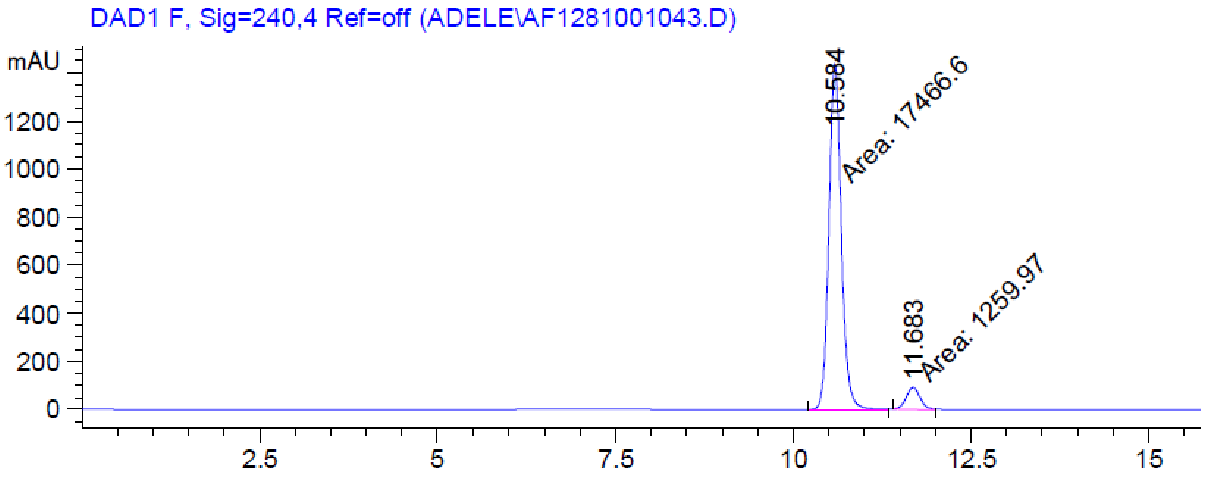

Peak #	RetTime [min]	Type	Width [min]	Area [mAU*s]	Height [mAU]	Area %
1	10.584	MM	0.2024	1.74666e4	1438.08533	93.2717
2	11.683	MM	0.2295	1259.96826	91.48811	6.7283

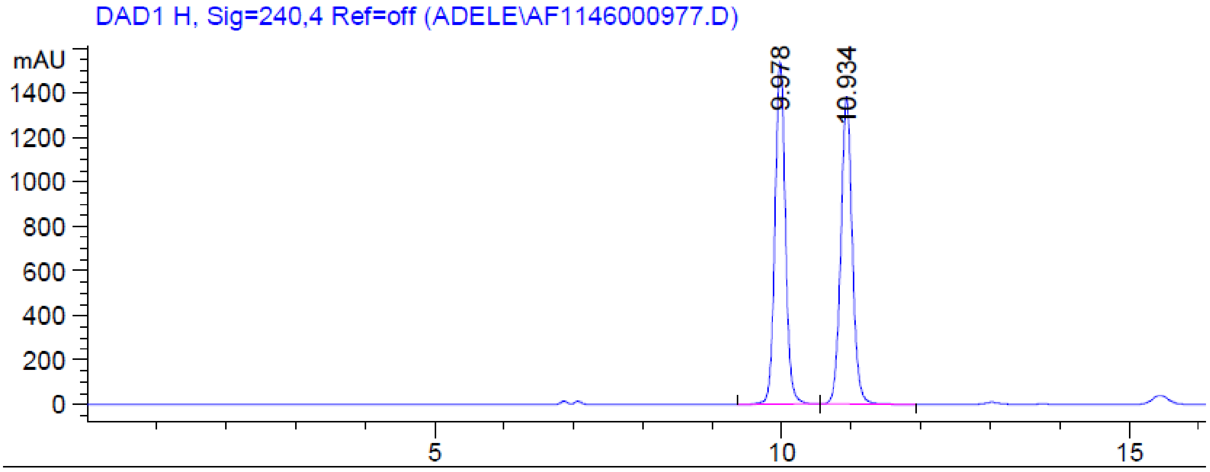

Peak #	RetTime [min]	Type	Width [min]	Area [mAU*s]	Height [mAU]	Area %
1	9.978	BB	0.1593	1.59592e4	1535.26404	50.0121
2	10.934	BB	0.1767	1.59514e4	1381.67615	49.9879

(*E*)-4-Methyl-1-(naphthalen-2-yl)-6-phenylhex-4-en-1-one

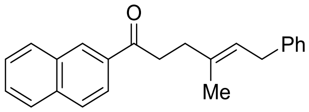

General Procedure C: Methyl 3-(naphthalen-2-yl)-3-oxopropanoate (0.65 g, 2.84 mmol) and (*E*)-(4-bromo-3-methylbut-2-en-yl)benzene[13] (0.70 g, 3.12 mmol) were employed. FCC (hexane:EtOAc 45:1) afforded the title compound (0.59 g, 66%) as a colorless oil.

^{1}H NMR (400 MHz, $CDCl_3$): 8.48 (s, 1H, ArH), 8.04 (dd, *J* = 8.0 and 1.5 Hz, 2H, ArH), 7.97 (d, *J* = 8.0 Hz, 2H, ArH), 7.93-7.86 (m, 2H, ArH), 7.32-7.26 (m, 2H, ArH), 7.22-7.16 (m, 3H, ArH), 5.45 (tq, *J* = 7.5 and 1.5 Hz, 1H, C=CH), 3.40 (d, *J* = 8.0 Hz, 2H, CH_2Ar), 3.28-3.21 (t, *J* = 7.0 Hz, 2H, Ar(CO)CH_2), 2.56 (t, *J* = 7.0 Hz, 2H, Ar(CO)CH_2CH_2), 1.83 (s, 3H, CH_3).

^{13}C NMR (100 MHz, $CDCl_3$): 200.0 (C=O), 141.4, 135.5 (2 × ArC), 135.1 (C=CH), 134.3, 132.5 (2 × ArC), 129.6, 129.5, 128.4 (3 signals), 128.3, 127.8, 126.7, 125.8, 123.9 (10 × ArCH), 123.7 (C=CH), 37.3 (Ar(CO)CH_2), 34.2 (CH_2Ar), 34.1 (Ar(CO)CH_2CH_2), 16.5 (CH_3).

FTIR: 1680, 1493 cm^{-1}.

MS: (ESI^+) Found $[M+H]^+$: 315.1741, $C_{23}H_{23}O$ requires 315.1743.

(4*E*)-4-Methyl-1-(naphthalen-2-yl)-6-phenylhex-4-en-1-one *O*-perfluorobenzoyl oxime 2k

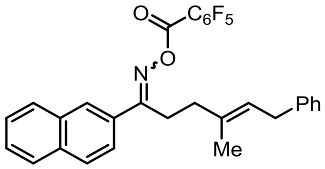

General Procedure D: Part A: (*E*)-4-Methyl-1-(naphthalen-2-yl)-6-phenylhex-4-en-1-one (0.58 g, 1.85 mmol) was used. The reaction was heated at 75 °C for 2 hours to afford the corresponding oxime (0.56 g, 92%) as a colorless solid. Part B: The corresponding oxime (0.56 g, 1.70 mmol) was employed and the reaction was stirred for 2 hours. FCC (hexane: EtOAc 50:1 to EtOAc) afforded the title compound (0.70 g, 79%, 1:0.25 mixture of oxime isomers) as a colorless solid.

^{1}H NMR (400 MHz, $CDCl_3$): *Signals for both isomers:* 8.17 (s, 1H, maj. ArH), 7.92-7.74 (m, 5H, ArH), 7.61-7.49 (m, 2.5H, ArH), 7.36- 7.10 (m, 6.5H, ArH), 5.38 (tq, *J* = 7.5 and 1.0 Hz, 1H, maj. C=CH), 5.29 (tq, *J* = 7.5 and 1.0 Hz, 0.25H, min. C=CH), 3.37-3.31 (m, 2.5H, min. and maj. CH_2Ar), 3.16 (t, *J* = 8.0 Hz, 2H, maj. Ar(CN)CH_2), 3.02 (t, *J* = 8.0 Hz, 0.5H, min. Ar(CN)CH_2), 2.37 (t, *J* = 8.0 Hz, 2H,

maj. Ar(CN)$CH_2C\underline{H_2}$), 2.27 (t, *J* = 8.0 Hz, 0.5H, maj. Ar(CN)$CH_2C\underline{H_2}$), 1.76 (s, 3.75H, min. and maj. $C\underline{H_3}$).

^{13}C NMR (100 MHz, $CDCl_3$): *Signals for the major isomer only:* 168.4 (C=N), 141.1, 134.5 (2 × ArC), 134.0 (C=CH), 132.9, 130.4 (2 × ArC), 128.8, 128.6, 128.4, 128.2, 128.0, 127.7, 127.6, 126.7, 125.9 (9 × ArCH), 125.0 (C=CH), 124.0 (ArCH), 36.5 (Ar(CN)CH_2CH_2), 34.2 (CH_2Ar), 27.6 (Ar(CN)CH_2), 16.0 (CH_3). *Signals corresponding to the perfluorinated benzoyl ester moiety were not resolvable due to their anticipated weak intensity.*

^{19}F NMR (376 MHz, $CDCl_3$): *Signals for the major isomers:* -136.9 (2F), -147.3 (1F), -159.6 (2F), *Signals for the minor isomer:* -137.2 (0.5F), -148.0 (0.25F), -159.9 (0.5F).

FTIR: 1754, 1524, 1492, 1321, 1190 cm^{-1}.

MS: (ESI^+) Found $[M+Na]^+$: 546.1464, $C_{30}H_{22}NO_2F_5Na$ requires 546.1463.

(*R*,*E*)-2-Methyl-5-(naphthalen-2-yl)-2-styryl-3,4-dihydro-2*H*-pyrrole 3k

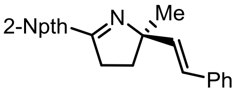

General Procedure E: Oxime ester **2k** (55.0 mg, 0.105 mmol) was employed and the reaction was heated at 120 °C for 2 hours. FCC (toluene:EtOAc 80:1) afforded the title compound (25 mg, 76%, 94:6 e.r.) as a pale yellow solid.

$[\alpha]_D^{23}$ +33.3 (c = 0.51, $CHCl_3$).

M.P. 112-113 °C (CH_2Cl_2-pentane).

^{1}H NMR (400 MHz, $CDCl_3$): 8.24 (s, 1H, ArH), 8.16 (d, *J* = 8.0 Hz, 1H, ArH), 7.95-7.82 (m, 3H, ArH), 7.57-7.48 (m, 2H, ArH), 7.44-7.17 (m, 5H, ArH), 6.54 (d, *J* = 16.0 Hz, 1H, HC=CHAr), 6.49 (d, *J* = 16.0 Hz, 1H, HC=CHAr), 3.28-3.10 (m, 2H, Ar(CN)CH_2), 2.30-2.19 (m, 1H, (CN)CH_2CH_2), 2.11-2.00 (m, 1H, (CN)CH_2CH_2), 1.59 (s, 3H, CH_3).

^{13}C NMR (100 MHz, $CDCl_3$): 171.5 (C=N), 137.4 (ArC), 136.4 (HC=CHAr), 134.4, 133.0, 132.2 (3 × ArC), 128.7, 128.5, 128.2, 128.1, 127.8, 127.1 (2 signals) (7 × ArCH), 126.6 (HC=CHAr), 126.4 (2 signals), 124.8 (3 × ArCH), 76.6 (quaternary C), 36.1 (Ar(CN)CH_2CH_2), 35.0 (Ar(CN)CH_2), 27.6 (CH_3).

FTIR: 1611, 1279, 1132 cm^{-1}.

MS: (ESI^+) Found $[M+H]^+$:312.1745, $C_{23}H_{22}N$ requires 312.1747.

The enantiomeric purity of this compound was determined by chiral HPLC (Chiralpak IB, isocratic hexane-*i*-PrOH (90:10, 0.5 mL/min, 20 °C) against a racemic standard prepared under similar conditions using $P(3,5-(CF_3)_2C_6H_3)_3$ (15 mol%) as ligand; t_R (minor) = 10.1 min and t_R (major) = 11.0 min.

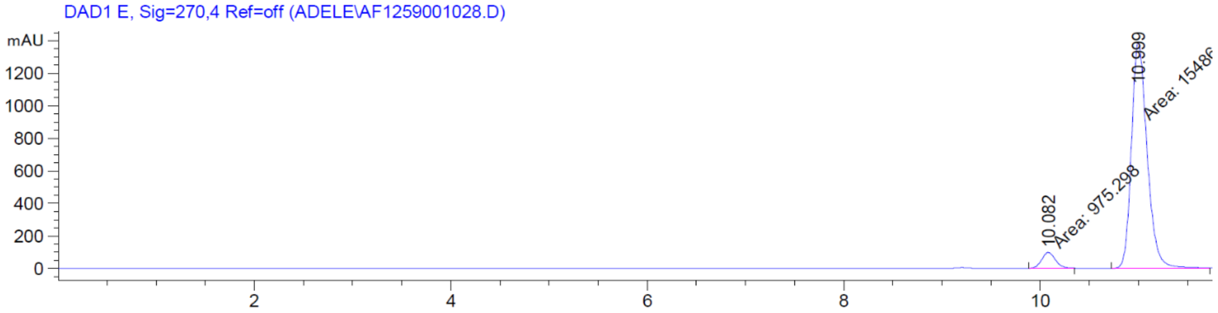

Peak #	RetTime [min]	Type	Width [min]	Area [mAU*s]	Height [mAU]	Area %
1	10.082	MM	0.1638	975.29828	99.23787	5.9245
2	10.999	MM	0.1855	1.54867e4	1391.38110	94.0755

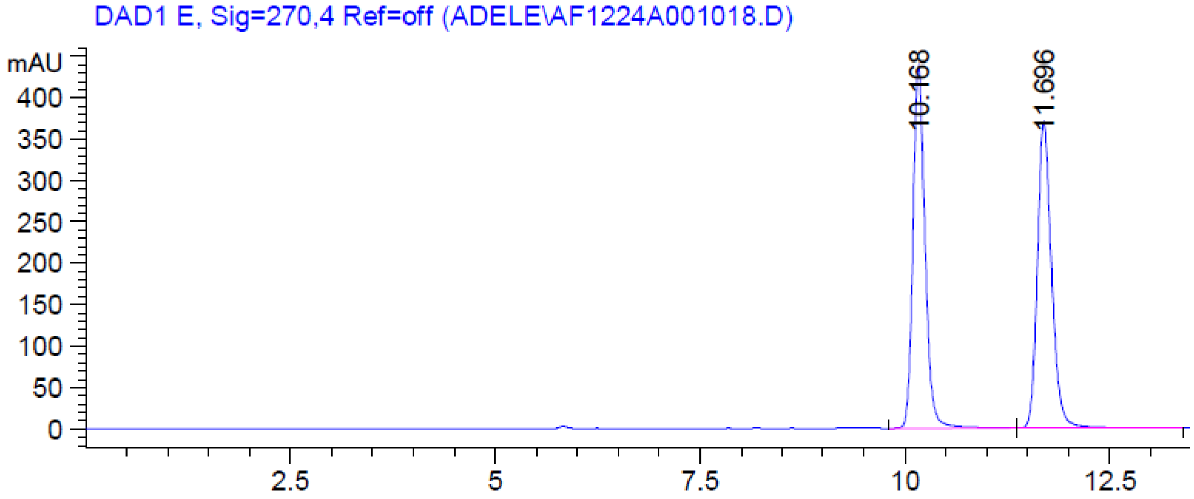

Peak #	RetTime [min]	Type	Width [min]	Area [mAU*s]	Height [mAU]	Area %
1	10.168	BB	0.1581	4505.23633	437.77521	49.9809
2	11.696	BB	0.1860	4508.68408	370.51431	50.0191

Methyl 3-oxo-3-(4-(trifluoromethyl)phenyl)propanoate and Methyl (*Z*)-3-hydroxy-3-(4-(trifluoromethyl)phenyl)acrylate

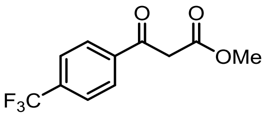

A

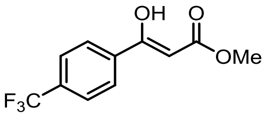

B

To a suspension of NaH (0.34 g, 8.55 mmol) in THF (10 mL) at room temperature was added dimethyl carbonate (0.61 mL, 7.20 mmol). 1-(4-(Trifluoromethyl)phenyl)ethan-1-one (1.34 g, 7.13 mmol) was added and the reaction was heated at 75 °C for 16 hours. The reaction was cooled to room temperature and then concentrated *in vacuo*. To the residue was added ice-cold water (30 mL) and aq. 1M HCl (30

mL) and the mixture was extracted with EtOAc (2 × 50 mL). The organic extracts were combined, dried (Na_2SO_4) and concentrated *in vacuo*. The residue was purified by FCC (hexane:EtOAc 30:1) to afford the title compound (1.37 g, 78%, 1:0.5 mixture of keto and enol tautomers) as a pale yellow oil.

^{1}H NMR (400 MHz, $CDCl_3$): *Signals for both tautomers:* 12.47 (s, 0.5H, **B**-OH), 8.05 (d, *J* = 8.0 Hz, 2H, **A**-ArH), 7.88 (d, *J* = 8.0 Hz, 1H, **B**-ArH), 7.75 (d, *J* = 8.0 Hz, 2H, **A**-ArH), 7.67 (d, *J* = 8.0 Hz, 1H, **B**-ArH), 5.72 (s, 0.5H, **B**-C=CH), 4.02 (s, 2H, **A**-CH_2), 3.82 (s, 1.5H, **B**-OCH_3), 3.75 (s, 3H, **A**-OCH_3).

^{13}C NMR (100 MHz, $CDCl_3$): *Signals for both tautomers*: 191.4 (**A**-Ar(CO), 173.2 (**B**-(CO)OCH_3), 169.6 (**B**-C(OH)), 167.4 (**A**-(CO)OCH_3), 138.5 (**A**-ArC(CO)), 136.7 (**B**-ArC(CO)), 135.0 (q, *J* = 33.5 Hz, **A**-F_3C-C), 132.8 (q, *J* = 33.0 Hz, **B**-F_3C-C), 128.9 (**A**-ArCH), 126.4 (**B**-ArCH), 125.9 (q, *J* = 4.0 Hz, **A**-ArCH), 125.6 (q, *J* = 4.0 Hz, **B**-ArCH), 123.8 (q, *J* = 272.0 Hz, **B**-CF_3), 123.5 (q, *J* = 272.0 Hz, **A**-CF_3), 88.7 (**B**-C=CH), 52.7 (**A**-OCH_3), 51.7 (**B**-OCH_3), 45.8 (**A**-CH_2).

^{19}F NMR (376 MHz, $CDCl_3$): -62.9 (1.5F), -63.1 (3F).

FTIR: 1744, 1694, 1321, 1125, 1112, 1067 cm^{-1}.

MS: (ESI^+) Found $[M+H]^+$: 269.0391, $C_{11}H_9F_3NaO_3$ requires 269.0396.

(*E*)-4-Methyl-6-phenyl-1-(4-(trifluoromethyl)phenyl)hex-4-en-1-one

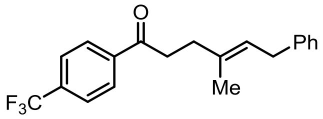

General Procedure C: Methyl 3-oxo-3-(4-(trifluoromethyl)phenyl)propanoate (0.60 g, 2.44 mmol) and (*E*)-(4-bromo-3-methylbut-2-en-yl)benzene[13] (0.60 g, 2.67 mmol) were employed. FCC (hexane:EtOAc 30:1) afforded the title compound (0.20 g, 25%) as a pale yellow oil.

^{1}H NMR (400 MHz, $CDCl_3$): 8.03 (d, *J* = 8.0 Hz, 2H, ArH), 7.71 (d, *J* = 8.0 Hz, 2H, ArH), 7.30-7.23 (m, 2H, ArH), 7.21-7.11 (m, 3H, ArH), 5.38 (t, *J* = 7.5 Hz, 1H, C=CH), 3.35 (d, *J* = 7.5 Hz, 2H, C=CHCH_2), 3.15-3.08 (m, 2H, Ar(CO)CH_2), 2.48 (t, *J* = 7.0 Hz, 2H, Ar(CO)CH_2CH_2), 1.78 (s, 3H, CH_3).

^{13}C NMR (100 MHz, $CDCl_3$): 198.9 (C=O), 141.3 (ArC), 139.6 (ArC(CO)), 134.6 (C=CH), 134.3 (q, *J* = 33.5 Hz, F_3CC), 128.4 (2 signals), 128.3, 125.8 (4 × ArCH), 125.7 (q, *J* = 4.0 Hz, ArCH), 124.0 (C=CH), 123.6 (q, *J* = 275.0 Hz, CF_3), 37.5 (Ar(CO)CH_2), 34.2 (C=CHCH_2Ar), 33.7 (Ar(CO)CH_2CH_2), 16.4 (CH_3).

^{19}F NMR (376 MHz, $CDCl_3$): -63.0.

FTIR: 1692, 1325, 1129, 1066 cm^{-1}.

MS: (ESI^+) Found $[M+H]^+$: 333.1449, $C_{20}H_{20}F_3O$ requires 333.1461.

(*E*)-4-Methyl-6-phenyl-1-(4-(trifluoromethyl)phenyl)hex-4-en-1-one *O*-perfluorobenzoyl oxime 2l

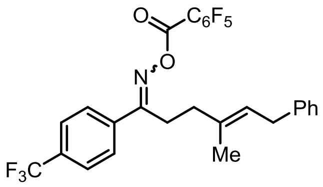

General Procedure D: Part A: (*E*)-4-Methyl-6-phenyl-1-(4-(trifluoromethyl)phenyl)hex-4-en-1-one (0.19 g, 0.572 mmol) was used. The reaction was heated at 75 °C for 2 hours to afford the corresponding oxime (0.16 g, 81%) as a colorless solid. Part B: The corresponding oxime (0.16 g, 0.46 mmol) was employed and the reaction was stirred for 2 hours. FCC (hexane: EtOAc 50:1to 40:1) afforded the title compound (0.16 g, 64%) as a colorless oil.

^{1}H NMR (400 MHz, $CDCl_3$): 7.79 (d, *J* = 8.0 Hz, 2H, ArH), 7.66 (d, *J* = 8.0 Hz, 2H, ArH), 7.29-7.24 (m, 2H, ArH), 7.21-7.08 (m, 3H, ArH), 5.30 (t, *J* = 7.5 Hz, 1H, C=CH), 3.30 (d, *J* = 7.5 Hz, 2H, C=CHCH_2), 3.08-3.01 (m, 2H, Ar(CN)CH_2), 2.28 (t, *J* = 7.0 Hz, 2H, Ar(CN)CH_2CH_2), 1.71 (s, 3H, CH_3).

^{13}C NMR (100 MHz, $CDCl_3$): 167.5 (C=N), 156.3 (C=O), 140.9 (ArC), 136.7 (ArC(CN)), 133.5 (C=CH), 132.8 (q, *J* = 34.0 Hz, F_3CC), 128.5, 128.2, 127.9, 126.0 (4 × ArCH), 125.8 (q, *J* = 4.0 Hz, ArCH), 125.4 (C=CH), 123.7 (q, *J* = 275.0 Hz, CF_3), 36.1 (Ar(CN)CH_2CH_2), 34.2 (C=CHCH_2Ar), 27.7 (Ar(CN)CH_2), 16.0 (CH_3).

^{19}F NMR (376 MHz, $CDCl_3$): -62.9 (3F), -136.8 (2F), -146.8 (1F), -159.4 (2F).

FTIR: 1767, 1497, 1323, 1191 cm^{-1}.

MS: (ESI^+) Found $[M+Na]^+$: 564.1177, $C_{27}H_{19}F_8NNaO_2$ requires 564.1180.

(*R*,*E*)-2-Methyl-2-styryl-5-(4-(trifluoromethyl)phenyl)-3,4-dihydro-2*H*-pyrrole 3l

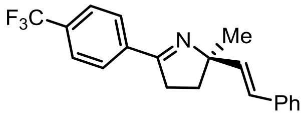

General Procedure E: Oxime ester **2l** (46.0 mg, 0.085 mmol) was employed and the reaction was heated at 120 °C for 1 hour. FCC (toluene:EtOAc 60:1) afforded the title compound (19 mg, 70%, 93:7 e.r.) as a pale yellow oil.

$[\alpha]_D^{23}$ +35.7 (c = 0.62, $CHCl_3$).

^{1}H NMR (400 MHz, $CDCl_3$): 8.00 (d, *J* = 8.0 Hz, 2H, ArH), 7.68 (d, *J* = 8.0 Hz, 2H, ArH), 7.37 (d, *J* = 7.5 Hz, 2H, ArH), 7.32-7.16 (m, 3H, ArH), 6.48 (d, *J* = 16.0 Hz, 1H, HC=CH), 6.44 (d, *J* = 16.0 Hz, 1H, HC=CH), 3.14-2.97 (m, 2H, Ar(CN)CH_2), 2.26-2.15 (m, 1H, Ar(CN)CH_2CH_2), 2.07-1.96 (m, 1H, Ar(CN)CH_2CH_2), 1.54 (s, 3H, CH_3).

^{13}C NMR (100 MHz, $CDCl_3$): 170.3 (C=N), 137.9 (ArC(CN)), 137.2 (ArC), 135.9 (HC=CHAr), 132.1 (q, *J* = 33.5 Hz, F_3CC), 128.5, 128.1, 127.2 (3 × ArCH), 126.8 (HC=CHAr), 126.3 (ArCH), 125.4 (q,

J = 4.0 Hz, ArCH), 124.0 (q, *J* = 275.0 Hz, CF_3), 76.8 (quaternary C), 35.9 (Ar(CN)CH_2CH_2), 35.0 (Ar(CN)CH_2), 27.5 (CH_3).

^{19}F NMR (376 MHz, $CDCl_3$): -62.6.

FTIR: 1323, 1273, 1132 cm^{-1}.

MS: (ESI$^+$) Found $[M+H]^+$: 330.1467, $C_{20}H_{19}F_3N$ requires 330.1464.

The enantiomeric purity of this compound was determined by chiral HPLC (Chiralpak IB, isocratic hexane-*i*-PrOH (95:5, 0.5 mL/min, 20 °C) against a racemic standard prepared under similar conditions using P(3,5-$(CF_3)_2C_6H_3)_3$ (15 mol%) as ligand; t_R (major) = 8.3 min and t_R (minor) = 9.5 min.

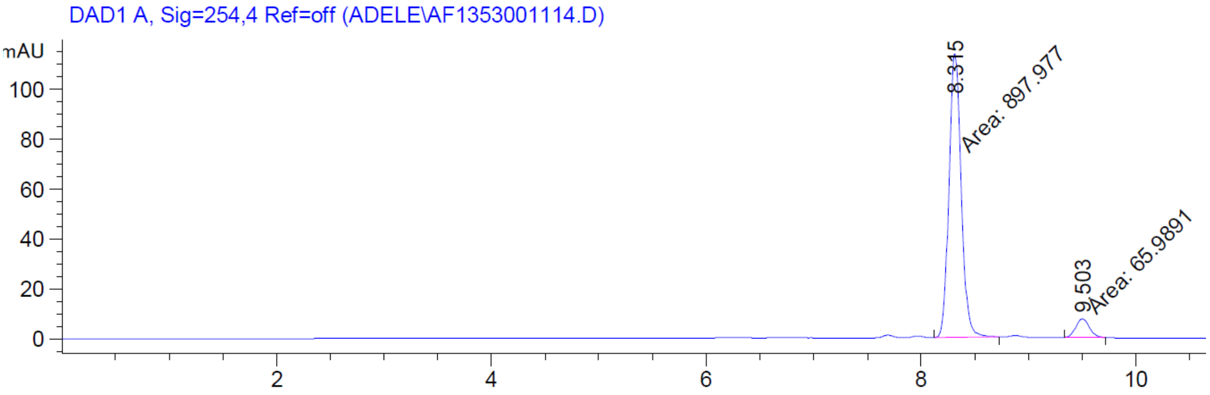

Peak #	RetTime [min]	Type	Width [min]	Area [mAU*s]	Height [mAU]	Area %
1	8.315	MM	0.1314	897.97705	113.91364	93.1544
2	9.503	MM	0.1474	65.98911	7.45956	6.8456

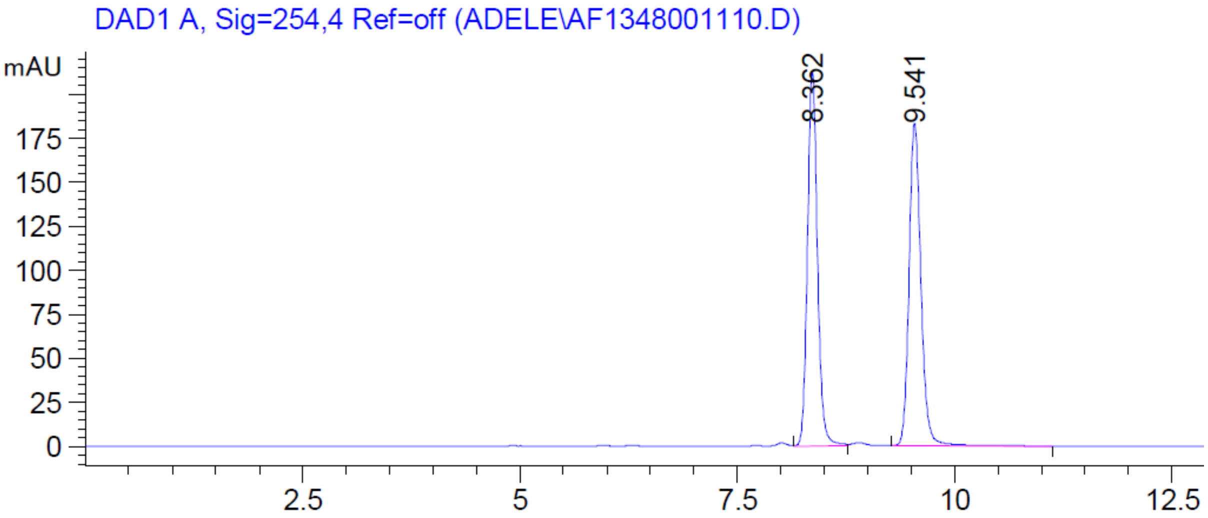

Peak #	RetTime [min]	Type	Width [min]	Area [mAU*s]	Height [mAU]	Area %
1	8.362	VV	0.1239	1717.10730	213.30144	49.8550
2	9.541	BB	0.1458	1727.09558	183.44353	50.1450

Methyl 3-(4-methoxyphenyl)-3-oxopropanoate and Methyl (Z)-3-hydroxy-3-(4-methoxyphenyl)acrylate

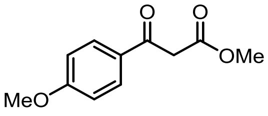

A

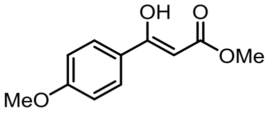

B

To a suspension of NaH (1.27 g, 31.92 mmol) in THF (40 mL) at room temperature was added dimethyl carbonate (2.27 mL, 26.87 mmol). 1-(4-methoxyphenyl)ethan-1-one (4.00 g, 26.60 mmol) was added and the reaction was heated at 75 °C for 16 hours. The reaction was cooled to room temperature and then concentrated *in vacuo*. To the residue was added ice-cold water (50 mL) and aq. 1M HCl (50 mL) and the mixture was extracted with EtOAc (2 × 100 mL). The organic extracts were combined, dried (Na_2SO_4) and concentrated *in vacuo*. The residue was purified by FCC (hexane:EtOAc 50:1 to 10:1) to afford the title compound (4.02 g, 72%, 1:0.1 mixture of keto and enol tautomers) as a pale yellow oil.

^{1}H NMR (400 MHz, $CDCl_3$): *Signals for both tautomers:* 12.53 (s, 0.1H, **B**-OH), 7.91 (d, *J* = 8.0 Hz, 2H, **A**-ArH), 7.75-7.70 (m, 0.2H, **B**-ArH), 6.97-6.88 (m, 2.2H, **A**-ArH and **B**-ArH), 5.58 (s, 0.1H, **B**-C=CH), 3.95 (s, 2H, **A**-CH_2), 3.86 (s, 3H, **A**-Ar-OCH_3), 3.84 (s, 0.3H, **B**-OCH_3), 3.78 (s, 0.3H, **B**-OCH_3), 3.74 (s, 3H, **A**-(CO)OCH_3).

^{13}C NMR (100 MHz, $CDCl_3$): *Signals for the keto tautomer:* 190.8 (Ar(C=O)), 168.1 ((CO)OCH_3), 164.0 (ArC(OCH_3), 130.9 (ArCH), 129.0 (ArC(CO)), 114.0 (ArCH), 55.5 (Ar(OCH_3), 52.4 ((CO)OCH_3), 45.5 (CH_2).

The spectroscopic properties of this compound were consistent with the data in the literature.[21]

(*E*)-1-(4-Methoxyphenyl)-4-methyl-6-phenylhex-4-en-1-one

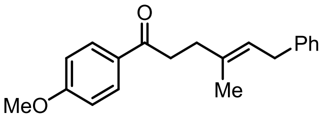

General Procedure C: Methyl 3-(4-methoxyphenyl)-3-oxopropanoate (0.46 g, 2.23 mmol) and (*E*)-(4-bromo-3-methylbut-2-en-yl)benzene[13] (0.55 g, 2.45 mmol) were employed. FCC (hexane:EtOAc 50:1 to 30:1) afforded the title compound (0.58 g, 89%) as a pale yellow oil.

^{1}H NMR (400 MHz, $CDCl_3$): 7.94 (d, *J* = 8.0 Hz, 2H, ArH), 7.31-7.12 (m, 5H, ArH), 6.92 (d, *J* = 8.0 Hz, 2H, ArH), 5.40 (t, *J* = 7.5 Hz, 1H, C=CH), 3.86 (s, 3H, OCH_3), 3.36 (d, *J* = 7.5 Hz, 2H, C=CHCH_2Ar), 3.04 (t, *J* = 7.5 Hz, 2H, Ar(CO)CH_2), 2.46 (t, *J* = 7.5 Hz, 2H, Ar(CO)CH_2CH_2), 1.78 (s, 3H, CH_3).

^{13}C NMR (100 MHz, $CDCl_3$): 198.6 (C=O), 163.3 (ArC(OCH_3), 141.5 (ArC), 135.2 (C=CH), 130.3 (ArCH), 130.1 (ArC(CO)), 128.4, 128.3, 125.7 (3 × ArCH), 123.5 (C=CH), 113.7 (ArCH), 55.4 (Ar(OCH_3), 37.0 (Ar(CO)CH_2), 34.2 (2 signals) ((C=CHCH_2Ar and Ar(CO)CH_2CH_2), 16.4 (CH_3).

FTIR: 1675, 1599, 1256, 1169 cm^{-1}.

MS: (ESI^+) Found $[M+H]^+$: 295.1687, $C_{20}H_{23}O_2$ requires 295.1693.

(4*E*)-1-(4-methoxyphenyl)-4-methyl-6-phenylhex-4-en-1-one *O*-perfluorobenzoyl oxime 2m

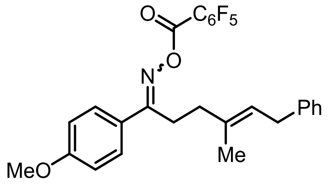

General Procedure D: Part A: (*E*)-1-(4-Methoxyphenyl)-4-methyl-6-phenylhex-4-en-1-one (0.57 g, 1.93 mmol) was used. The reaction was heated at 75 °C for 2 hours to afford the corresponding oxime (0.55 g, 92%) as a colorless solid. Part B: The corresponding oxime (0.55 g, 1.78 mmol) was employed and the reaction was stirred for 2 hours. FCC (hexane: EtOAc 40:1) afforded the title compound (0.65 g, 73%, 1:0.1 mixture of oxime isomers) as a colorless solid.

^{1}H NMR (400 MHz, $CDCl_3$): *Data for the major isomer:* 7.68 (d, *J* = 8.0 Hz, 2H, ArH), 7.30-7.08 (m, 5H, ArH), 6.91 (d, *J* = 8.0 Hz, 2H, ArH), 5.33 (t, *J* = 7.5 Hz, 1H, C=CH), 3.84 (s, 3H, OCH_3), 3.32 (d, *J* = 7.5 Hz, 2H, C=CHCH_2Ar), 2.99 (t, *J* = 7.5 Hz, 2H, Ar(CN)CH_2), 2.28 (t, *J* = 7.5 Hz, 2H, Ar(CN)CH_2CH_2), 1.72 (s, 3H, CH_3). *Characteristic signals for the minor isomer:* 5.24 (t, *J* = 7.5 Hz, 0.1H, C=CH), 3.82 (s, 0.3H, OCH_3), 2.87 (t, *J* = 7.5 Hz, 0.2H, Ar(CN)CH_2), 2.20 (t, *J* = 7.5 Hz, 0.2H, Ar(CN)CH_2CH_2),

^{13}C NMR (100 MHz, $CDCl_3$): 167.8 (C=N), 161.9 (ArC(OCH_3), 141.1 (ArC), 134.1 (C=CH), 129.0, 128.4, 128.2, 125.8 (4 × ArCH), 125.2 (ArC(CN)), 124.8 (C=CH), 114.2 (ArCH), 55.4 (Ar(OCH_3), 36.5 (Ar(CN)CH_2CH_2), 34.2 (C=CHCH_2Ar), 27.5 (Ar(CN)CH_2), 16.0 (CH_3).

^{19}F NMR (376 MHz, $CDCl_3$): *Data for the major isomer:*-137.1 (2F), -147.6 (1F), -159.7 (2F). *Data for the minor isomer:* -137.2 (0.2F), -148.1 (0.1F), -160.0 (0.2F).

FTIR: 1760, 1495, 1325, 1193, 1180 cm^{-1}.

MS: (ESI^+) Found $[M+Na]^+$: 526.1405, $C_{27}H_{22}F_5NNaO_3$ requires526.1412.

(*R*,*E*)-5-(4-Methoxyphenyl)-2-methyl-2-styryl-3,4-dihydro-2*H*-pyrrole 3m

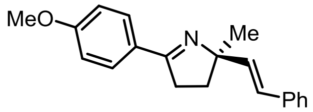

General Procedure E: Oxime ester **2m** (55 mg, 0.11 mmol) was employed and the reaction was heated at 120 °C for 1.25 hours. FCC (toluene:EtOAc 60:1) afforded the title compound (18 mg, 56%, 94:6 e.r.) as a pale yellow oil.

$[\alpha]^{23}_{D}$ +80.6 (c = 0.53, $CHCl_3$).

^{1}H NMR (400 MHz, $CDCl_3$): 7.85 (d, *J* = 8.0 Hz, 2H, ArH), 7.37 (d, *J* = 8.0 Hz, 2H, ArH), 7.31-7.14 (m, 3H, ArH), 6.93 (d, *J* = 8.0 Hz, 2H, ArH), 6.48 (d, *J* = 16.0 Hz, 1H, HC=CH), 6.44 (d, *J* = 16.0 Hz, 1H, HC=CH), 3.84 (s, 3H, OCH_3), 3.10-2.90 (m, 2H, Ar(CN)CH_2), 2.20-2.09 (m, 1H, Ar(CN)CH_2CH_2), 2.01-1.90 (m, 1H, Ar(CN)CH_2CH_2), 1.52 (s, 3H, CH_3).

^{13}C NMR (100 MHz, $CDCl_3$): 170.8 (C=N), 161.4 (ArC(OCH_3), 137.5 (ArC), 136.6 (HC=CHAr), 129.4, 128.4 (2 × ArCH), 127.5 (ArC(CN)), 127.0 (ArCH), 126.4, 126.3 (ArCH and HC=CHAr), 113.7 (ArCH), 76.2 (quaternary C), 55.3 (Ar(OCH_3), 36.0 (Ar(CN)CH_2), 34.9 (Ar(CN)CH_2CH_2), 27.6 (CH_3).

FTIR: 1605, 1514, 1251, 1172 cm^{-1}.

MS: (ESI^+) Found $[M+H]^+$: 292.1707, $C_{20}H_{22}NO$ requires 292.1696.

The enantiomeric purity of this compound was determined by chiral HPLC (Chiralpak IB, isocratic hexane-*i*-PrOH (90:10, 0.5 mL/min, 20 °C) against a racemic standard prepared under similar conditions using $P(3,5-(CF_3)_2C_6H_3)_3$ (15 mol%) as ligand; t_R (minor) = 10.7 min and t_R (major) = 13.8 min.

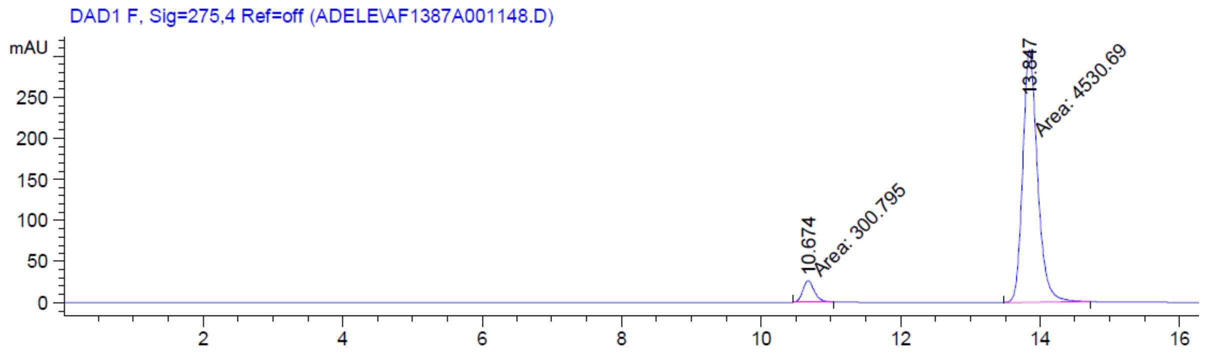

Peak #	RetTime [min]	Type	Width [min]	Area [mAU*s]	Height [mAU]	Area %
1	10.674	MM	0.1923	300.79514	26.06825	6.2257
2	13.847	MM	0.2457	4530.68896	307.32416	93.7743

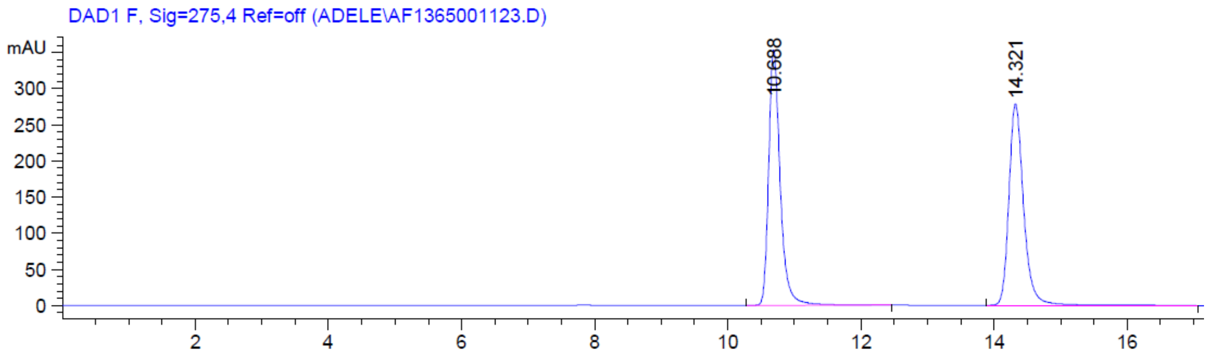

Peak #	RetTime [min]	Type	Width [min]	Area [mAU*s]	Height [mAU]	Area %
1	10.688	BB	0.1826	4253.07910	353.00546	49.9437
2	14.321	BB	0.2325	4262.66699	278.56393	50.0563

Methyl 3-(3-ethylphenyl)-3-oxopropanoate (A) and Methyl (*Z*)-3-(3-ethylphenyl)-3-hydroxyacrylate (B).

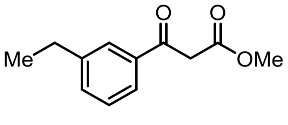

A

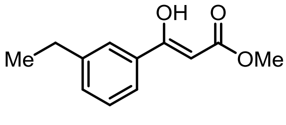

B

To a suspension of NaH (0.72 g, 18.20 mmol) in THF (30 mL) at room temperature was added dimethyl carbonate (1.30 mL, 15.4 mmol). 1-(3-Ethylphenyl)ethan-1-one (2.25 g, 15.2 mmol) was added and the reaction was heated at 75 °C for 16 hours. The reaction was cooled to room temperature and then concentrated *in vacuo*. To the residue was added ice-cold water (40 mL) and aq. 1M HCl (40 mL) and the mixture was extracted with EtOAc (2 × 80 mL). The organic extracts were combined, dried (Na_2SO_4) and concentrated *in vacuo*. The residue was purified by FCC (hexane:EtOAc 40:1) to afford the title compound (1.85 g, 59%, 1:0.3 mixture of keto and enol tautomers) as a pale yellow oil.

^{1}H NMR (400 MHz, $CDCl_3$): *Signals for both tautomers:* 12.49 (s, 0.3H, **B**-OH), 7.80-7.71 (m, 2H, **A**-Ar$\underline{H}$), 7.63-7.55 (m, 0.6H, **B**-Ar$\underline{H}$), 7.46-7.27 (m, 2.6H, **A**-Ar$\underline{H}$ and **B**-Ar$\underline{H}$), 5.66 (s, 0.3H, **B**-C=C$\underline{H}$), 4.00 (s, 2H, **A**-C$\underline{H_2}$), 3.79 (s, 0.9H, **B**-(CO)OC$\underline{H_3}$), 3.74 (s, 3H, **A**-(CO)OC$\underline{H_3}$), 2.75-2.64 (m, 2.6H, **A** and **B** C$\underline{H_2}$$CH_3$), 1.28-1.22 (m, 3.9H, **A** and **B** CH_2C$\underline{H_3}$).

^{13}C NMR (100 MHz, $CDCl_3$): *Signals for the keto tautomer:* 192.5 (Ar($\underline{C}$=O)), 168.0 (($\underline{C}$O)OCH_3), 145.0 (Ar$\underline{C}$$CH_2CH_3$), 136.0 (Ar$\underline{C}$(CO)), 133.5, 128.7, 127.8, 126.0 (Ar$\underline{C}$H), 52.4 ((CO)O$\underline{C}$$H_3$), 45.8 ((CO)$\underline{C}$$H_2$(CO)), 28.7 ($\underline{C}$$H_2CH_3$), 15.4 ($CH_2$$\underline{C}$$H_3$).

FTIR: 2966, 1743, 1684, 1436, 1212 cm^{-1}.

MS: (ESI^+) Found $[M+Na]^+$: 229.0839, $C_{12}H_{14}NaO_3$ requires 229.0835.

(*E*)-1-(3-Ethylphenyl)-4-methyl-6-phenylhex-4-en-1-one

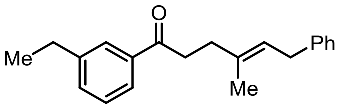

General Procedure C: Methyl 3-(3-ethylphenyl)-3-oxopropanoate (0.84 g, 4.05 mmol) and (*E*)-(4-bromo-3-methylbut-2-en-yl)benzene[13] (1.00 g, 4.46 mmol) were employed. FCC (hexane:EtOAc 50:1) afforded the title compound (1.00 g, 85%) as a pale yellow oil.

^{1}H NMR (400 MHz, $CDCl_3$): 7.80-7.73 (m, 2H, Ar$\underline{H}$), 7.42-7.13 (m, 7H, Ar$\underline{H}$), 5.40 (t, J = 7.5 Hz, 1H, C=C$\underline{H}$), 3.36 (d, J = 7.5 Hz, 2H, C=CHC$\underline{H_2}$Ar), 3.09 (t, J = 8.0 Hz, 2H, Ar(CO)C$\underline{H_2}$), 2.70 (q, J = 7.5 Hz, 2H, C$\underline{H_2}$$CH_3$), 2.47 (t, J = 8.0 Hz, 2H, Ar(CO)CH_2C$\underline{H_2}$), 1.78 (s, 3H, $\underline{H_3}$CC=CH), 1.25 (t, J = 7.5 Hz, 3H, CH_2C$\underline{H_3}$).

^{13}C NMR (100 MHz, $CDCl_3$): 200.2 ($\underline{C}$=O), 144.7 (Ar$\underline{C}$$CH_2CH_3$), 141.4 (Ar$\underline{C}$), 137.1 (Ar$\underline{C}$(CO)), 135.1 ($\underline{C}$=CH), 132.6, 128.5, 128.4, 128.3, 127.4, 125.8, 125.6 (7 × Ar$\underline{C}$H), 123.6 (C=C$\underline{H}$), 37.3 (Ar(CO)$\underline{C}$$H_2$), 34.2, 34.0 (C=CH$\underline{C}$$H_2$Ar and Ar(CO)$CH_2$$\underline{C}$$H_2$), 28.8 ($\underline{C}$$H_2CH_3$), 16.4 ($H_3$$\underline{C}$C=CH), 15.5 ($CH_2$$\underline{C}$$H_3$).

FTIR: 1683, 1452, 1157 cm^{-1}.

MS: (ESI^+) Found $[M+H]^+$: 293.1909, $C_{21}H_{25}O$ requires 293.1900.

(*E*)-1-(3-Ethylphenyl)-4-methyl-6-phenylhex-4-en-1-one *O*-perfluorobenzoyl oxime 2n

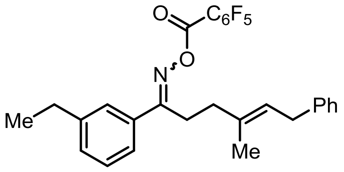

General Procedure D: Part A: (*E*)-1-(3-ethylphenyl)-4-methyl-6-phenylhex-4-en-1-one (0.25 g, 0.85 mmol) was used. The reaction was heated at 75 °C for 2 hours to afford the corresponding oxime (0.24 g, 92%) as a colorless solid. Part B: The corresponding oxime (0.24 g, 0.78 mmol) was employed and the reaction was stirred for 2 hours. FCC (hexane: EtOAc 50:1) afforded the title compound (0.37 g, 94%, 1:0.05 mixture of oxime isomers) as a colorless solid.

^{1}H NMR (400 MHz, $CDCl_3$): *Data for the major isomer:* 7.56-7.45 (m, 2H, ArH), 7.36-7.09 (m, 7H, ArH), 5.33 (t, *J* = 7.5 Hz, 1H, C=CH), 3.31 (d, *J* = 7.5 Hz, 2H, C=CHCH_2Ar), 3.02 (t, *J* = 8.0 Hz, 2H, Ar(CN)CH_2), 2.67 (q, *J* = 7.5 Hz, 2H, CH_2CH_3), 2.28 (t, *J* = 8.0 Hz, 2H, Ar(CN)CH_2CH_2), 1.71 (s, 3H, H_3CC=CH), 1.23 (t, *J* = 7.5 Hz, 3H, CH_2CH_3). *Characteristic signals for the minor isomer:* 2.91-2.84 (m, 0.1H, Ar(CN)CH_2), 2.61 (q, *J* = 7.5 Hz, 0.1H, CH_2CH_3), 1.18 (t, *J* = 7.5 Hz, 0.15H, CH_2CH_3).

^{13}C NMR (100 MHz, $CDCl_3$): 168.9 (C=N), 144.8 (ArCCH_2CH_3), 141.1 (ArC), 134.0 (C=CH), 133.1 (ArC(CN)), 130.6, 128.7, 128.4, 128.2, 126.9, 125.8 (6 × ArCH), 124.9, 124.8 (ArCH and C=CH), 36.3 (Ar(CN)CH_2CH_2), 34.2 (C=CHCH_2Ar), 28.8 (CH_2CH_3), 27.9 (Ar(CN)CH_2), 16.0 (H_3CC=CH), 15.5 (CH_2CH_3).

^{19}F NMR (376 MHz, $CDCl_3$): *Data for the major isomer:*-137.0 (2F), -147.5 (1F), -159.7 (2F). *Data for the minor isomer:* -137.2 (0.1F), -148.1 (0.05F), -160.1 (0.1F).

FTIR: 1764, 1523, 1497, 1325, 1190 cm^{-1}.

MS: (ESI^+) Found $[M+Na]^+$: 524.1624, $C_{28}H_{24}F_5NNaO_2$ requires 524.1619.

(*R*,*E*)-5-(3-Ethylphenyl)-2-methyl-2-styryl-3,4-dihydro-2*H*-pyrrole 3n

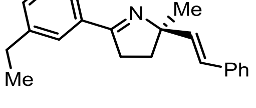

General Procedure E: Oxime ester **2n** (50.0 mg, 0.100 mmol) was employed and the reaction was heated at 120 °C for 1 hour. FCC (toluene to toluene:EtOAc 80:1) afforded the title compound (18.5 mg, 64%, 93:7 e.r.) as a pale yellow oil.

$[\alpha]_D^{23}$ +52.5 (c = 0.53, $CHCl_3$).

^{1}H NMR (400 MHz, $CDCl_3$): 7.80 (s, 1H, ArH), 7.66 (dt, *J* = 7.5 and 1.5 Hz, 1H, ArH), 7.41-7.15 (m, 7H, ArH), 6.49 (d, *J* = 16.0 Hz, 1H, HC=CH), 6.44 (d, *J* = 16.0 Hz, 1H, HC=CH), 3.14-2.92 (m, 2H,

Ar(CN)C<u>H</u>$_2$), 2.69 (q, J = 7.5 Hz, 2H, C<u>H</u>$_2$CH$_3$), 2.22-2.10 (m, 1H, Ar(CN)CH$_2$C<u>H</u>$_2$), 2.03-1.92 (m, 1H, Ar(CN)CH$_2$C<u>H</u>$_2$), 1.53 (s, 3H, <u>H</u>$_3$CC=CH), 1.26 (t, J = 7.5 Hz, 3H, CH$_2$C<u>H</u>$_3$).

^{13}C NMR (100 MHz, CDCl$_3$): 171.7 (<u>C</u>=N), 144.5 (Ar<u>C</u>CH$_2$CH$_3$), 137.4 (Ar<u>C</u>), 136.5 (<u>C</u>=CH), 134.6 (Ar<u>C</u>(CN)), 130.1, 128.4 (2 signals), 127.2, 127.1 (5 × Ar<u>C</u>H), 126.5 (C=C<u>H</u>), 126.3, 125.4 (2 × Ar<u>C</u>H), 76.4 (quaternary <u>C</u>), 36.0 (Ar(CN)CH$_2$<u>C</u>H$_2$), 35.1 (Ar(CN)<u>C</u>H$_2$), 28.8 (<u>C</u>H$_2$CH$_3$), 27.5 (H$_3$<u>C</u>C=CH), 15.7 (CH$_2$<u>C</u>H$_3$).

FTIR: 1615, 1600, 1581 cm^{-1}.

MS: (ESI$^+$) Found [M+H]$^+$: 290.1916, $C_{21}H_{24}N$ requires 290.1903.

The enantiomeric purity of this compound was determined by chiral HPLC (Chiralpak IB, isocratic hexane-*i*-PrOH (90:10, 0.5 mL/min, 20 °C) against a racemic standard prepared under similar conditions using P(3,5-(CF$_3$)$_2$C$_6$H$_3$)$_3$ (15 mol%) as ligand; t_R (minor) = 8.0 min and t_R (major) = 11.9 min.

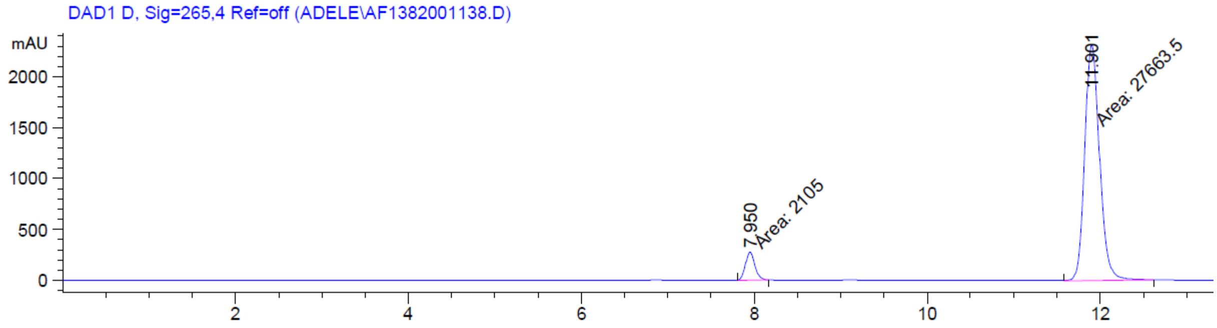

Peak #	RetTime [min]	Type	Width [min]	Area [mAU*s]	Height [mAU]	Area %
1	7.950	MM	0.1270	2104.99951	276.29443	7.0712
2	11.901	MM	0.1989	2.76635e4	2317.99878	92.9288

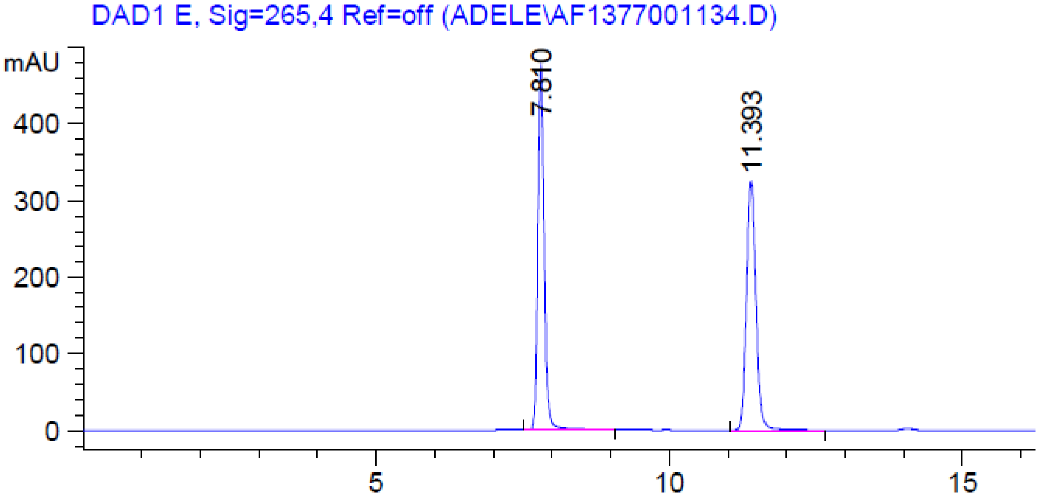

Peak #	RetTime [min]	Type	Width [min]	Area [mAU*s]	Height [mAU]	Area %
1	7.810	BB	0.1170	3638.02881	476.87405	50.1389
2	11.393	BB	0.1721	3617.86572	324.47696	49.8611

Methyl (*E*)-2-(cyclopropanecarbonyl)-4-methyl-6-phenylhex-4-enoate

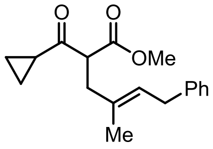

General Procedure C: Part A: Methyl 3-cyclopropyl-3-oxopropanoate (0.52 g, 3.65 mmol) and (*E*)-(4-bromo-3-methylbut-2-en-yl)benzene[13] (0.90 g, 4.01 mmol) were employed. FCC (toluene:hexane 5:2) afforded the title compound (0.55 g, 53%) as a colorless oil.

^{1}H NMR (400 MHz, $CDCl_3$): 7.31-7.23 (m, 2H, ArH), 7.21-7.10 (m, 3H, ArH), 5.41 (t, *J* = 7.5 Hz, 1H, C=CH), 3.83 (t, *J* = 7.5 Hz, 1H, (CO)CHCH$_2$C=CH), 3.67 (s, 3H, (CO)OCH$_3$), 3.34 (d, *J* = 7.5 Hz, 2H, C=CHCH$_2$Ar), 2.65 (d, *J* = 7.5 Hz, 2H, (CO)CHCH$_2$C=CH), 2.10-2.02 (m, 1H, CH(CH$_2$)$_2$), 1.75 (s, 3H, CH$_3$C=CH), 1.07-1.01 (m, 2H, CH(CH$_2$)$_2$), 0.93-0.86 (m, 2H, CH(CH$_2$)$_2$).

^{13}C NMR (100 MHz, $CDCl_3$): 204.8 (C=O), 170.1 ((CO)OCH$_3$), 141.0 (ArC), 132.1 (C=CH), 128.4, 128.3 (2 × ArCH), 125.9 (C=CH), 125.8 (ArCH), 58.5 ((CO)CHCH$_2$C=CH), 52.3 ((CO)OCH$_3$), 38.0 ((CO)CHCH$_2$C=CH), 34.2 (C=CHCH$_2$Ar), 19.7 (CH(CH$_2$)$_2$), 16.1 (CH$_3$C=CH), 11.9, 11.7 (CH(CH$_2$)$_2$).

FTIR: 1741, 1702 cm^{-1}.

MS: (ESI$^+$) Found [M+Na]$^+$: 309.1474, $C_{18}H_{22}O_3Na$ requires 309.1461.

(*E*)-1-Cyclopropyl-4-methyl-6-phenylhex-4-en-1-one

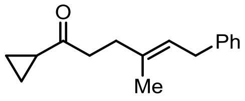

General Procedure C: Part B: Methyl (*E*)-2-(cyclopropanecarbonyl)-4-methyl-6-phenylhex-4-enoate (0.50 g, 1.76 mmol) was employed. FCC (hexane:EtOAc 30:1) afforded the title compound (0.37 g, 92%) as a colorless oil.

^{1}H NMR (400 MHz, $CDCl_3$): 7.32-7.25 (m, 2H, ArH), 7.22-7.14 (m, 3H, ArH), 5.41-5.34 (m, 1H, C=CH), 3.36 (d, *J* = 7.5 Hz, 2H, C=CHCH$_2$Ar), 2.69 (t, *J* = 7.5 Hz, 2H, (CO)CH$_2$CH$_2$), 2.36 (t, *J* = 7.5 Hz, 2H, (CO)CH$_2$CH$_2$), 1.97-1.88 (m, 1H, CH(CH$_2$)$_2$), 1.74 (s, 3H, CH$_3$C=CH), 1.04-0.97 (m, 2H, CH(CH$_2$)$_2$), 0.88-0.81 (m, 2H, CH(CH$_2$)$_2$).

^{13}C NMR (100 MHz, $CDCl_3$): 210.6 (C=O), 141.5 (ArC), 134.9 (C=CH), 128.4, 128.3, 125.8 (3 × ArCH), 123.5 (C=CH), 42.1 ((CO)CH$_2$CH$_2$), 34.2 (C=CHCH$_2$Ar), 33.7 ((CO)CH$_2$CH$_2$), 20.4 (CH(CH$_2$)$_2$), 16.3 (CH$_3$C=CH), 10.7 (CH(CH$_2$)$_2$).

FTIR: 1698, 1385, 1083 cm^{-1}.

MS: (ESI$^+$) Found $[M+Na]^+$: 251.1399, $C_{16}H_{20}ONa$ requires 251.1406.

(4*E*)-1-Cyclopropyl-4-methyl-6-phenylhex-4-en-1-one *O*-perfluorobenzoyl oxime 2o

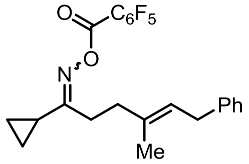

General Procedure D: Part A: (*E*)-1-Cyclopropyl-4-methyl-6-phenylhex-4-en-1-one (0.36 g, 1.58 mmol) was used. The reaction was heated at 75 °C for 1 hour to afford the corresponding oxime (0.35 g, 91%) as a colorless oil. Part B: The corresponding oxime (0.35 g, 1.44 mmol) was employed and the reaction was stirred for 2 hours. FCC (hexane:EtOAc 40:1) afforded the title compound (0.51 g, 81%, 1:0.5 mixture of oxime isomers) as a yellow oil.

^{1}H NMR (400 MHz, $CDCl_3$): *Signals for both isomers:* 7.30-7.24 (m, 3H, Ar$\underline{H}$), 7.21-7.13 (m, 4.5H, Ar$\underline{H}$), 5.41-5.35 (m, 1.5H, min. and maj. C=C$\underline{H}$), 3.38-3.32 (m, 3H, min. and maj. C=CHC$\underline{H_2}$Ar), 2.44-2.38 (m, 2H, maj. Ar(CN)C$\underline{H_2}$), 2.37-2.20 (m, 3.5H, min. and maj. Ar(CN)CH_2C$\underline{H_2}$ and min. C$\underline{H}$$(CH_2)_2$), 2.14-2.08 (m, 1H, min. Ar(CN)C$\underline{H_2}$), 1.76-1.65 (m, 5.5H, min. and maj. C$\underline{H_3}$C=CH and maj. C$\underline{H}$$(CH_2)_2$), 1.03-0.97 (m, 1H, min. CH(C$\underline{H_2}$)$_2$), 0.95-0.88 (m, 4H, maj. CH(C$\underline{H_2}$)$_2$), 0.88-0.82 (m, 1H, min. CH(C$\underline{H_2}$)$_2$).

^{13}C NMR (100 MHz, $CDCl_3$): *Signals for both isomers:* 173.1 (maj.$\underline{C}$=N), 171.9 (min.$\underline{C}$=N), 141.3 (min. Ar$\underline{C}$), 141.1 (maj. Ar$\underline{C}$), 134.7 (min. ($\underline{C}$=CH), 134.2 (maj. ($\underline{C}$=CH), 128.4 (2 signals), 128.3, 128.2, 125.9, 125.8 (3 × min. and maj. Ar$\underline{C}$H), 124.6, 124.2 (min. and maj. (C=$\underline{C}$H), 36.7, 36.1 (min. and maj. Ar(CN)$CH_2$$\underline{C}H_2$), 34.2 (2 signals) (min. and maj. (C=CH$\underline{C}H_2$Ar), 28.1, 27.8 (min. and maj. (Ar(CN)$\underline{C}H_2$), 16.2, 16.0 (min. and maj. $\underline{C}H_3$C=CH), 14.3 (maj. $\underline{C}$H$(CH_2)_2$), 10.2 (min. $\underline{C}$H$(CH_2)_2$), 6.9, 6.5 (min. and maj. (CH($\underline{C}H_2$)$_2$). *Signals corresponding to the perfluorinated benzoyl ester moiety were not resolvable due to their anticipated weak intensity.*

^{19}F NMR (376 MHz, $CDCl_3$): *Signals for both isomers:* -137.2 (3F), -147.9 (1F), -148.1 (0.5F), -159.9 (3F).

FTIR: 1759, 1523, 1497, 1325, 1200 cm^{-1}.

MS: (ESI$^+$) Found $[M+Na]^+$: 460.1301, $C_{23}H_{21}NO_2F_5Na$ requires 460.1306.

(*R*,*E*)-5-Cyclopropyl-2-methyl-2-styryl-3,4-dihydro-2*H*-pyrrole 3o

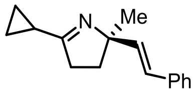

General Procedure E: Oxime ester **2o** (50.0 mg, 0.114 mmol) was employed and the reaction was heated at 120 °C for 3 hours. FCC (hexane:EtOAc 4:1 to 2:1) afforded the title compound (15.5 mg, 61%, 90:10 e.r.) as a pale yellow oil.

$[\alpha]_D^{23}$ +81.0 (c = 0.33, $CHCl_3$).

^{1}H NMR (400 MHz, $CDCl_3$): 7.39-7.34 (m, 2H, ArH), 7.31-7.35 (m, 2H, ArH), 7.19 (tt, *J* = 8.0 and 1.5 Hz, 1H, ArH), 6.41 (d, *J* = 16.0 Hz, 1H, CH=CHAr), 6.34 (d, *J* = 16.0 Hz, 1H, CH=CHAr), 2.47-2.32 (m, 2H, (CN)CH_2), 2.02-1.92 (m, 1H, (CN)CH_2CH_2), 1.88-1.72 (m, 2H, (CN)CH_2CH_2 and CH$(CH_2)_2$), 1.39 (s, 3H, (NC)CH_3), 0.93-0.80 (m, 4H, CH$(CH_2)_2$).

^{13}C NMR (100 MHz, $CDCl_3$): 177.8 (C=N), 137.5 (ArC), 136.6 (CH=CHAr), 128.4, 127.0, 126.3 (3 × ArCH), 126.1 (CH=CHAr), 75.4 (quaternary C), 35.6 ((CN)CH_2CH_2), 34.2 ((CN)CH_2), 27.4 ((NC)CH_3), 14.4 (CH$(CH_2)_2$), 7.4, 7.2 (CH$(CH_2)_2$).

FTIR: 1631, 1448 cm^{-1}.

MS: (ESI^+) Found $[M+H]^+$: 226.1590, $C_{16}H_{20}N$ requires 226.1590.

The enantiomeric purity of this compound was determined by chiral HPLC (Chiralpak IA, isocratic hexane-*i*-PrOH-diethylamine (100:1:0.1, 0.5 mL/min, 20 °C) against a racemic standard prepared under similar conditions using P(3,5-$(CF_3)_2C_6H_3)_3$ (15 mol%) as ligand; t_R (major) = 12.4 min and t_R (minor) = 18.8 min.

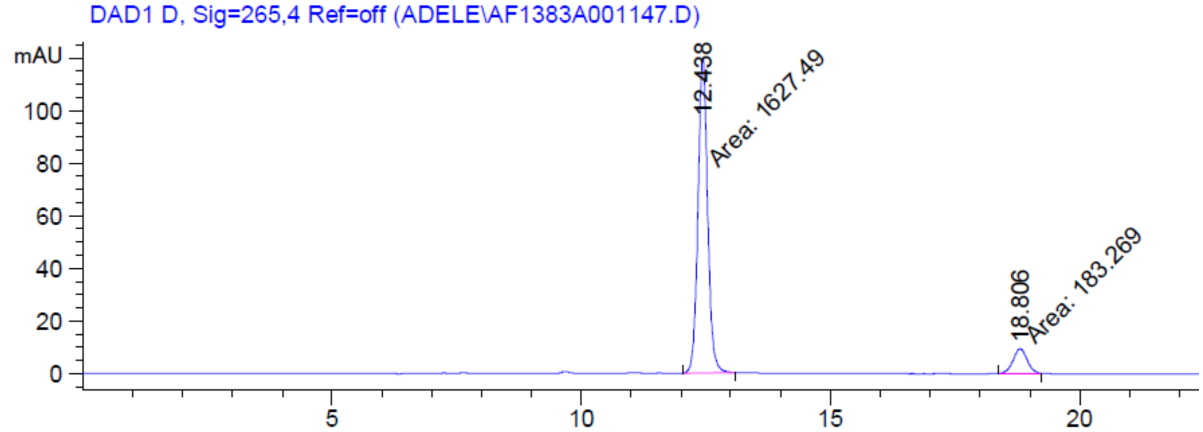

Peak #	RetTime [min]	Type	Width [min]	Area [mAU*s]	Height [mAU]	Area %
1	12.438	MM	0.2257	1627.48987	120.15942	89.8789
2	18.806	MM	0.3272	183.26920	9.33633	10.1211

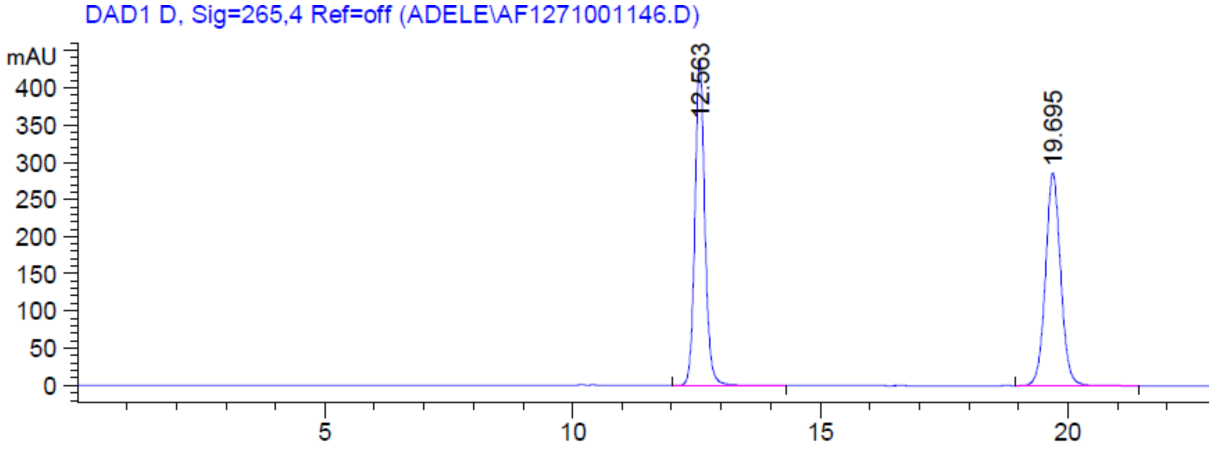

Peak #	RetTime [min]	Type	Width [min]	Area [mAU*s]	Height [mAU]	Area %
1	12.563	BB	0.2119	6088.07373	438.68225	49.9894
2	19.695	BB	0.3267	6090.65918	285.72601	50.0106

Methyl 3-cyclohexyl-3-oxopropanoate (A) and methyl (*Z*)-3-cyclohexyl-3-hydroxyacrylate (B).

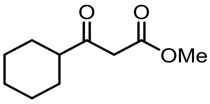

A

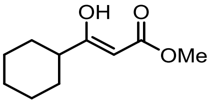

B

To a solution of diisopropylamine (6.18 mL, 44.2 mmol) in THF (30 mL) at -78 °C was added *n*-BuLi (1M in hexane, 44.2 mL, 44.2 mmol) dropwise. The reaction was stirred for 30 minutes. Methyl acetate was added slowly (3.50 mL, 44.17 mmol) at -78 °C and the reaction was stirred for 30 minutes. Cyclohexanecarbonyl chloride (1.99 mL, 14.70 mmol) was added and the reaction was warmed to room temperature over ~ 30 minutes. The reaction was stirred for a further 2 hours. To the reaction 1M HCl (50 mL) was added. The organic phase was extracted with EtOAc (3 × 100 mL). The organic extracts were combined, dried (Na_2SO_4) and concentrated *in vacuo*. The residue was purified by FCC (hexane:EtOAc 20:1) to afford the title compound (2.51 g, 93%, 1:0.2 mixture of keto and enol tautomers) as a pale yellow oil.

^{1}H NMR (400 MHz, $CDCl_3$): *Signals for both tautomers:* 12.02 (s, 0.2H, **B**-OH), 4.94 (s, 0.2H, **B**-C=CH), 3.72 (s, 2H, **A**-(CO)OCH_3), 3.70 (s, 0.6H, **B**-(CO)OCH_3), 3.48 (s, 2H, **A**-(CO)CH_2(CO)), 2.49-2.39 (m, 1H, **A**-CH(CO)), 2.13-2.02 (m, 0.2H, **B**-CH(CO)), 1.95-1.56 (m, 6H, **A** and **B** 2.5 × CH_2), 1.41-1.11 (m, 6H, **A** and **B** 2.5 × CH_2).

^{13}C NMR (100 MHz, $CDCl_3$): *Signals for the keto tautomer:* 205.7 (CH(CO)), 167.9 ((CO)OCH_3), 52.3 ((CO)OCH_3), 50.9 (CH(CO)), 47.0 ((CO)CH_2(CO)), 28.2, 25.7, 25.4 (3 × CH_2).

The spectroscopic properties of this compound were consistent with the data in the literature.[22]

(*E*)-1-Cyclohexyl-4-methyl-6-phenylhex-4-en-1-one

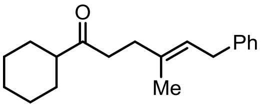

General Procedure C: Methyl 3-cyclohexyl-3-oxopropanoate (0.41 g, 2.23 mmol) and (*E*)-(4-bromo-3-methylbut-2-en-yl)benzene[13] (0.55 g, 2.45 mmol) were employed. FCC (hexane:EtOAc 40:1 to 35:1) afforded the title compound (0.35 g, 58%) as a pale yellow oil.

^{1}H NMR (400 MHz, $CDCl_3$): 7.30-7.12 (m, 5H, ArH), 5.33 (t, J = 7.5 Hz, 1H, C=CH), 3.34 (d, J = 7.5 Hz, 2H, C=CHCH_2Ar), 2.55 (t, J = 8.0 Hz, 2H, Ar(CO)CH_2), 2.37-2.24 (m, 3H, Ar(CO)CH_2CH_2 and CH(CO)), 1.85-1.60 (m, 8H, CH_3 and 2.5 × CH_2), 1.38-1.11 (m, 5H, 2.5 × CH_2).

^{13}C NMR (100 MHz, $CDCl_3$): 213.7 (C=O), 141.5 (ArC), 135.1 (C=CH), 128.3 (2 signals), 125.7 (3 × ArCH), 123.3 (C=CH), 50.9 (CH(CO)), 39.2 (Ar(CO)CH_2), 34.2 (C=CHCH_2Ar), 33.3 (Ar(CO)CH_2CH_2), 28.5, 25.8, 25.7 (3 × CH_2), 16.4 (CH_3).
FTIR: 2928, 1707, 1450 cm^{-1}.
MS: (ESI$^+$) Found $[M+H]^+$: 271.2060, $C_{19}H_{27}O$ requires 271.2056.

(*E*)-1-Cyclohexyl-4-methyl-6-phenylhex-4-en-1-one *O*-perfluorobenzoyl oxime 2p

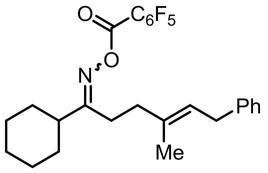

General Procedure D: Part A: (*E*)-1-cyclohexyl-4-methyl-6-phenylhex-4-en-1-one (0.35 g, 1.30 mmol) was used. The reaction was heated at 75 °C for 2 hours to afford the corresponding oxime (0.34 g, 92%) as a colorless solid. Part B: The corresponding oxime (0.34 g, 1.19 mmol) was employed and the reaction was stirred for 2 hours. FCC (hexane: EtOAc 30:1) afforded the title compound (0.55 g, 96%, 1:0.1 mixture of oxime isomers) as a colorless solid.
^{1}H NMR (400 MHz, $CDCl_3$): *Data for the major isomer:* 7.33-7.11 (m, 5H, ArH), 5.35 (t, J = 7.5 Hz, 1H, C=CH), 3.33 (d, J = 7.5 Hz, 2H, C=CHCH_2Ar), 2.52-2.45 (m, 2H, Ar(CN)CH_2), 2.44-2.33 (m, 1H, CH(CN)), 2.27-2.20 (m, 2H, Ar(CN)CH_2CH_2), 1.89-1.65 (m, 8H, CH_3 and 2.5 × CH_2), 1.48-1.11 (m, 5H, 2.5 × CH_2). *Characteristic signals for the minor isomer:* 5.43-5.38 (m, 0.1H, C=CH), 3.37-3.35 (m, 0.2H, C=CHCH_2Ar).
^{13}C NMR (100 MHz, $CDCl_3$): *Signals for the major isomer:* 174.8 (C=N), 141.1 (ArC), 134.4 (C=CH), 128.4, 128.2, 125.9 (3 × ArCH), 124.5 (C=CH), 44.1 (CH(CN)), 35.9 (Ar(CN)CH_2CH_2), 34.2 (C=CHCH_2Ar), 30.0 (CH_2), 29.1 (Ar(CN)CH_2), 25.9, 25.7 (2 × CH_2), 15.9 (CH_3).
^{19}F NMR (376 MHz, $CDCl_3$): *Data for the major isomer:*-137.3 (2F), -147.9 (1F), -159.9 (2F). *Data for the minor isomer:* -137.4 (0.2F), -148.0 (0.1F), -159.9 (0.2F).
FTIR: 1760, 1523, 1496, 1324, 1194 cm^{-1}.
MS: (ESI$^+$) Found $[M+Na]^+$: 502.1770, $C_{26}H_{26}F_5NNaO_2$ requires 502.1776.

(*R*,*E*)-5-Cyclohexyl-2-methyl-2-styryl-3,4-dihydro-2*H*-pyrrole 3p

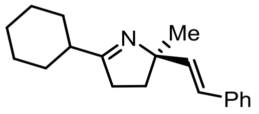

General Procedure E: Oxime ester **2p** (50.0 mg, 0.104 mmol) was employed and the reaction was heated at 120 °C for 1.3 hours. FCC (toluene:EtOAc 30:1 to 8:1) afforded the title compound (19.5 mg, 71%, 93:7 e.r.) as a pale yellow oil.
$[\alpha]_D^{22}$ -14.8 (c = 0.61, $CHCl_3$).

^{1}H NMR (400 MHz, $CDCl_3$): 7.37-7.32 (m, 2H, ArH), 7.30-7.24 (m, 2H, ArH), 7.18 (tt, J = 7.5 and 2.0 Hz, 1H, ArH), 6.40 (d, J = 16.0 Hz, 1H, HC=CH), 6.34 (d, J = 16.0 Hz, 1H, HC=CH), 2.62-2.46 (m, 2H, Ar(CN)CH_2), 2.42-2.32 (m, 1H, CH(CN)), 2.01-1.64 (m, 7H, Ar(CN)CH_2CH_2 and 2.5 × CH_2), 1.44-1.16m (m, 8H, CH_3 and 2.5 × CH_2).

^{13}C NMR (100 MHz, $CDCl_3$): 180.6 (C=N), 137.5 (ArC), 136.7 (HC=CHAr), 128.4, 127.0, 126.3 (3 × ArCH), 126.1 (HC=CHAr), 75.2 (quaternary C), 42.7 (CH(CN)), 35.7 (Ar(CN)CH_2CH_2), 34.6 (Ar(CN)CH_2), 30.6 (CH_2), 27.6 (CH_3), 26.1, 26.0 (2 × CH_2).

FTIR: 2925, 1633, 1448 cm^{-1}.

MS: (ESI^+) Found $[M+H]^+$: 268.2057, $C_{19}H_{26}N$ requires 268.2060.

The enantiomeric purity of this compound was determined by chiral HPLC (Chiralpak IA, isocratic hexane-*i*-PrOH (99:1, 0.5 mL/min, 20 °C) against a racemic standard prepared under similar conditions using $P(3,5\text{-}(CF_3)_2C_6H_3)_3$ (15 mol%) as ligand; t_R (major) = 14.2 min and t_R (minor) = 16.0 min. The absolute structure of this molecule was confirmed by Vibrational Circular Dichroism (VCD).

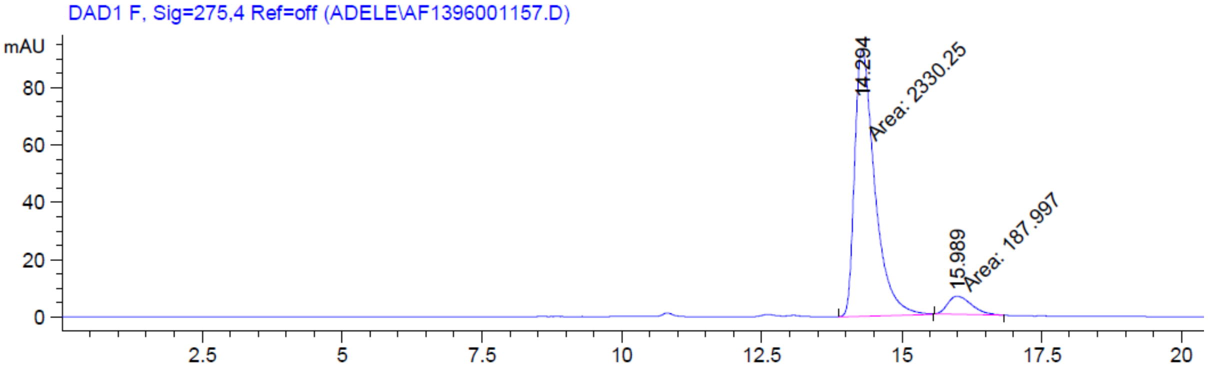

Peak #	RetTime [min]	Type	Width [min]	Area [mAU*s]	Height [mAU]	Area %
1	14.294	MM	0.4170	2330.25171	93.13181	92.5346
2	15.989	MM	0.4942	187.99690	6.33954	7.4654

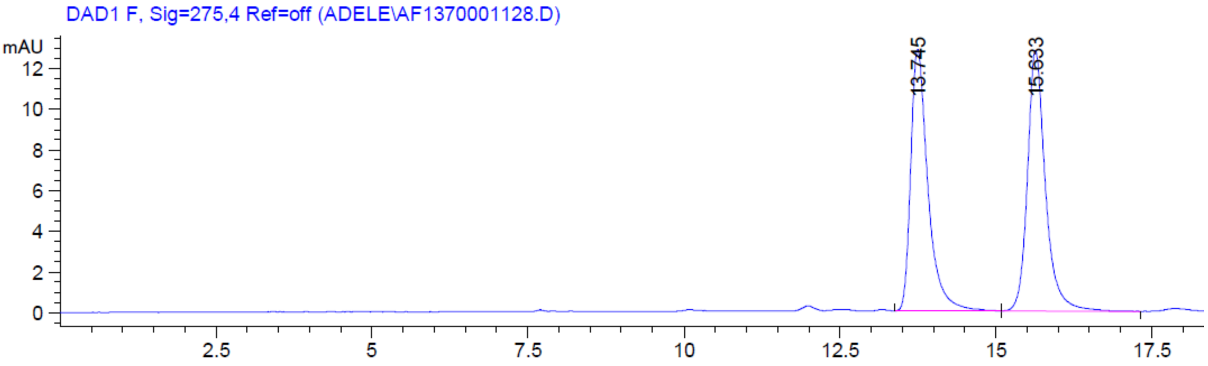

Peak #	RetTime [min]	Type	Width [min]	Area [mAU*s]	Height [mAU]	Area %
1	13.745	BB	0.2859	537.31555	28.00338	49.2121
2	15.634	BB	0.2966	554.52039	27.81294	50.7879

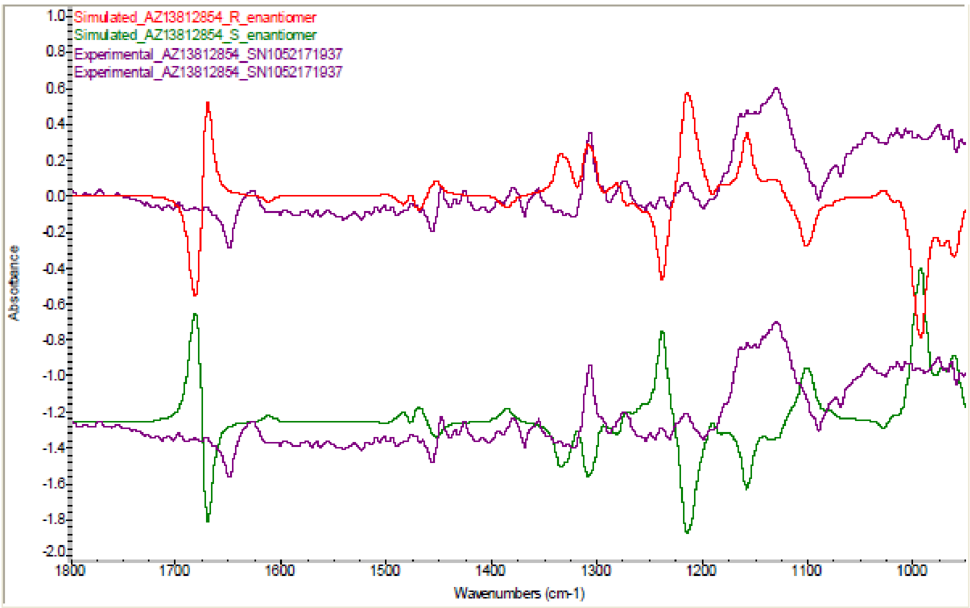

Comparison of experimental VCD spectrum of dihydropyrrole **3p** *(in duplicate, purple) and simulated spectra of the R (red) and S (green) enantiomers. The agreement between the simulated spectrum of the R enantiomer and the experimental spectrum is good, therefore dihydropyrrole* **3p** *has R configuration.*

Experimental: A 11.9 mg sample of dihydropyrrole **3p** was dissolved in $CDCl_3$ (110 µL). A VCD spectrum was acquired in 0.100 mm BaF_2 cells for 7 hours each in a BioTools ChiralIR instrument equipped with dual source and dual photoelastic modulator. The resolution was 4 cm^{-1}. The experimental VCD spectrum shows weak characteristic bands.

Computational Spectral Simulations: A Monte Carlo molecular mechanics search for low energy geometries was conducted for full structures of the two enantiomers, *R* and *S*. *MacroModel* within the *Maestro* graphical interface (Schrödinger Inc.) was used to generate starting coordinates for conformers. All conformers within 5 kcal/mole of the lowest energy conformer were used as starting points for density functional theory (DFT) minimizations within *Gaussian09*. Optimized structures, harmonic vibrational frequencies/intensities, VCD rotational strengths, and free energies at STP (including zero-point energies) were determined for each conformer. In these calculations, the functional B3LYP and the basis set 6-31G* were used. Simulations of infrared and VCD spectra for each conformation were generated using an in-house built program to fit Lorentzian line shapes (12 cm^{-1} line width) to the computed spectra thereby allowing direct comparisons between simulated and experimental spectra.

Results: The experimental spectrum was compared with simulated spectra of the two enantiomers based on DFT calculations starting with full structures. The comparison is presented above. The agreement between the simulated spectrum of the *R* enantiomer and the experimental spectrum is good. It is therefore concluded that dihydropyrrole **3p** has *R* configuration.

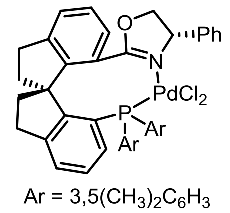

Dichloro[(*S*)-2-((*S*)-7'-(Bis(3,5-dimethylphenyl)phosphanyl)-2,2',3,3'-tetrahydro-1,1'-spirobi[inden]-7-yl)-4-phenyl-4,5-dihydrooxazole]palladium [(*S*$_a$,*S*)-L-2f]$PdCl_2$ 4

A solution of (COD)$PdCl_2$ (4.6 mg, 0.016 mmol) and (*S*$_a$,*S*)-**L-2f** (10.0 mg, 0.016 mmol) in CH_2Cl_2 (0.7 mL) was stirred at room temperature for 90 minutes, after which time it was carefully concentrated *in vacuo*. The resulting yellow residue was washed with pentane (2 mL) and dried under vacuum to give the title compound (10.0 mg, 78%) as a yellow solid. Crystals suitable for X-ray diffraction were grown from slow evaporation of $CDCl_3$ (0.3 mL) layered with hexane (0.5 mL) at 20 °C. δ_H (400 MHz, $CDCl_3$) 8.12 (1H, d, *J* = 7.5 Hz, ArC<u>H</u>), 7.63-7.53 (4H, m, ArC<u>H</u>), 7.50-7.37 (5H, m, ArC<u>H</u>), 7.34 (1H, d, *J* = 7.5 Hz, ArC<u>H</u>), 7.29-7.15 (4H, m, ArC<u>H</u>), 7.10 (1H, s, ArC<u>H</u>), 7.02 (1H, s, ArC<u>H</u>), 4.32 (1H, dd, *J* = 12.0 and 9.0 Hz, C$\underline{H}_2$CHPh), (1H, dd, *J* = 10.0 and 9.0 Hz, C$\underline{H}_2$CHPh), 3.61 (1H, dd, *J* = 12.0 and 10.0 Hz, CH_2C<u>H</u>Ph), 3.05-2.85 (2H, m, C<u>H</u>), 2.67-2.53 (1H, m, C<u>H</u>), 2.35-2.13 (13H, m, 4 × C$\underline{H_3}$ and C<u>H</u>), 2.10-1.90 (2H, m, C<u>H</u>), 1.69-1.58 (1H, m, C<u>H</u>), 0.83-0.69 (1H, m, C<u>H</u>); δ_P (162 MHz, $CDCl_3$) 28.6 (s). *The structure of this compound was determined unambiguously by X-ray crystallography.*

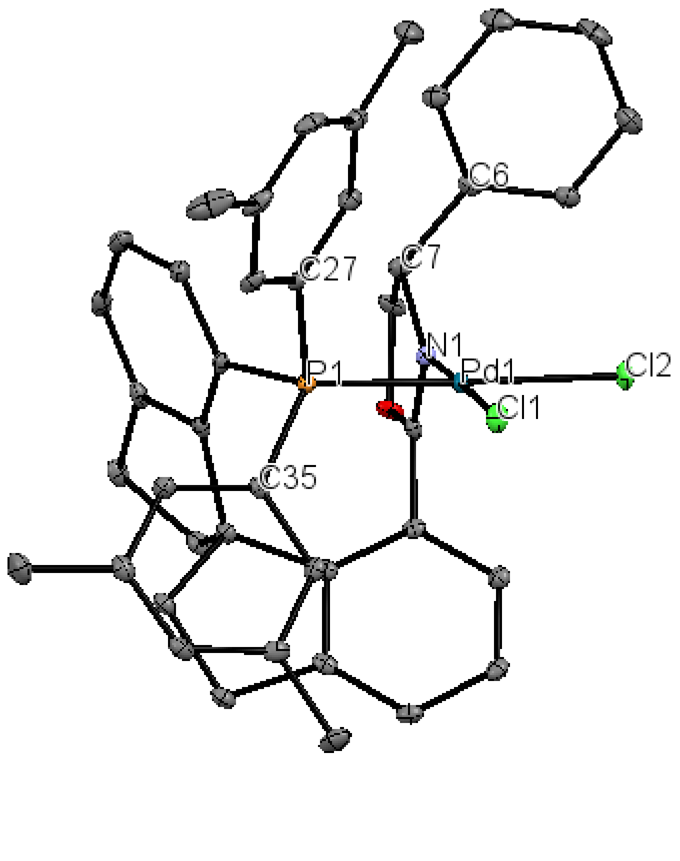

ORTEP view of [(S_a,S)-**L-2f**]$PdCl_2$ **4**, ellipsoids at 30%, hydrogen atoms and chloroform solvent in the lattice have been omitted for clarity; Crystal data for [(S_a,S)-**L-2f**]$PdCl_2$ **4**: $C_{42}H_{40}Cl_2NOPPd$, MW = 781.13, orthorhombic, space group $P2_12_12_1$, a = 13.7983(8) Å, b = 18.7174 (11) Å, c = 19.3028(11) Å, V = 4985.3(5) $Å^3$, α = 90°, β = 90°, γ = 90°, Z = 4, D_c = 1.520 g/cm^3, MoKα radiation, λ = 0.71073 Å, μ = 1.029 mm^{-1}, T = 100 K; crystal size 0.51×0.26×0.16 mm, Kappa Apex II, 44882 reflections collected, 11959 were unique, R_{int} = 0.0492; refinement method: on F^2, gave R = 0.0328 and R_w = 0.0636, GOF = 1.020 for 554 refined parameters.

(*S*)-3-Methyl-6-phenylhexan-3-amine 5

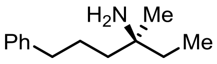

A pressure vessel, fitted with magnetic stirrer, was charged with 10% Pd/C (13.5 mg, 0.13 mmol), MeOH (6 mL), H_2O (1 mL) and (*R*)-**3a** (50 mg, 0.27 mmol). The vessel was pressurised with H_2 (6 atm) and vigorously stirred at room temperature for 4 days. The reaction vessel was depressurised and the mixture was filtered through a pad of celite, washing with EtOAc (25 mL). The filtrate was concentrated *in vacuo* to afford amine **5** (50.1 mg, 97%) as a colourless oil.

$[\alpha]_D^{23}$ -17.5 (c = 0.5, $CHCl_3$).

^{1}H NMR (400 MHz, $CDCl_3$): 7.29-7.23 (m, 2H, Ar<u>H</u>), 7.20-7.13 (m, 3H, Ar<u>H</u>), 6.67-5.91 (br s, 2H, N<u>H2</u>), 2.62 (t, *J* = 7.5 Hz, 2H, ArC<u>H2</u>), 1.79-1.60 (m, 6H, $ArCH_2$C<u>H2</u>C<u>H2</u> and C<u>H2</u>CH_3), 1.28 (s, 3H, CC<u>H3</u>), 0.92 (t, *J* = 7.5 Hz, 3H, CH_2C<u>H3</u>).

^{13}C NMR (100 MHz, $CDCl_3$): 141.7 (Ar<u>C</u>), 128.3 (2 signals), 125.9 (3 × Ar<u>C</u>H), 56.9 (<u>C</u>NH_2), 37.9 ($ArCH_2CH_2$<u>C</u>H_2), 35.8 (Ar<u>C</u>H_2), 31.5 (<u>C</u>H_2CH_3), 25.0 ($ArCH_2$<u>C</u>H_2), 24.0 (C<u>C</u>H_3), 7.9 (CH_2<u>C</u>H_3).

FTIR 2886, 1601, 1508, 1460, 1383 cm^{-1}.

MS (CI^+) Found $[M+H]^+$: 192.1758, $C_{13}H_{22}N$ requires 192.1752.

*The enantiospecificity of the process was determined to be >95% by analysis of the Mosher's amide derivatives of compound **5**.*

(2*S,5*R**)-2-isopropyl-5-(naphthalen-2-yl)-2-vinylpyrrolidine 6**

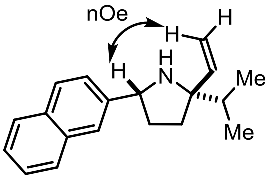

To a solution of imine ***rac*-3j** (60 mg, 0.23 mmol) in dry toluene (1.5 mL), stirring under nitrogen at -78 °C, was added a solution of DIBAL-H (1.43 mmol, 6.2 eq) dropwise. The reaction mixture was stirred at -78 °C for 6 hours then warmed to 0 °C and quenched with saturated Rochelle's Salt solution (5 mL). The phases were separated and the aqueous layer was extracted with Et_2O (5 x 10 mL). The organic layers united were washed with water (30 mL), dried over $MgSO_4$ and the solvents removed *in*

vacuo. The crude product was purified by FCC (5 to 10% EtOAc in hexane) and preparative TLC (2 runs in 3% EtOAc in hexane) to yield the title compound **6** (43 mg, 0.16 mmol, 70%, 5:1 d.r.).

^{1}H NMR (400 MHz, $CDCl_3$): *Data for the major diastereomer:* 7.88 – 7.80 (m, 4H), 7.56 (dd, *J* = 8.5, 1.7 Hz, 1H), 7.52 – 7.37 (m, 2H), 5.89 (dd, *J* = 17.4, 10.7 Hz, 1H), 5.21 (dd, *J* = 10.7, 1.3 Hz, 1H), 5.15 (dd, *J* = 17.4, 1.3 Hz, 1H), 4.40 (t, *J* = 7.5 Hz, 1H), 2.35 – 2.15 (m, 1H), 1.99 – 1.71 (m, 5H), 0.99 (d, *J* = 6.9 Hz, 6H). *A signal corresponding to NH was not observed. Characteristic signals for the minor diastereomer:* 5.99 (dd, *J* = 17.1, 10.6 Hz, 1H), 5.42 (dd, *J* = 17.2, 2.0 Hz, 1H), 4.47 (dd, *J* = 10.0, 5.7 Hz, 1H).

^{13}C NMR (100 MHz, $CDCl_3$): *Signals for the major diastereomer only:* 143.4, 142.5, 133.4, 132.7, 128.0, 127.8, 127.6, 125.8, 125.4, 125.3, 124.8, 112.3, 69.5, 60.6, 38.3, 34.0, 33.9, 18.5, 18.0.

FTIR: 2960, 2878 cm^{-1}.

MS: (ESI^+) Found $[M+H]^+$: 266.1906, $C_{19}H_{24}N$ requires 266.1903.

The relative stereochemistry of this compound was assigned on the basis of nOe experiments as indicated on the compound structure.

X-ray Crystallography Experimental Details

X-ray diffraction data for **3b** was collected at 100K on a Bruker Microstar rotating anode diffractometer using Cu-K_α (λ = 1.54178 Å), while data for **4** were collected at 100 K on a Bruker APEX II diffractometer using Mo-K_α radiation (λ = 0.71073 Å). Data collections were performed using a CCD area detector from a single crystal mounted on a glass fibre. Intensities were integrated[23] in SAINT and absorption corrections were based on equivalent reflections using SADABS.[24] The structures were solved using Superflip[25] and all of the structures were refined against F^2 in SHELXL[26] using Olex2.[27] All of the non-hydrogen atoms were refined anisotropically. While all of the hydrogen atoms were located geometrically and refined using a riding model.

References

1. Zhao, Q.-Y.; Shi, M. *Tetrahedron* **2011**, *67*, 3724-3732.
2. Zhao, Q.-Y.; Yuan, Z.-L.; Shi, M. *Adv. Synth. Catal.* **2011**, *353*, 637-643.
3. Li, Q.; Wan, P.; Wang, S.; Zhuang, Y.; Li, L.; Zhou, Y.; He, Y.; Cao, R.; Qiu, L.; Zhou, Z. *Applied Catalysis A: General* **2013**, *458*, 201-206.
4. Vonlanthen, D.; Rotzler, J.; Neuburger, M.; Mayor, M. *Eur. J. Org. Chem.* **2010**, 120-133.
5. Andersh, B.; Murphy, D. L.; Olson, R. J. *Synth. Commun.* **2000**, *30*, 2091-2098.
6. Lan, K.; Shan, Z.; Fan, S. *Tetrahedron Lett.* **2006**, *47*, 4343-4345.
7. Li, Z.; Liang, X.; Wu, F.; Wan, B. *Tetrahedron: Asymmetry* **2004**, *15*, 665-669.
8. Li, Z.; Liang, X.; Wan, B.; Wu, F. *Synthesis* **2004**, 2805-2808.
9. Birman, V. B.; Rheingold, A. L.; Lam, K.-C. *Tetrahedron: Asymmetry* **1999**, *10*, 125-131.
10. Xie, J.-H.; Wang, L.-X.; Fu, Y.; Zhu, S.-F.; Fan, B.-M.; Duan, H.-F.; Zhou, Q.-L. *J. Am. Chem. Soc.* **2003**, *125*, 4404-4405.
11. Li, S.; Zhu, S.-F.; Zhang, C.-M.; Song, S.; Zhou, Q.-L. *J. Am. Chem. Soc.* **2008**, *130*, 8584-8585.
12. Faulkner, A.; Scott, J. S.; Bower, J. F. *Chem. Commun.* **2013**, *49*, 1521-1523.
13. Schleicher, K. D.; Jamison, T. F. *Beilstein J. Org.Chem.* **2013**, *9*, 1533-1550.
14. Mitsudome, T.; Takahashi, Y.; Mizugaki, T.; Jitsukawa, K.; Kaneda, K. *Angew. Chem., Int. Ed.* **2014**, *53*, 8348-8351.
15. Hutchby, M.; Houlden, C. E.; Haddow, M. F.; Tyler, S. N. G.; Lloyd-Jones, G. C.; Booker-Milburn, K. I. *Angew. Chem., Int. Ed.* **2012**, *51*, 548-551.
16. Chowdhury, A. D.; Ray, R.; Lahiri, G. K. *Chem. Commun.* **2012**, *48*, 5497-5499.
17. Tueting, D. R.; Echavarren, A. M.; Stille, J. K. *Tetrahedron* **1989**, *45*, 979-992.
18. Duboudin, J. G.; Jousseaume, B.; Saux, A. *J. Organomet. Chem.* **1979**, *168*, 1-11.
19. Navarre, L.; Darses, S.; Genet, J.-P. *Chem.Commun.* **2004**, 1108-1109.
20. Bausch, C. C.; Patman, R. L.; Breit, B.; Krische, M. J. *Angew. Chem., Int. Ed.* **2011**, *50*, 5687-5690.
21. Borowiecki, P.; Bretner, M. *Tetrahedron: Asymmetry* **2013**, *24*, 925-936.
22. Bonnett, S. A.; Whicher, J. R.; Papireddy, K.; Florova, G.; Smith, J. L.; Reynolds, K. A. *Chem. Biol.* **2013**, *20*, 772-783.
23. Bruker-AXS SAINT V8.27B Madison, Wisconsin.
24. Sheldrick, G. M. SADABS V2012/1, University of Göttingen, Germany.
25. Palatinus, L.; Chapuis, G. *J. Appl. Cryst.* **2007**, *40*, 786-790; Palatinus, L.; van der Lee, A. *J. Appl. Cryst.* **2008**, *41*, 975-984; Palatinus, L.; Prathapa, S. J.; van Smaalen, S. *J. Appl. Cryst.* **2012**, *45*, 575-580.
26. Sheldrick, G. M. *Acta Cryst. A* **2008**, *64*, 112.
27. Dolomanov, O. V.; Bourhis, L. J.; Gildea, R. J.; Howard, J. A. K.; Puschmann, H. *J. Appl. Cryst.* **2009,** *42*, 339-341.

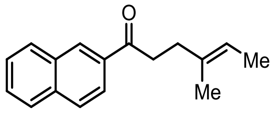
O
Me
Me

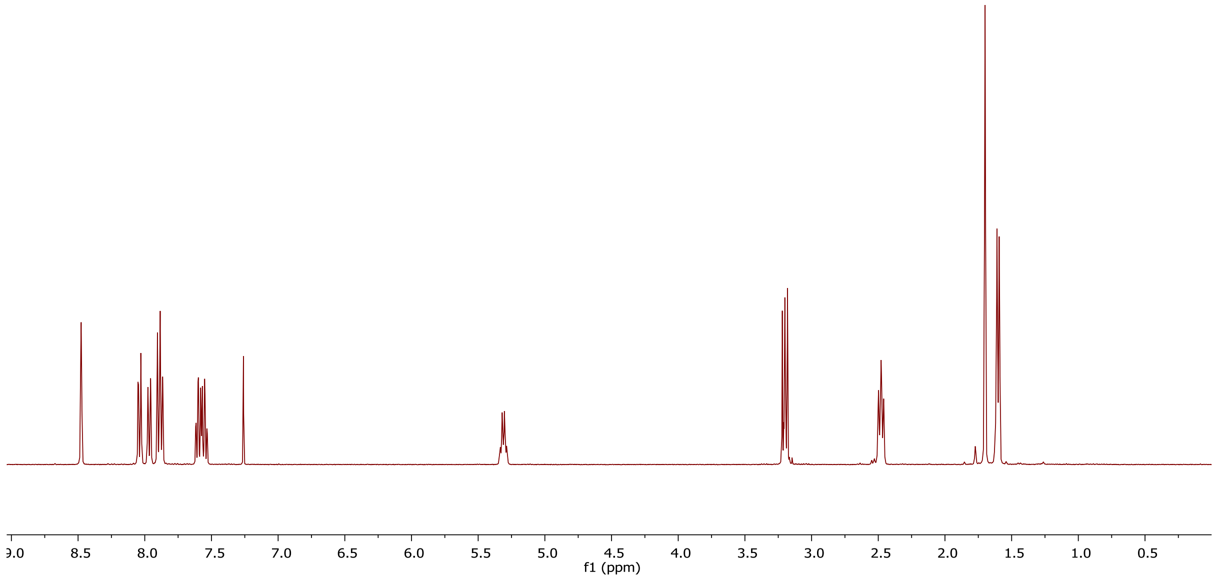
9.0
8.5
8.0
7.5
7.0
6.5
6.0
5.5
5.0
4.5
4.0
3.5
3.0
2.5
2.0
1.5
1.0
0.5
f1 (ppm)

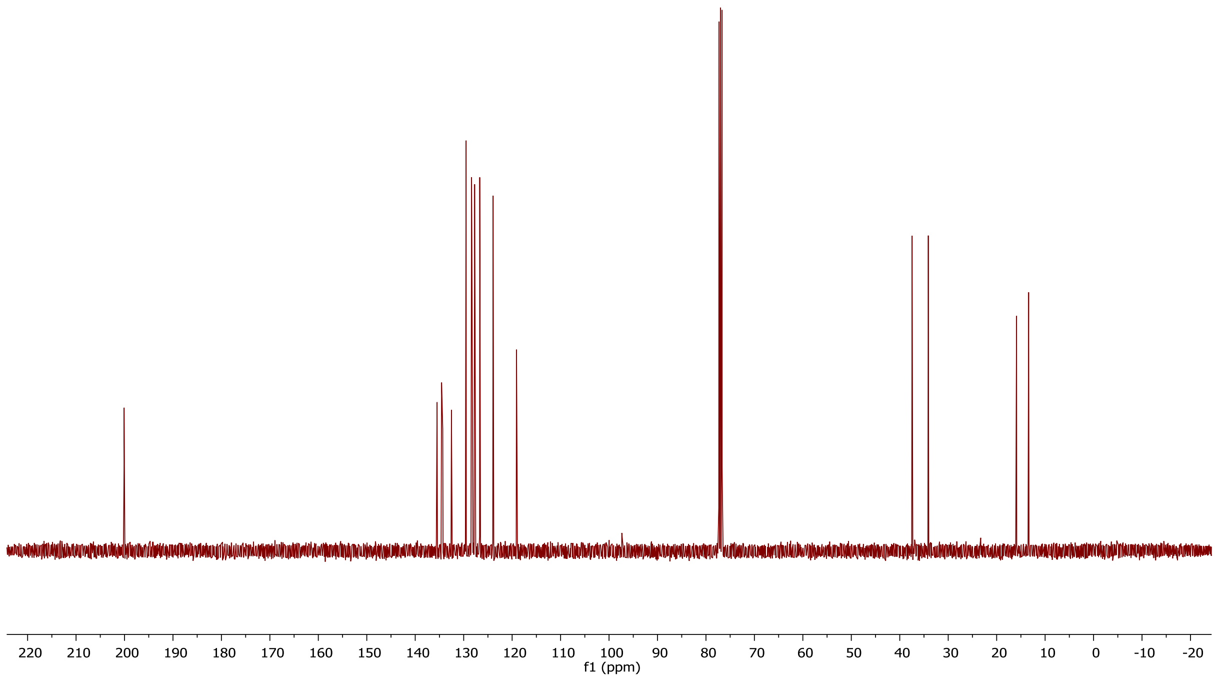
220
210
200
190
180
170
160
150
140
130
120
110
100
90
80
70
60
50
40
30
20
10
0
-10
-20
f1 (ppm)

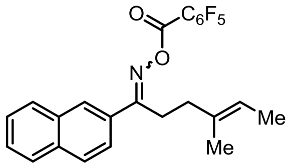
O
C6F5
N
O
Me
Me

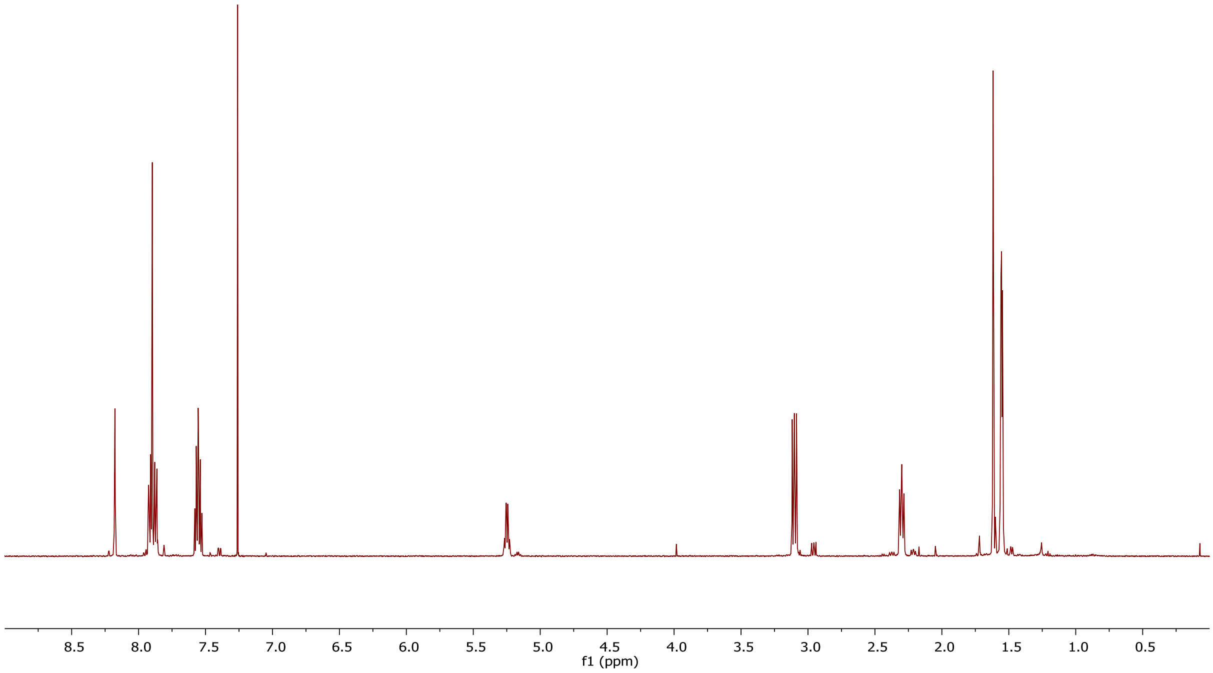
8.5
8.0
7.5
7.0
6.5
6.0
5.5
5.0
4.5
4.0
3.5
3.0
2.5
2.0
1.5
1.0
0.5
f1 (ppm)

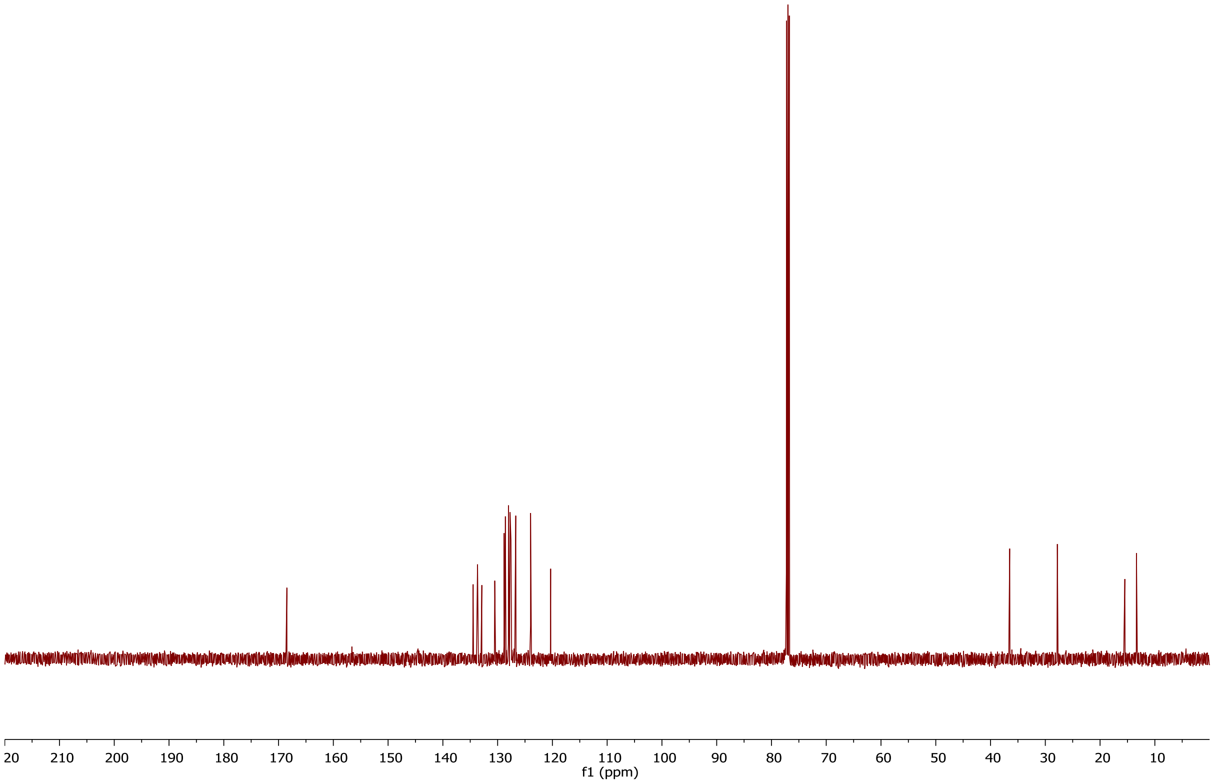
210
200
190
180
170
160
150
140
130
120
110
100
90
80
70
60
50
40
30
20
10
f1 (ppm)

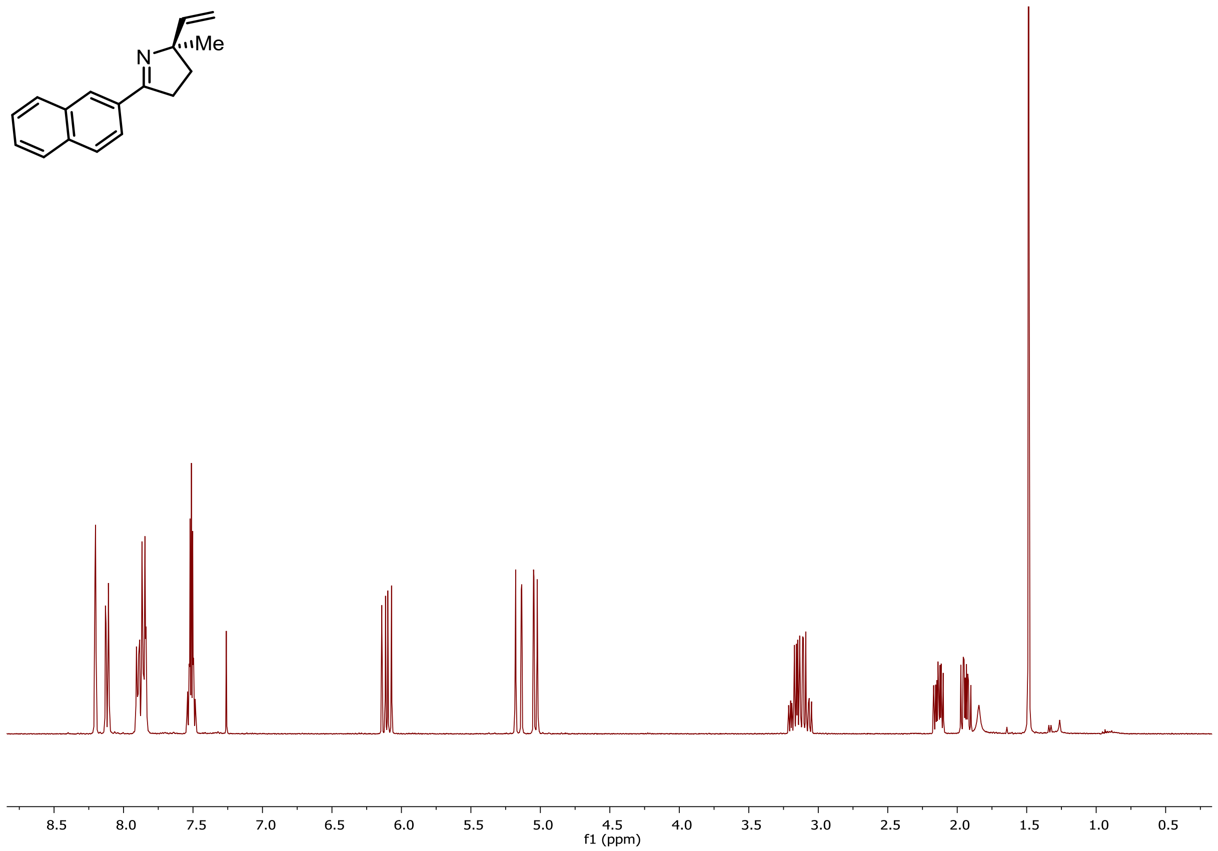
N
Me
8.5 8.0 7.5 7.0 6.5 6.0 5.5 5.0 4.5 4.0 3.5 3.0 2.5 2.0 1.5 1.0 0.5
f1 (ppm)

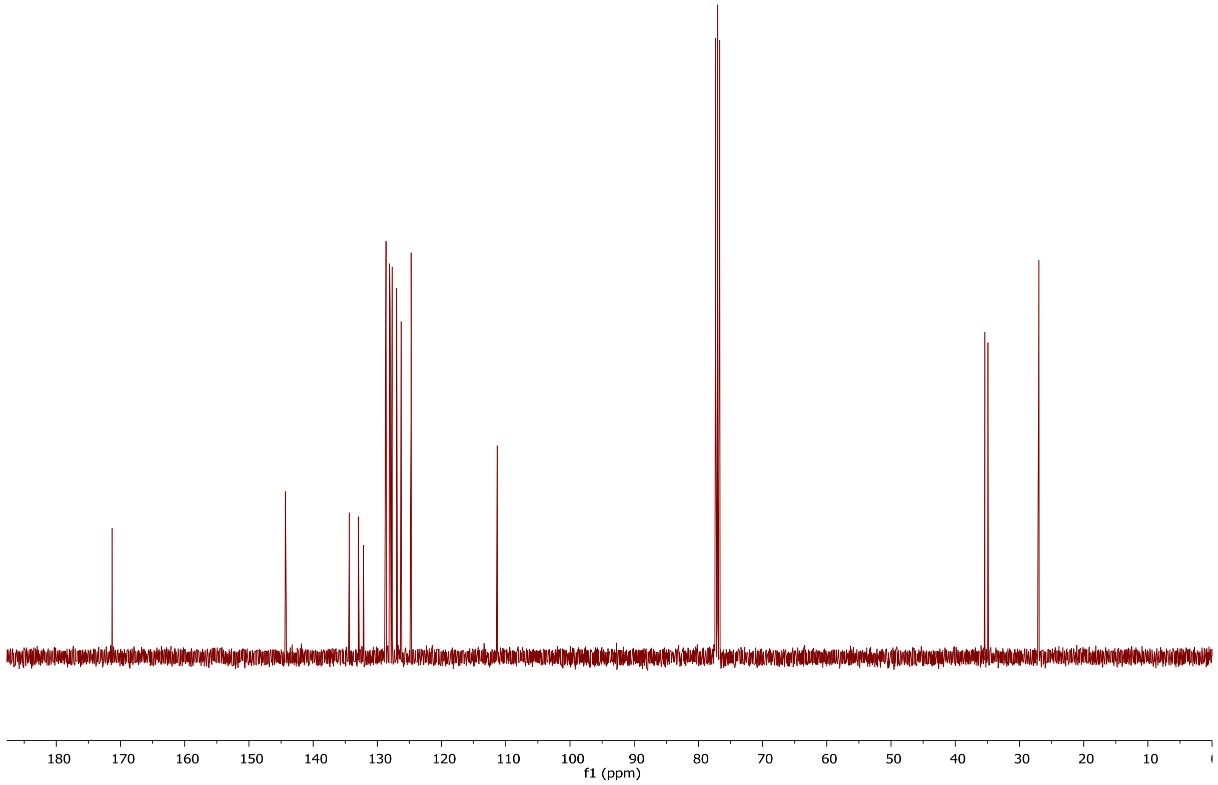
180 170 160 150 140 130 120 110 100 90 80 70 60 50 40 30 20 10
f1 (ppm)

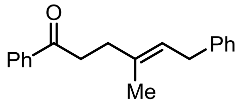

O
Ph
Ph
Me

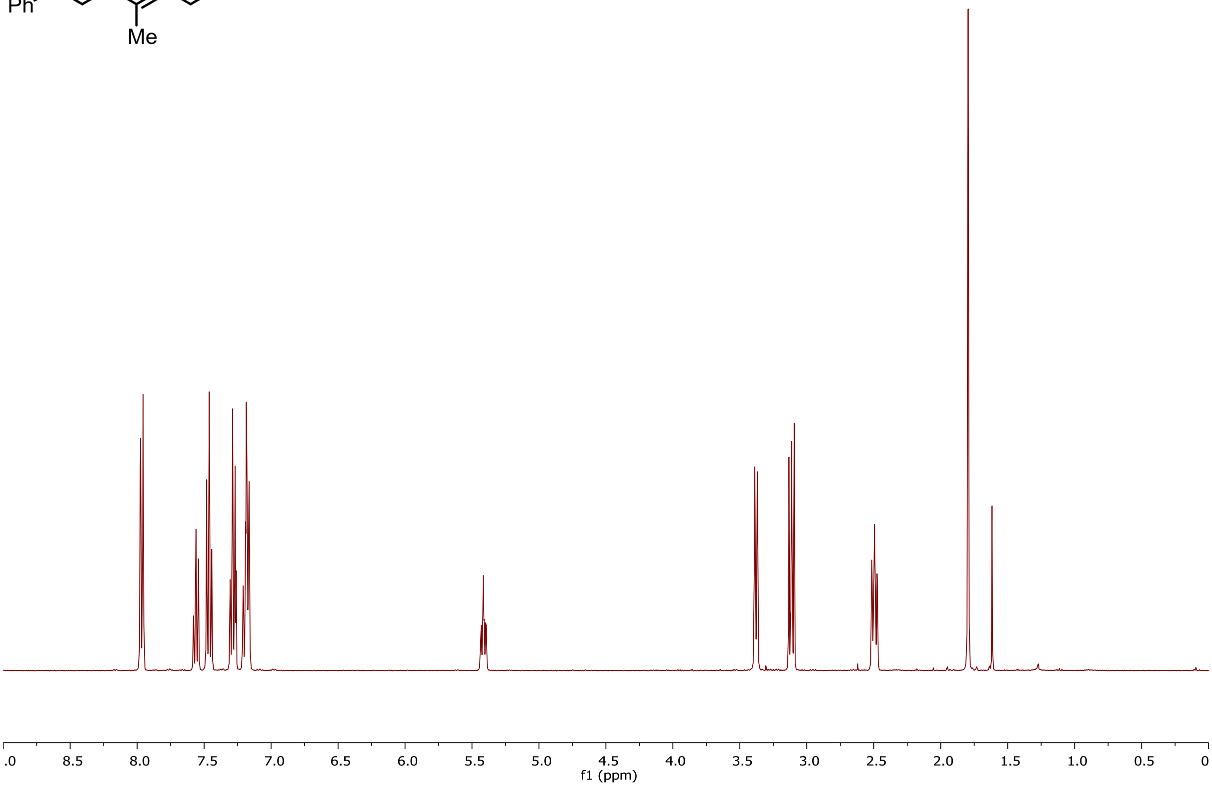

8.5
8.0
7.5
7.0
6.5
6.0
5.5
5.0
4.5
4.0
3.5
3.0
2.5
2.0
1.5
1.0
0.5
f1 (ppm)

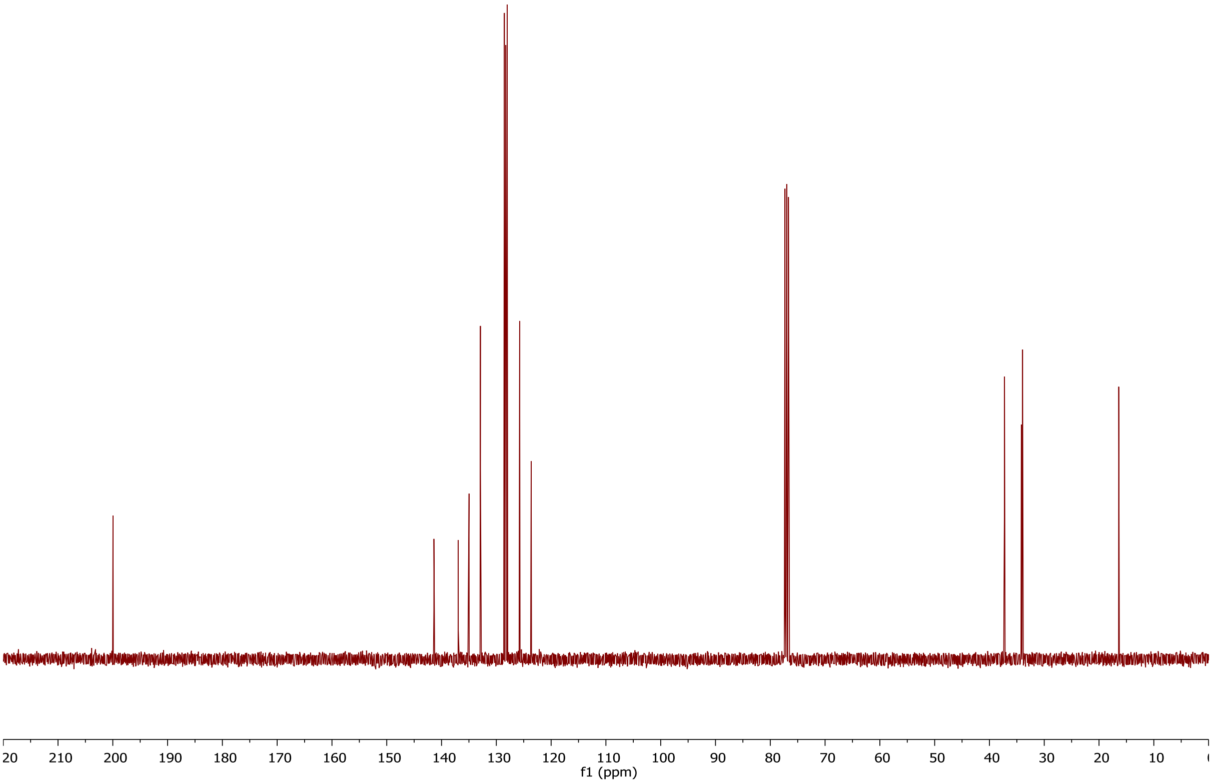

210
200
190
180
170
160
150
140
130
120
110
100
90
80
70
60
50
40
30
20
10
f1 (ppm)

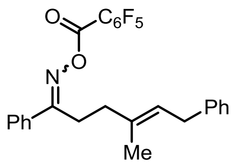

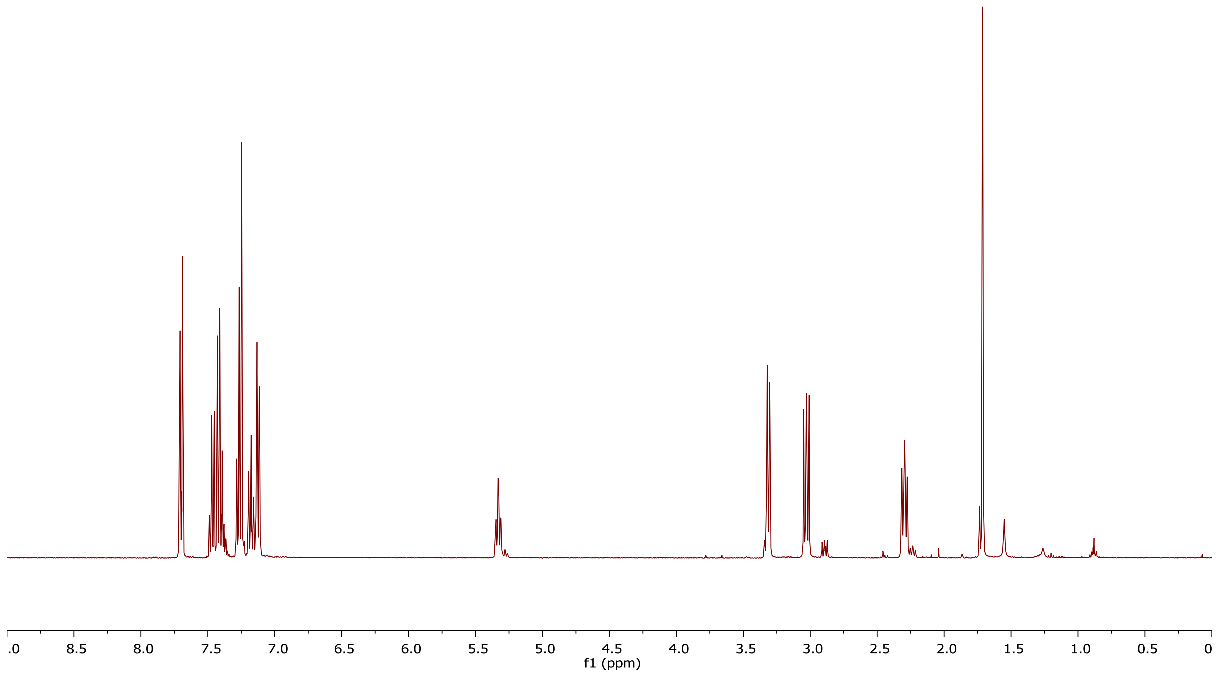

jb/nr23289_nr01344
single pulse decoupled gated NOE

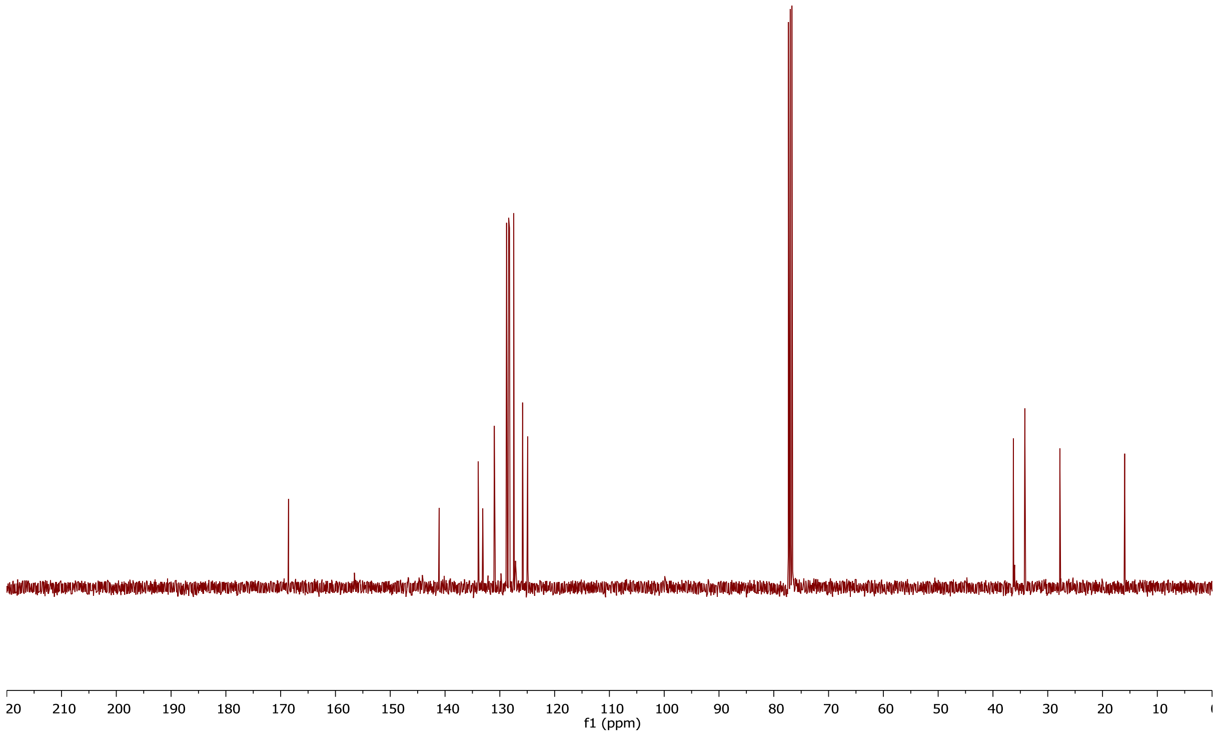

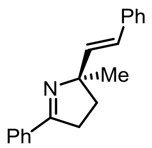
Ph
Me
N
Ph

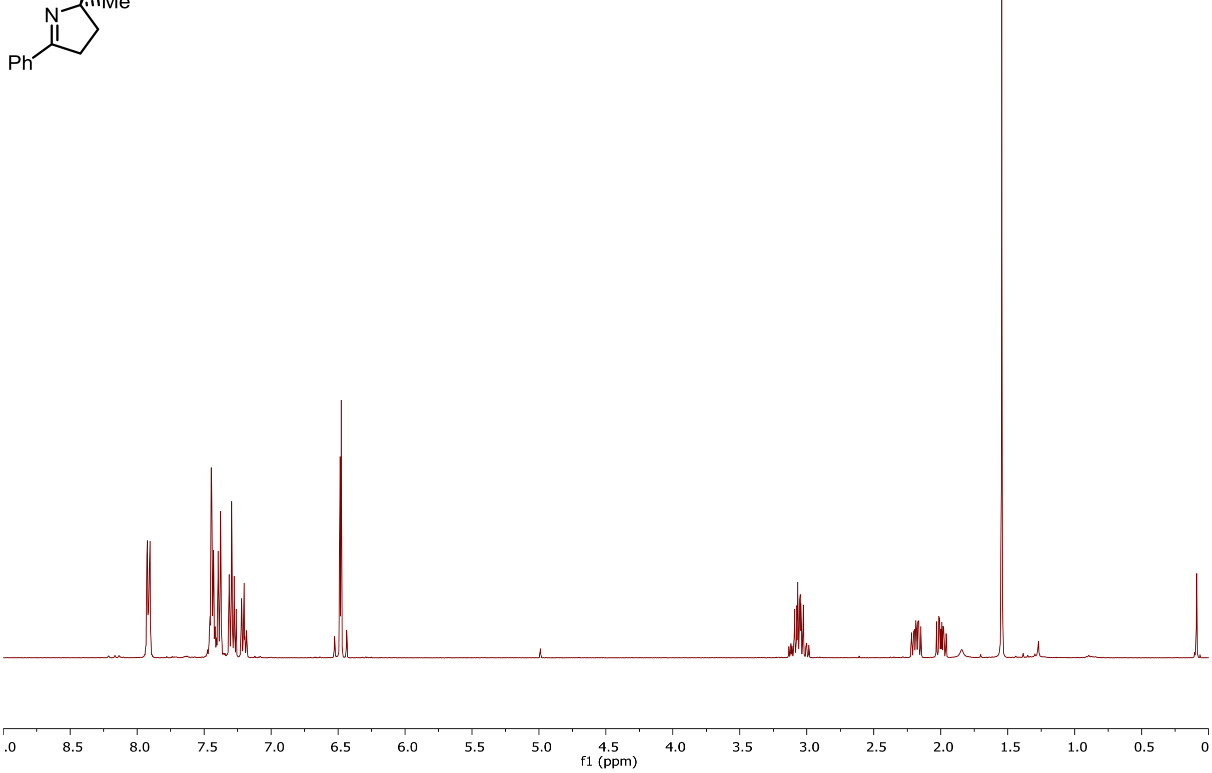
.0 8.5 8.0 7.5 7.0 6.5 6.0 5.5 5.0 4.5 4.0 3.5 3.0 2.5 2.0 1.5 1.0 0.5 0
f1 (ppm)

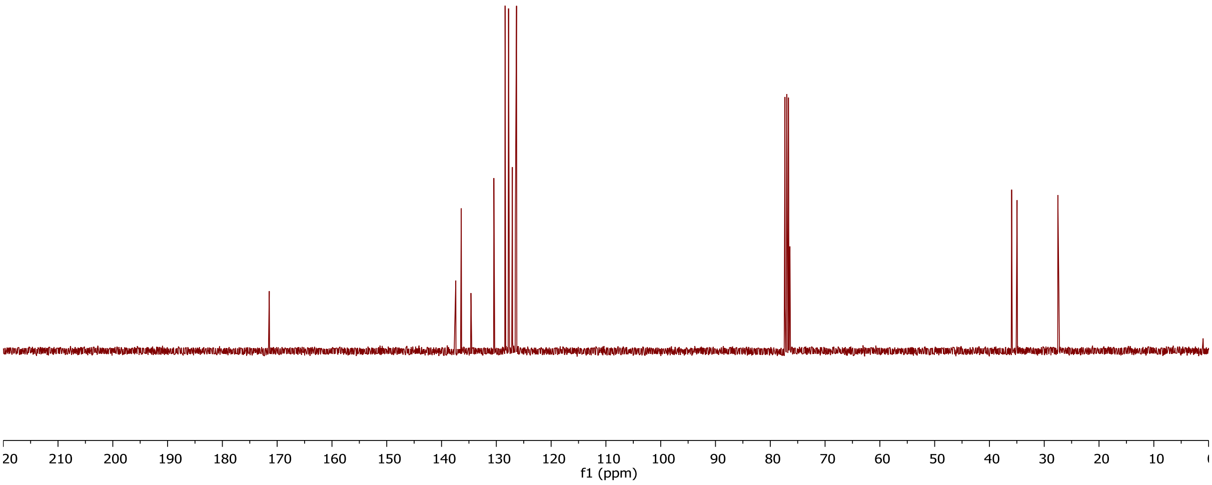
20 210 200 190 180 170 160 150 140 130 120 110 100 90 80 70 60 50 40 30 20 10
f1 (ppm)

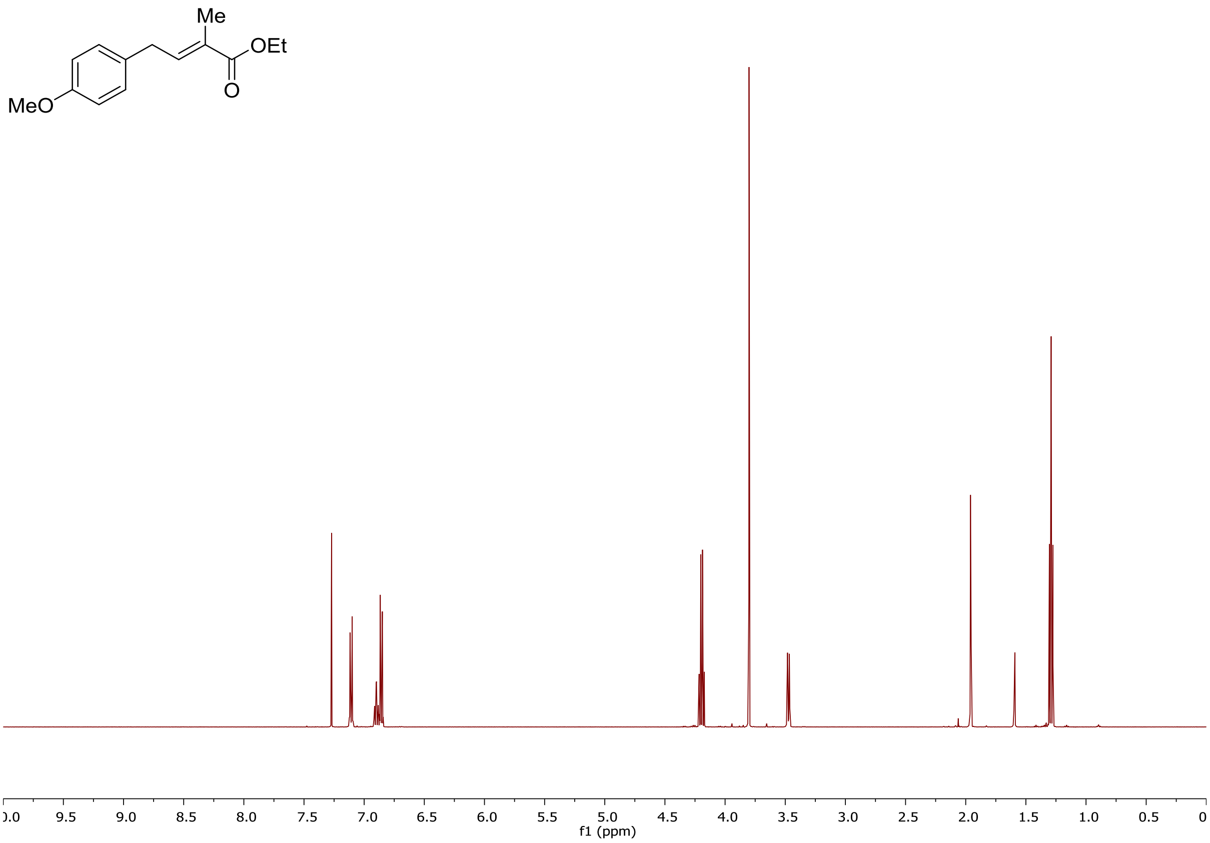
Me
OEt
O
MeO
f1 (ppm)

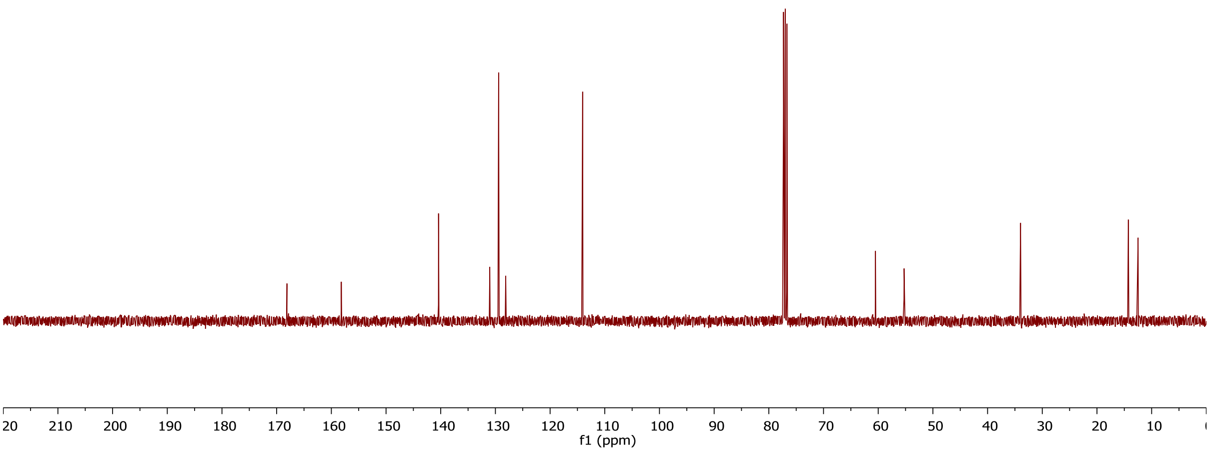
f1 (ppm)

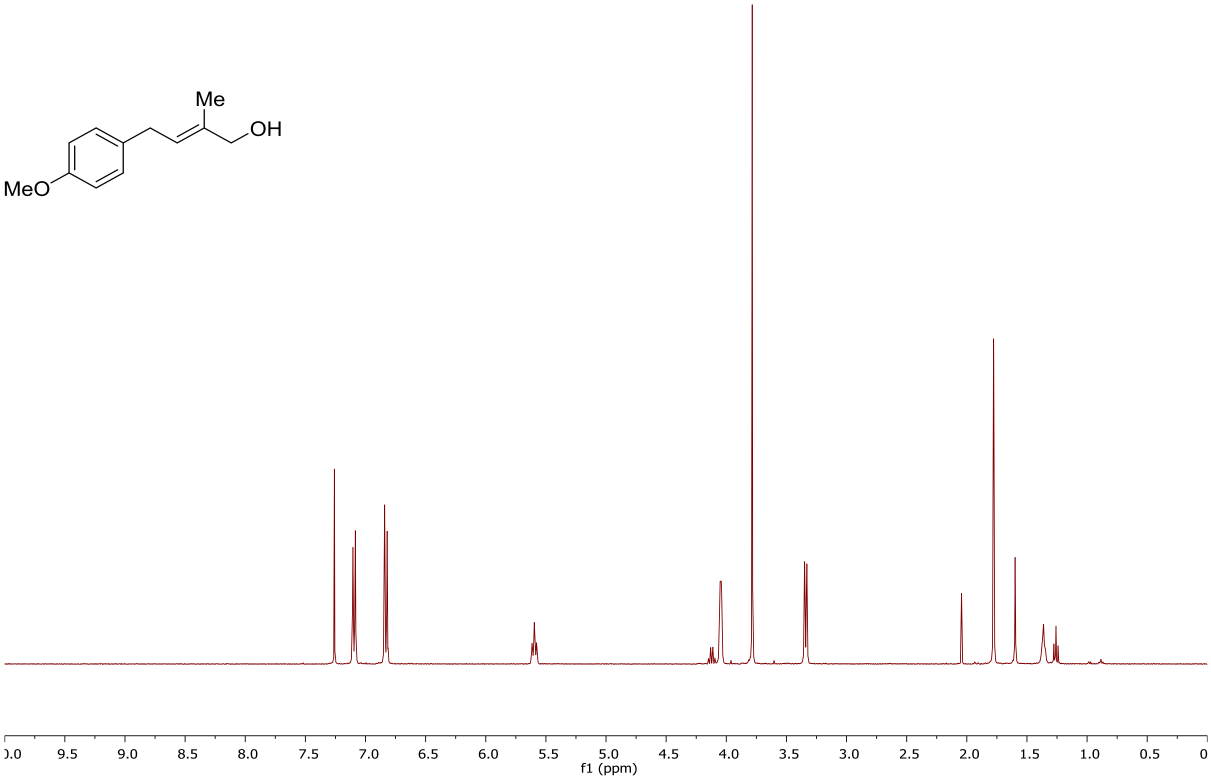
Me
OH
MeO
f1 (ppm)

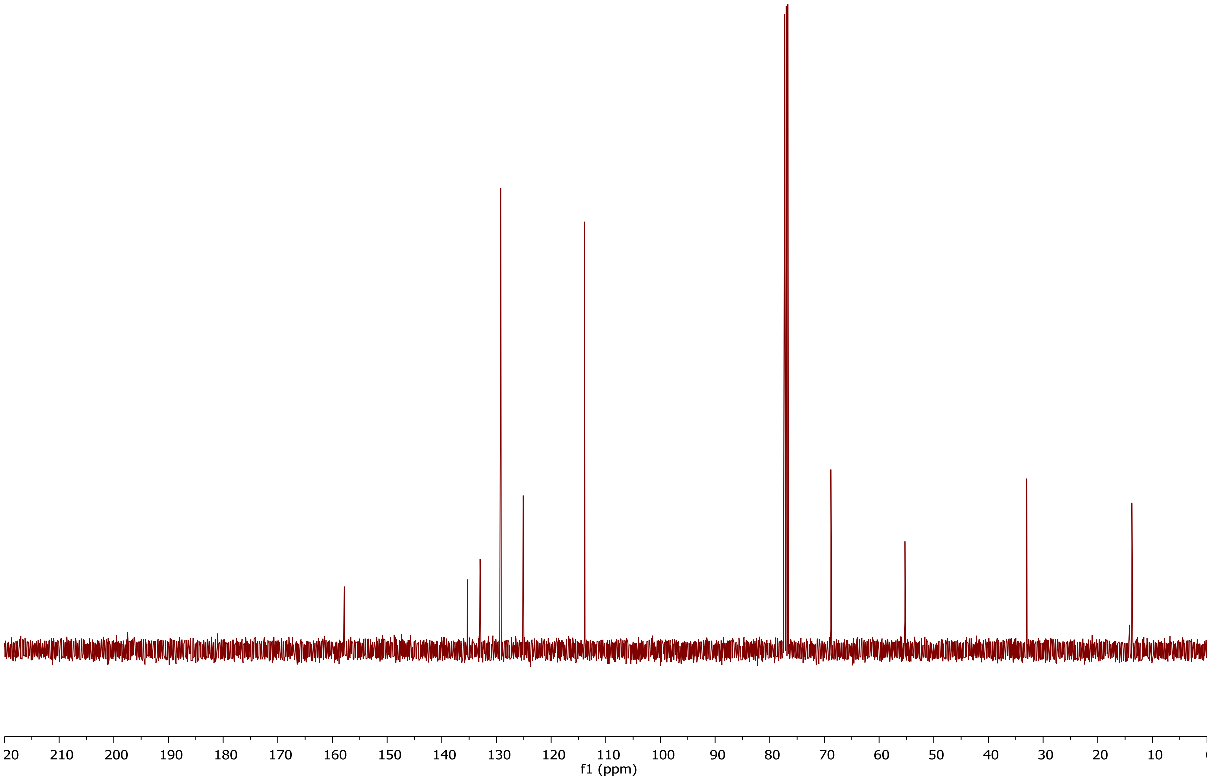
f1 (ppm)

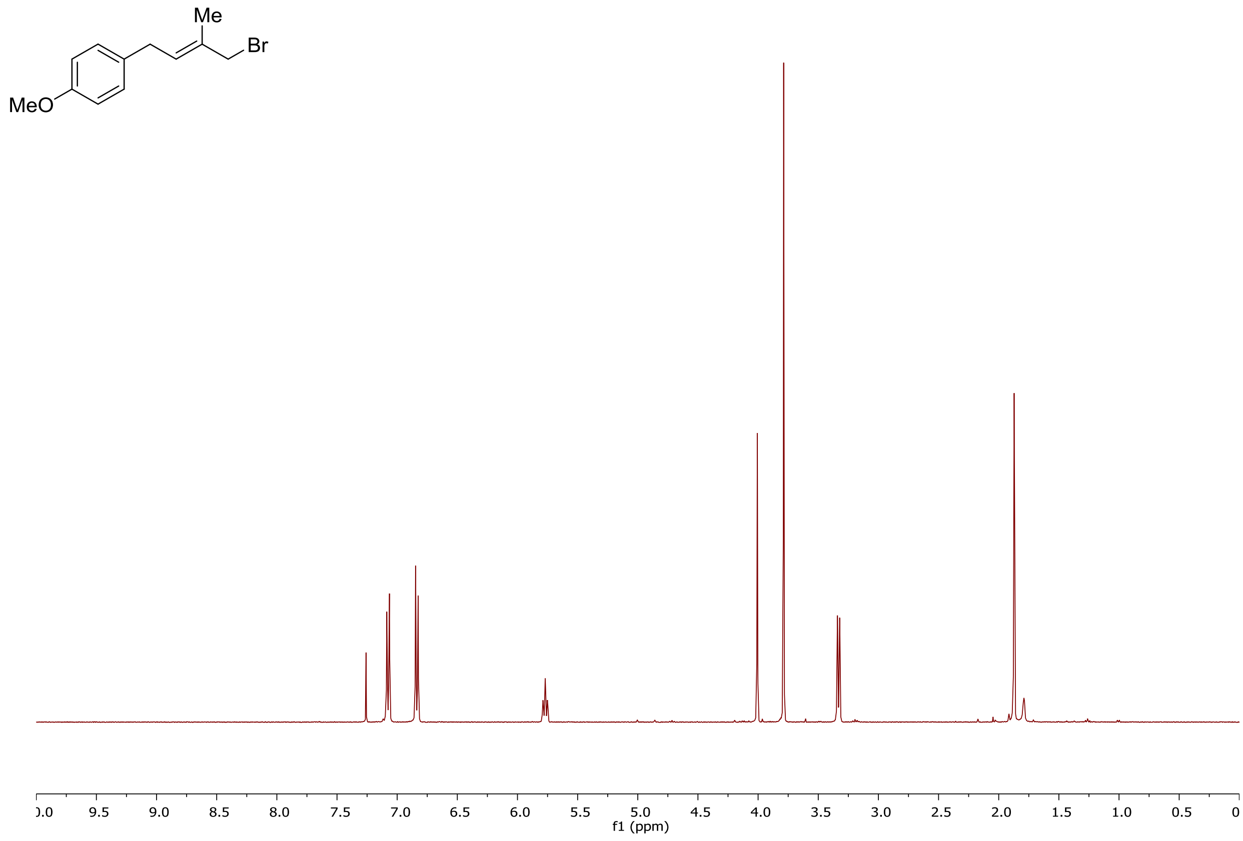
Me
Br
MeO
).0 9.5 9.0 8.5 8.0 7.5 7.0 6.5 6.0 5.5 5.0 4.5 4.0 3.5 3.0 2.5 2.0 1.5 1.0 0.5 0
f1 (ppm)

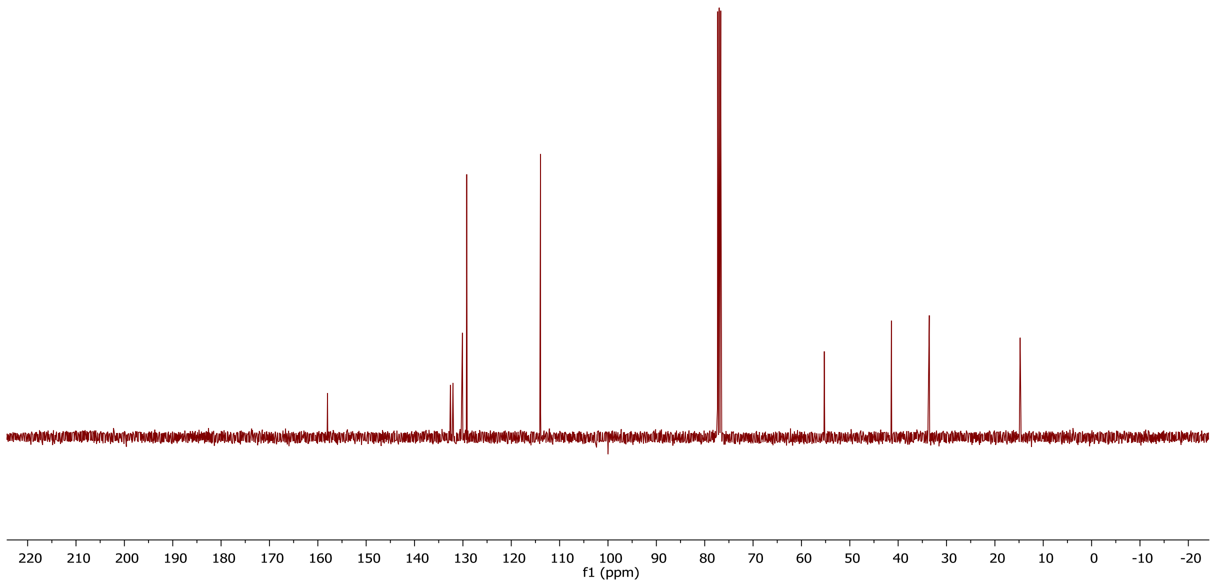
220 210 200 190 180 170 160 150 140 130 120 110 100 90 80 70 60 50 40 30 20 10 0 -10 -20
f1 (ppm)

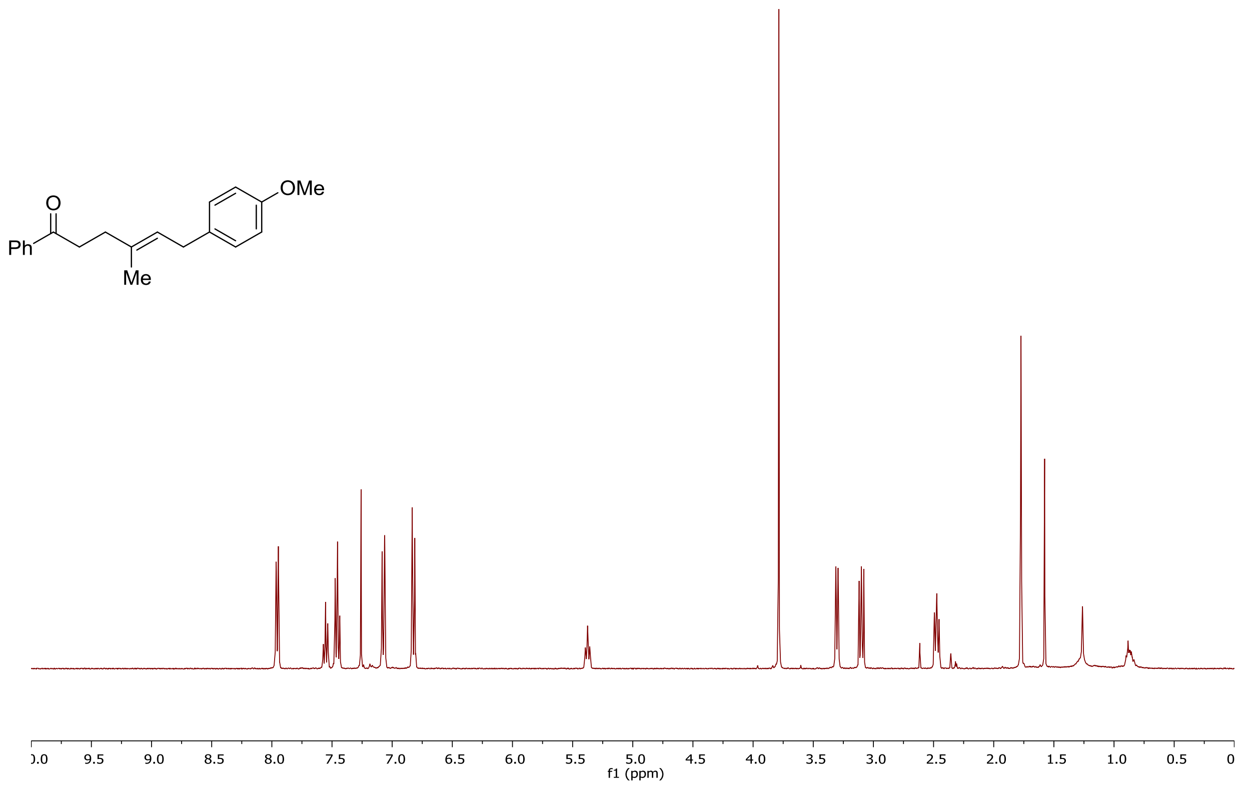
O
Ph
OMe
Me
0.0 9.5 9.0 8.5 8.0 7.5 7.0 6.5 6.0 5.5 5.0 4.5 4.0 3.5 3.0 2.5 2.0 1.5 1.0 0.5 0
f1 (ppm)

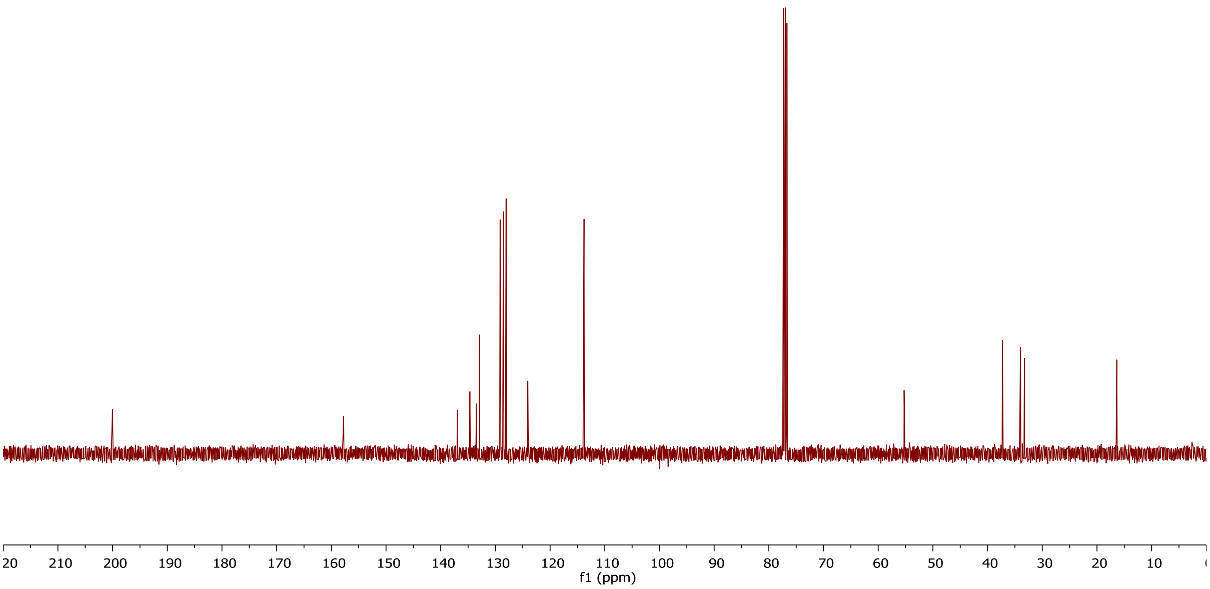
20 210 200 190 180 170 160 150 140 130 120 110 100 90 80 70 60 50 40 30 20 10
f1 (ppm)

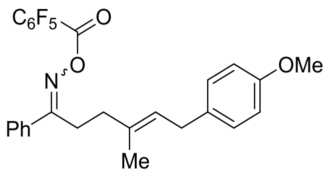
C6F5
O
O
N
OMe
Ph
Me

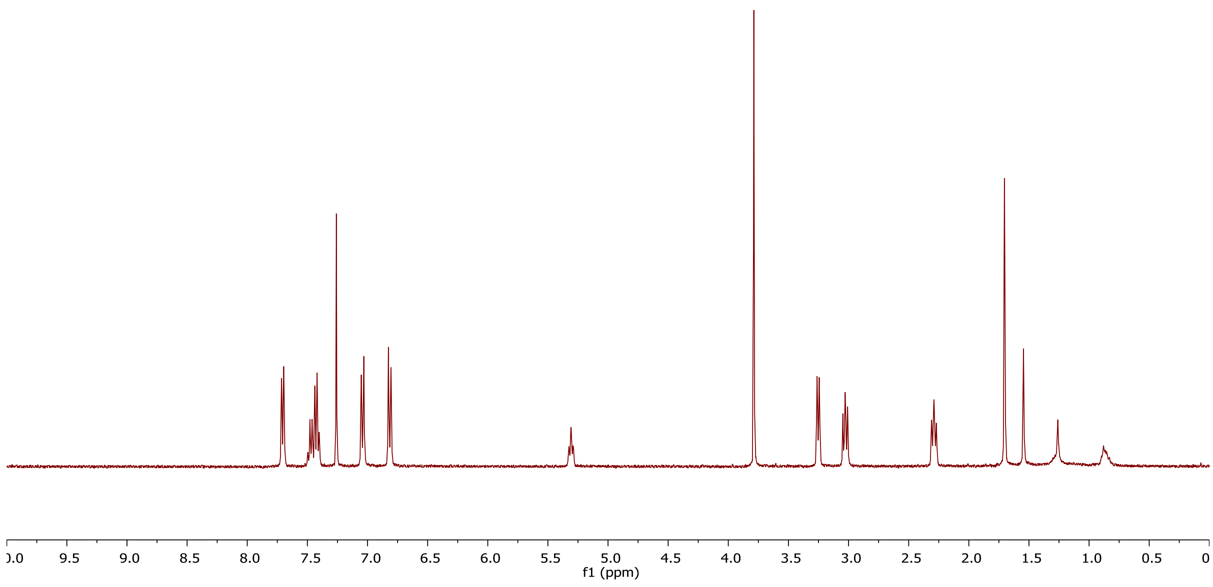
9.5 9.0 8.5 8.0 7.5 7.0 6.5 6.0 5.5 5.0 4.5 4.0 3.5 3.0 2.5 2.0 1.5 1.0 0.5 0
f1 (ppm)

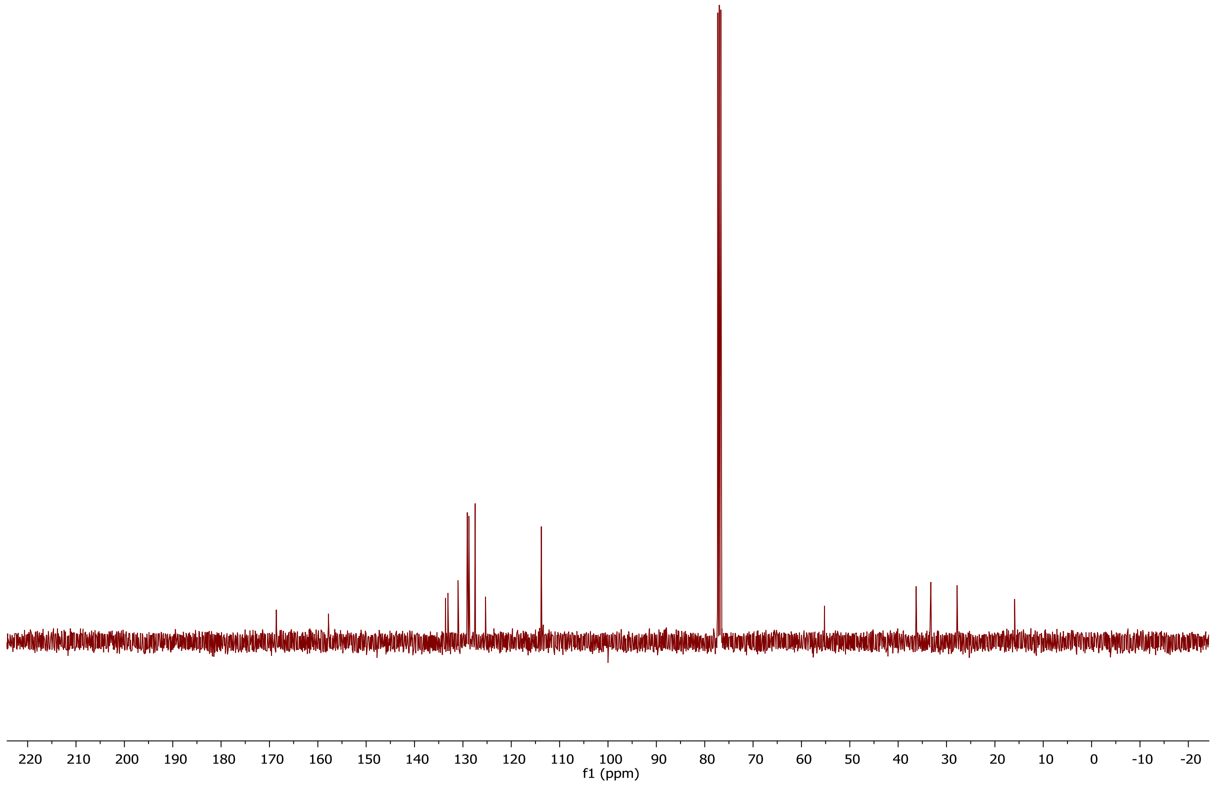
220 210 200 190 180 170 160 150 140 130 120 110 100 90 80 70 60 50 40 30 20 10 0 -10 -20
f1 (ppm)

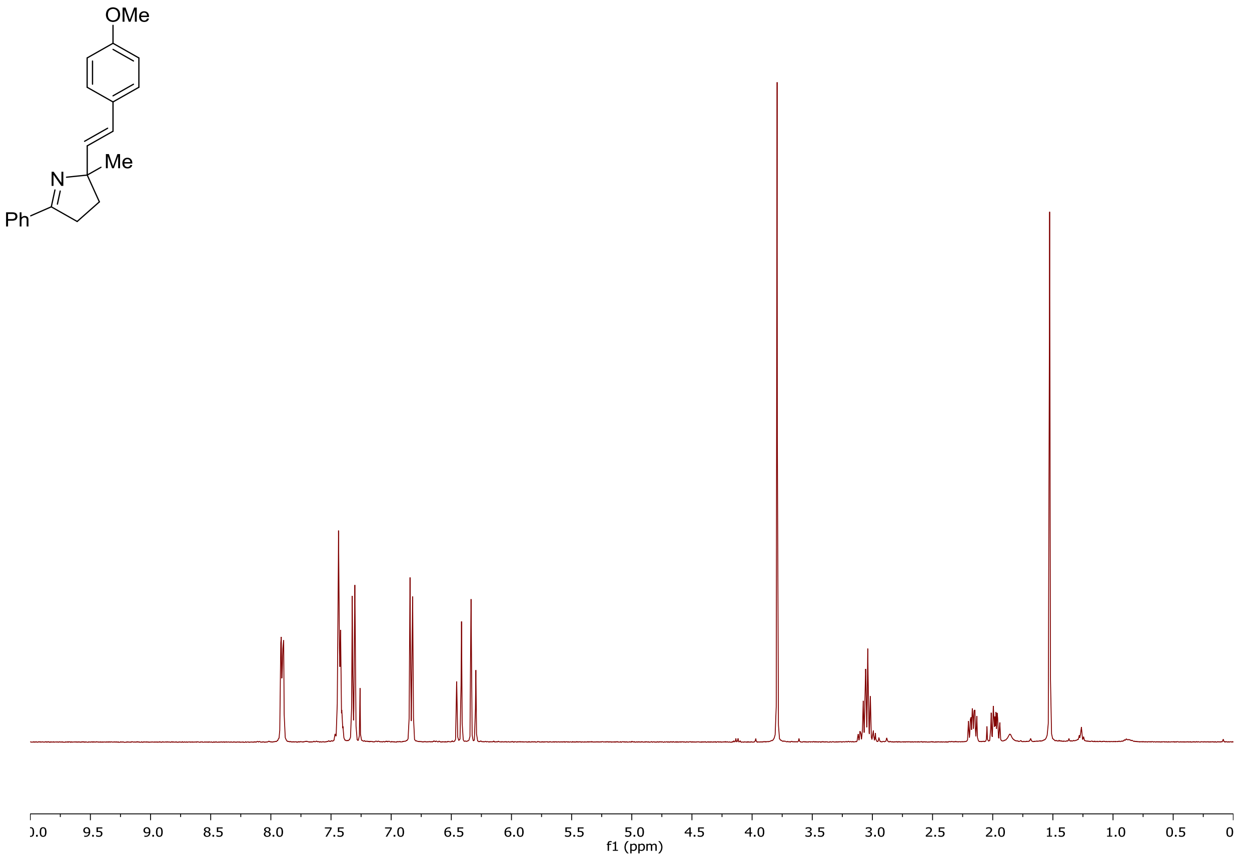
OMe
Me
N
Ph
f1 (ppm)

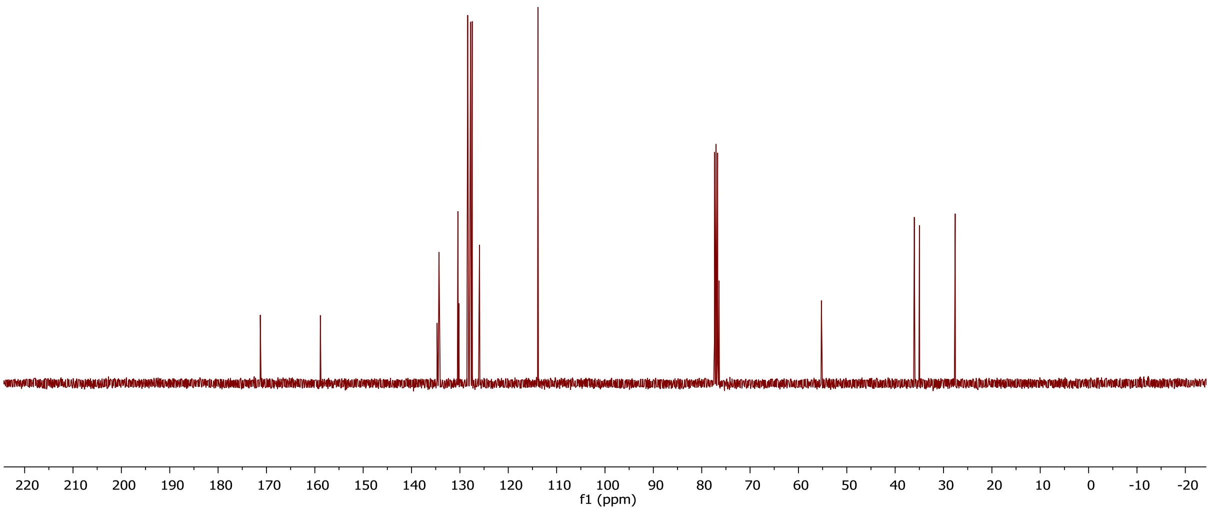
f1 (ppm)

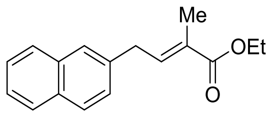
Me
OEt
O

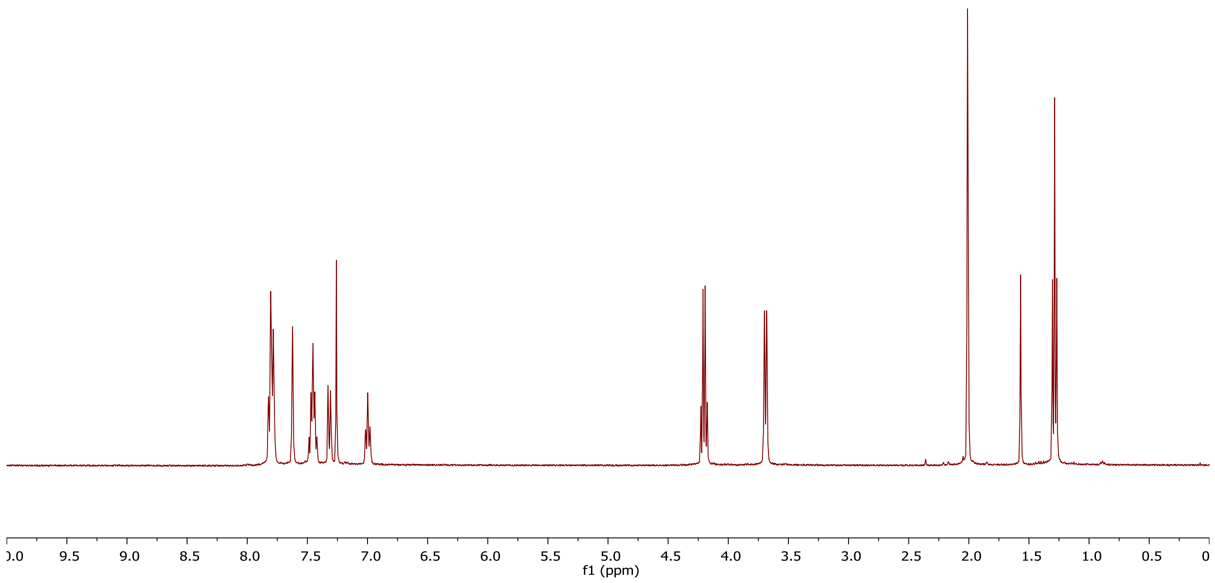
9.5 9.0 8.5 8.0 7.5 7.0 6.5 6.0 5.5 5.0 4.5 4.0 3.5 3.0 2.5 2.0 1.5 1.0 0.5
f1 (ppm)

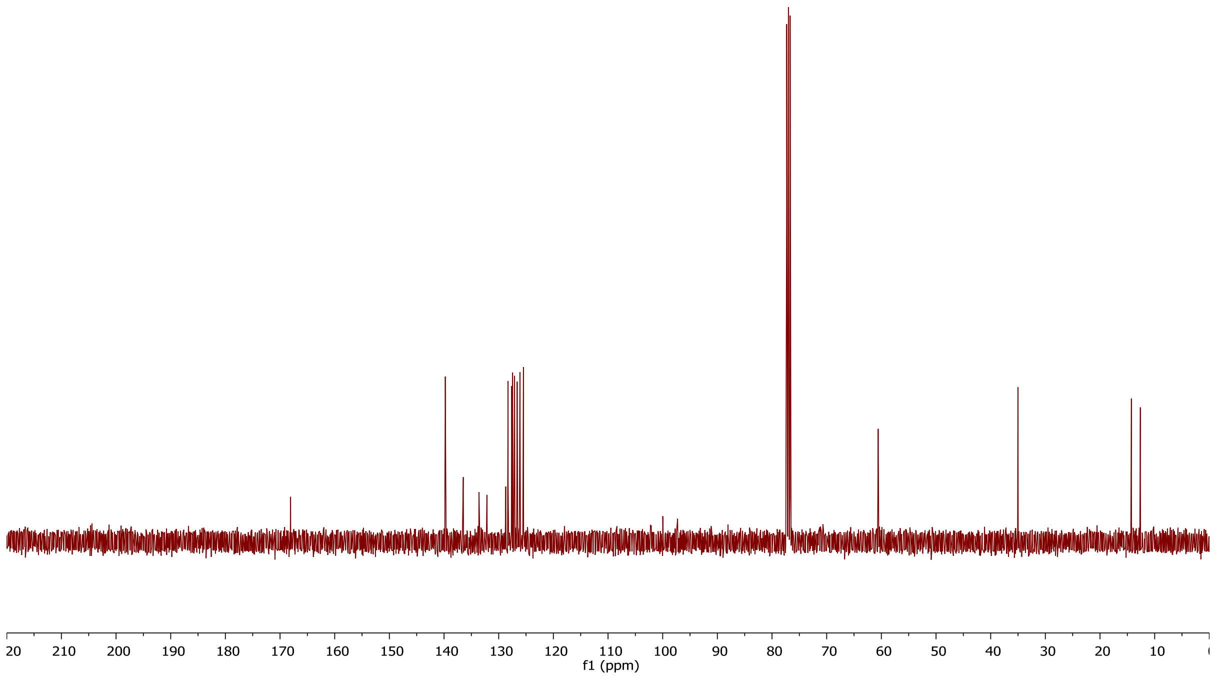
210 200 190 180 170 160 150 140 130 120 110 100 90 80 70 60 50 40 30 20 10
f1 (ppm)

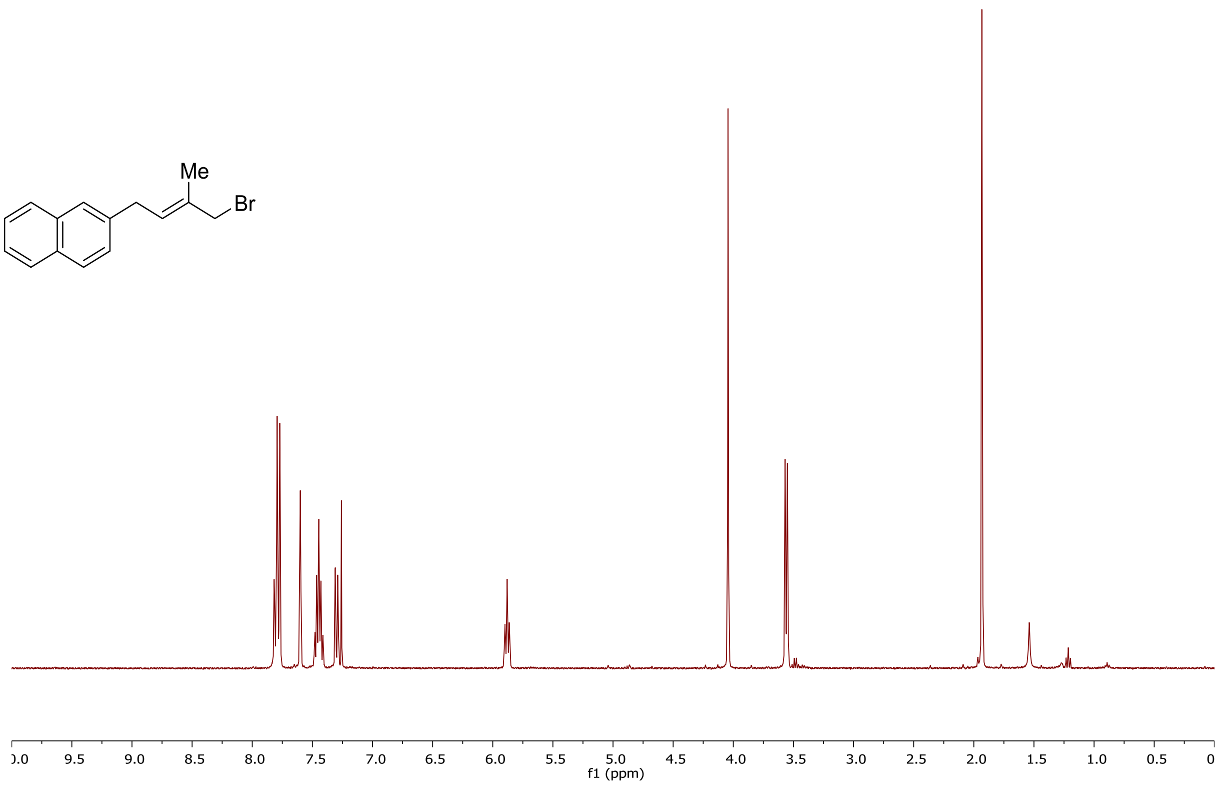
Me
Br
10.0 9.5 9.0 8.5 8.0 7.5 7.0 6.5 6.0 5.5 5.0 4.5 4.0 3.5 3.0 2.5 2.0 1.5 1.0 0.5 0
f1 (ppm)

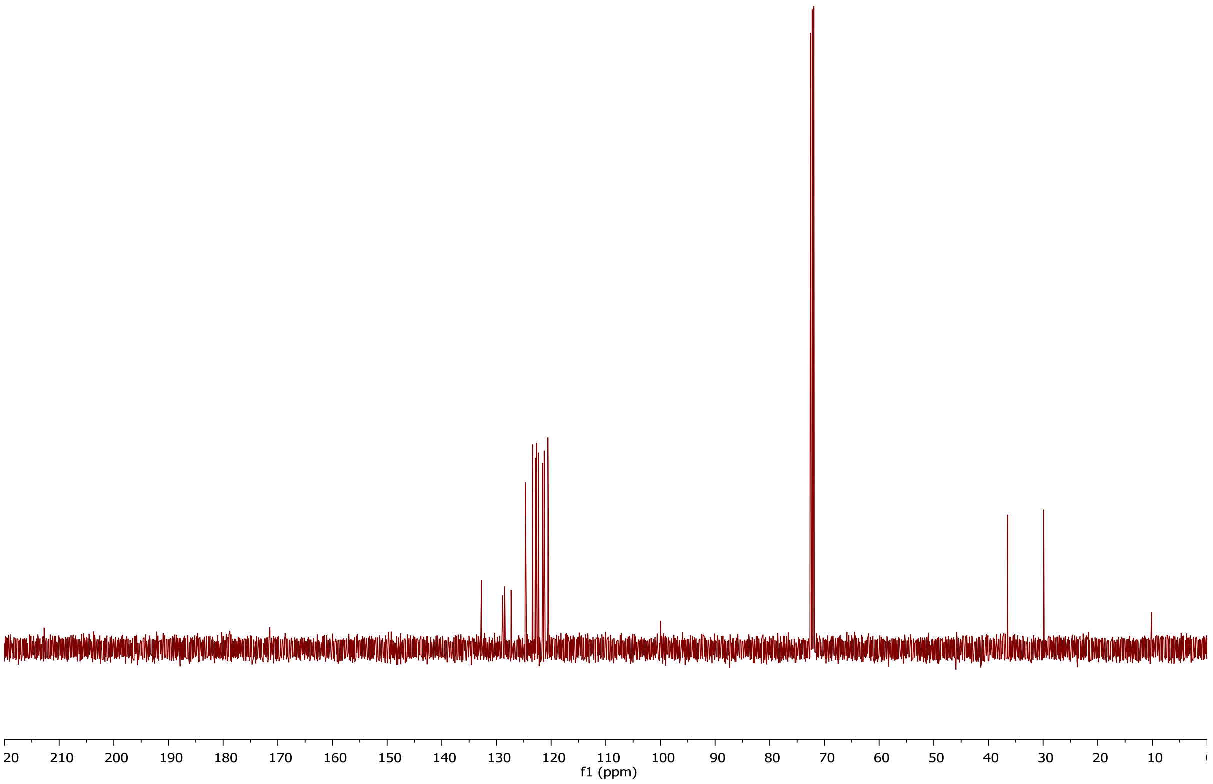
220 210 200 190 180 170 160 150 140 130 120 110 100 90 80 70 60 50 40 30 20 10 0
f1 (ppm)

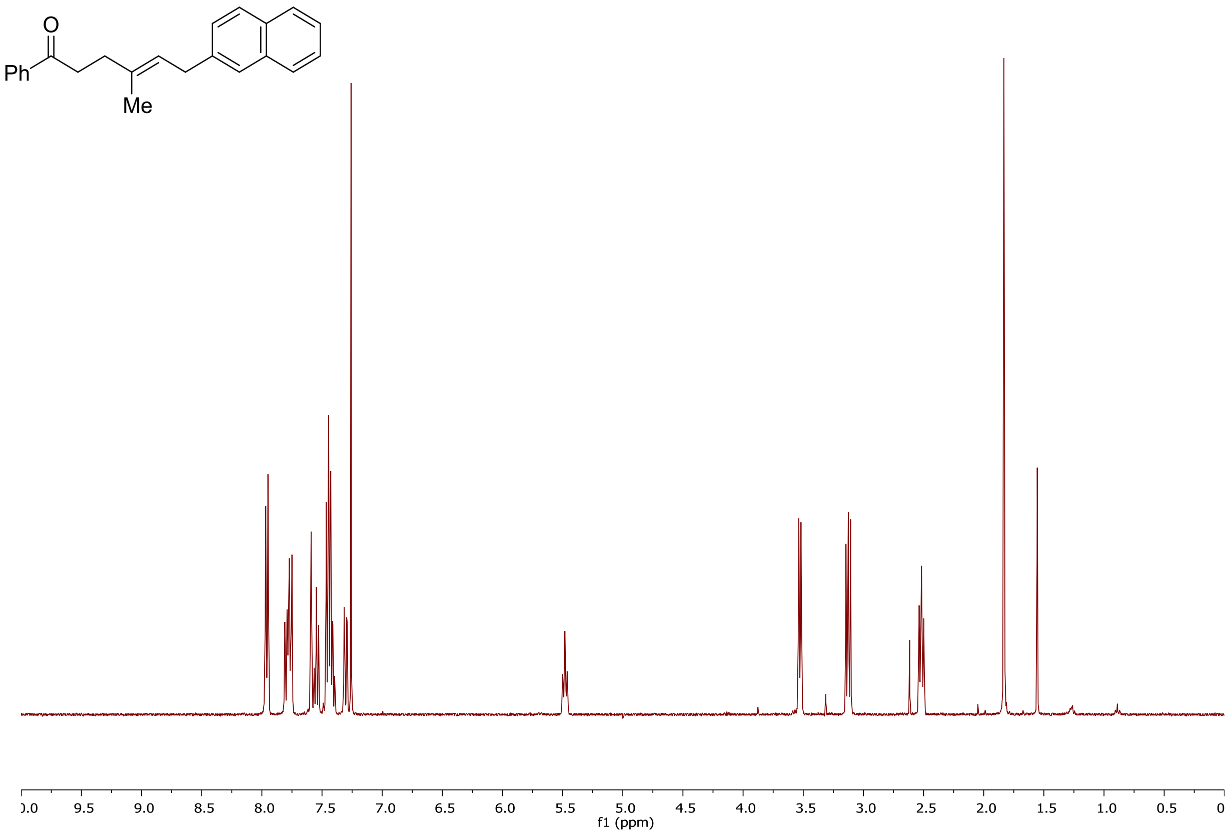
O
Ph
Me
f1 (ppm)

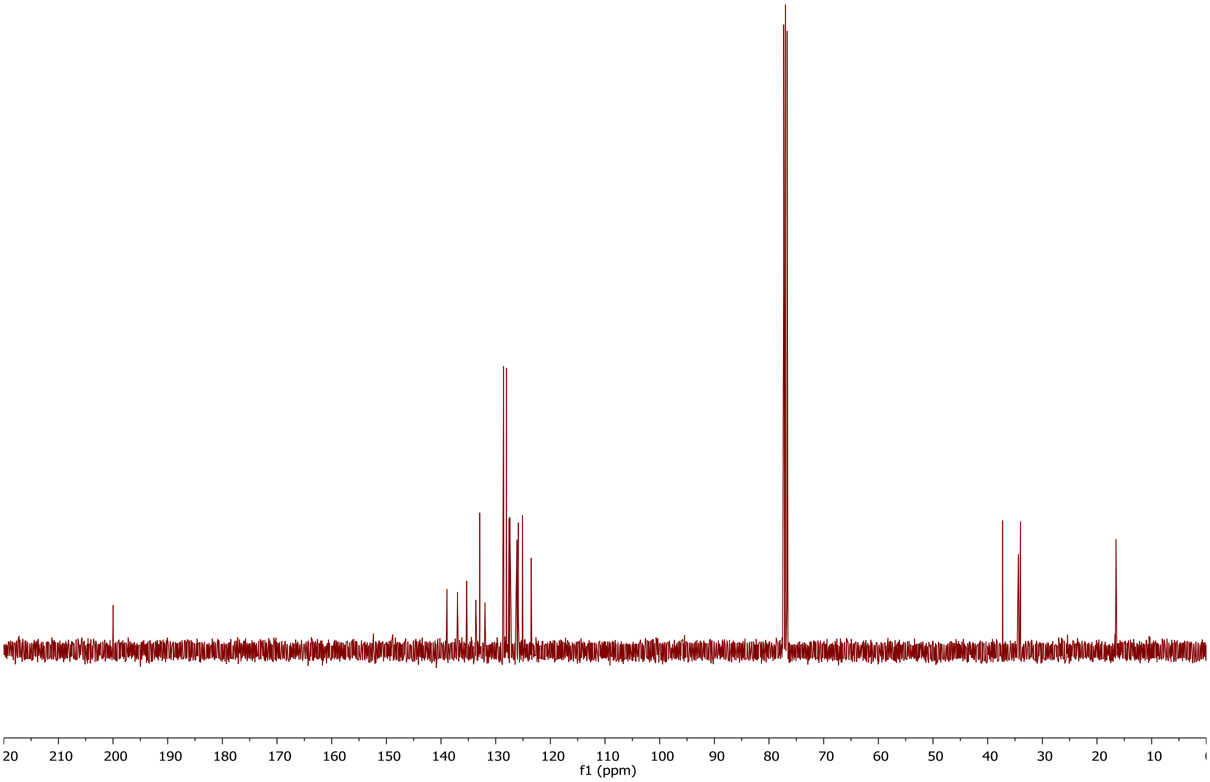
f1 (ppm)

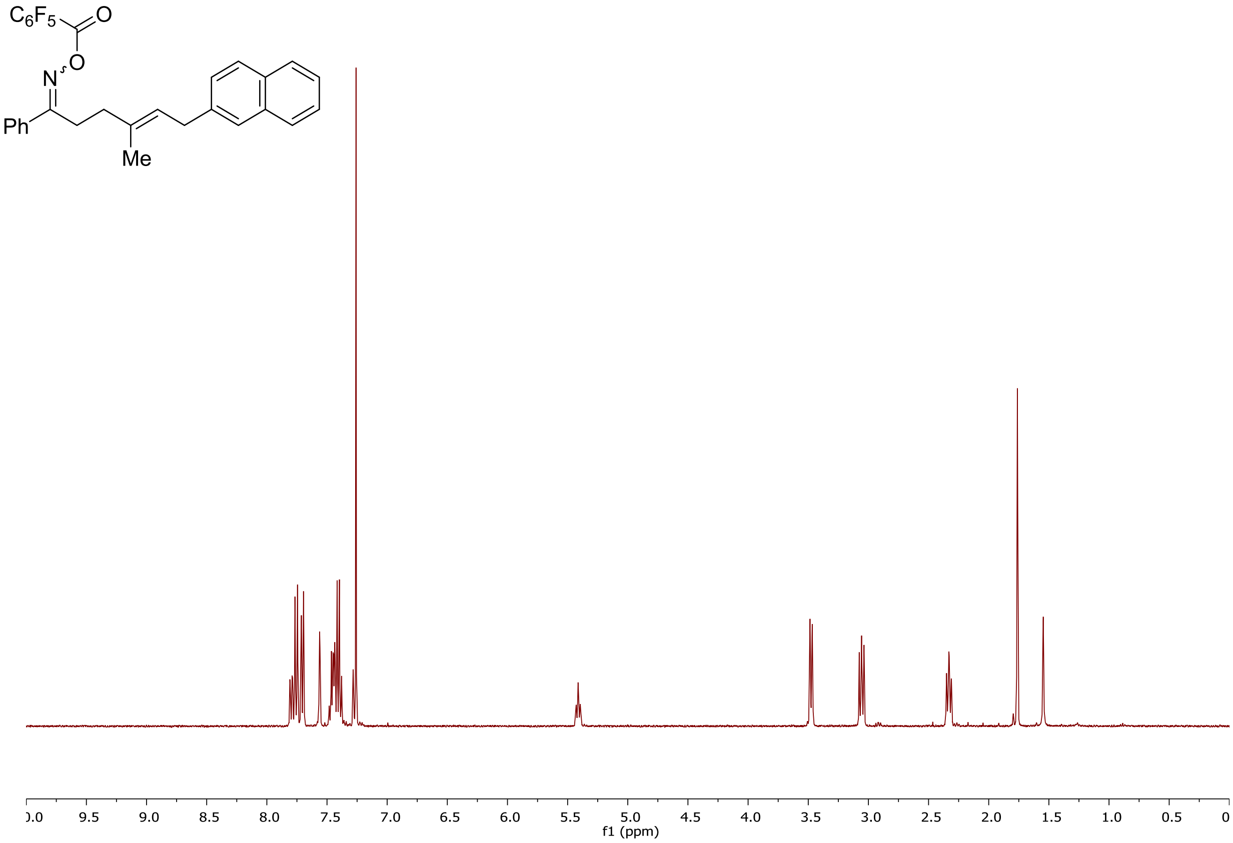
C6F5
O
N
O
Ph
Me
9.5
9.0
8.5
8.0
7.5
7.0
6.5
6.0
5.5
5.0
4.5
4.0
3.5
3.0
2.5
2.0
1.5
1.0
0.5
f1 (ppm)

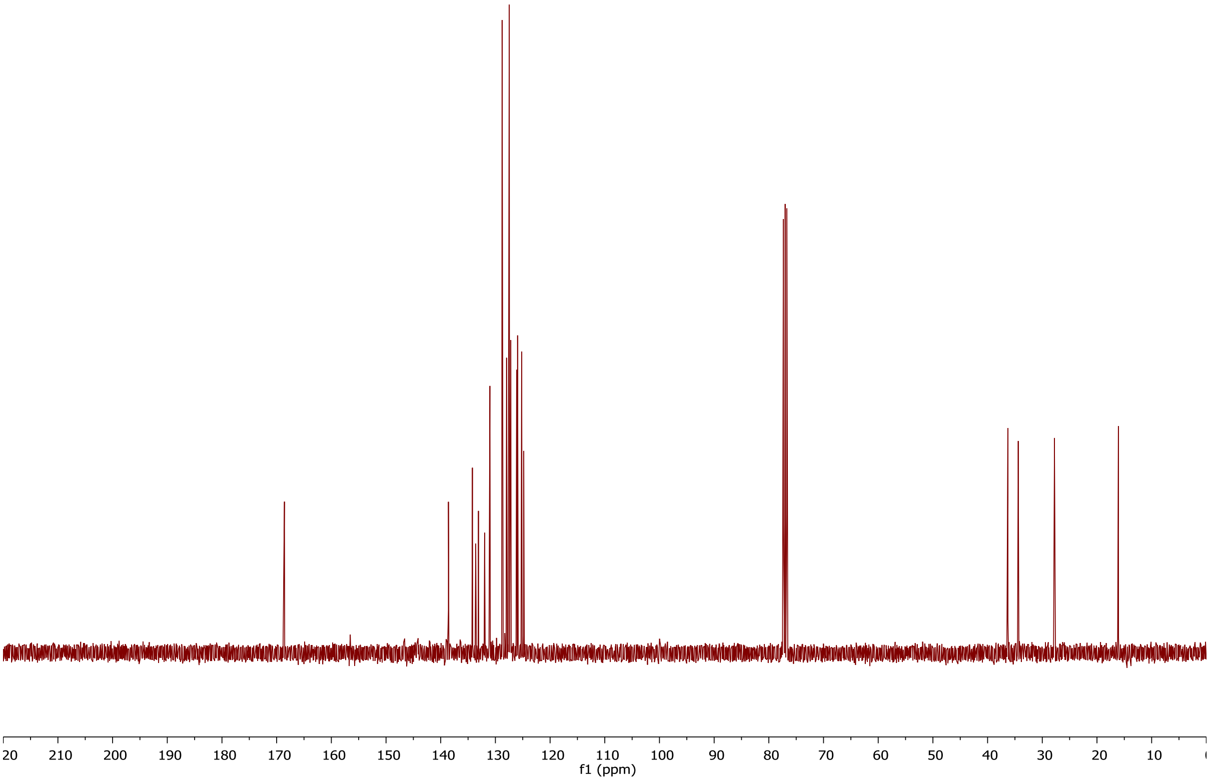
210
200
190
180
170
160
150
140
130
120
110
100
90
80
70
60
50
40
30
20
10
f1 (ppm)

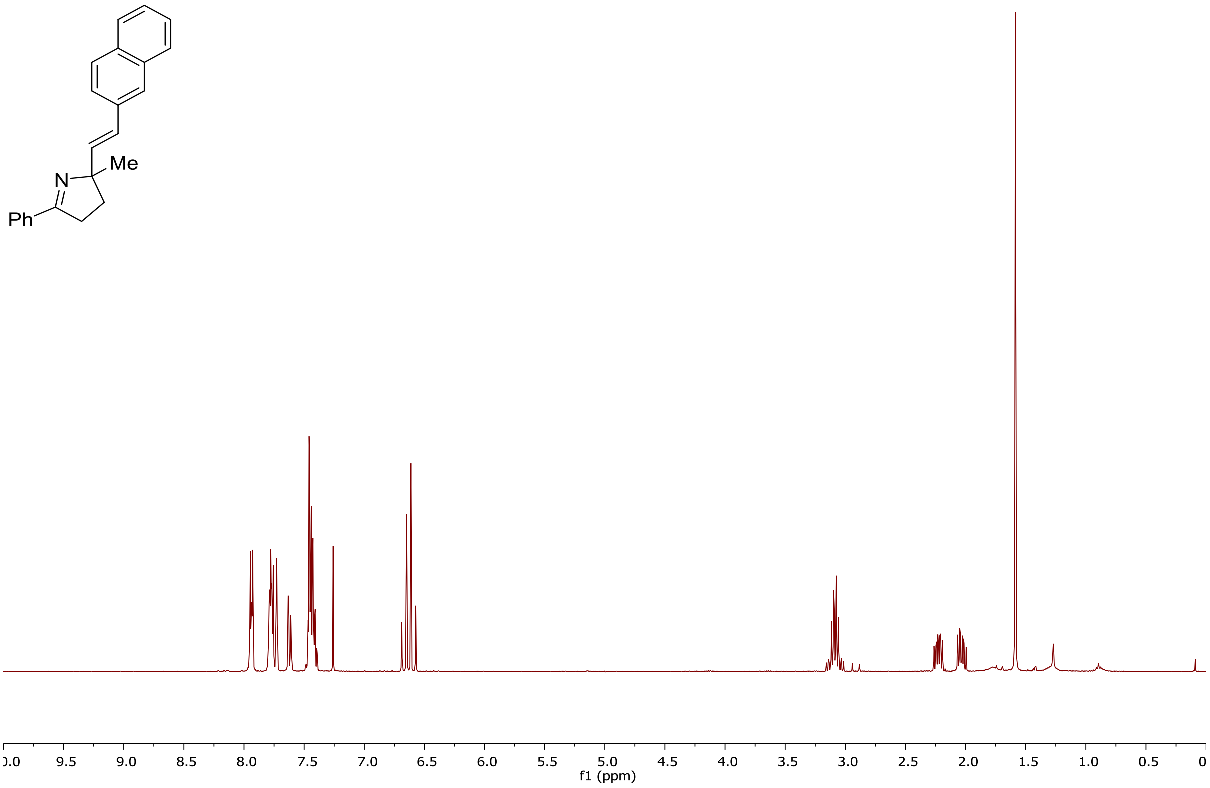
Me
N
Ph
f1 (ppm)

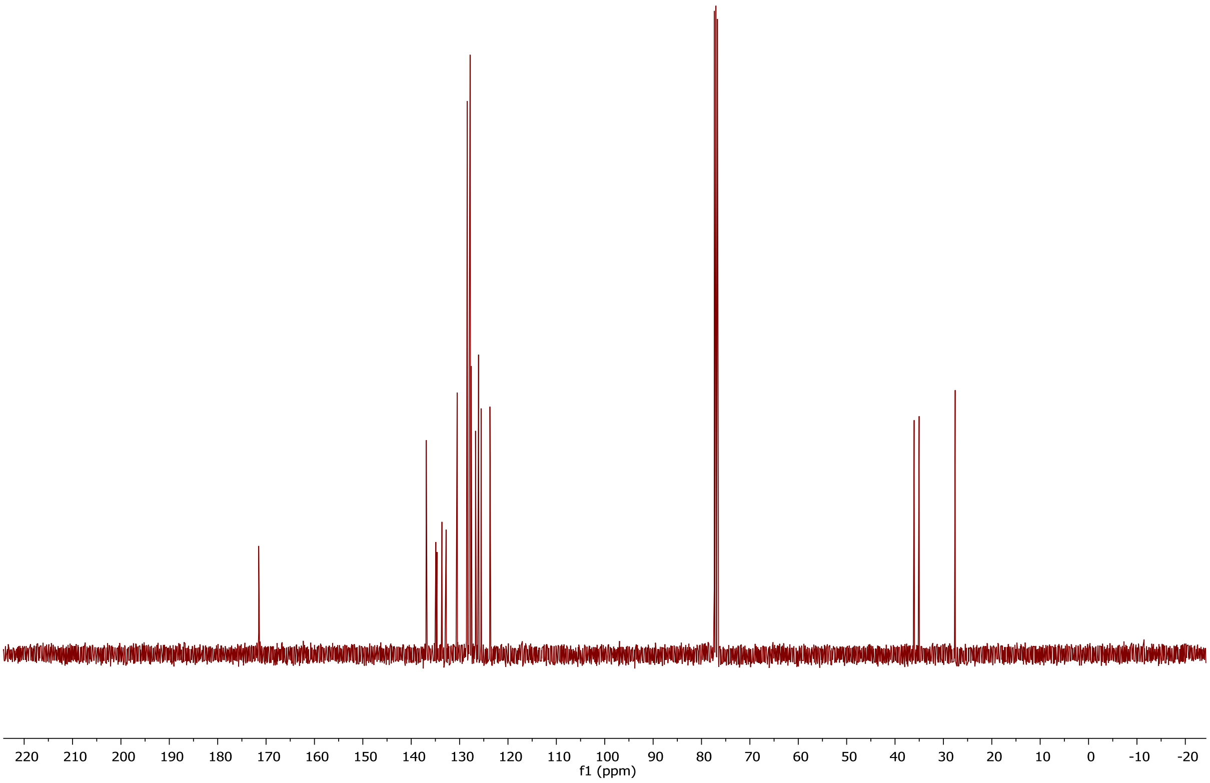
f1 (ppm)

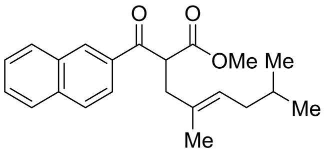
O
O
OMe
Me
Me
Me

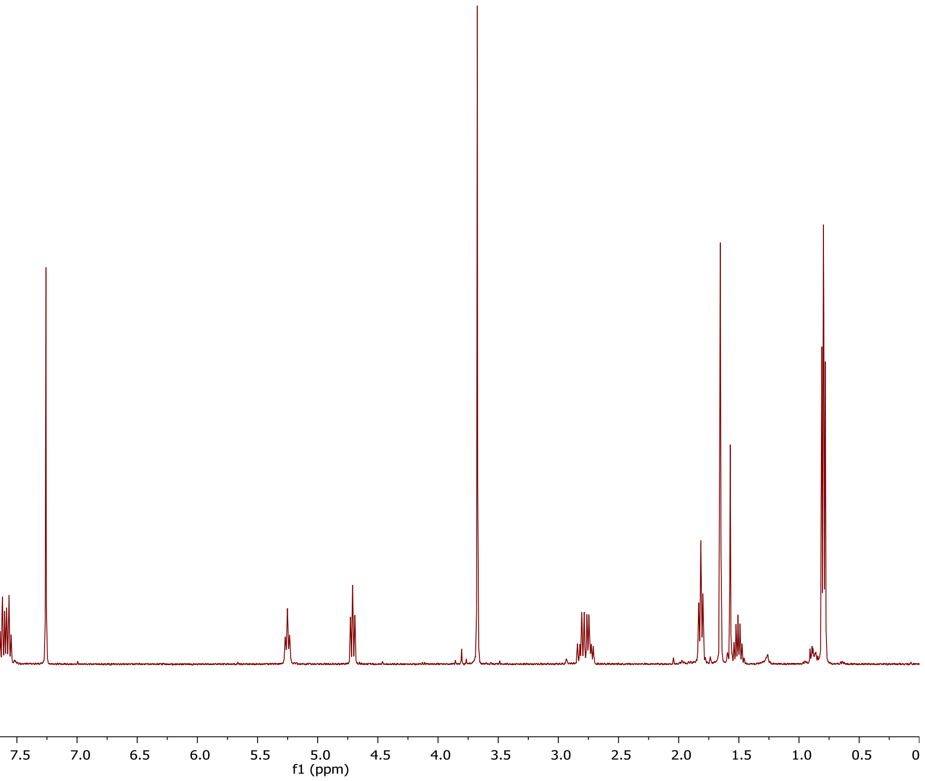
f1 (ppm)

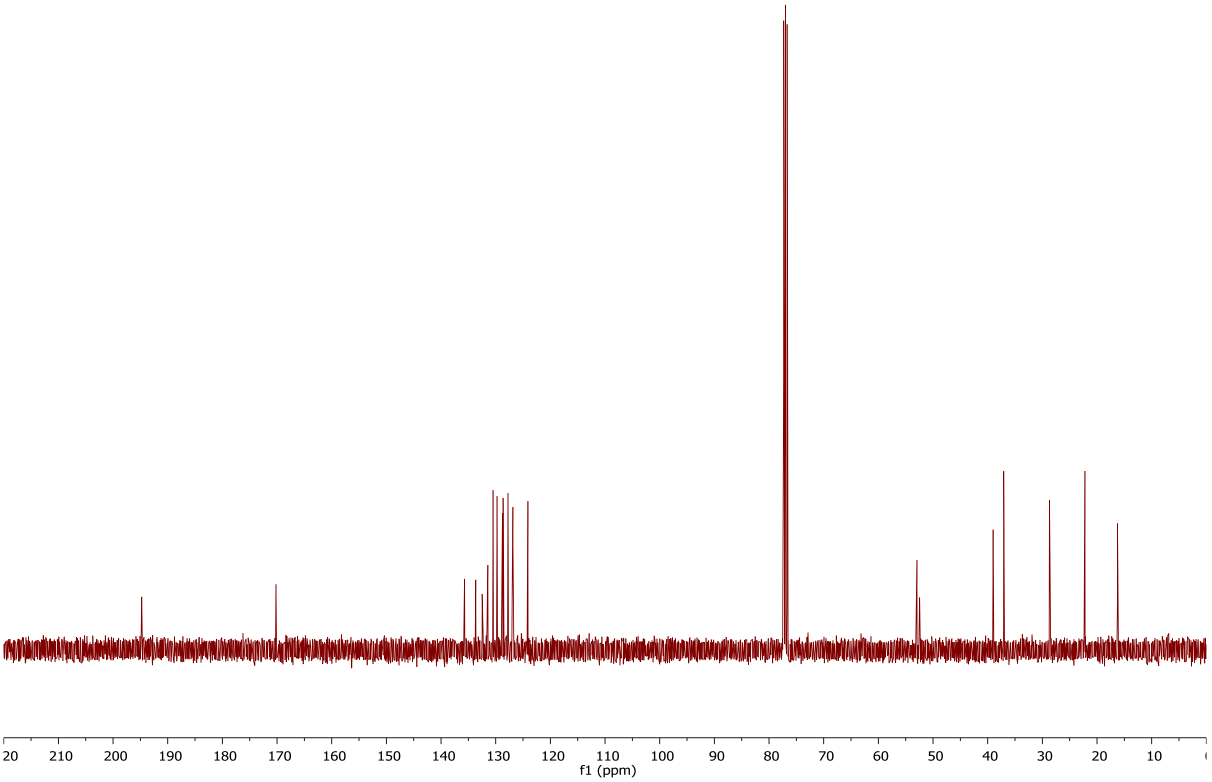
f1 (ppm)

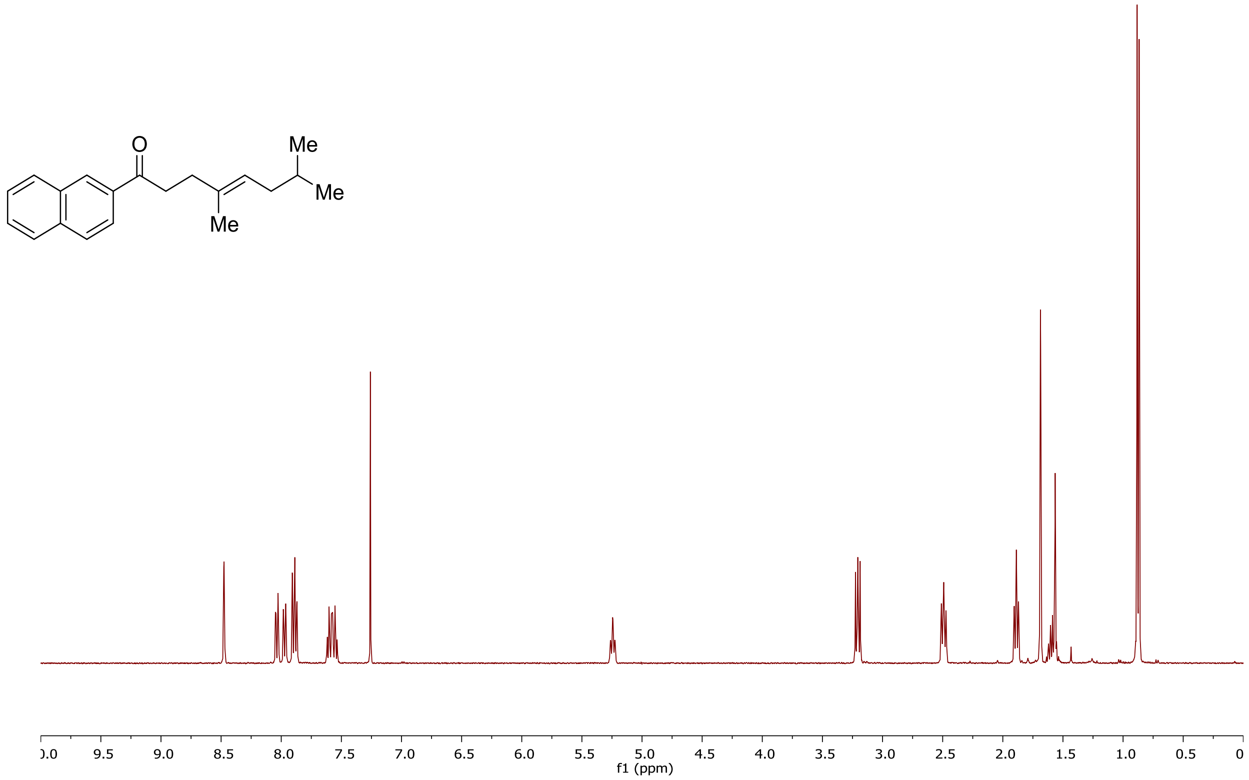
O
Me
Me
Me
9.5 9.0 8.5 8.0 7.5 7.0 6.5 6.0 5.5 5.0 4.5 4.0 3.5 3.0 2.5 2.0 1.5 1.0 0.5
f1 (ppm)

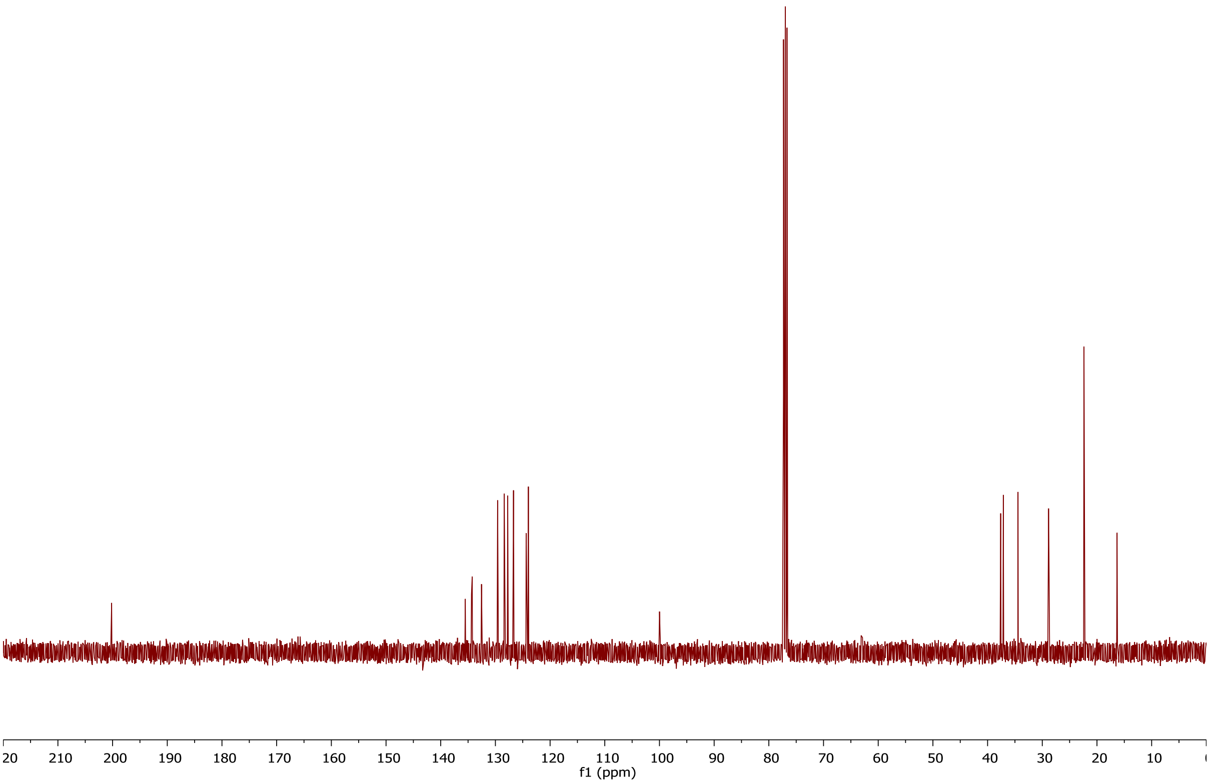
210 200 190 180 170 160 150 140 130 120 110 100 90 80 70 60 50 40 30 20 10
f1 (ppm)

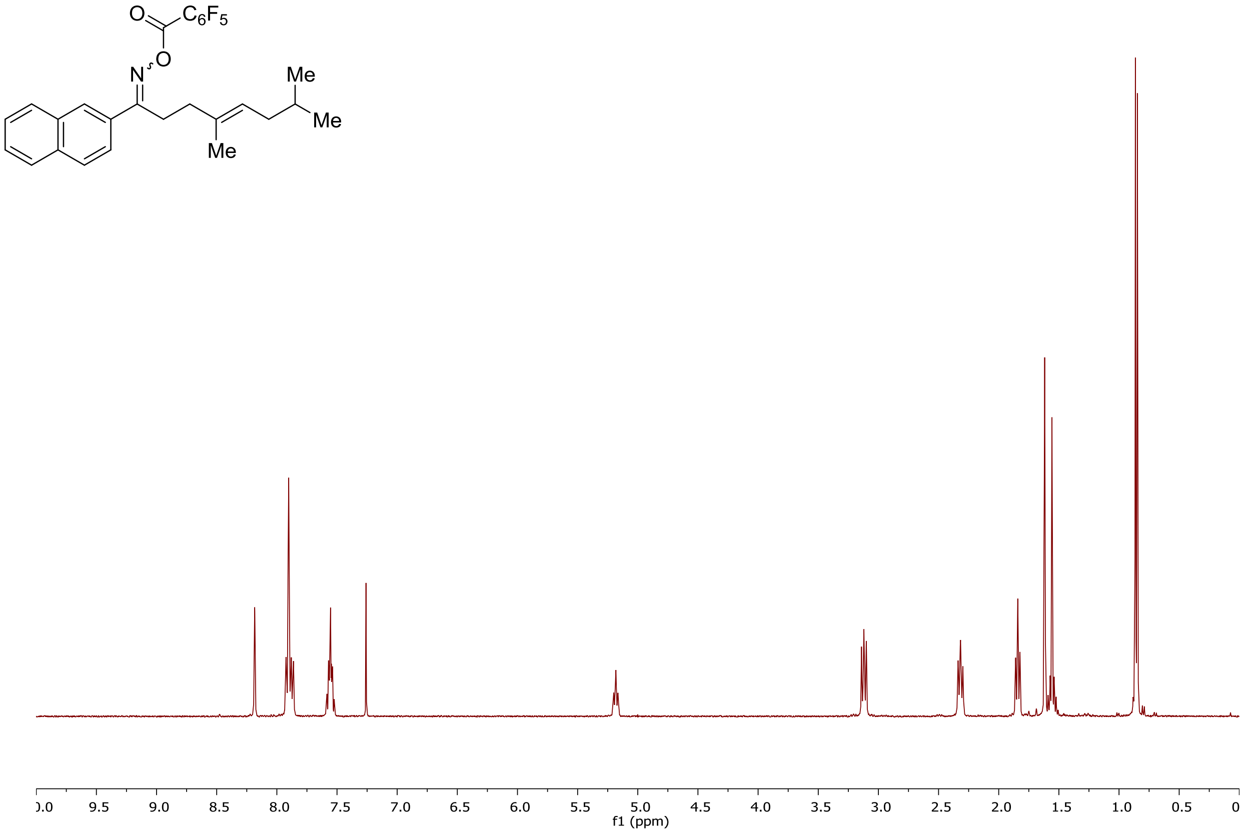
O
C_6F_5
O
N
Me
Me
Me
9.5 9.0 8.5 8.0 7.5 7.0 6.5 6.0 5.5 5.0 4.5 4.0 3.5 3.0 2.5 2.0 1.5 1.0 0.5 0
f1 (ppm)

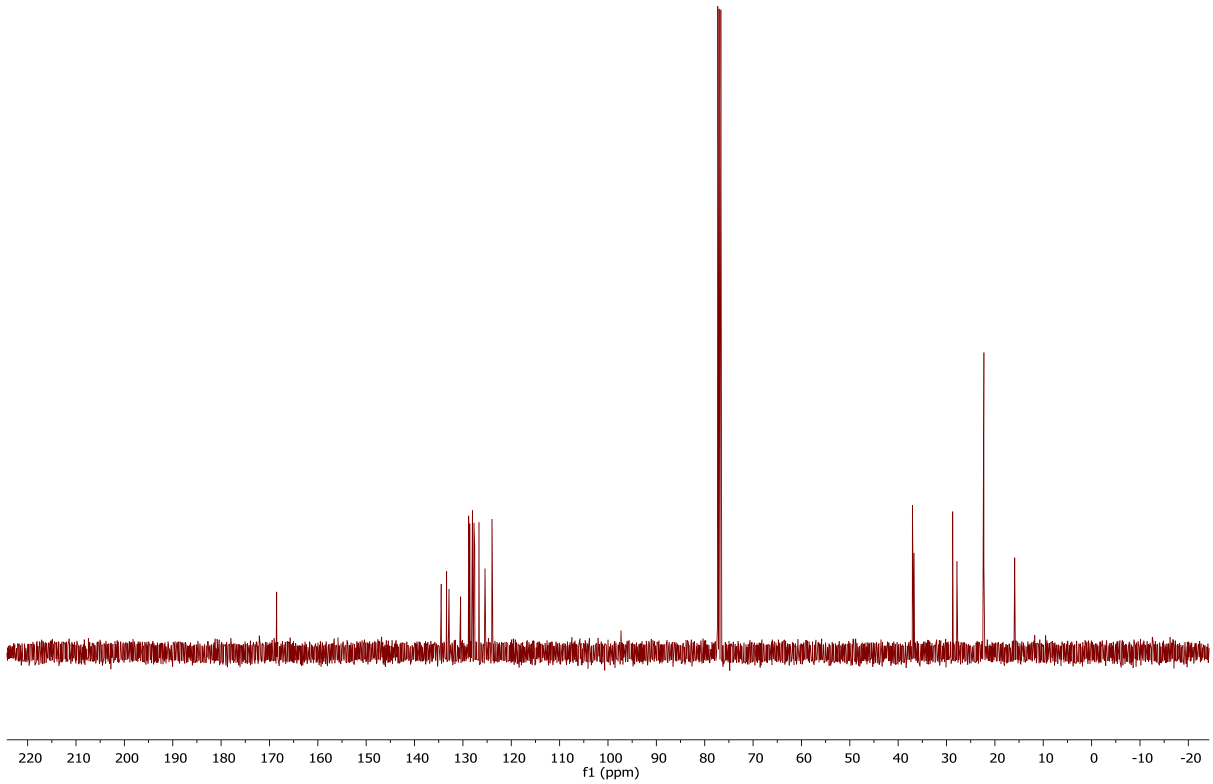
220 210 200 190 180 170 160 150 140 130 120 110 100 90 80 70 60 50 40 30 20 10 0 -10 -20
f1 (ppm)

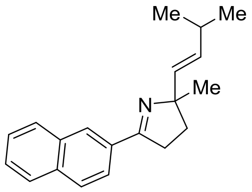
Me
Me
Me
N

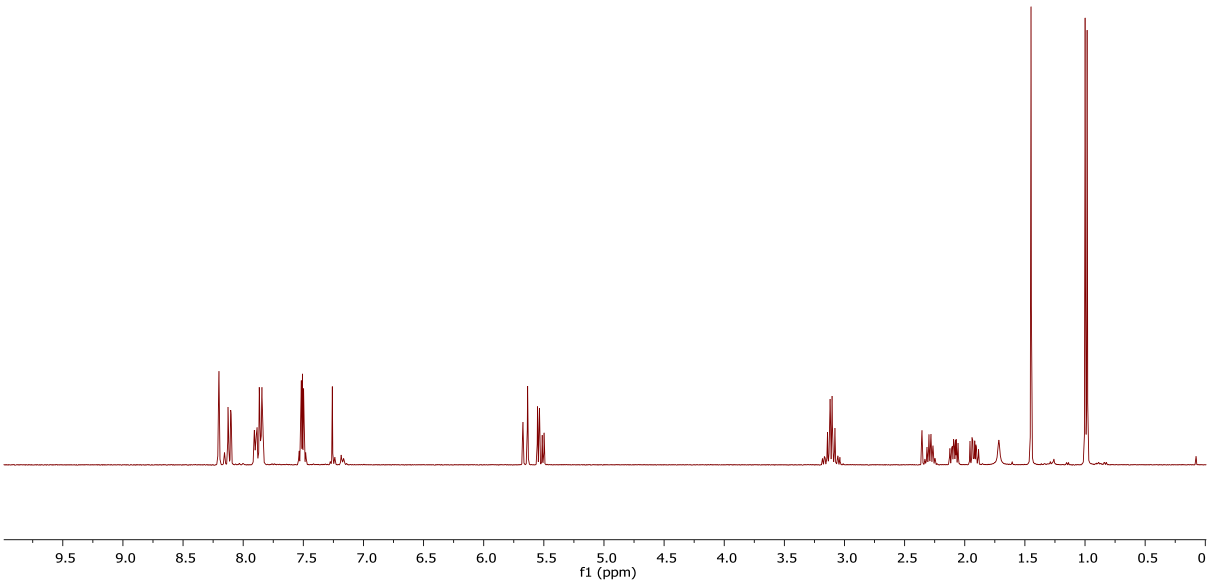
9.5 9.0 8.5 8.0 7.5 7.0 6.5 6.0 5.5 5.0 4.5 4.0 3.5 3.0 2.5 2.0 1.5 1.0 0.5 0
f1 (ppm)

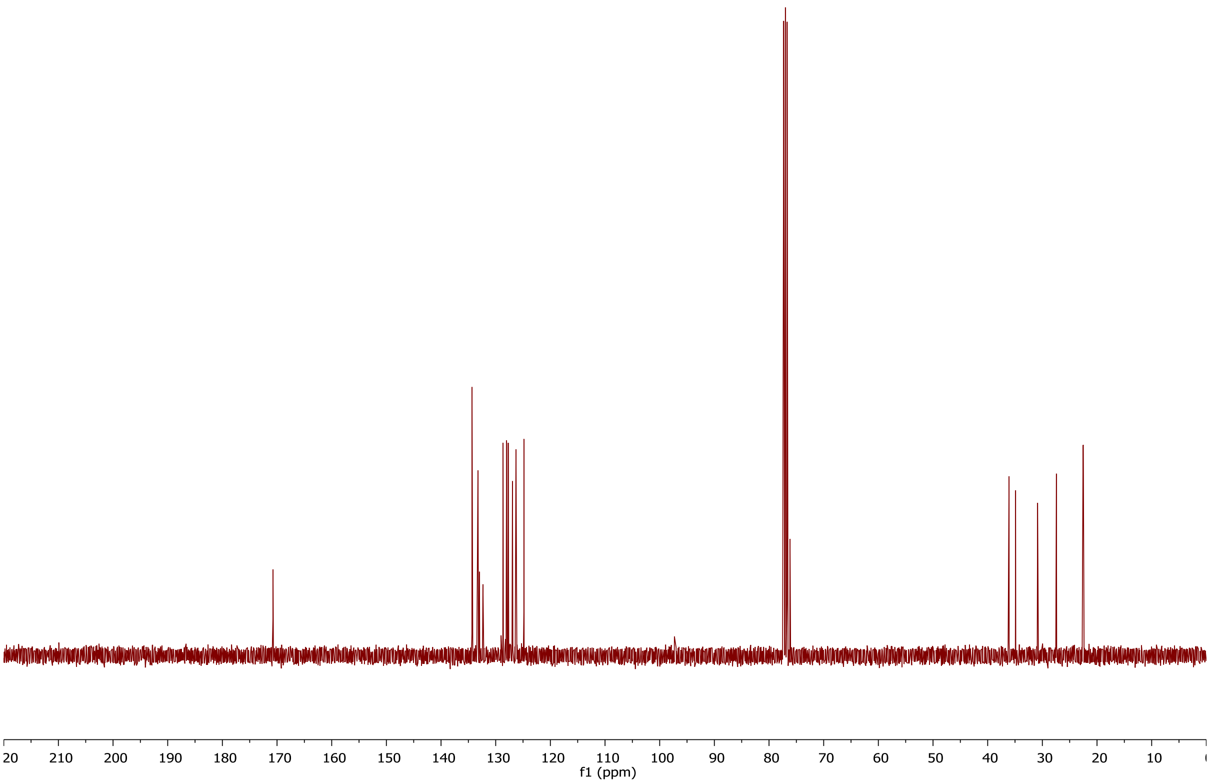
20 210 200 190 180 170 160 150 140 130 120 110 100 90 80 70 60 50 40 30 20 10
f1 (ppm)

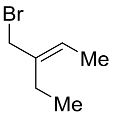
Br
Me
Me

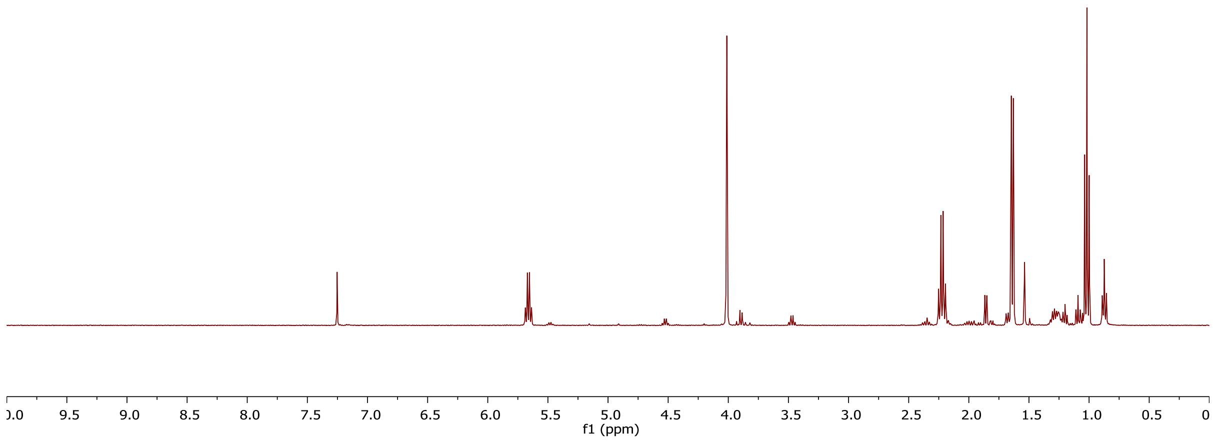
f1 (ppm)

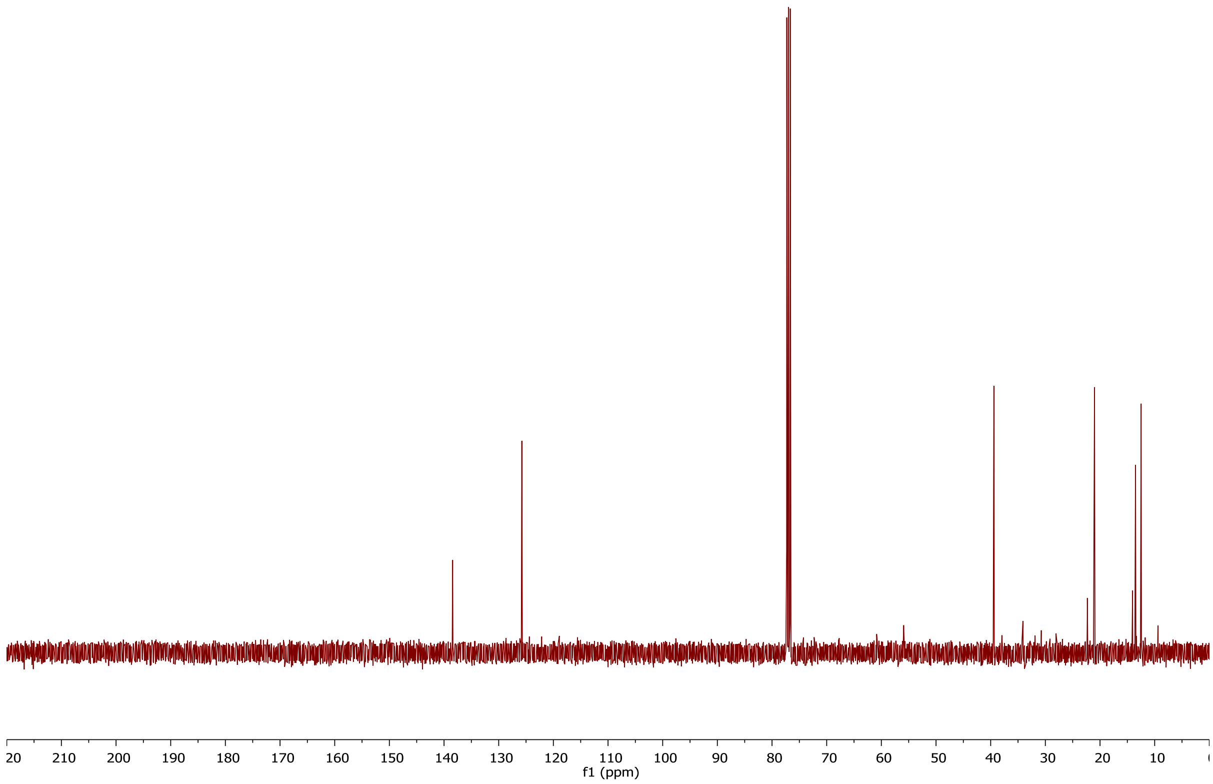
f1 (ppm)

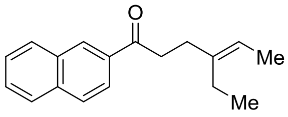
O
Me
Me

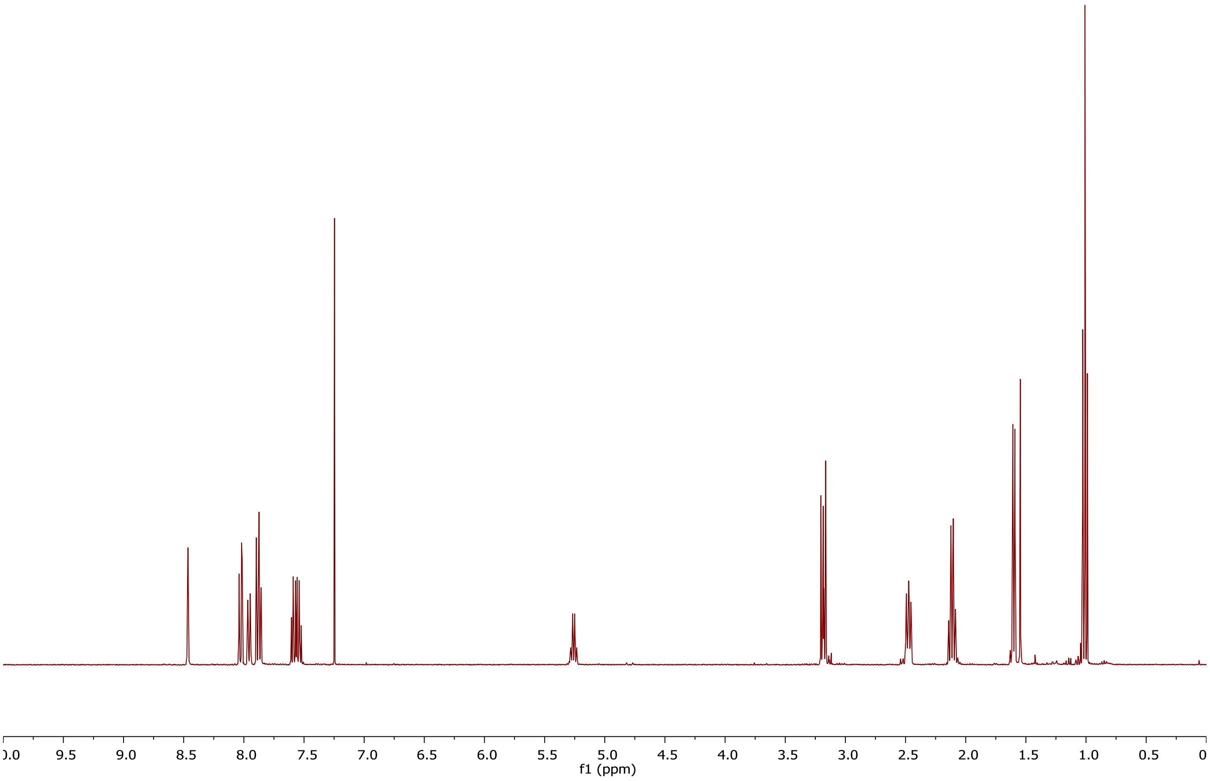
f1 (ppm)

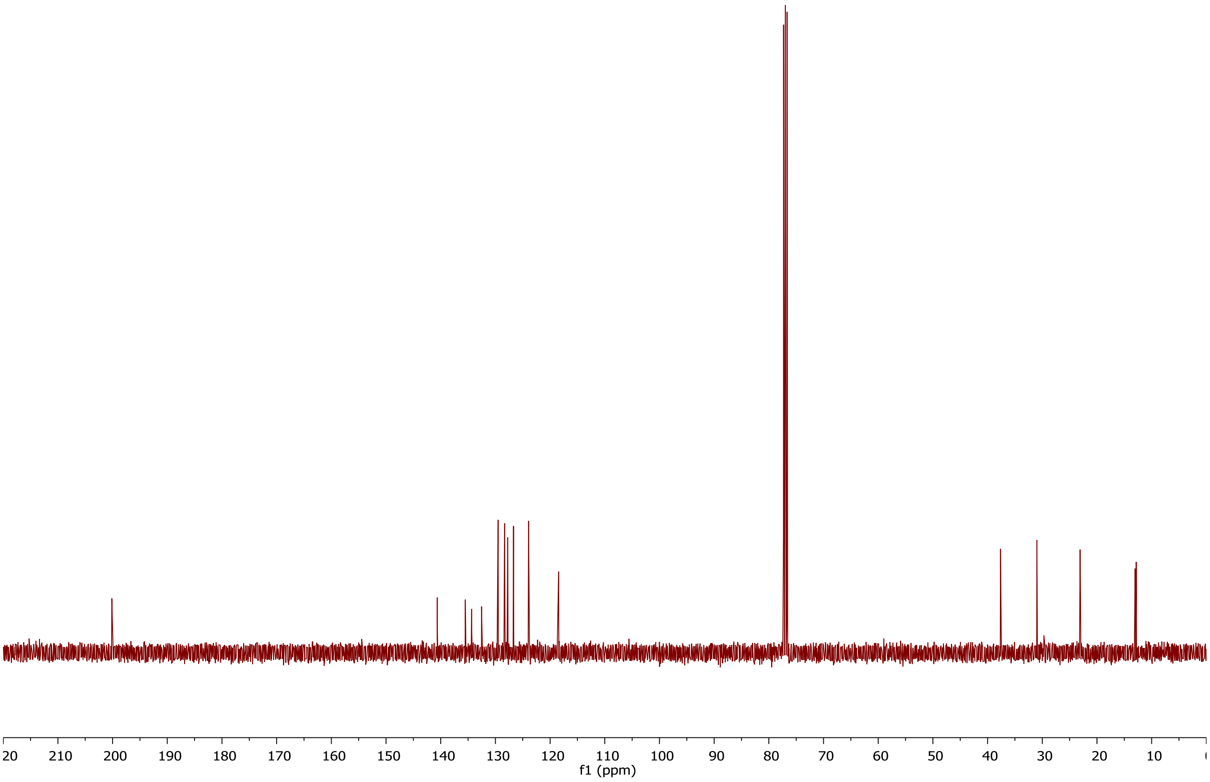
f1 (ppm)

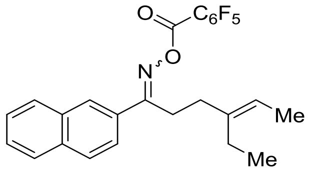
O
C6F5
O
N
Me
Me

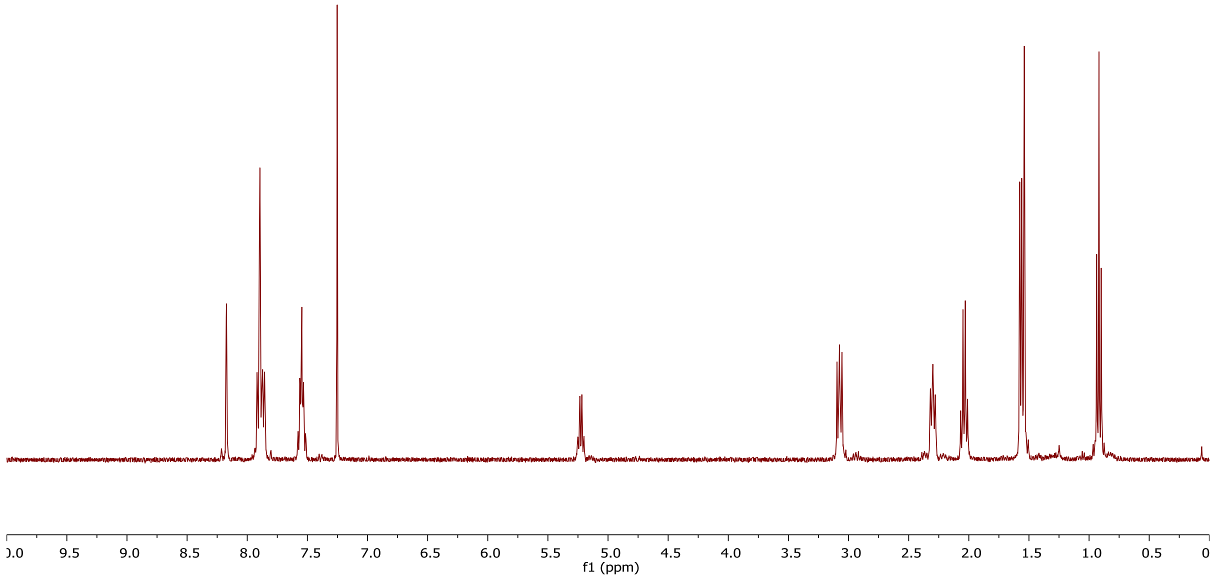
f1 (ppm)

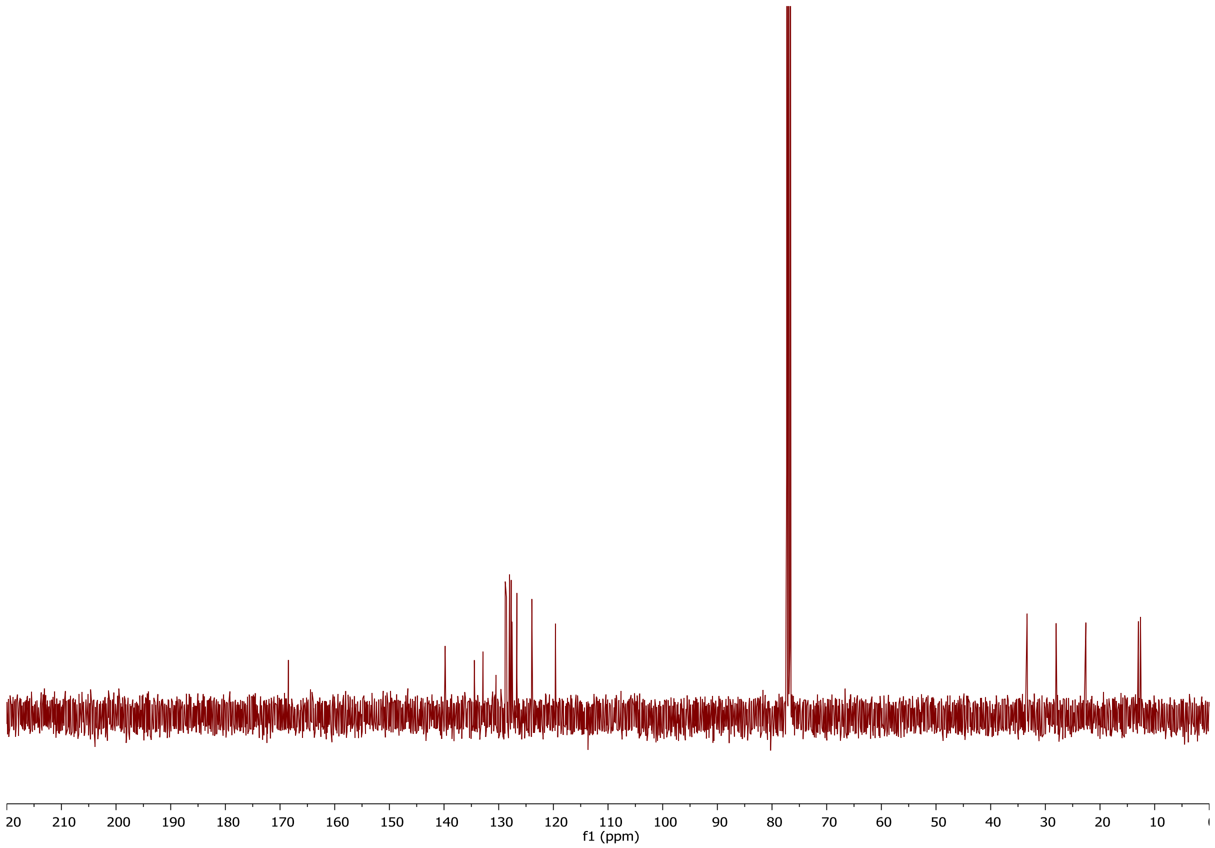
f1 (ppm)

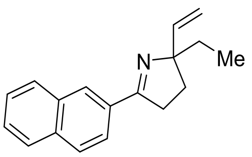
N
Me

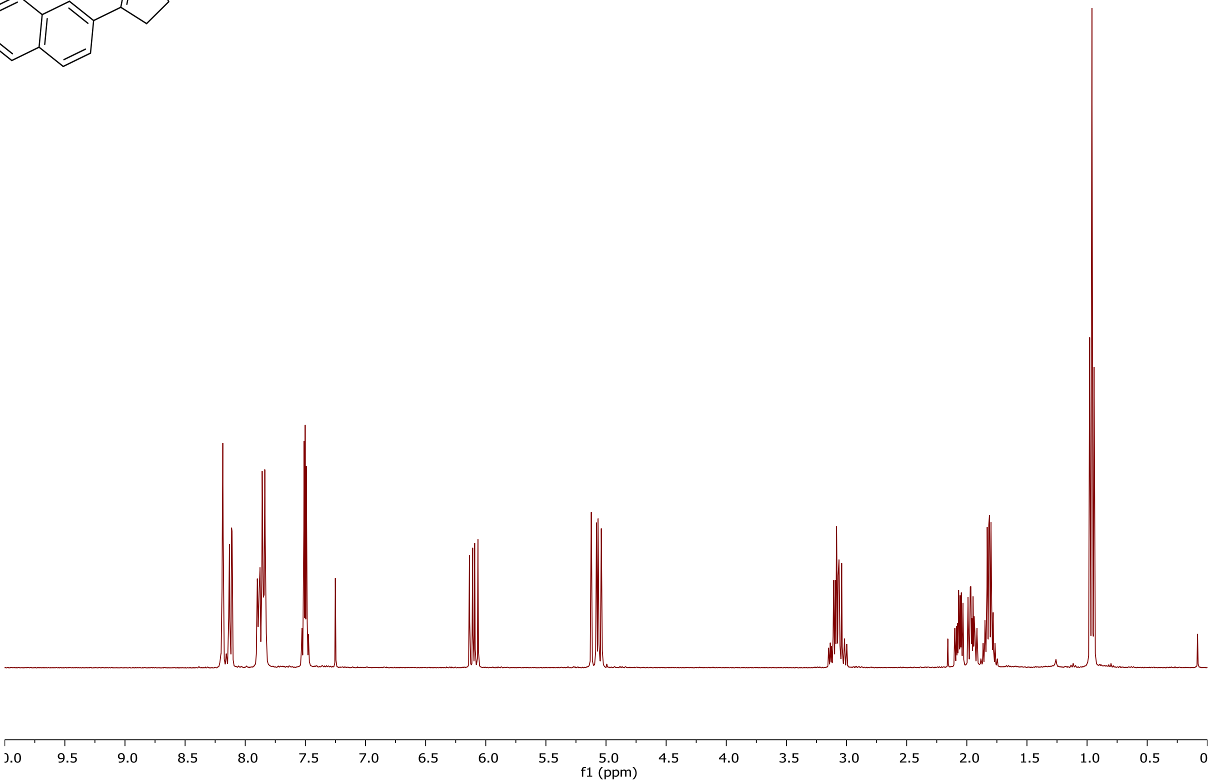
0.0 9.5 9.0 8.5 8.0 7.5 7.0 6.5 6.0 5.5 5.0 4.5 4.0 3.5 3.0 2.5 2.0 1.5 1.0 0.5 0
f1 (ppm)

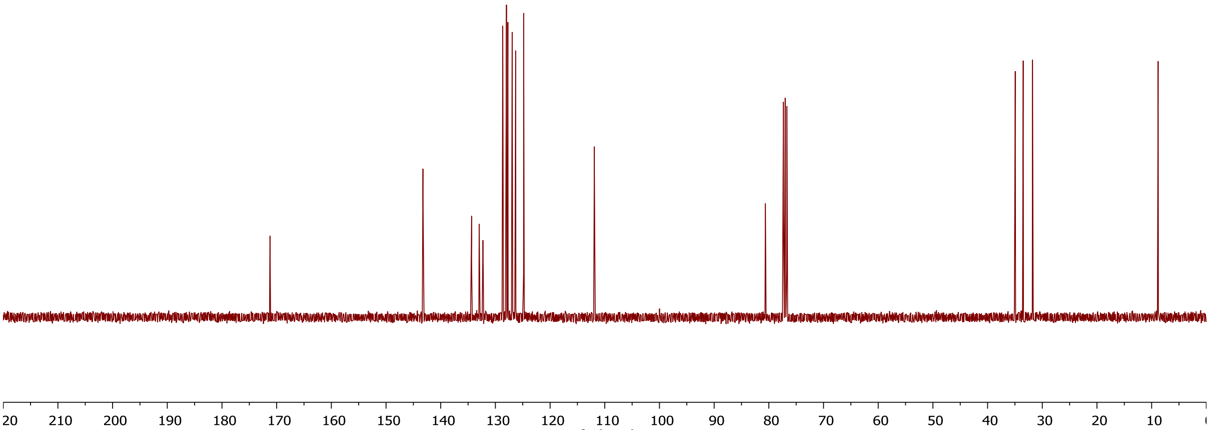
20 210 200 190 180 170 160 150 140 130 120 110 100 90 80 70 60 50 40 30 20 10 0
f1 (ppm)

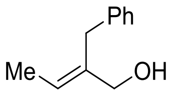
Ph
Me
OH

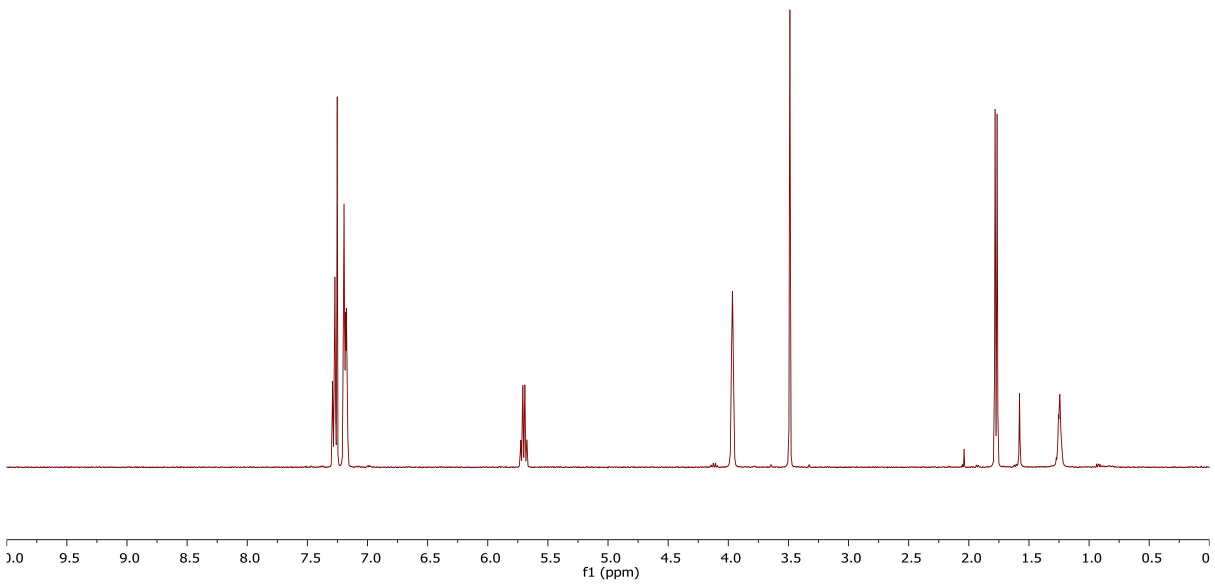
f1 (ppm)

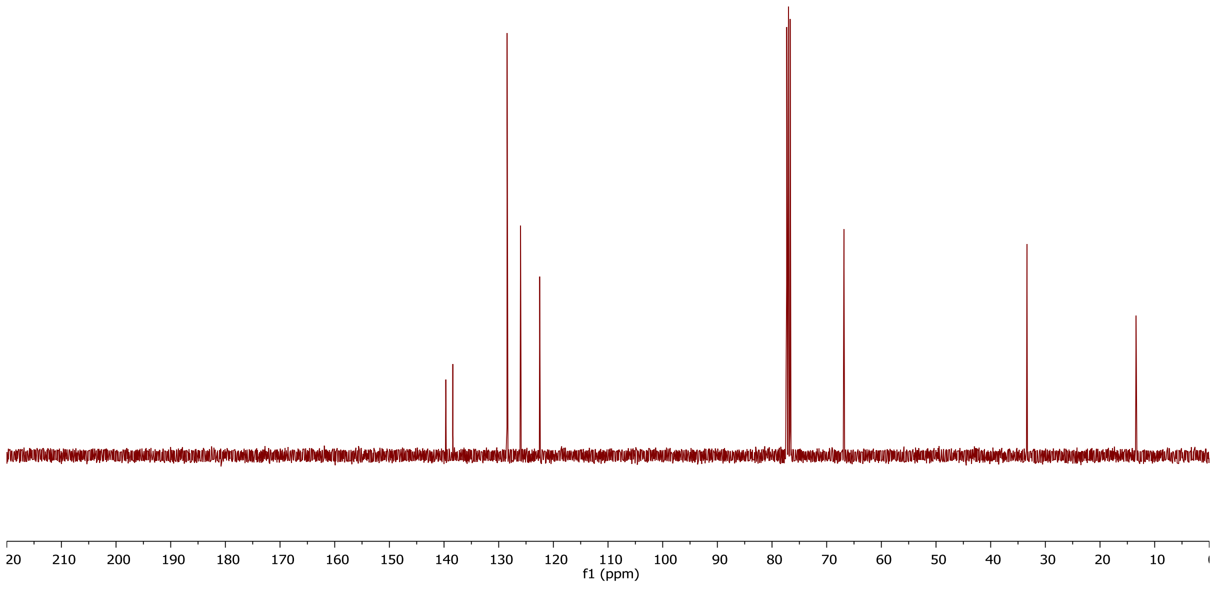
f1 (ppm)

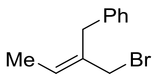
Ph
Me
Br

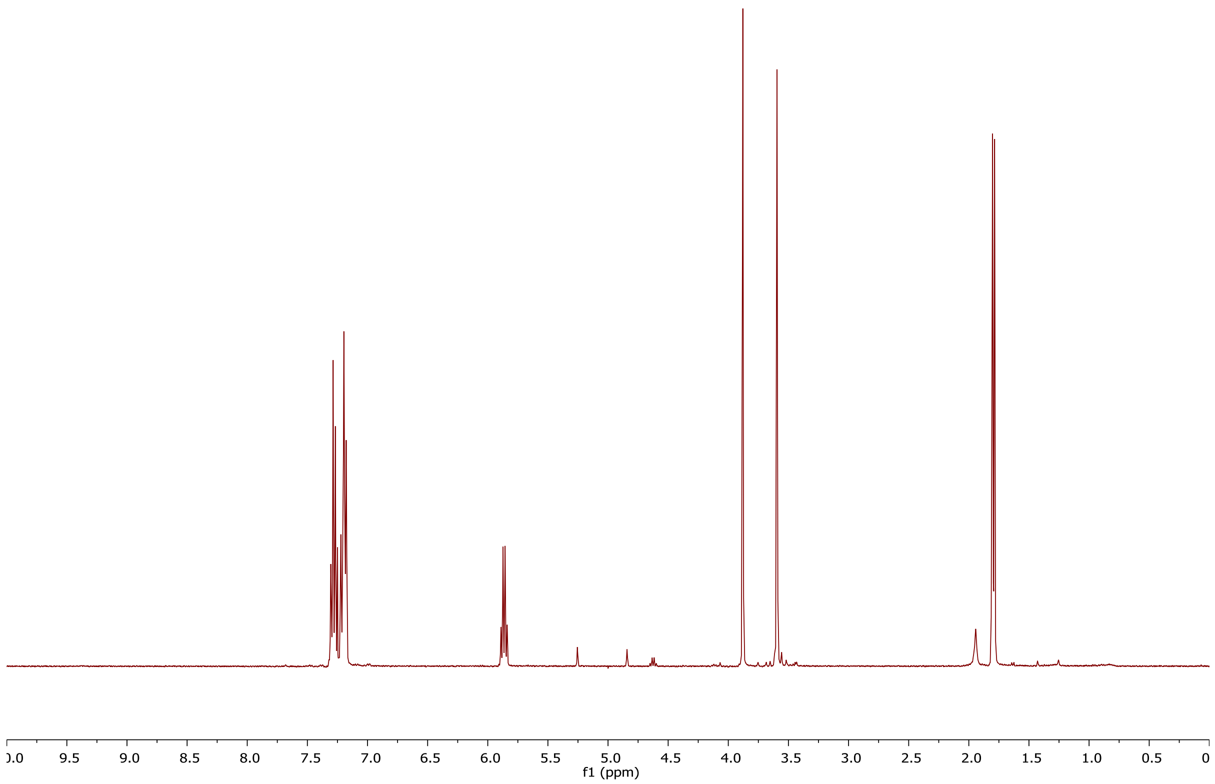
).0 9.5 9.0 8.5 8.0 7.5 7.0 6.5 6.0 5.5 5.0 4.5 4.0 3.5 3.0 2.5 2.0 1.5 1.0 0.5 0
f1 (ppm)

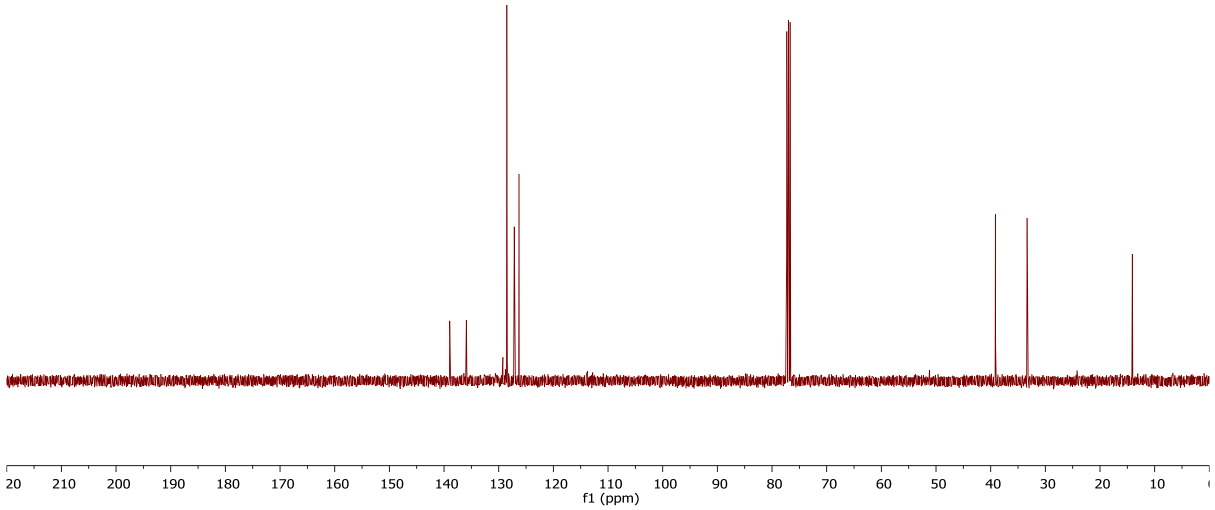
20 210 200 190 180 170 160 150 140 130 120 110 100 90 80 70 60 50 40 30 20 10
f1 (ppm)

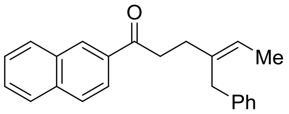
O
Me
Ph

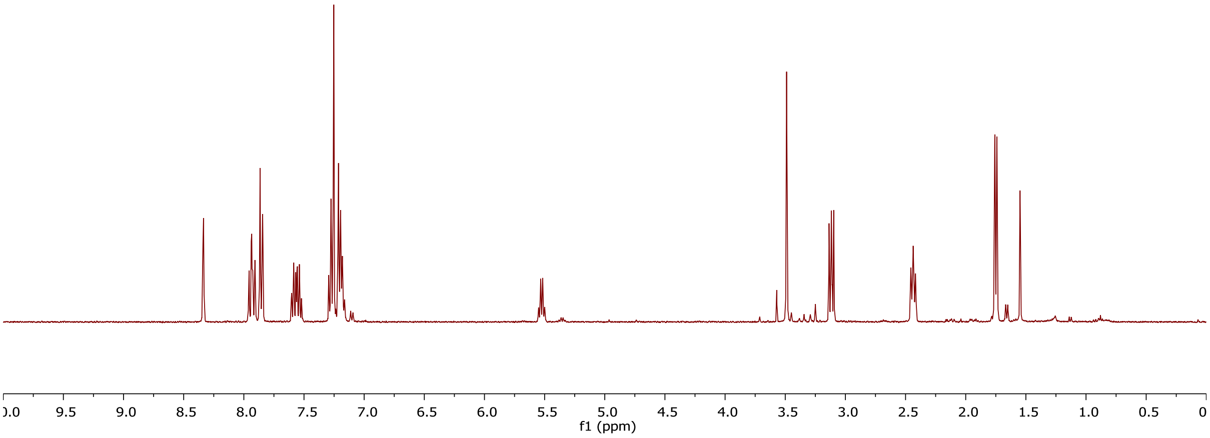
9.5
9.0
8.5
8.0
7.5
7.0
6.5
6.0
5.5
5.0
4.5
4.0
3.5
3.0
2.5
2.0
1.5
1.0
0.5
f1 (ppm)

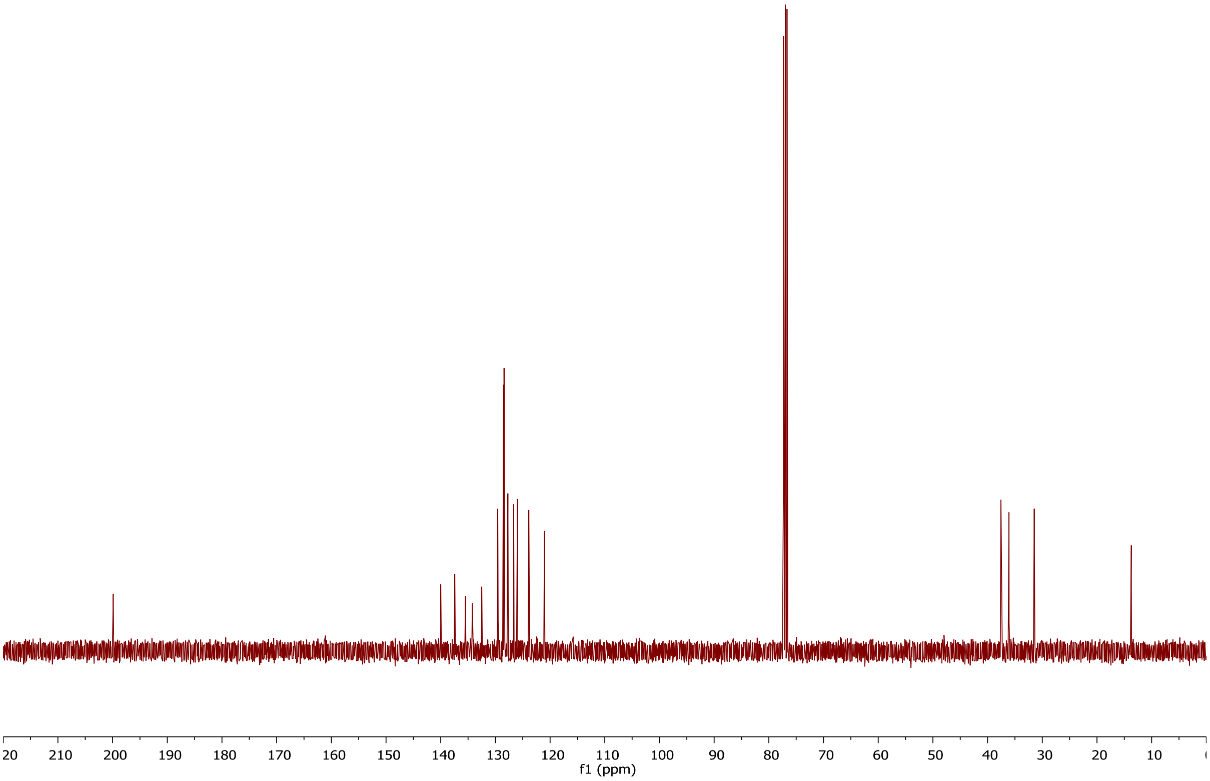
210
200
190
180
170
160
150
140
130
120
110
100
90
80
70
60
50
40
30
20
10
f1 (ppm)

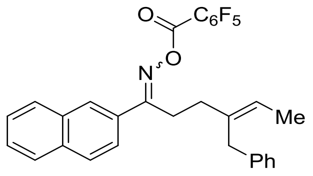

O
C_6F_5
O
N
Me
Ph

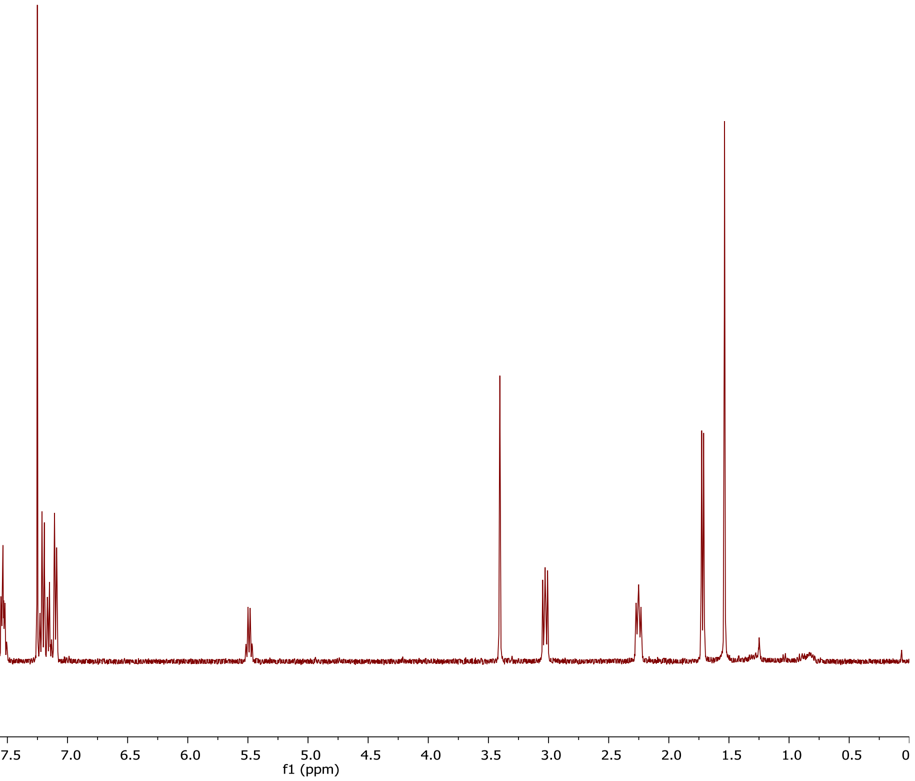

f1 (ppm)

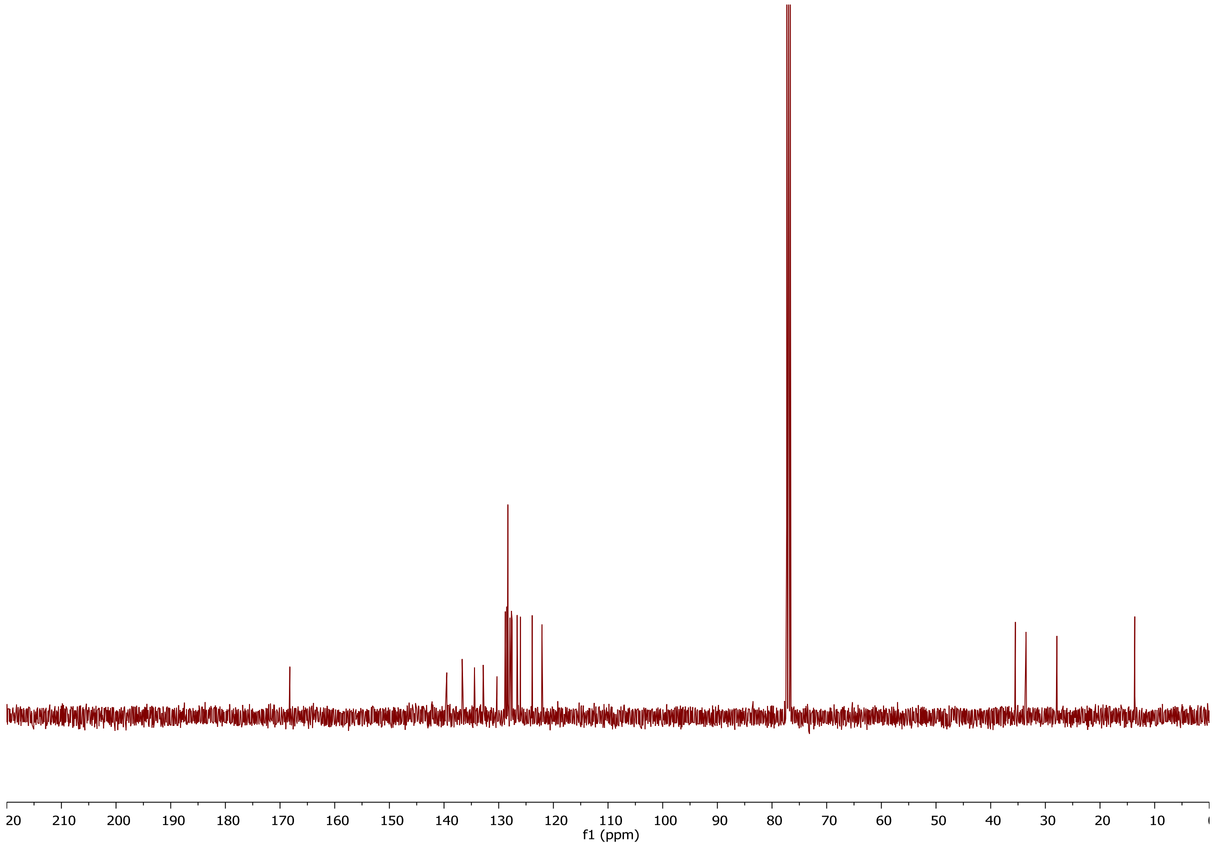

f1 (ppm)

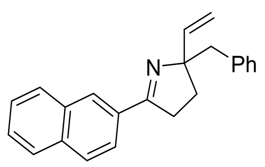
N
Ph

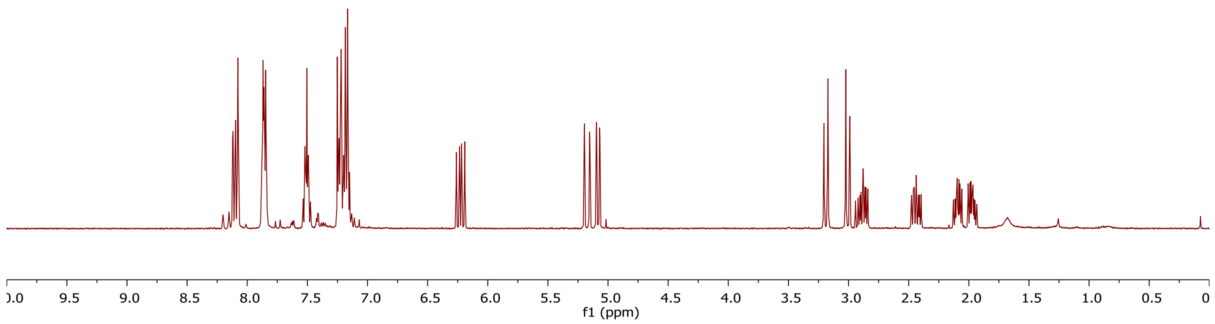
f1 (ppm)

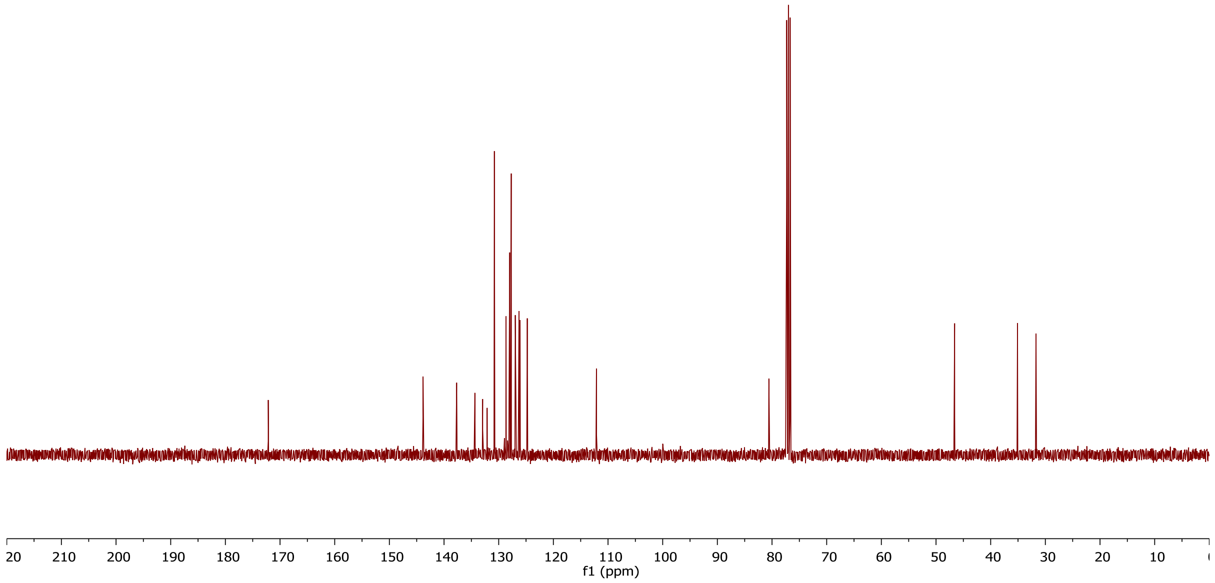
f1 (ppm)

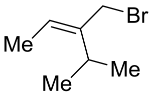
Br
Me
Me
Me

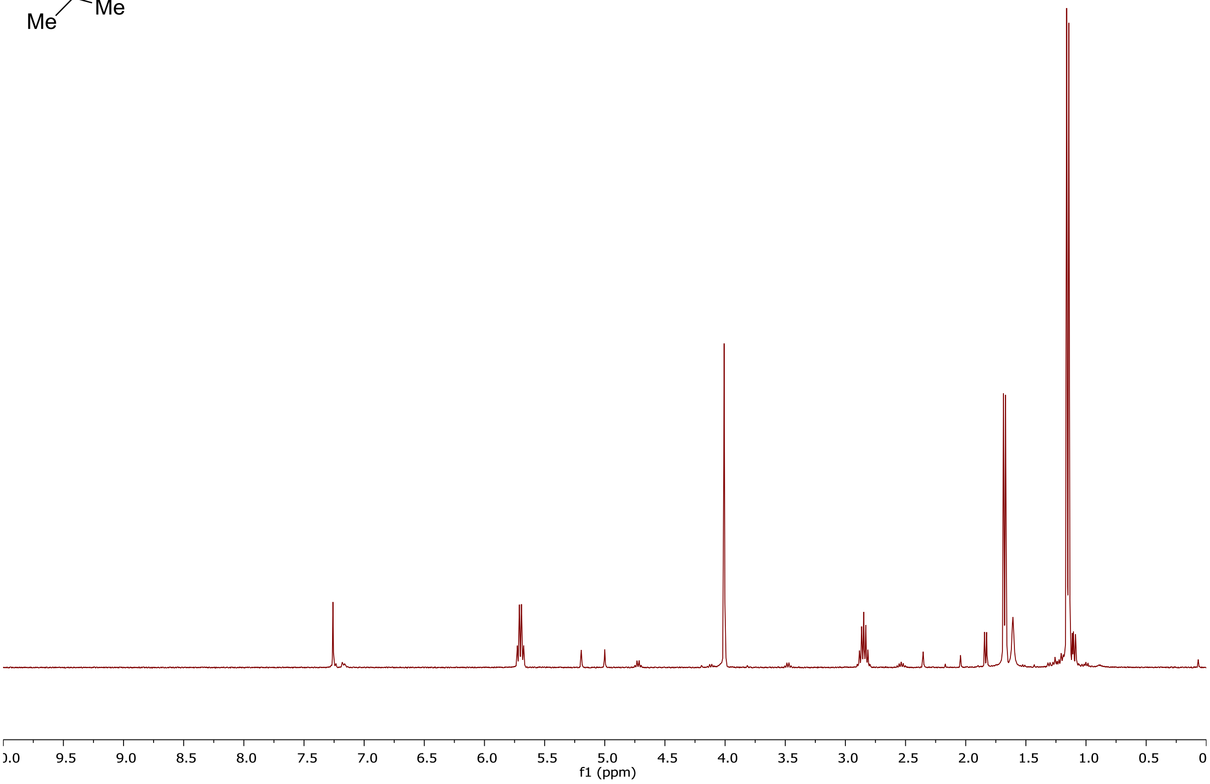
f1 (ppm)

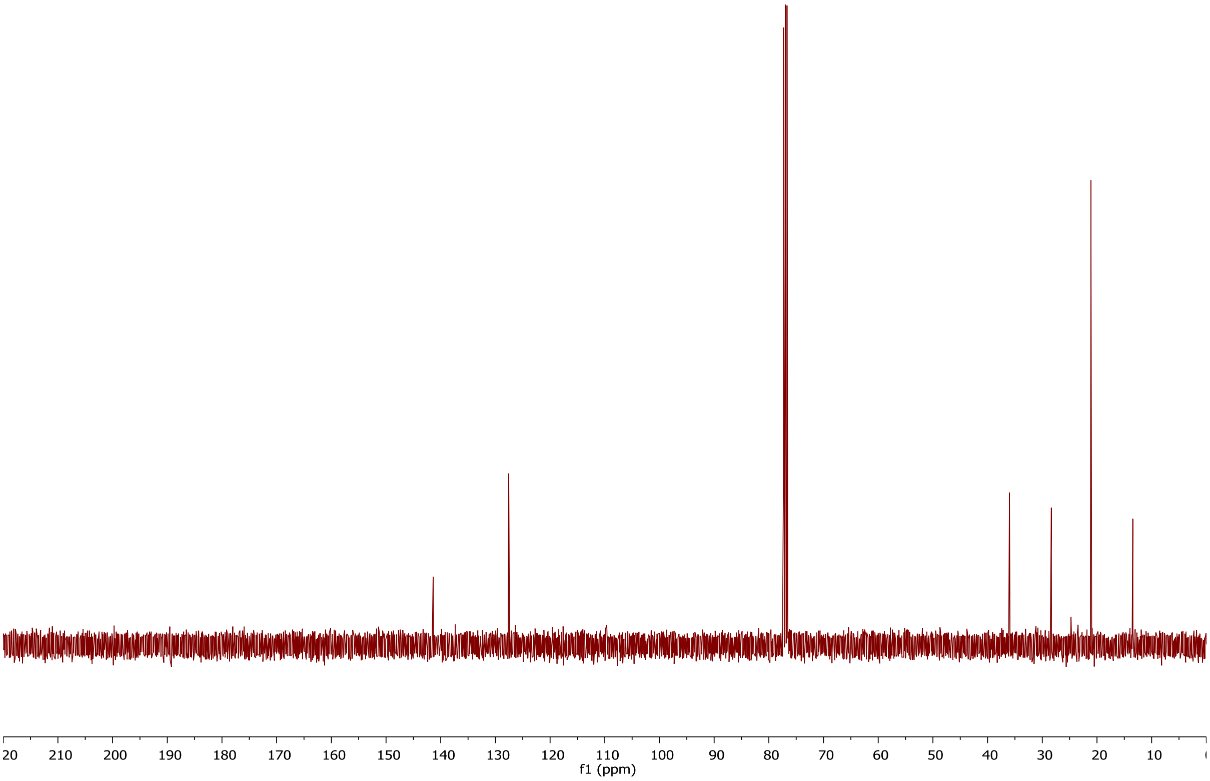
f1 (ppm)

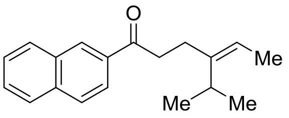
O
Me
Me
Me

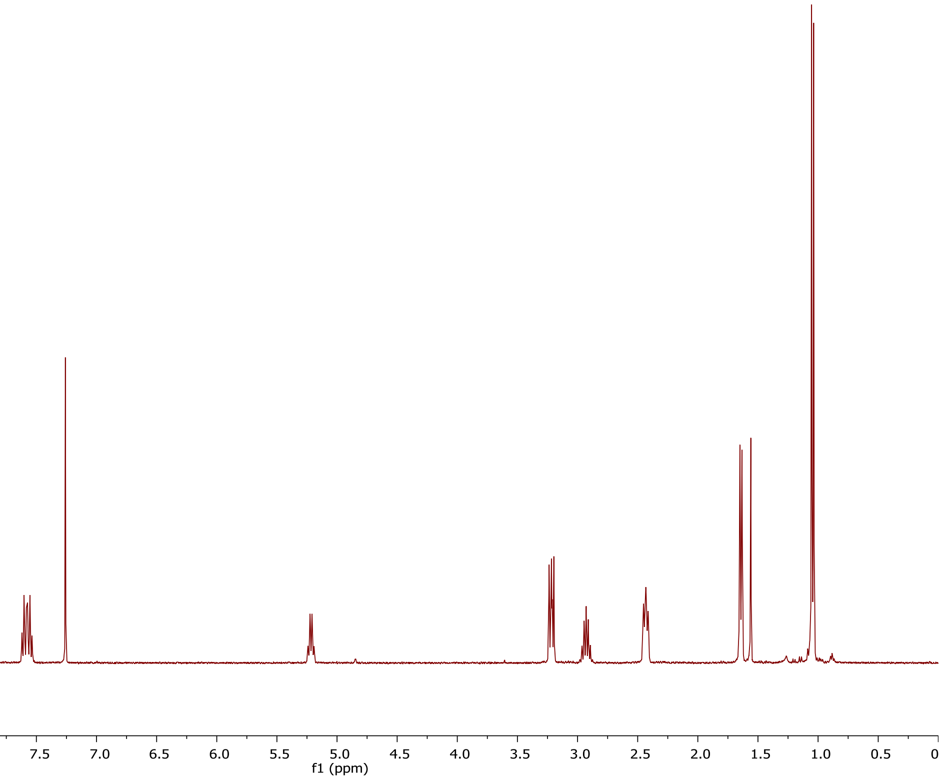
10.0 9.5 9.0 8.5 8.0 7.5 7.0 6.5 6.0 5.5 5.0 4.5 4.0 3.5 3.0 2.5 2.0 1.5 1.0 0.5 0
f1 (ppm)

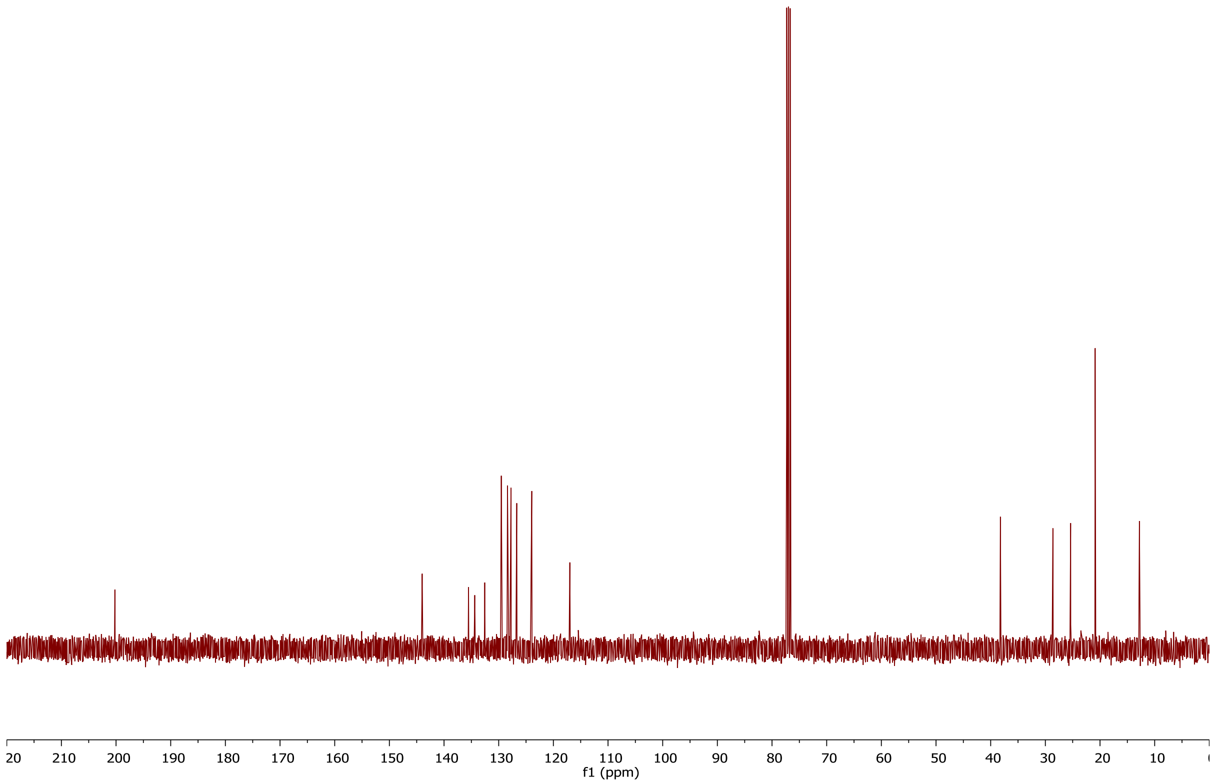
220 210 200 190 180 170 160 150 140 130 120 110 100 90 80 70 60 50 40 30 20 10 0
f1 (ppm)

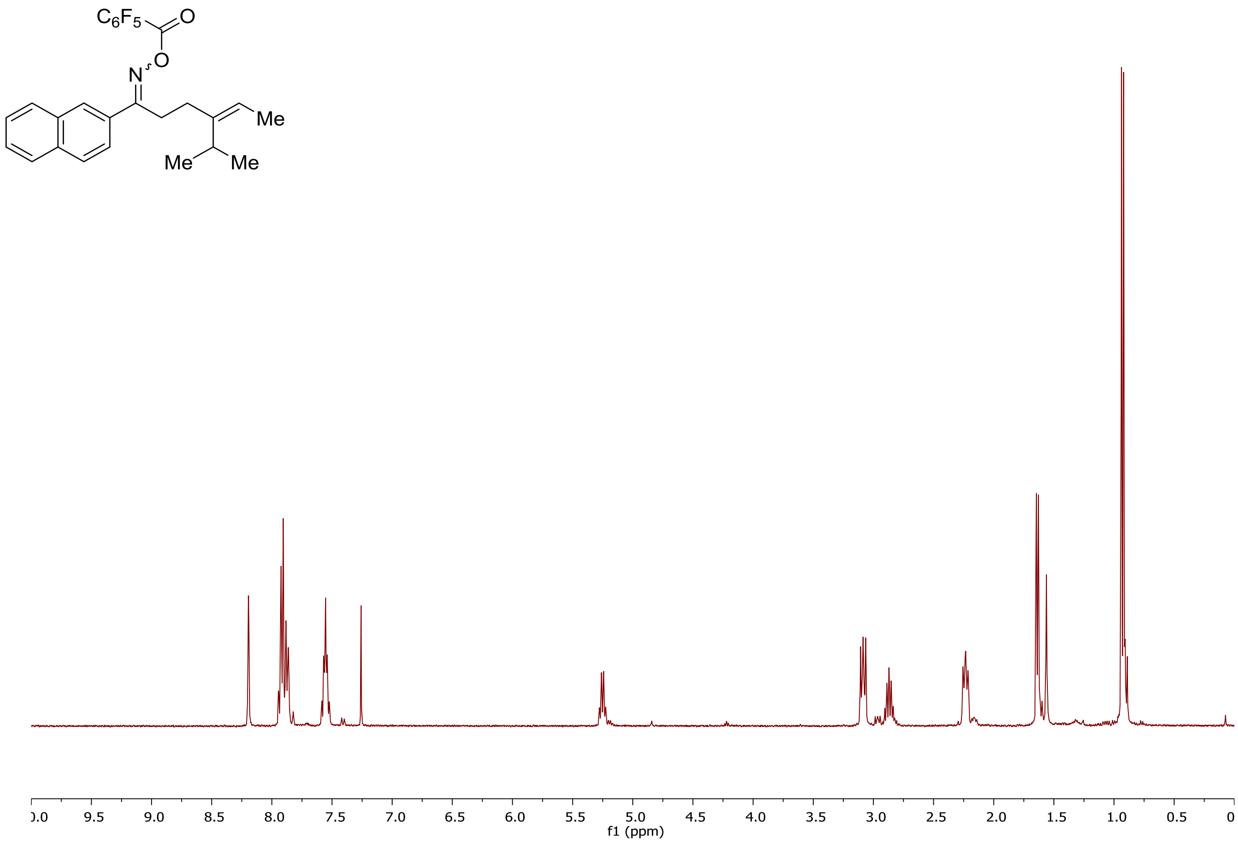
C_6F_5
O
N
O
Me
Me
Me
).0 9.5 9.0 8.5 8.0 7.5 7.0 6.5 6.0 5.5 5.0 4.5 4.0 3.5 3.0 2.5 2.0 1.5 1.0 0.5 0
f1 (ppm)

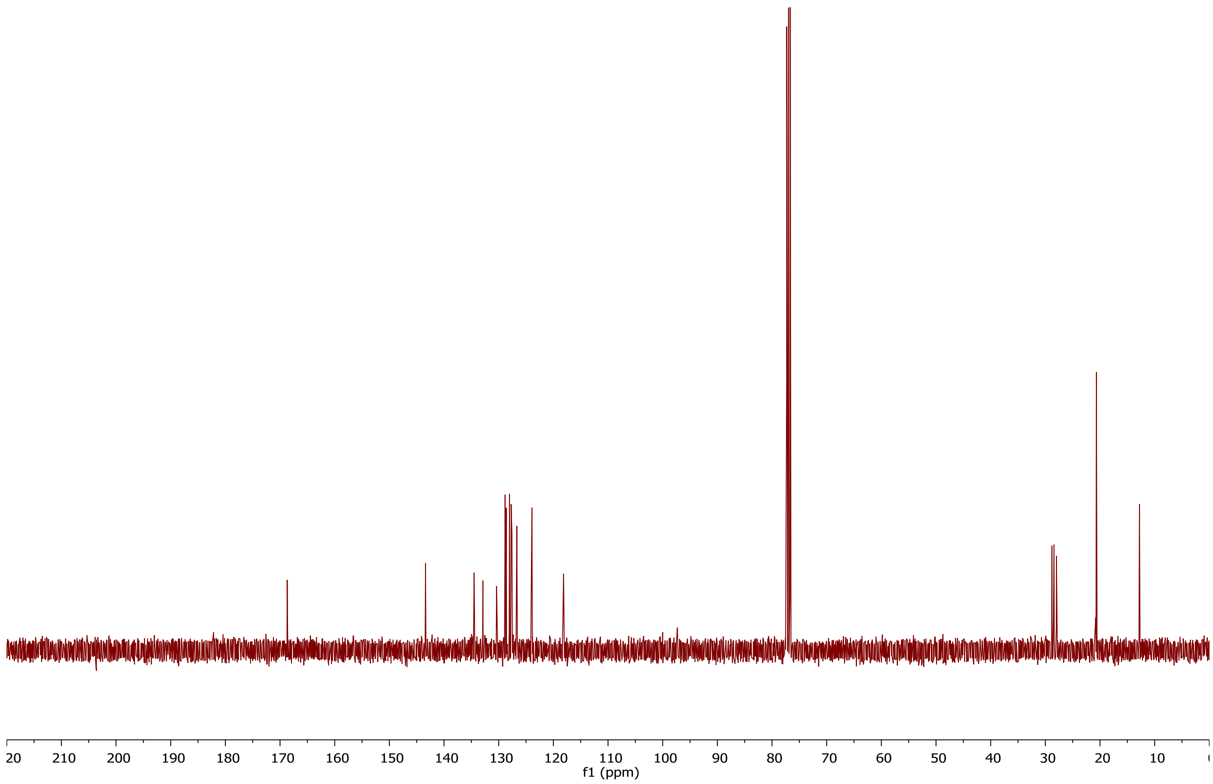
20 210 200 190 180 170 160 150 140 130 120 110 100 90 80 70 60 50 40 30 20 10
f1 (ppm)

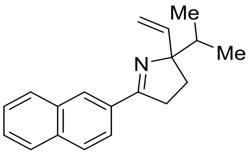
Me
N
Me

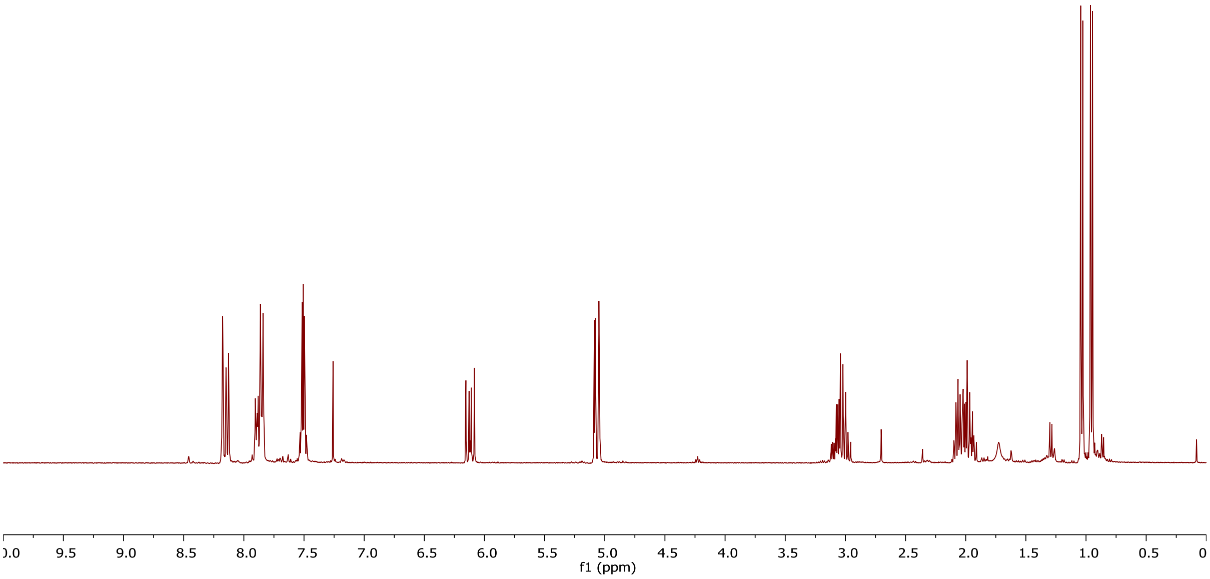
9.5 9.0 8.5 8.0 7.5 7.0 6.5 6.0 5.5 5.0 4.5 4.0 3.5 3.0 2.5 2.0 1.5 1.0 0.5
f1 (ppm)

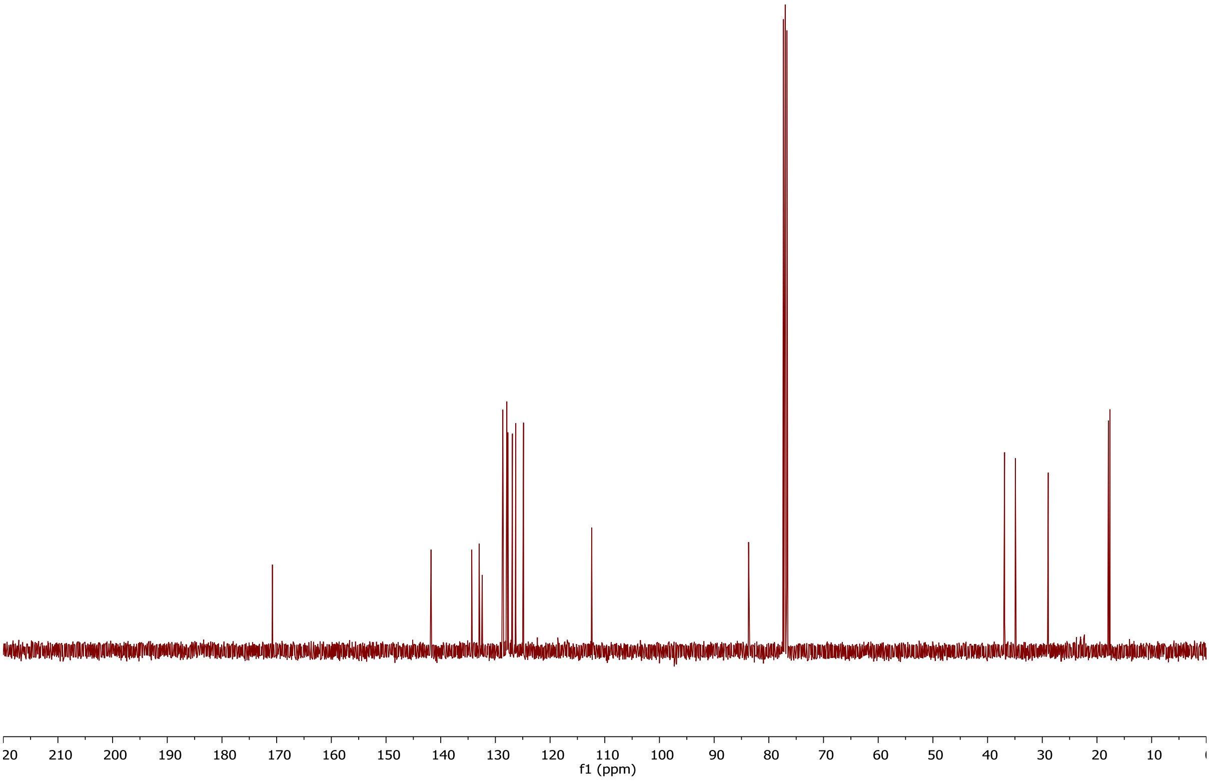
210 200 190 180 170 160 150 140 130 120 110 100 90 80 70 60 50 40 30 20 10
f1 (ppm)

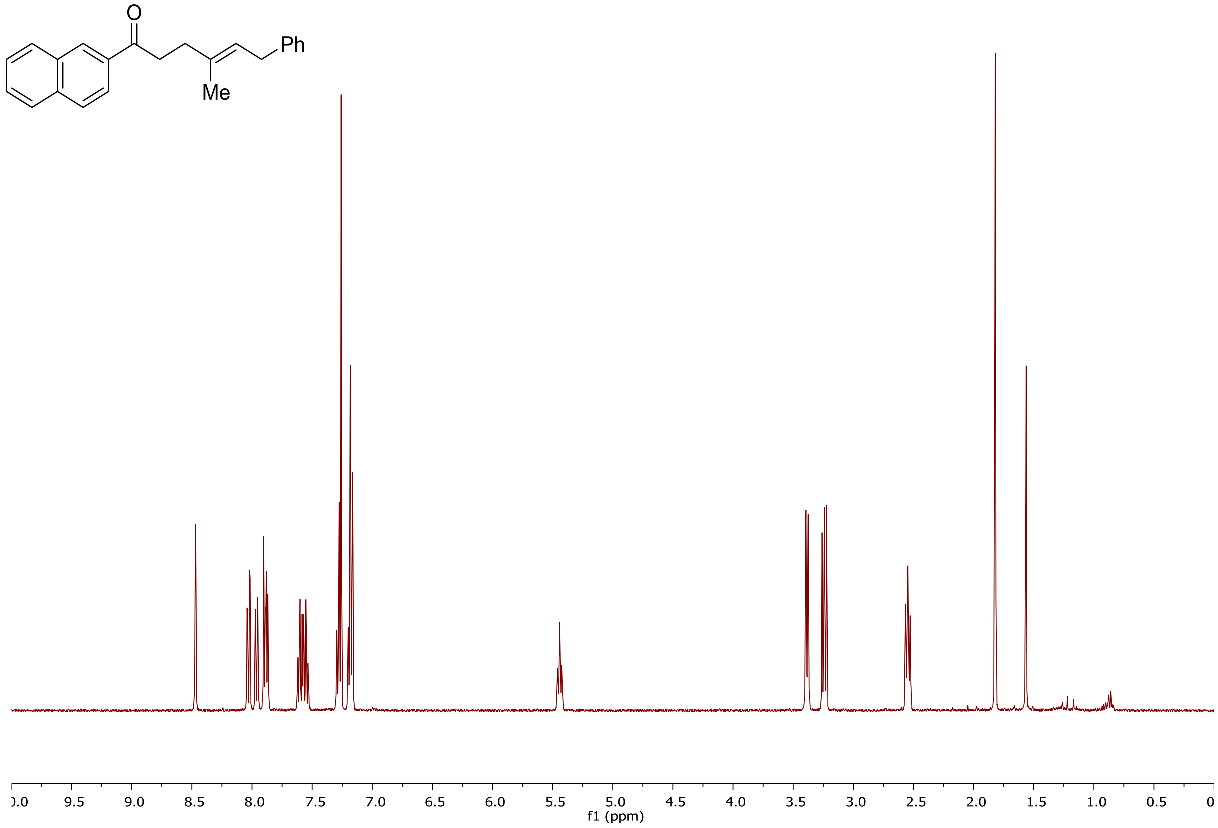
O
Ph
Me
9.5 9.0 8.5 8.0 7.5 7.0 6.5 6.0 5.5 5.0 4.5 4.0 3.5 3.0 2.5 2.0 1.5 1.0 0.5
f1 (ppm)

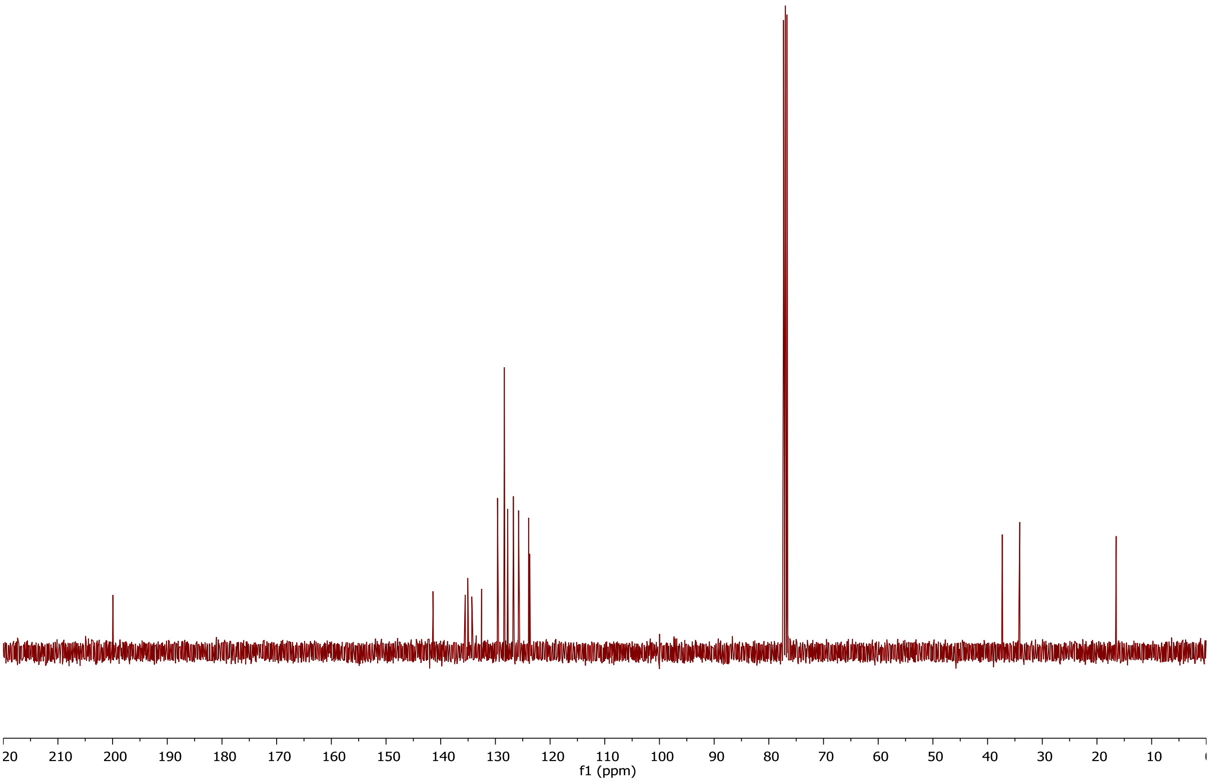
210 200 190 180 170 160 150 140 130 120 110 100 90 80 70 60 50 40 30 20 10
f1 (ppm)

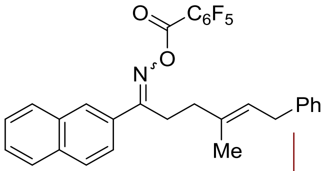
O
C6F5
N
O
Ph
Me

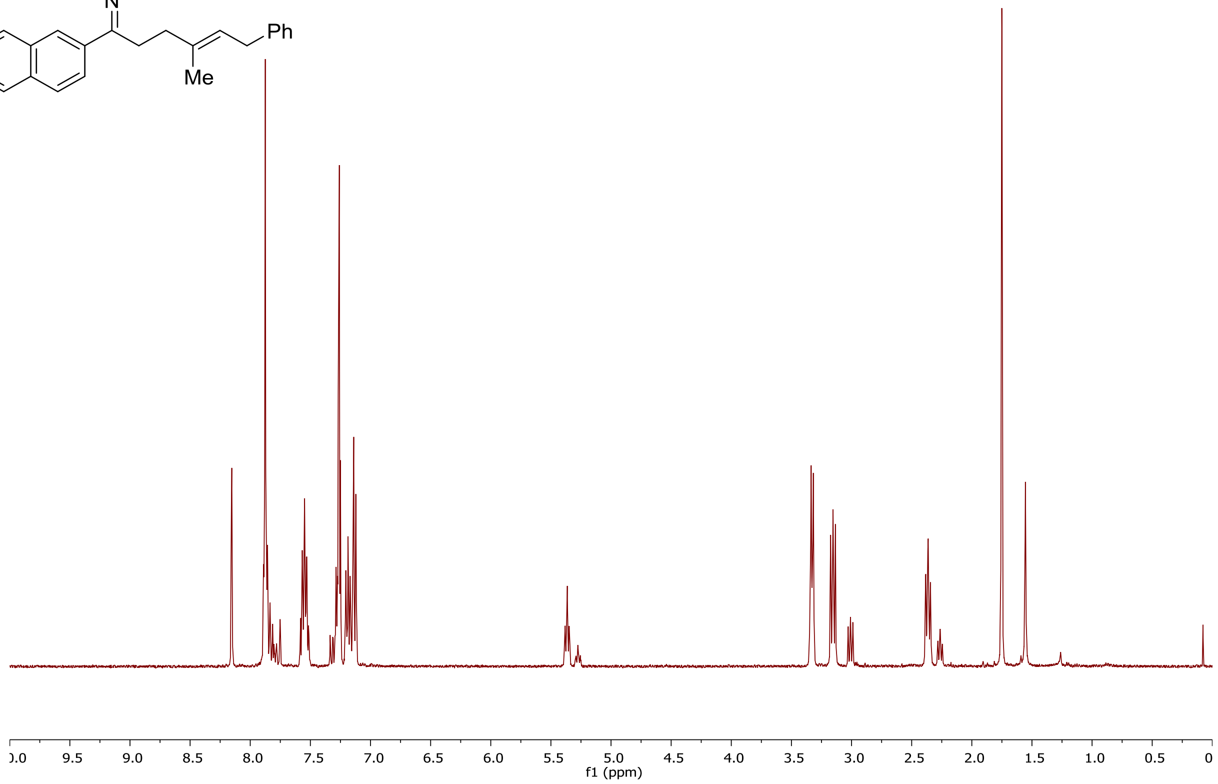
9.5 9.0 8.5 8.0 7.5 7.0 6.5 6.0 5.5 5.0 4.5 4.0 3.5 3.0 2.5 2.0 1.5 1.0 0.5
f1 (ppm)

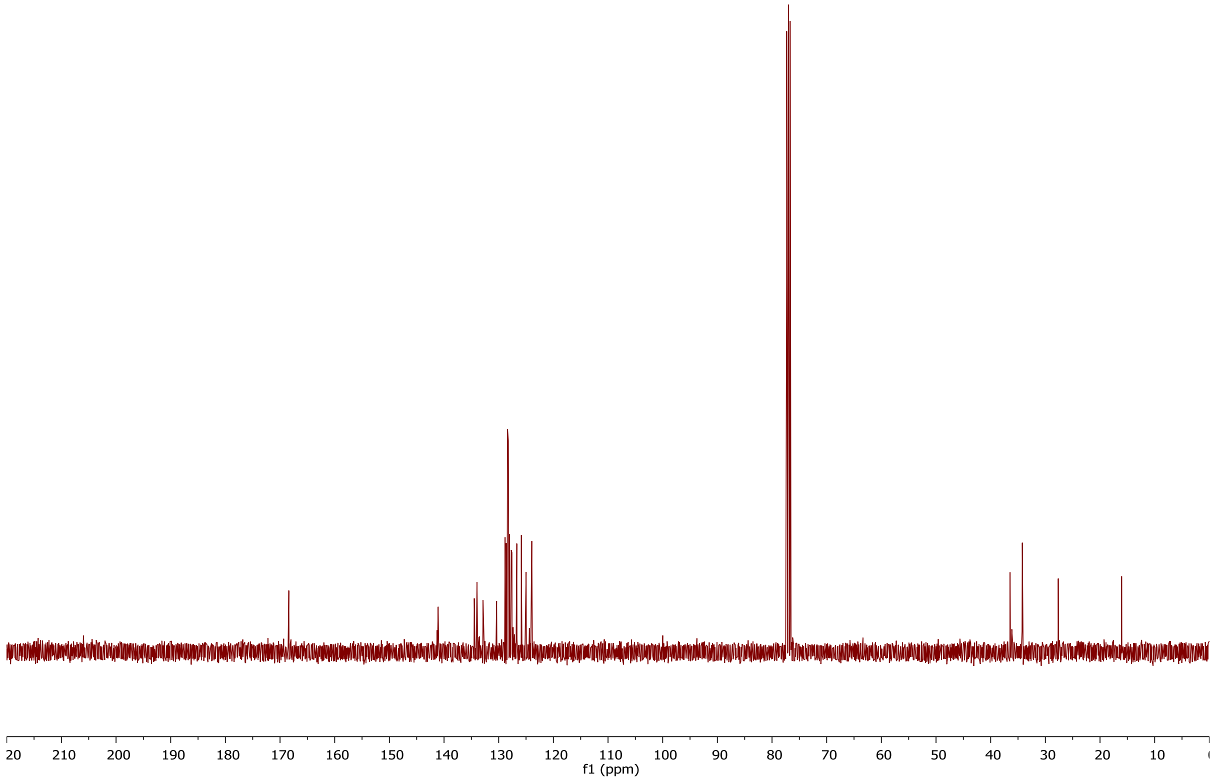
210 200 190 180 170 160 150 140 130 120 110 100 90 80 70 60 50 40 30 20 10
f1 (ppm)

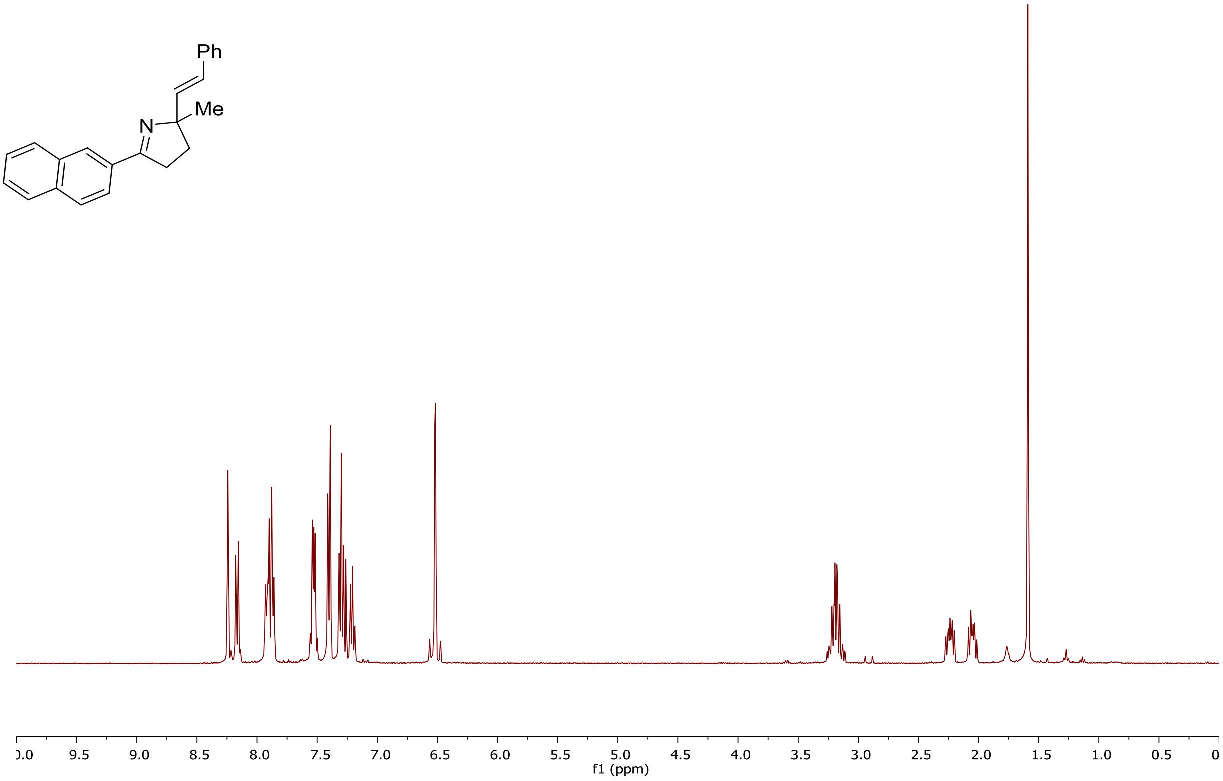
Ph
Me
N
9.5 9.0 8.5 8.0 7.5 7.0 6.5 6.0 5.5 5.0 4.5 4.0 3.5 3.0 2.5 2.0 1.5 1.0 0.5
f1 (ppm)

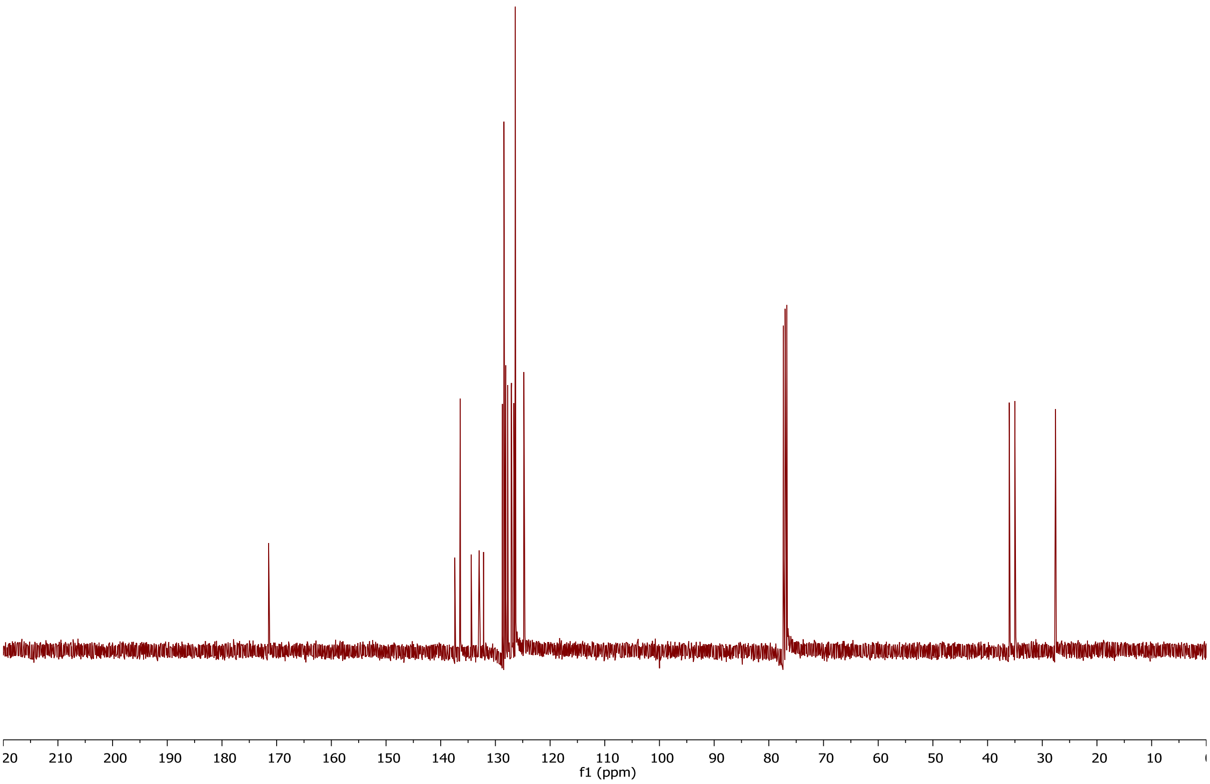
210 200 190 180 170 160 150 140 130 120 110 100 90 80 70 60 50 40 30 20 10
f1 (ppm)

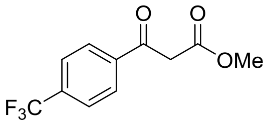
O
O
OMe
F3C

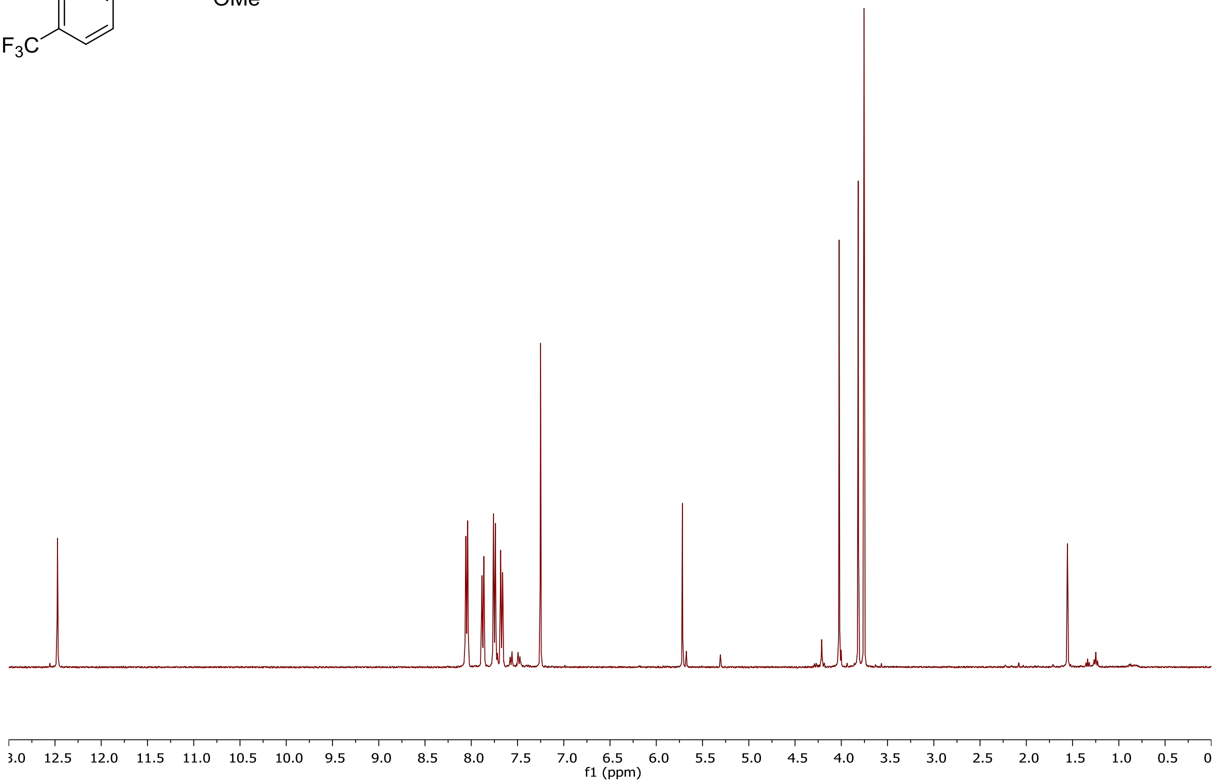
13.0 12.5 12.0 11.5 11.0 10.5 10.0 9.5 9.0 8.5 8.0 7.5 7.0 6.5 6.0 5.5 5.0 4.5 4.0 3.5 3.0 2.5 2.0 1.5 1.0 0.5 0
f1 (ppm)

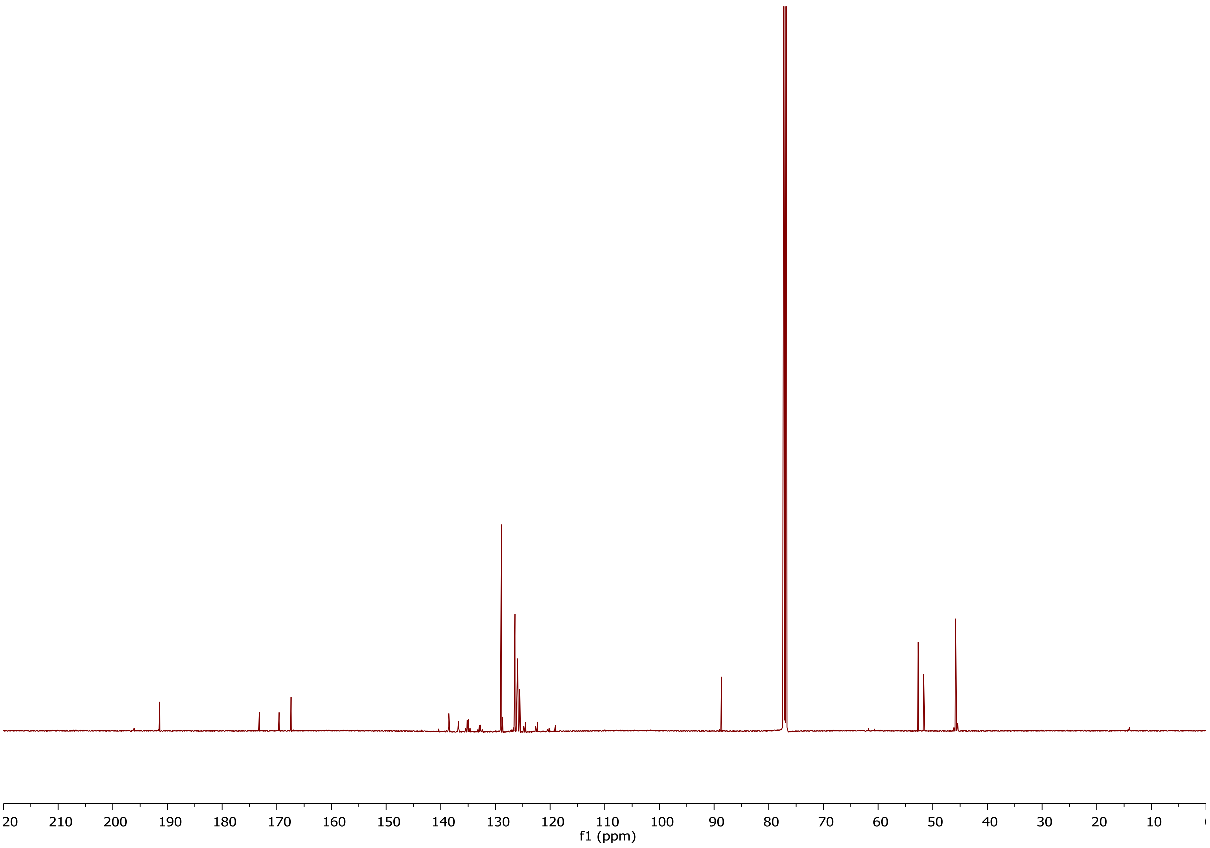
220 210 200 190 180 170 160 150 140 130 120 110 100 90 80 70 60 50 40 30 20 10 0
f1 (ppm)

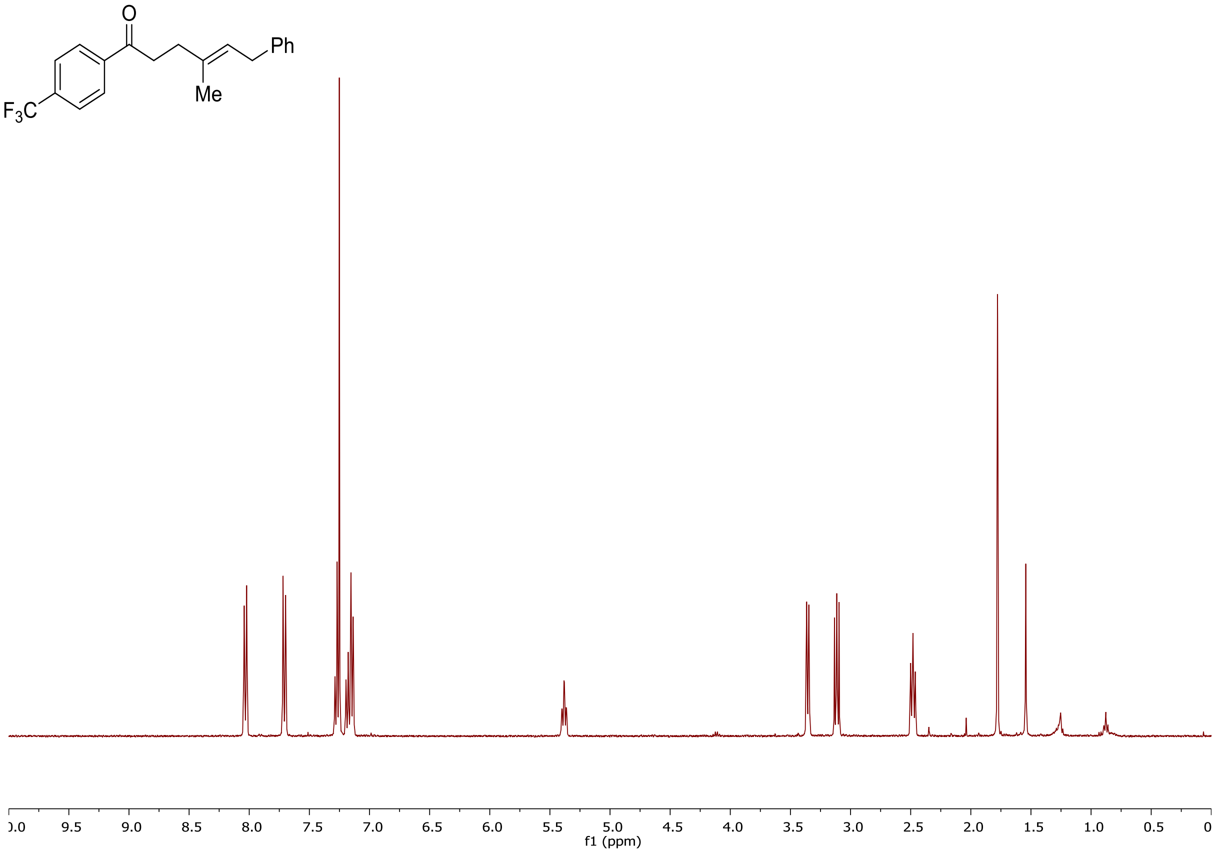
O
Ph
Me
F_3C
9.5 9.0 8.5 8.0 7.5 7.0 6.5 6.0 5.5 5.0 4.5 4.0 3.5 3.0 2.5 2.0 1.5 1.0 0.5
f1 (ppm)

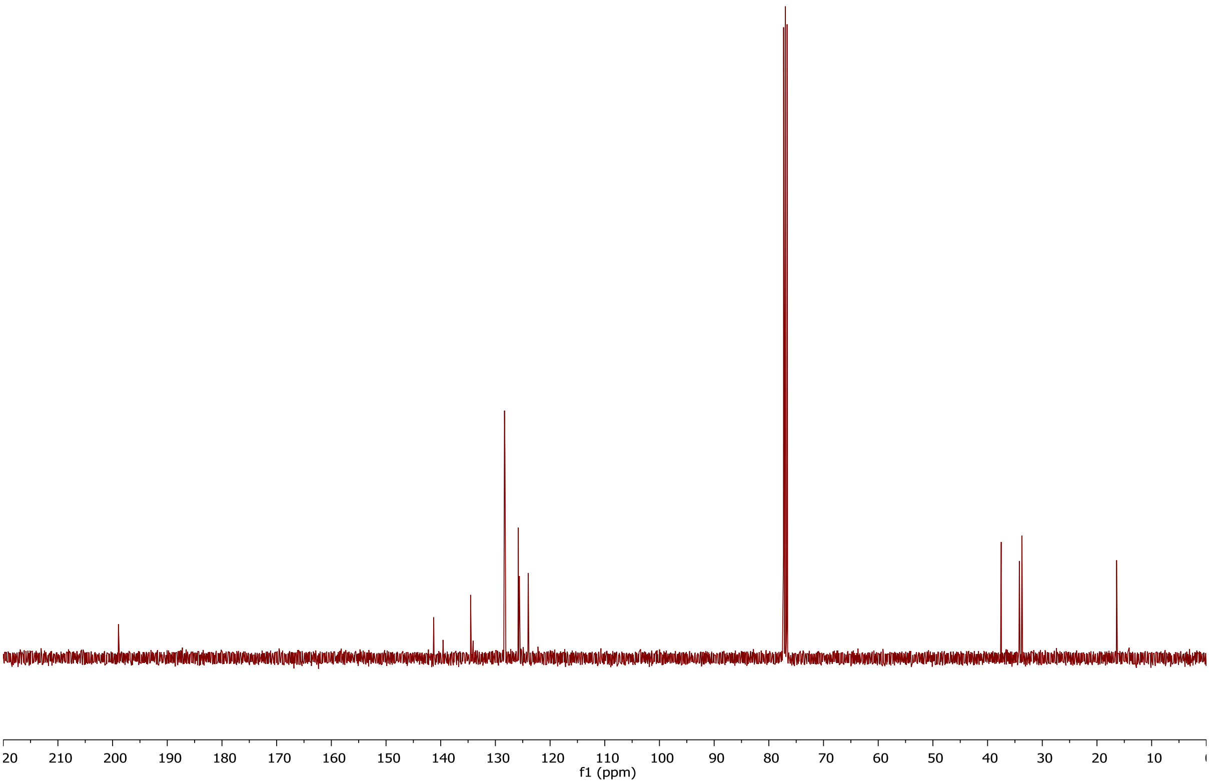
210 200 190 180 170 160 150 140 130 120 110 100 90 80 70 60 50 40 30 20 10
f1 (ppm)

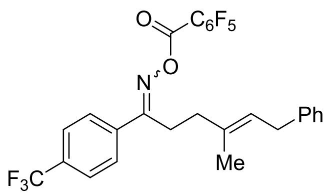
O
C6F5
O
N
Ph
Me
F3C

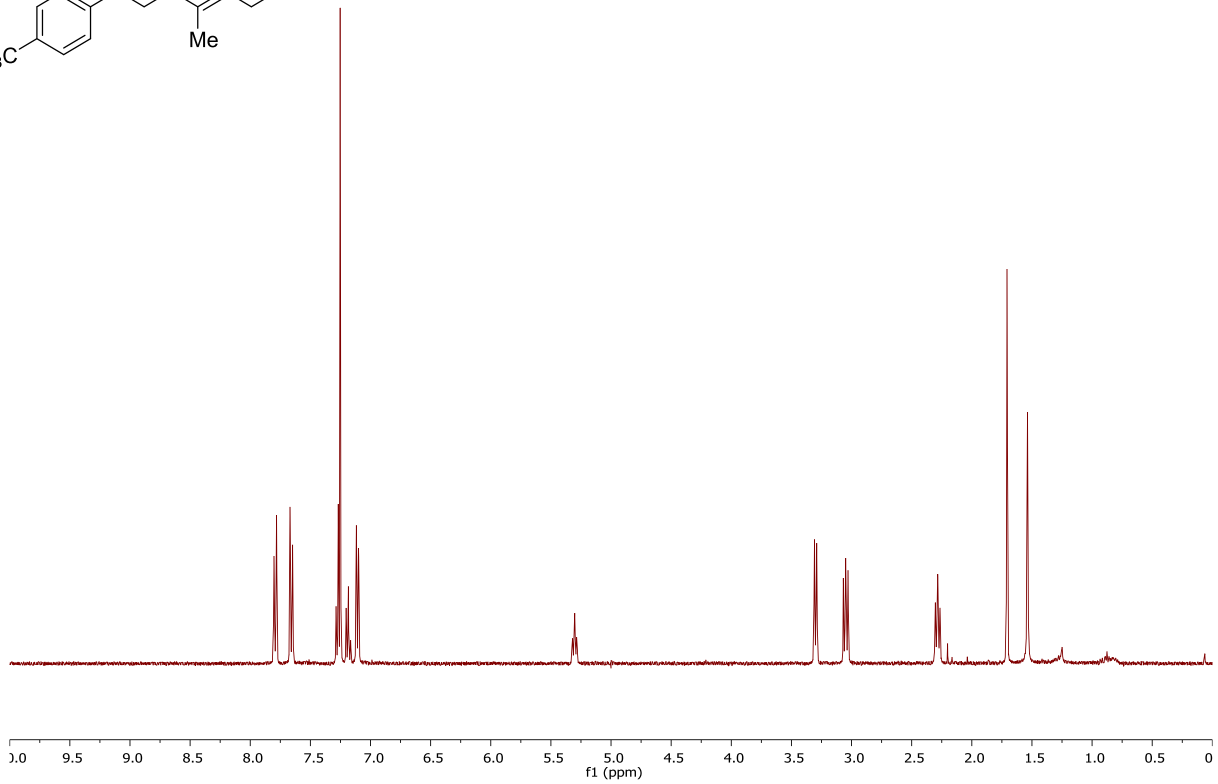
f1 (ppm)

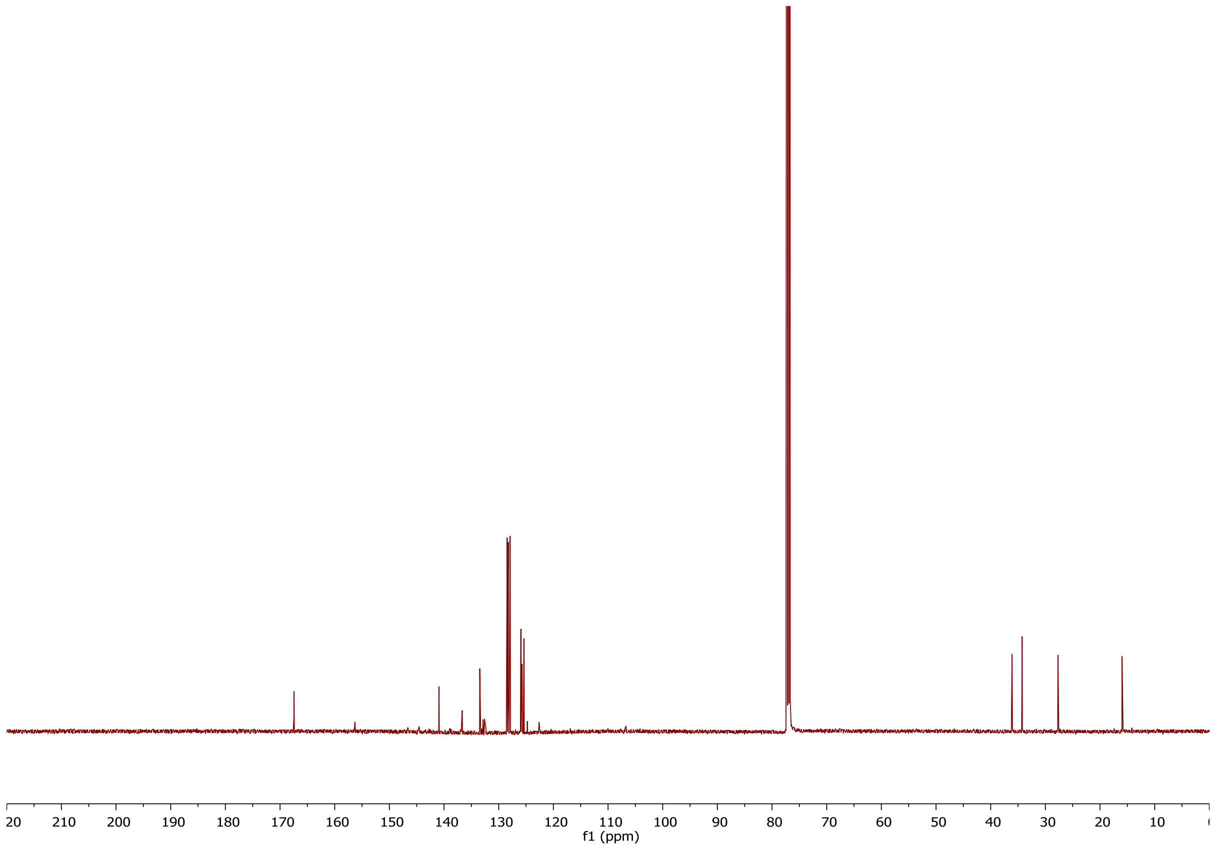
f1 (ppm)

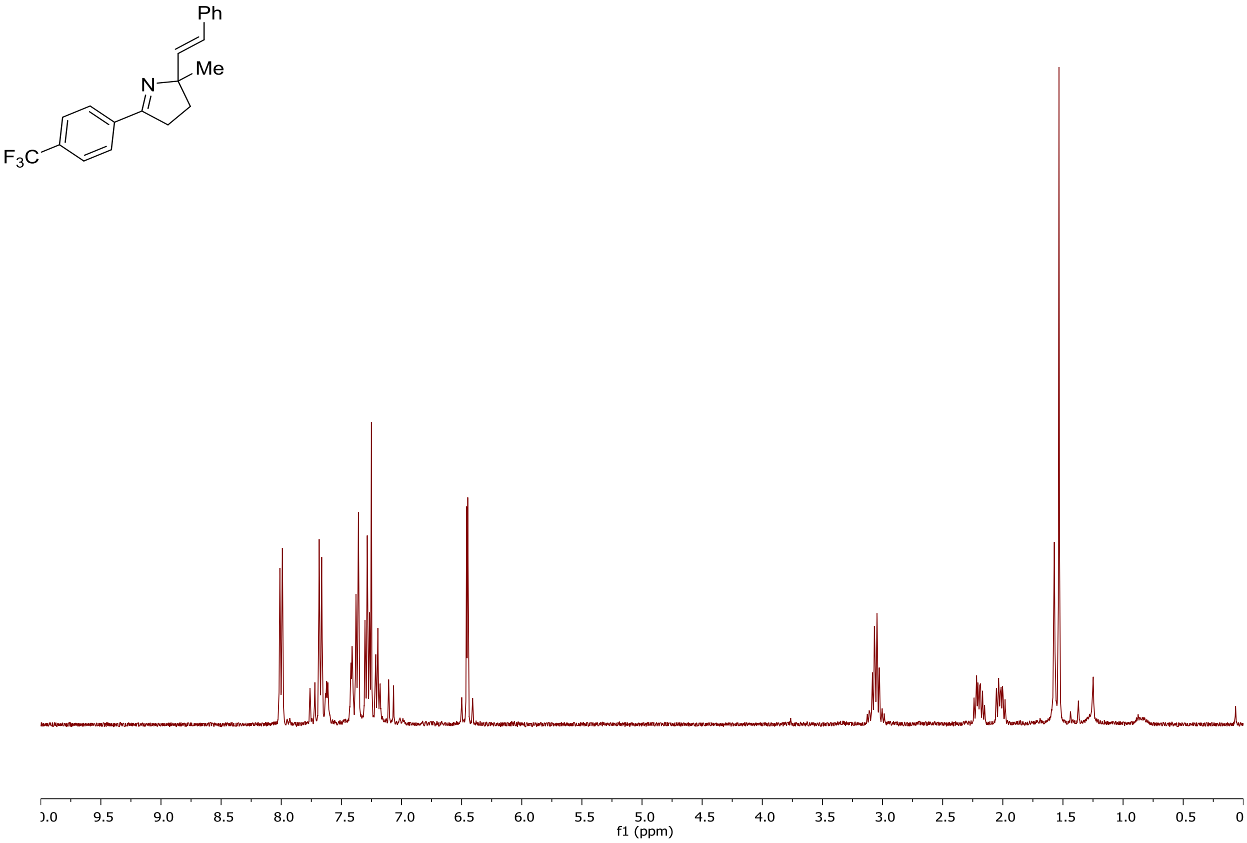
Ph
Me
N
F3C
0.0 9.5 9.0 8.5 8.0 7.5 7.0 6.5 6.0 5.5 5.0 4.5 4.0 3.5 3.0 2.5 2.0 1.5 1.0 0.5 0
f1 (ppm)

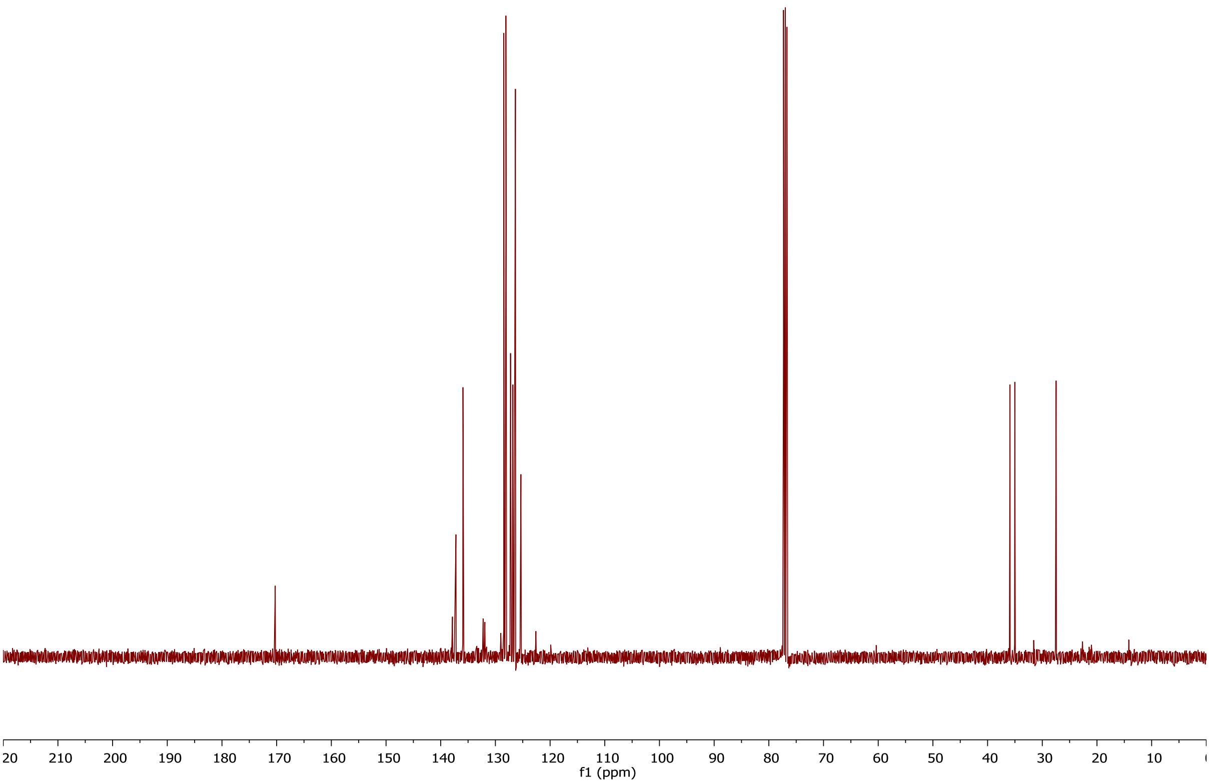
20 210 200 190 180 170 160 150 140 130 120 110 100 90 80 70 60 50 40 30 20 10
f1 (ppm)

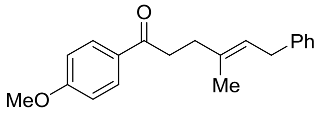
O
Ph
MeO
Me

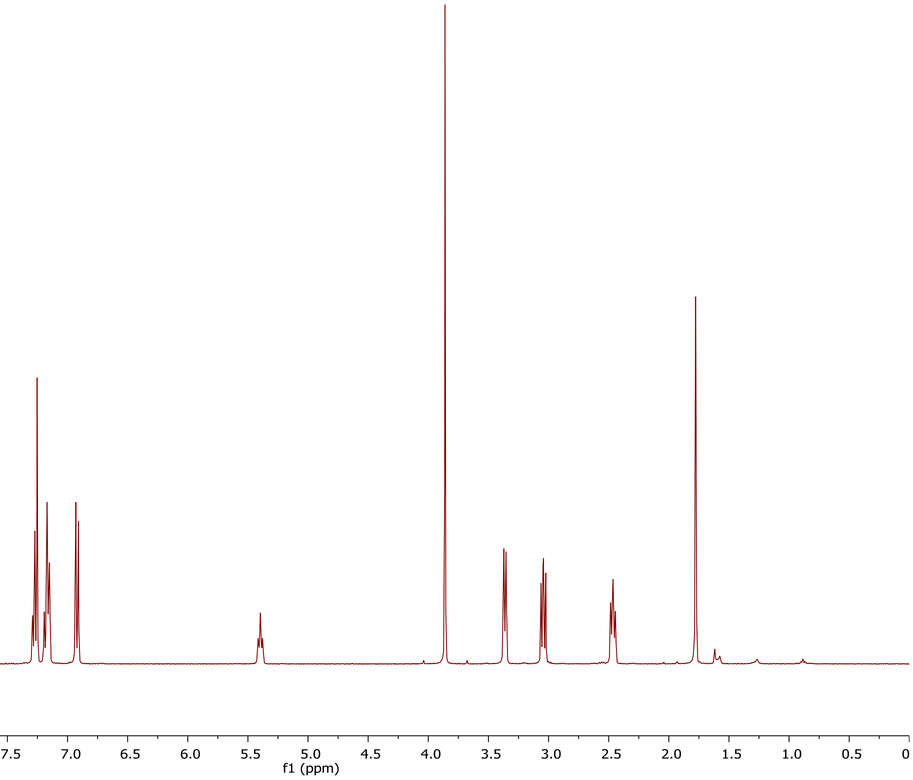
9.5 9.0 8.5 8.0 7.5 7.0 6.5 6.0 5.5 5.0 4.5 4.0 3.5 3.0 2.5 2.0 1.5 1.0 0.5 0
f1 (ppm)

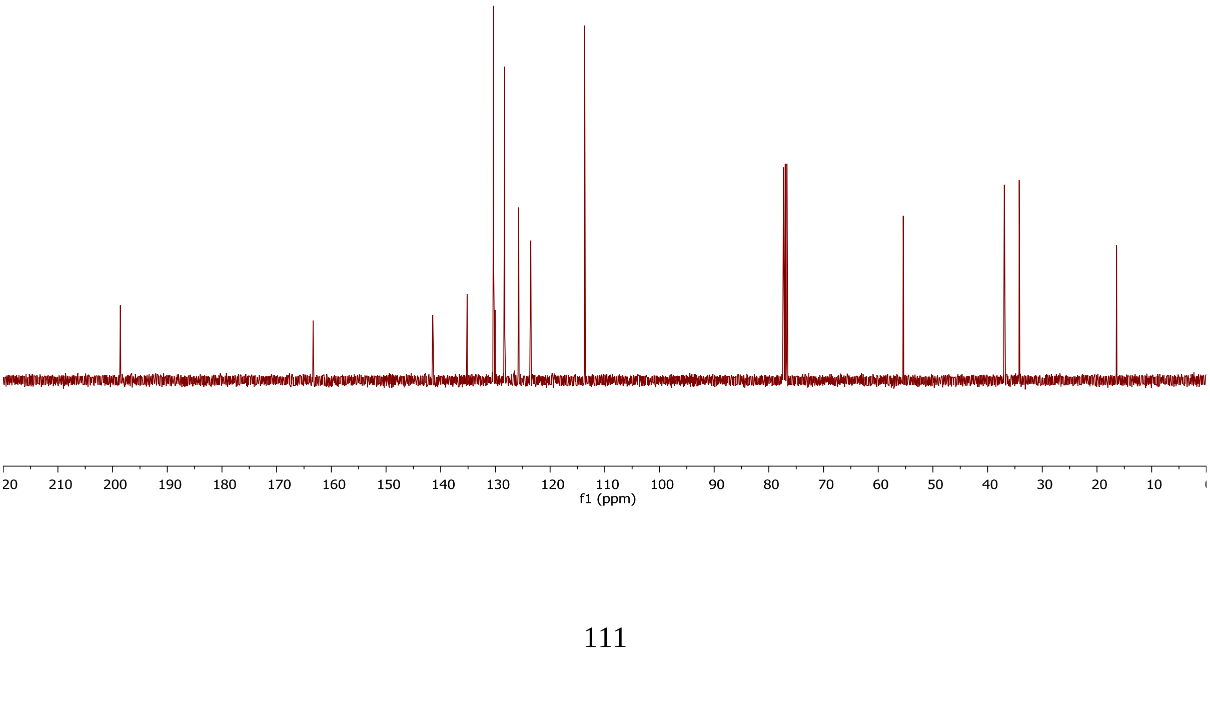
20 210 200 190 180 170 160 150 140 130 120 110 100 90 80 70 60 50 40 30 20 10
f1 (ppm)

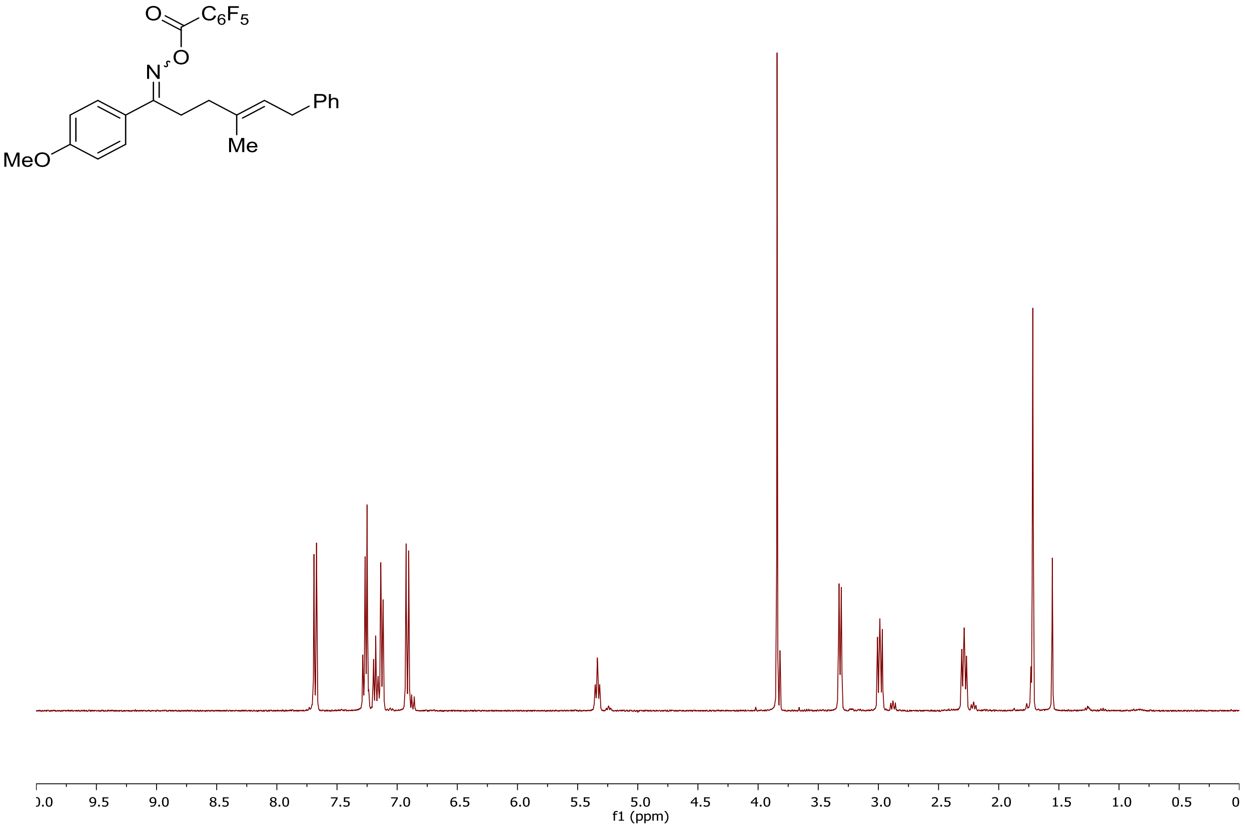

O
C6F5
N
O
Ph
MeO
Me
f1 (ppm)

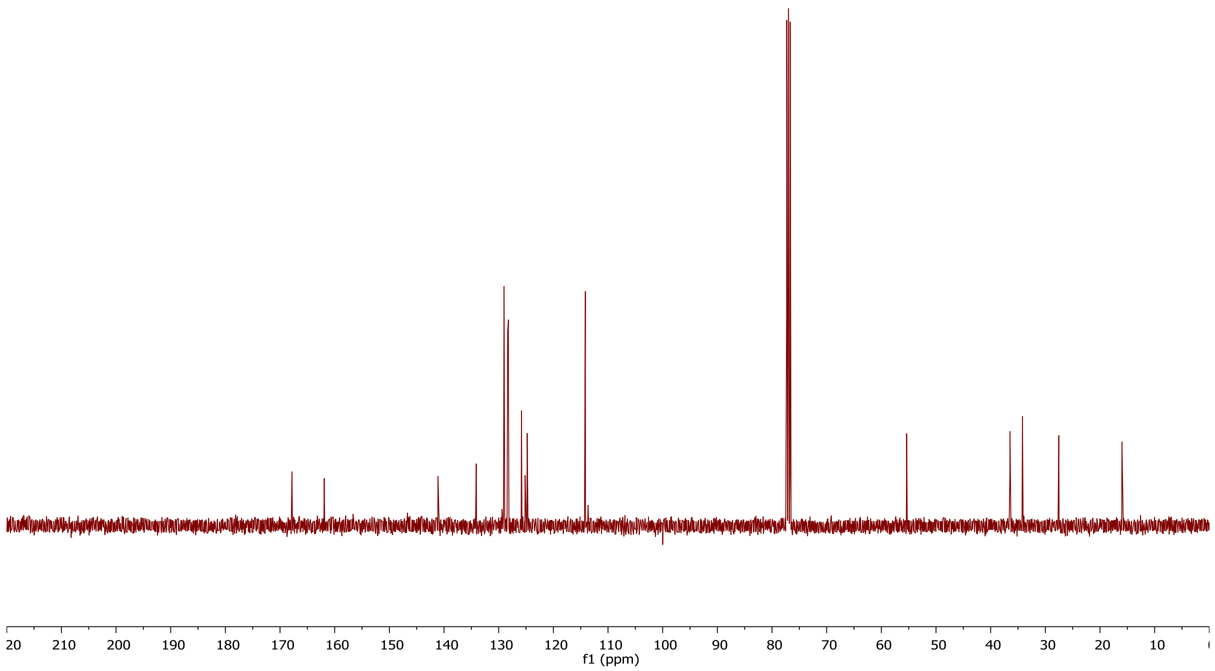

f1 (ppm)

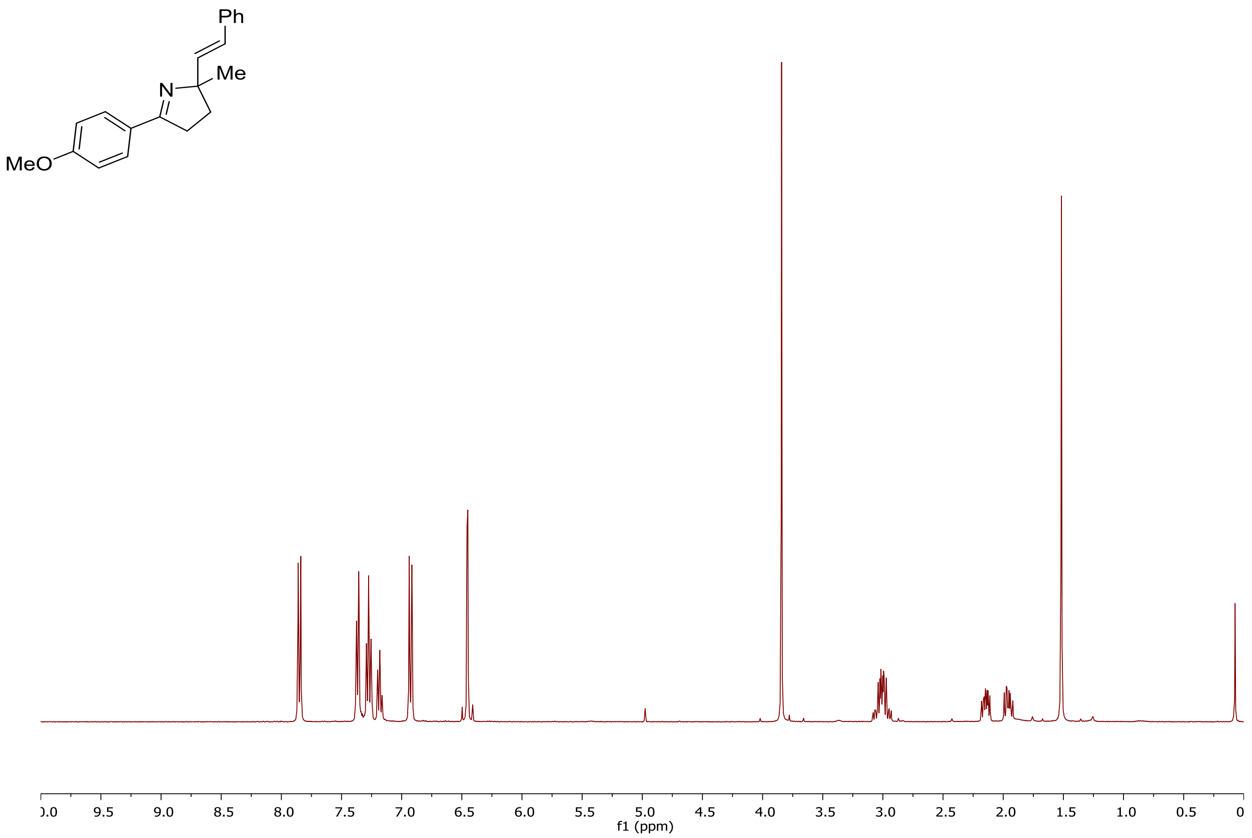
Ph
Me
N
MeO
f1 (ppm)

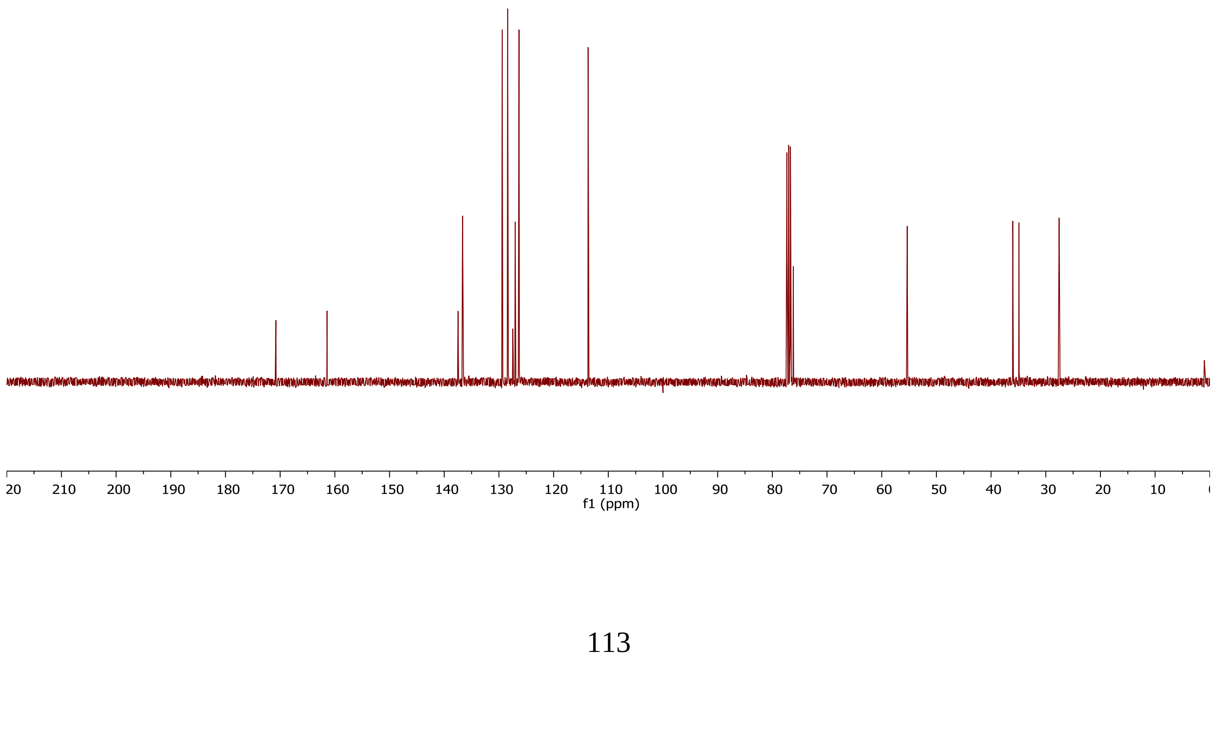
f1 (ppm)

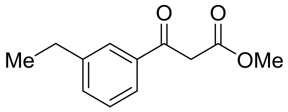
O
O
Me
OMe

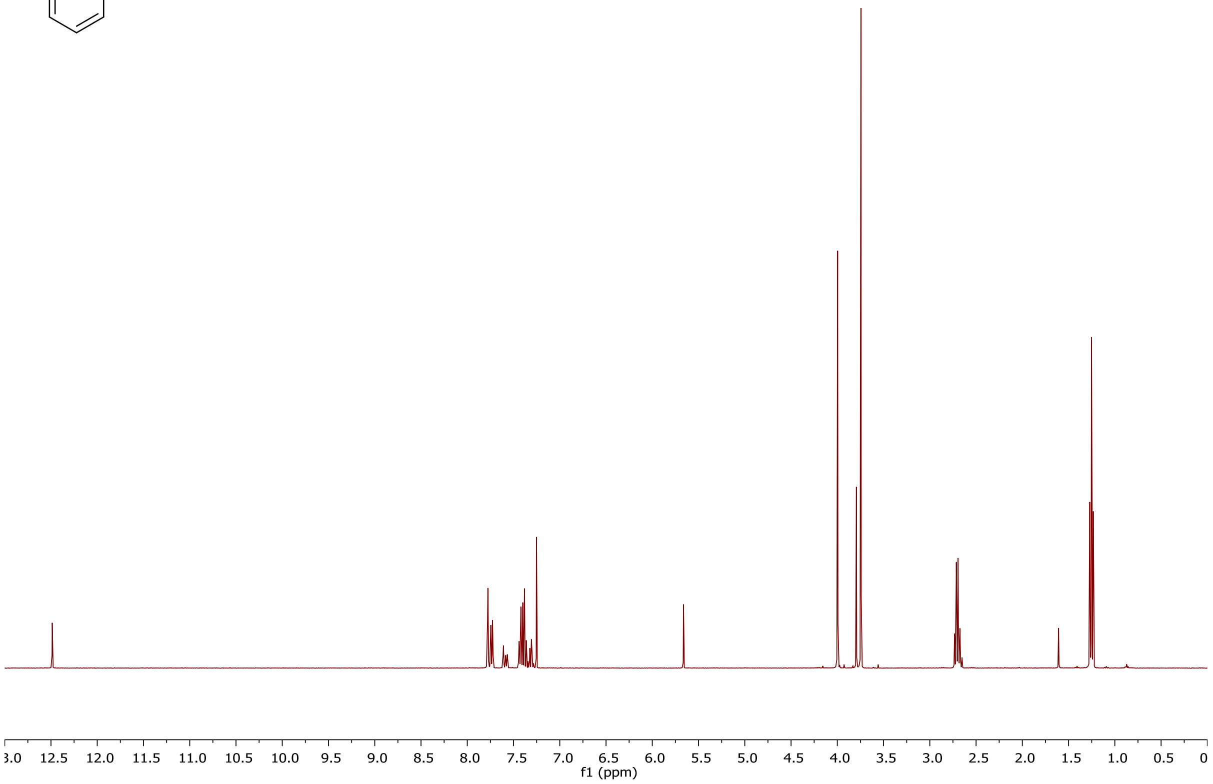
f1 (ppm)

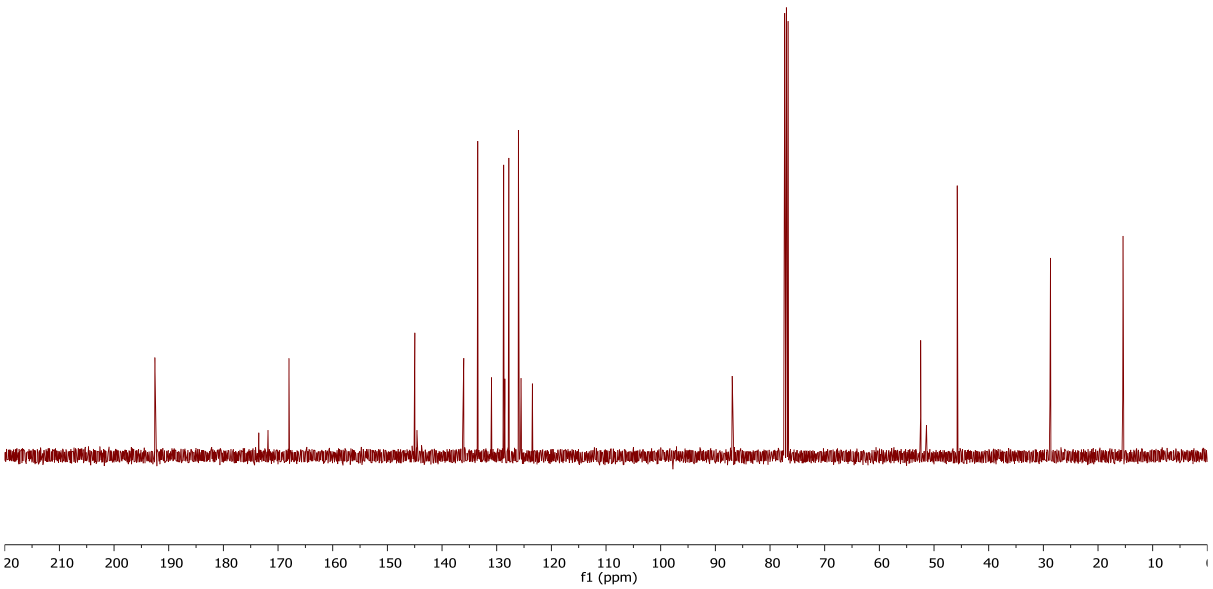
f1 (ppm)

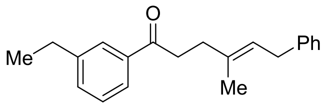
O
Me
Ph
Me

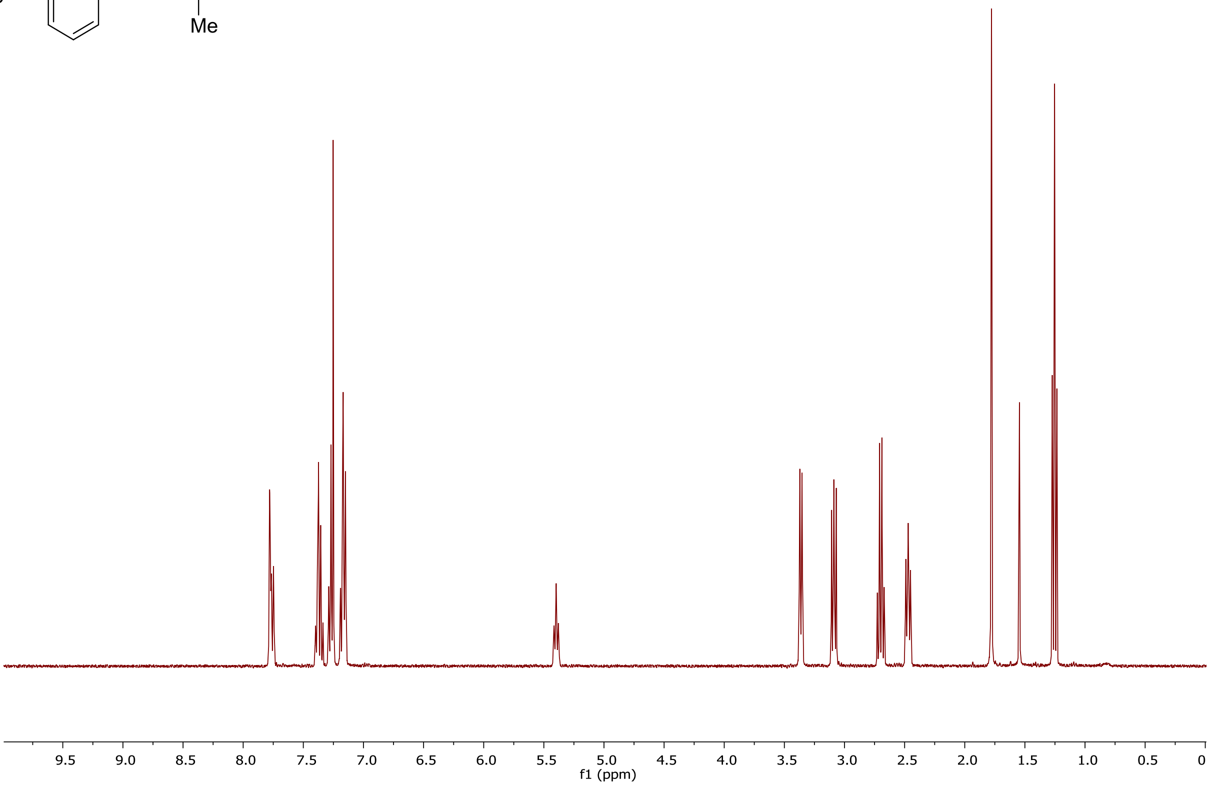
9.5 9.0 8.5 8.0 7.5 7.0 6.5 6.0 5.5 5.0 4.5 4.0 3.5 3.0 2.5 2.0 1.5 1.0 0.5 0
f1 (ppm)

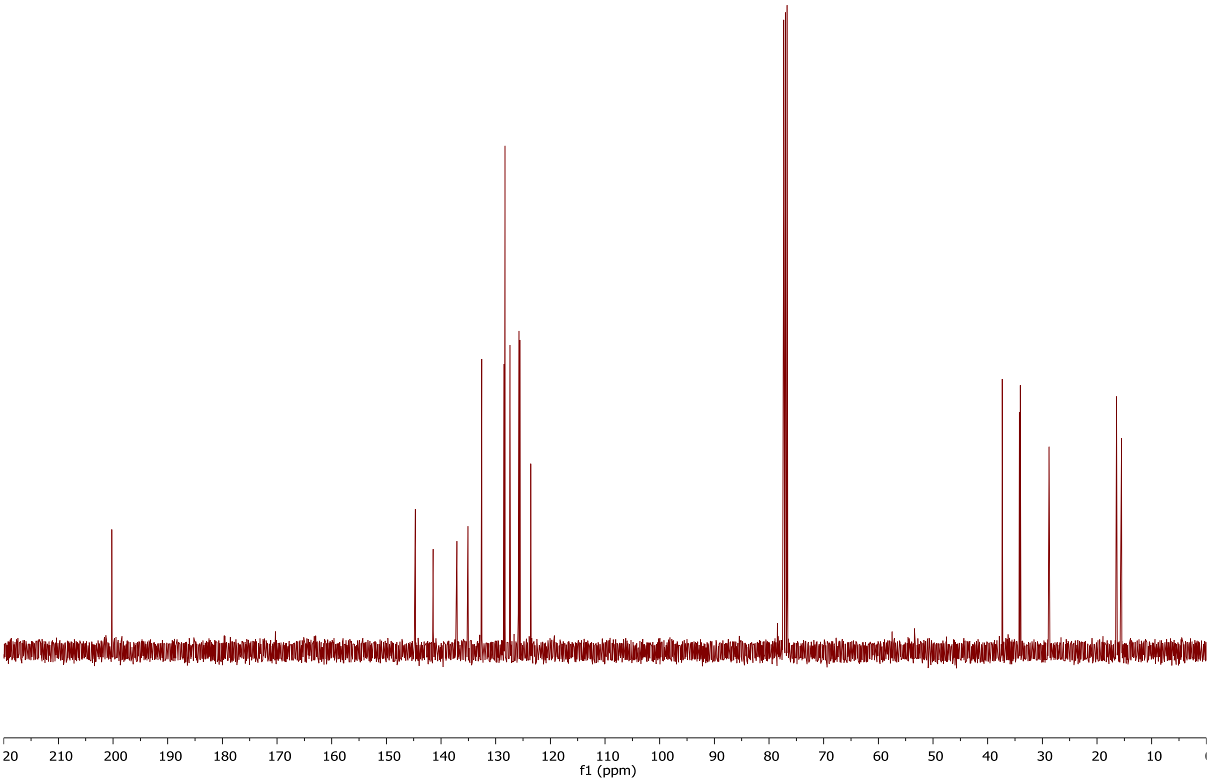
20 210 200 190 180 170 160 150 140 130 120 110 100 90 80 70 60 50 40 30 20 10
f1 (ppm)

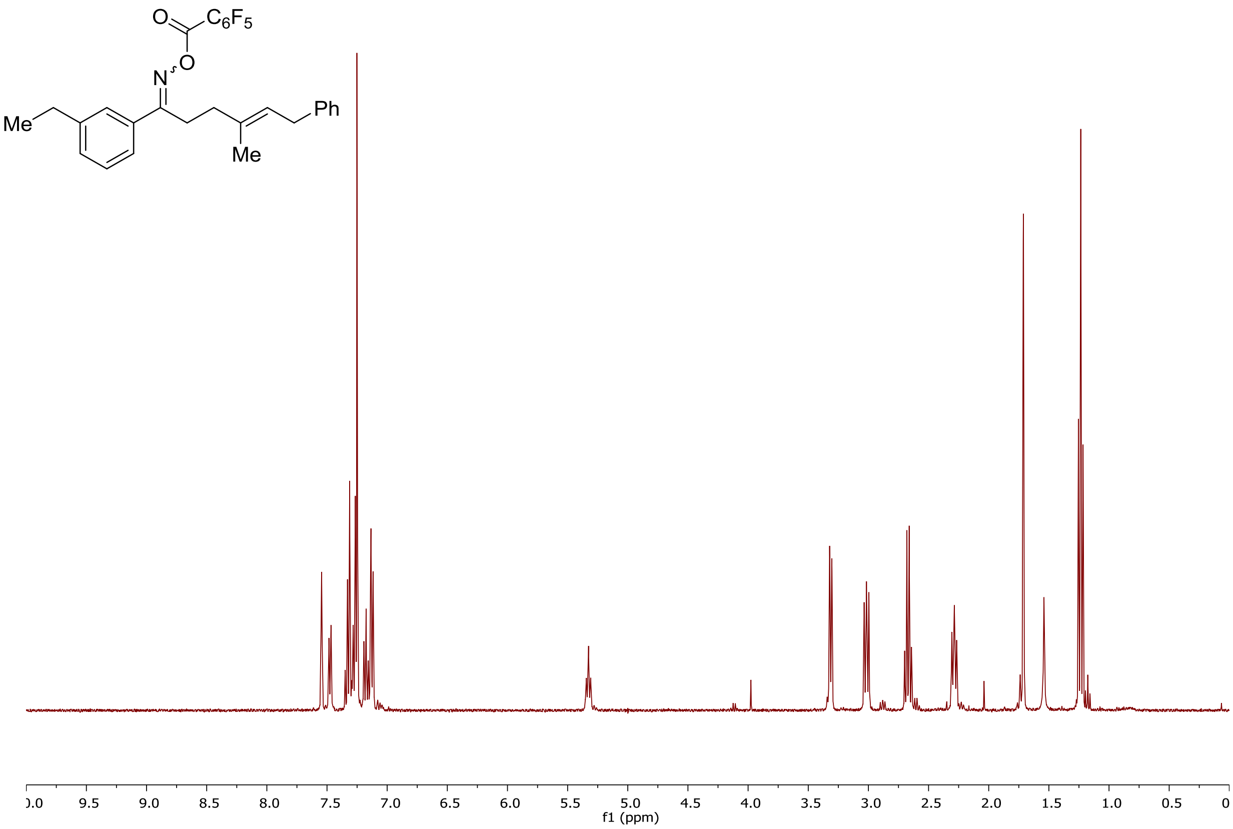
O
C6F5
O
N
Me
Ph
Me
f1 (ppm)

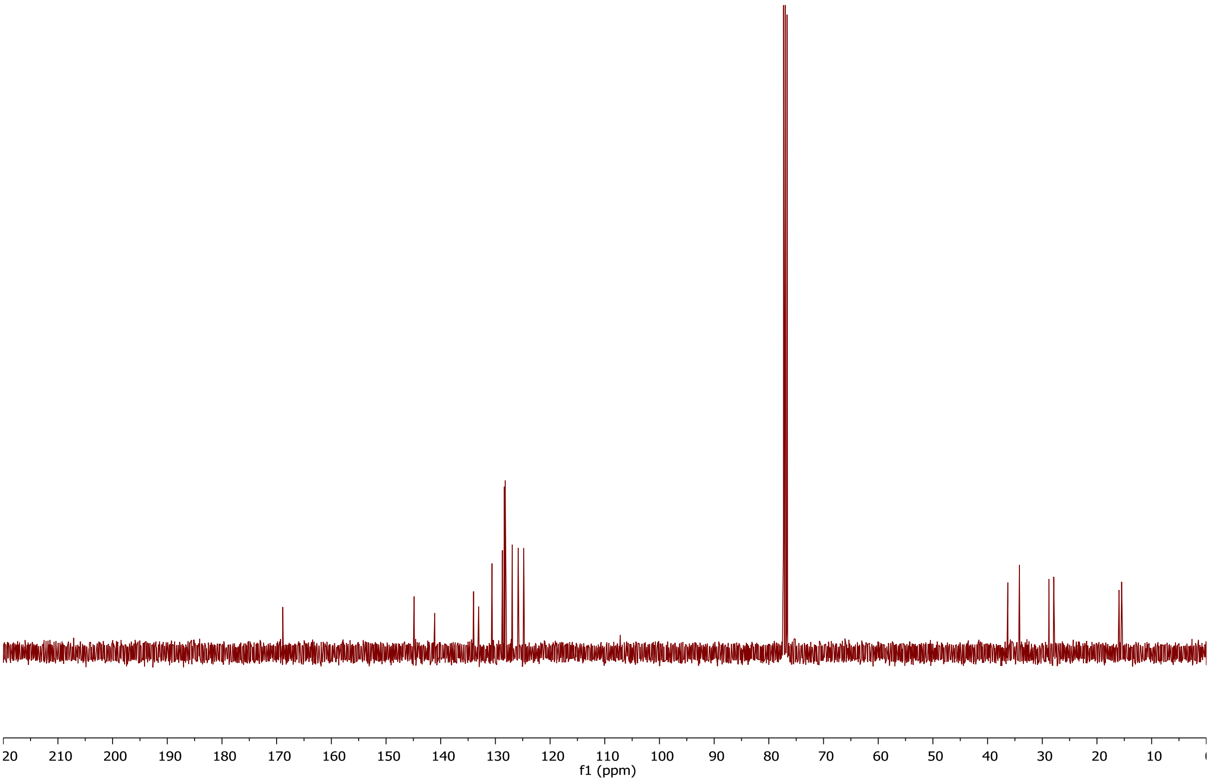
f1 (ppm)

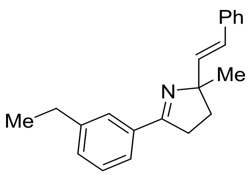
Ph
Me
N
Me

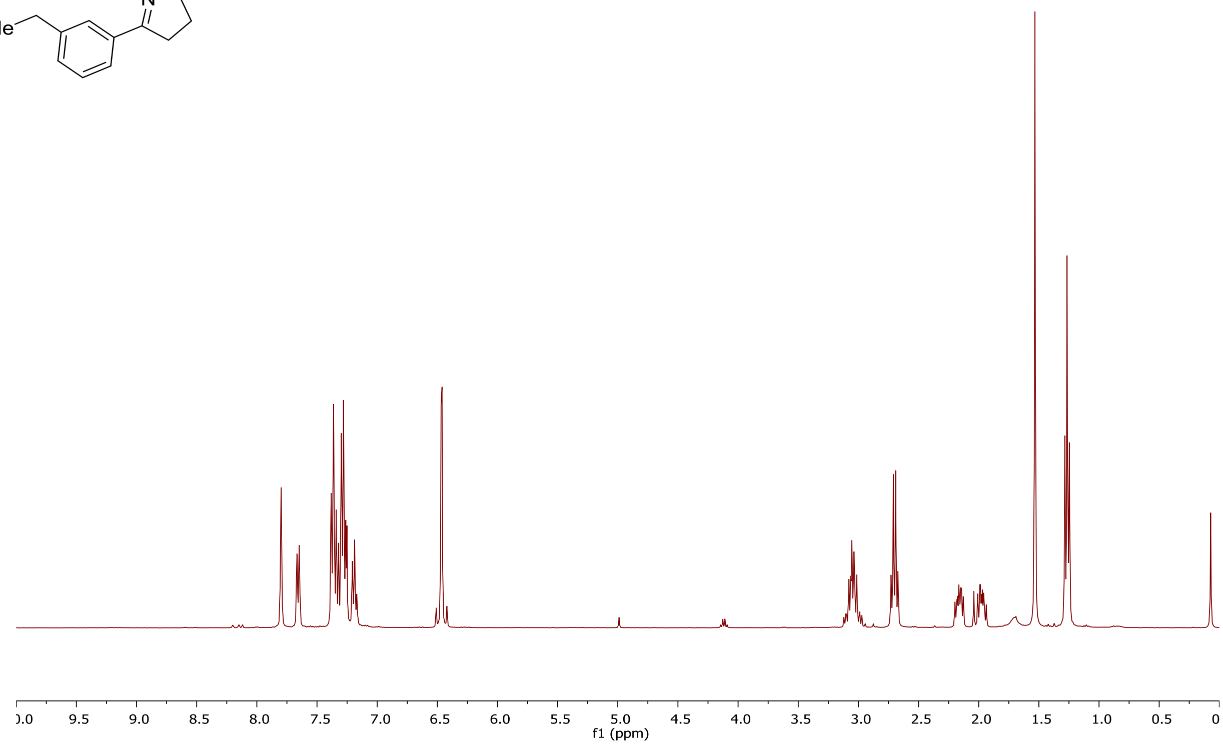
f1 (ppm)

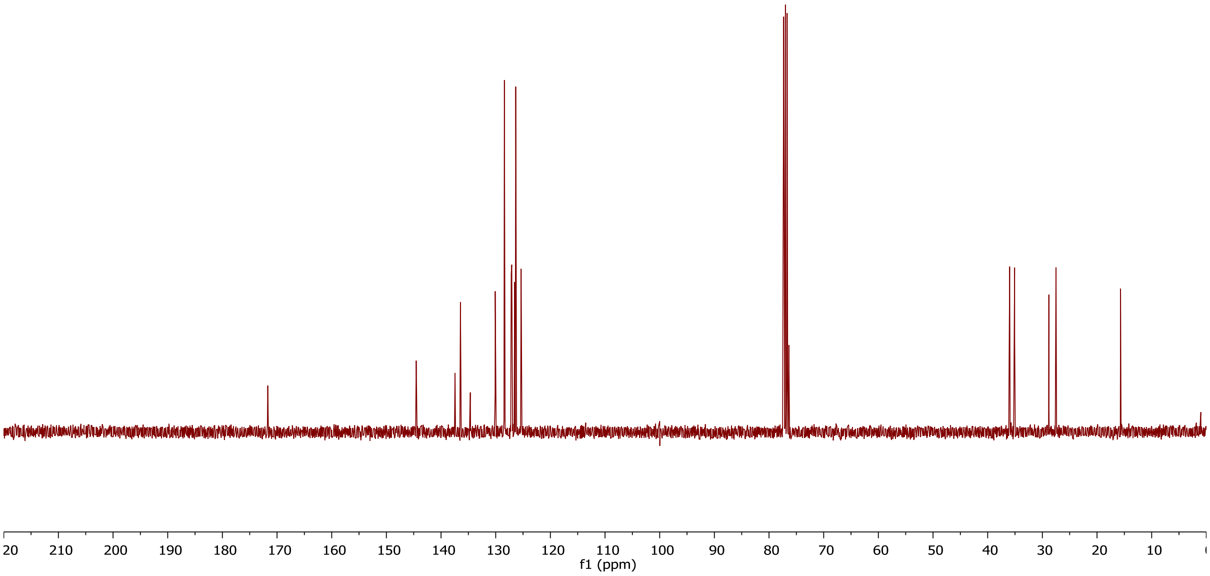
f1 (ppm)

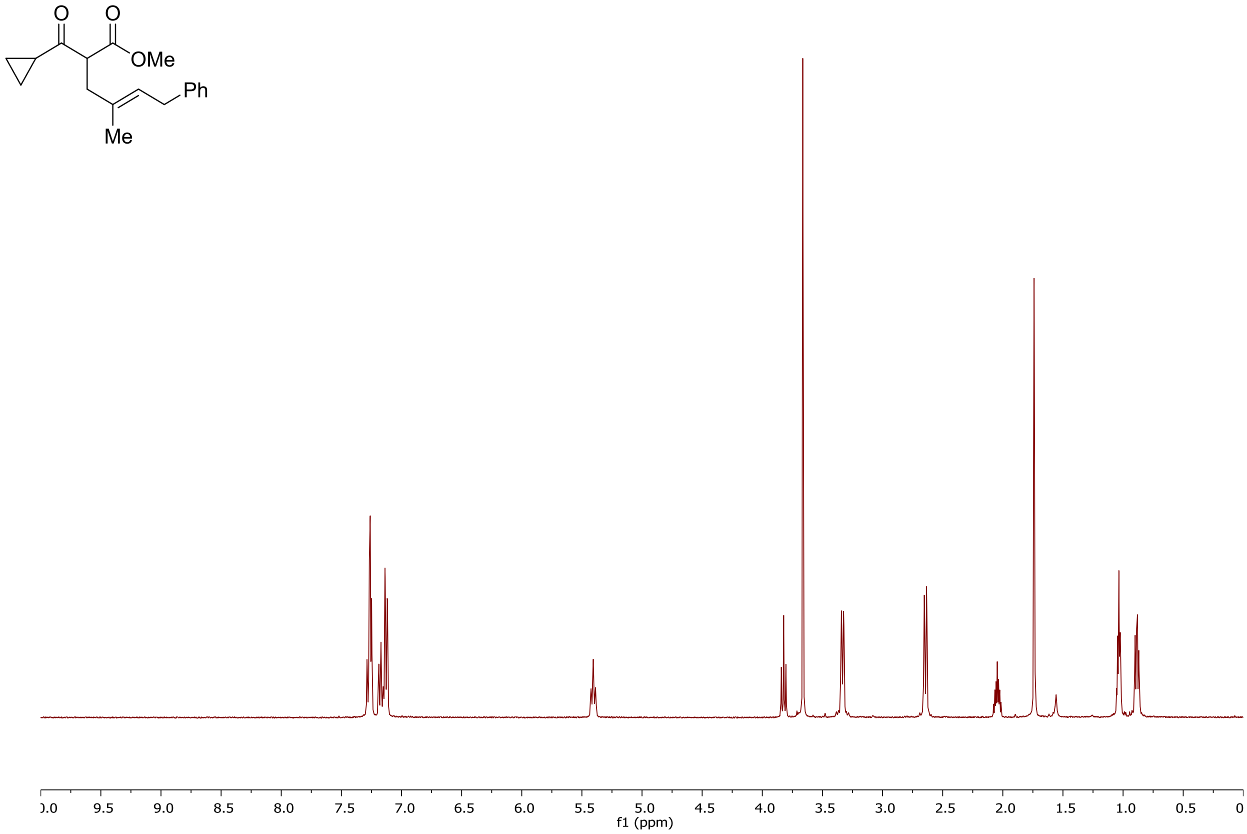
O
O
OMe
Ph
Me
f1 (ppm)

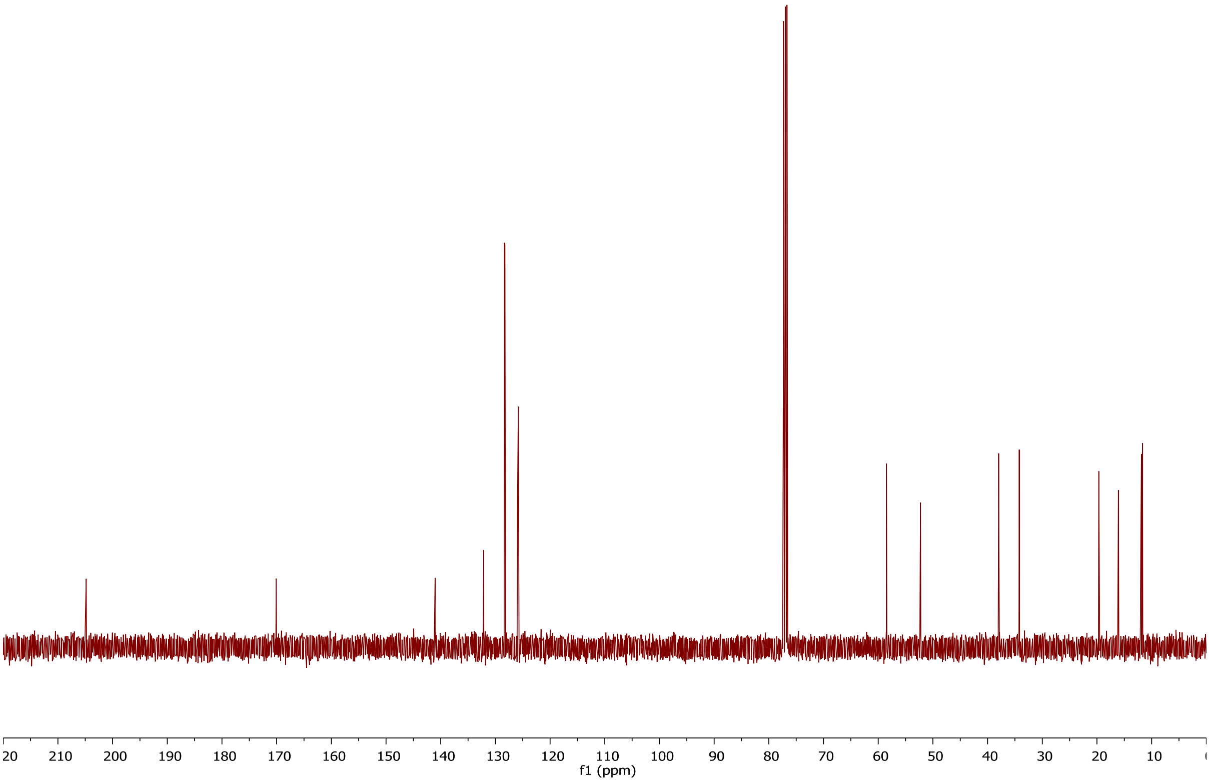
f1 (ppm)

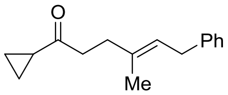
O
Ph
Me

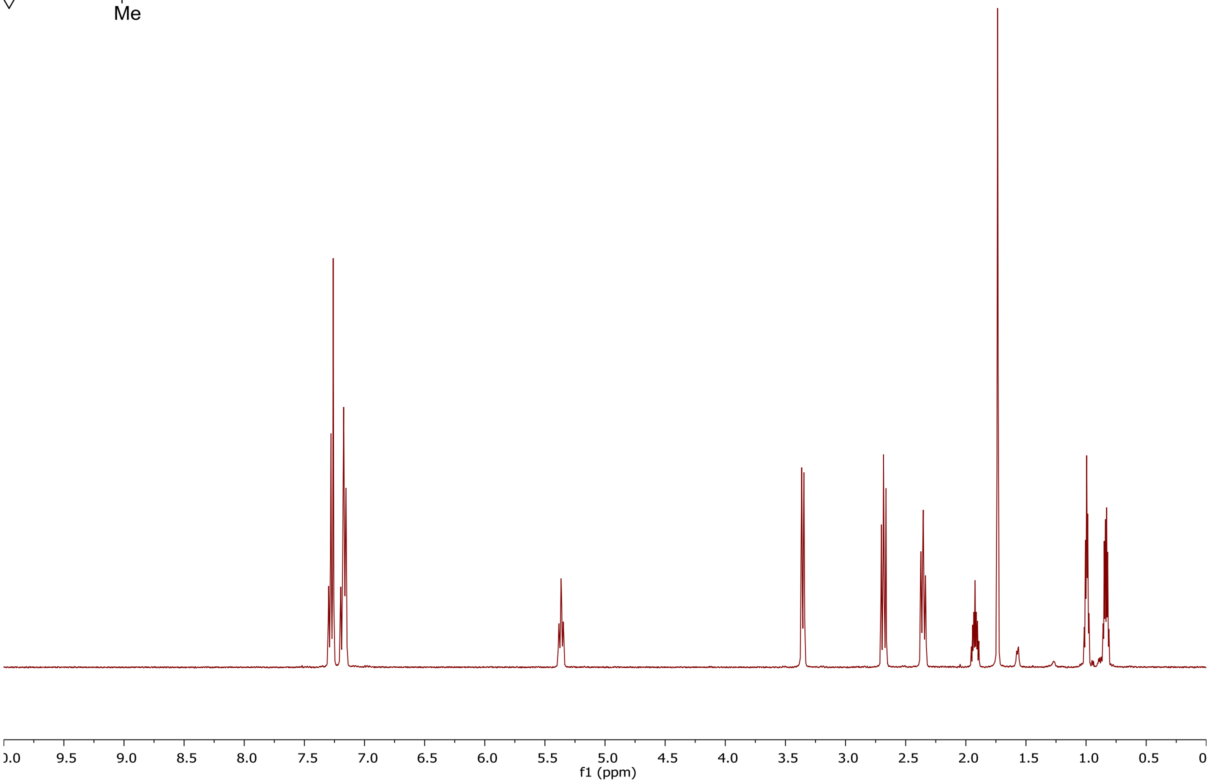
9.5 9.0 8.5 8.0 7.5 7.0 6.5 6.0 5.5 5.0 4.5 4.0 3.5 3.0 2.5 2.0 1.5 1.0 0.5
f1 (ppm)

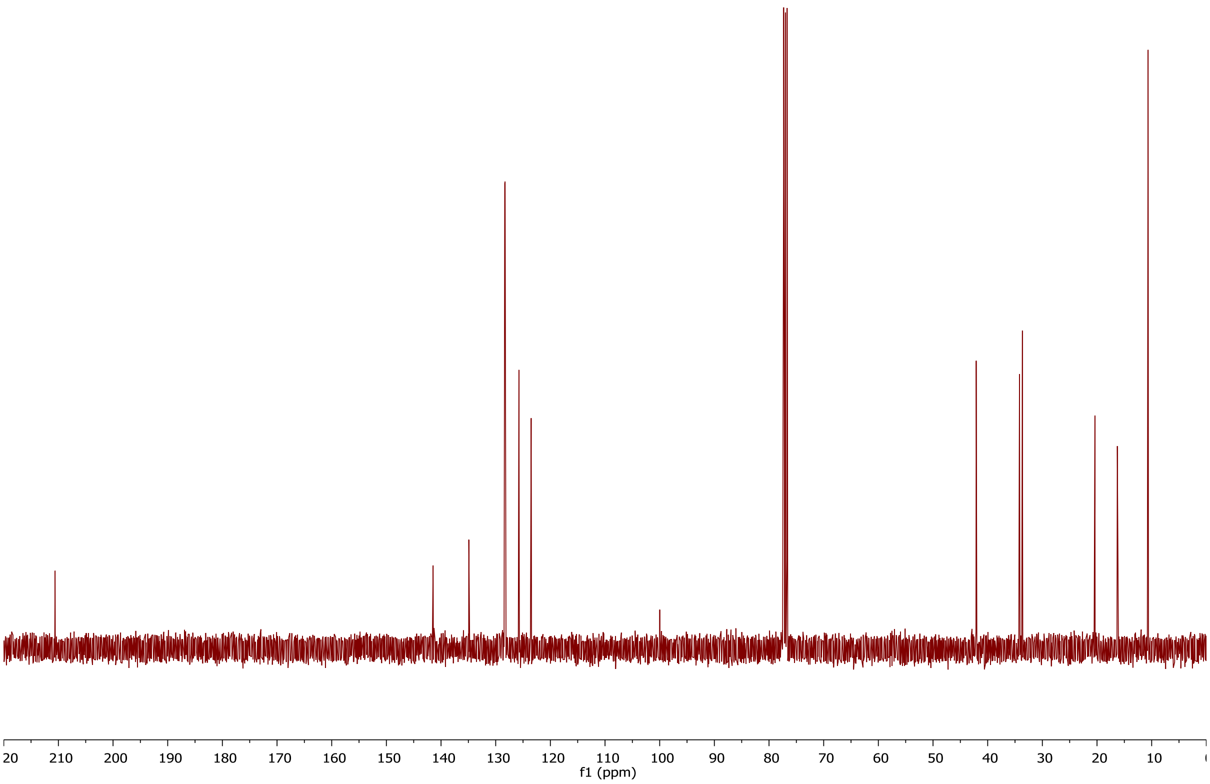
210 200 190 180 170 160 150 140 130 120 110 100 90 80 70 60 50 40 30 20 10
f1 (ppm)

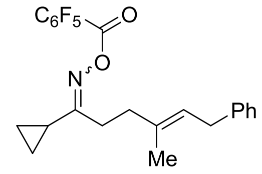
C6F5
O
N
O
Ph
Me

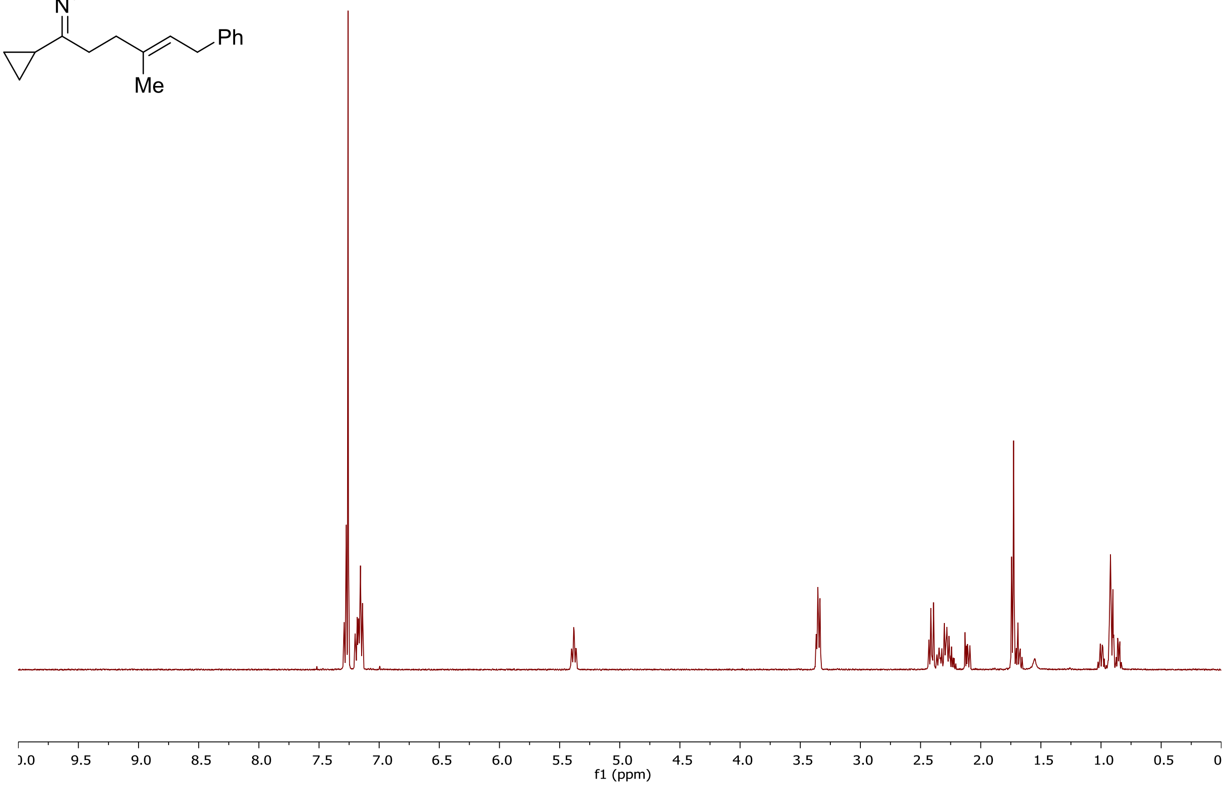
f1 (ppm)

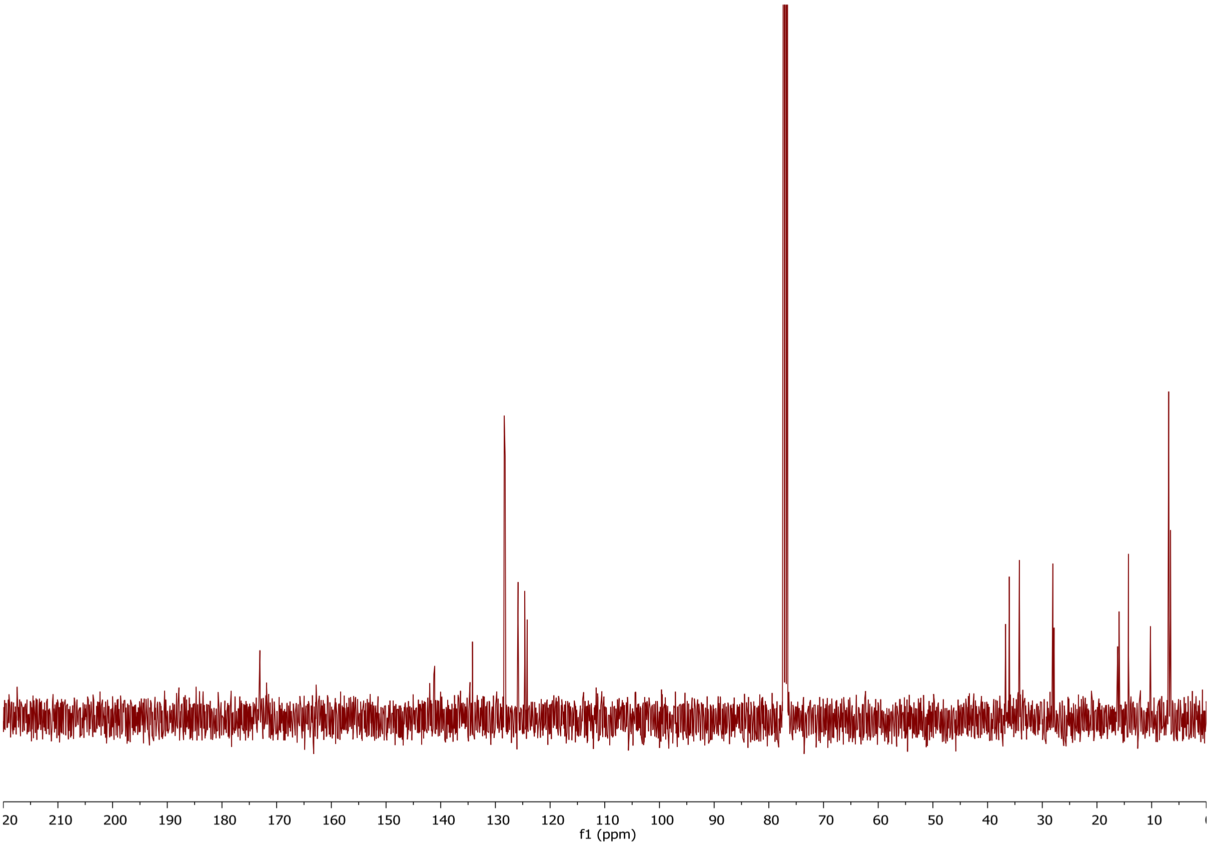
f1 (ppm)

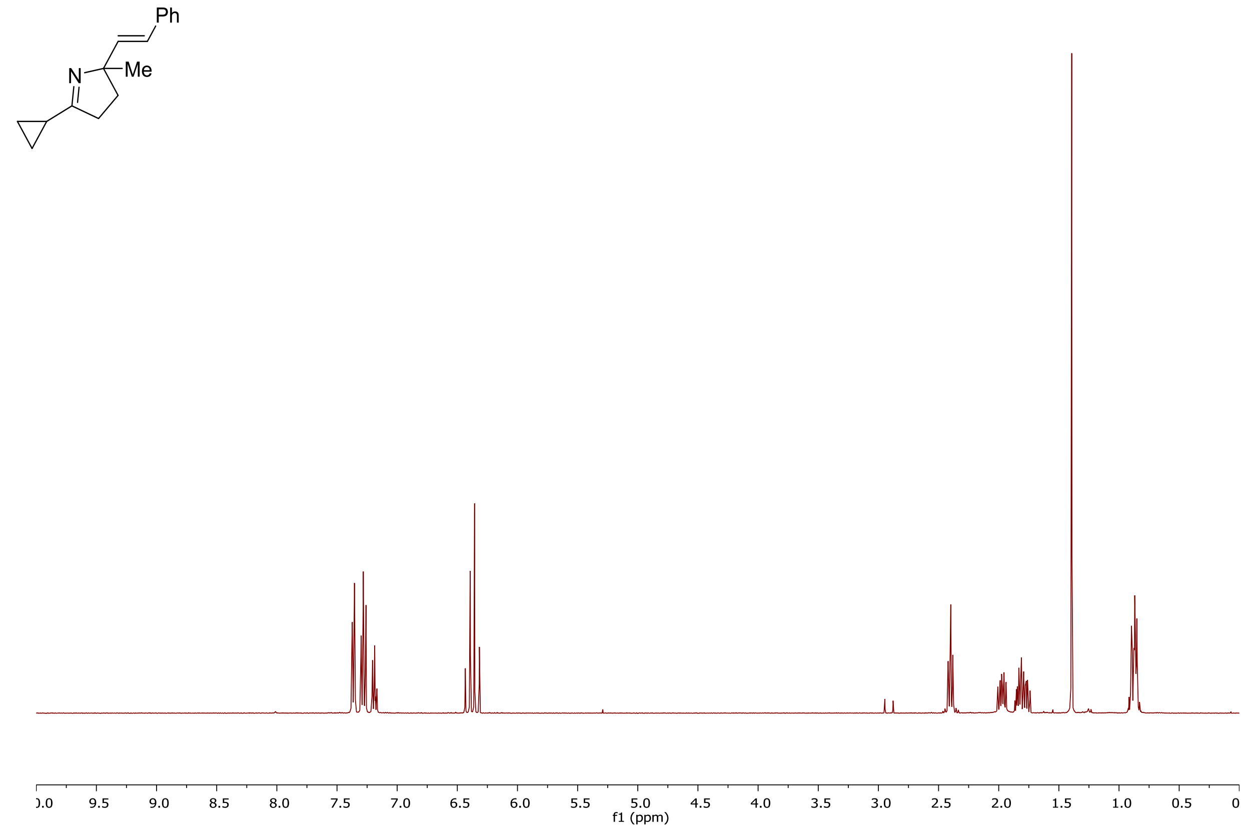
Ph
N
Me
f1 (ppm)

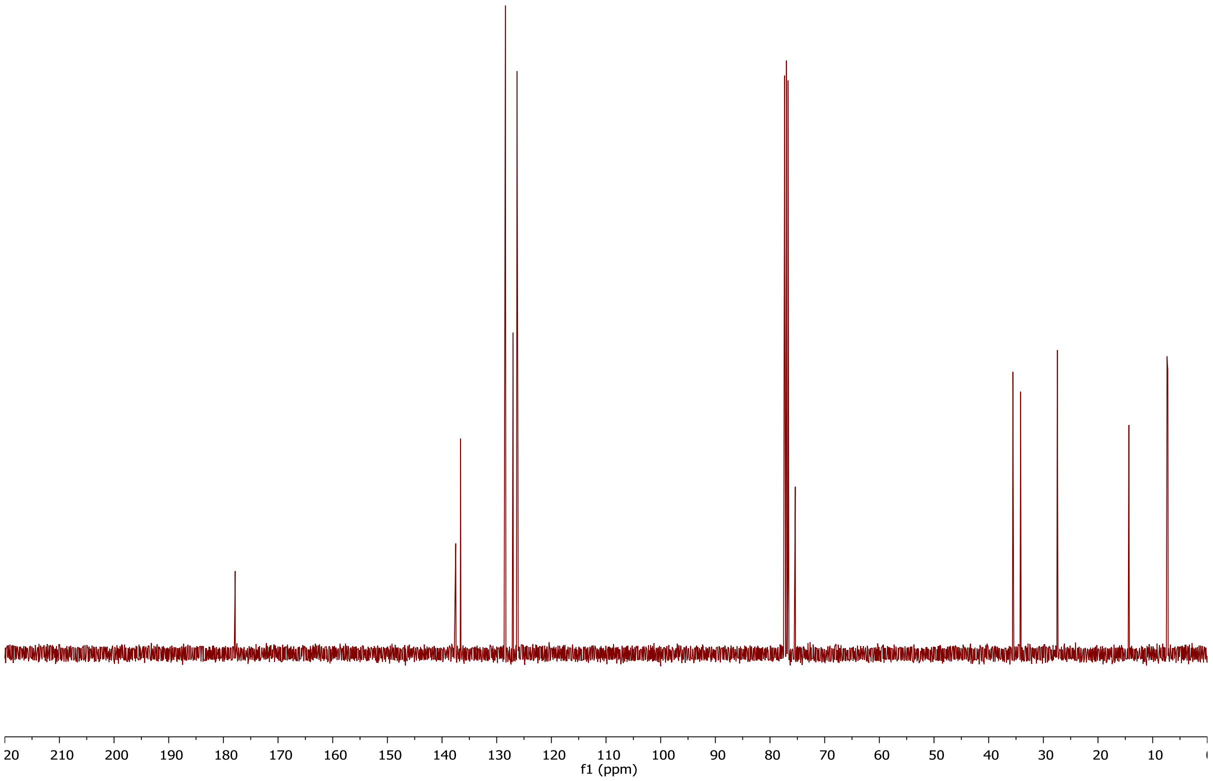
f1 (ppm)

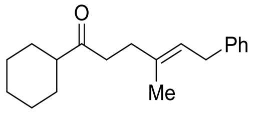
O
Ph
Me

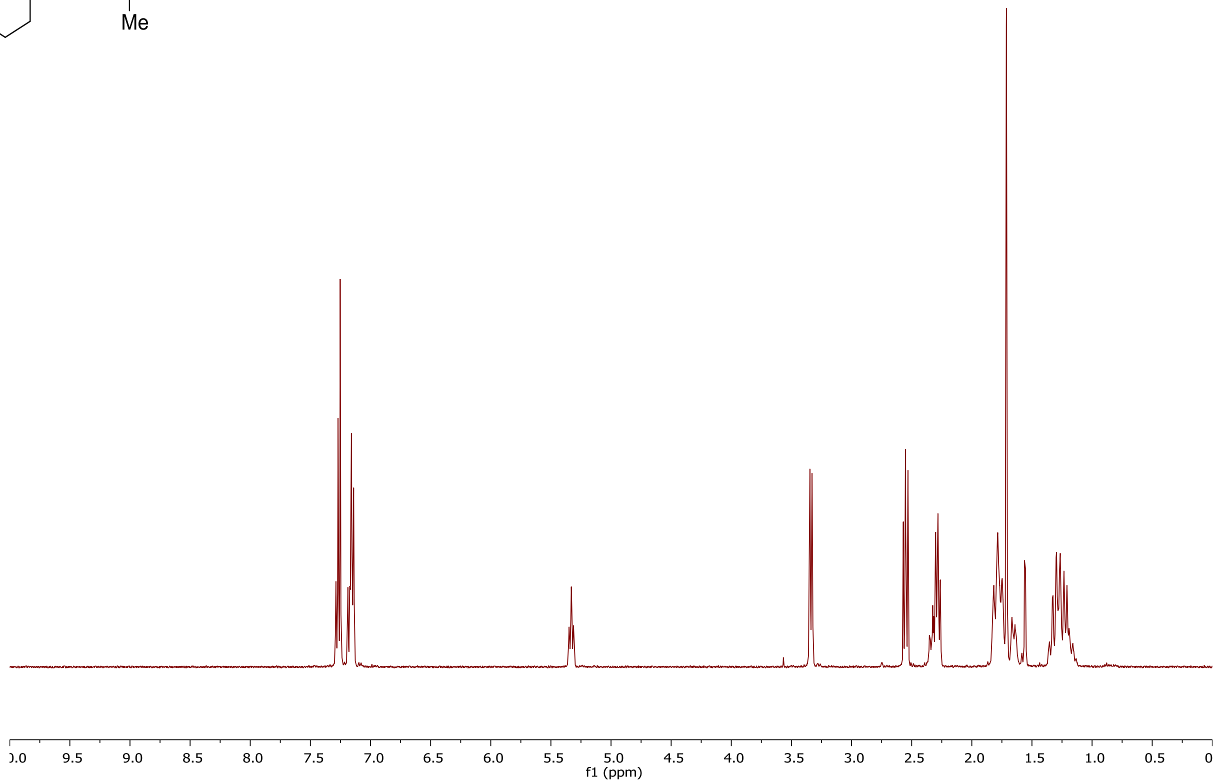
9.5 9.0 8.5 8.0 7.5 7.0 6.5 6.0 5.5 5.0 4.5 4.0 3.5 3.0 2.5 2.0 1.5 1.0 0.5
f1 (ppm)

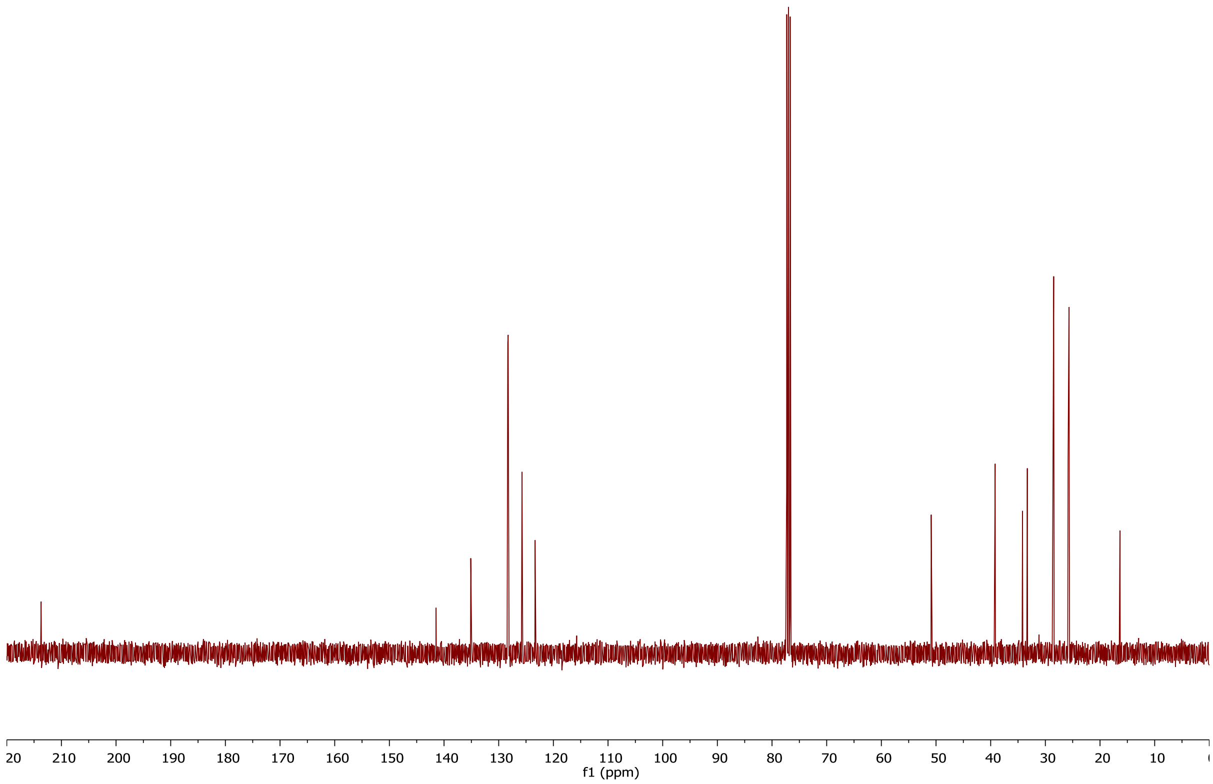
210 200 190 180 170 160 150 140 130 120 110 100 90 80 70 60 50 40 30 20 10
f1 (ppm)

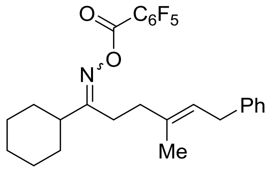
O
C6F5
O
N
Ph
Me

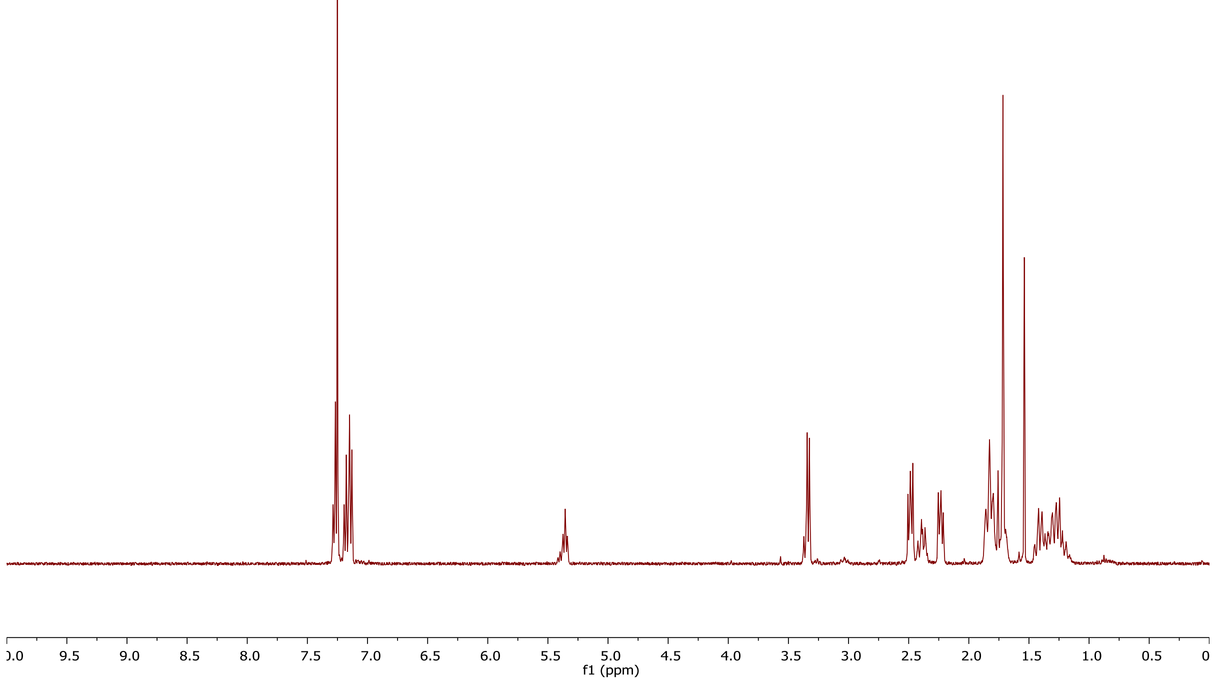
9.5 9.0 8.5 8.0 7.5 7.0 6.5 6.0 5.5 5.0 4.5 4.0 3.5 3.0 2.5 2.0 1.5 1.0 0.5
f1 (ppm)

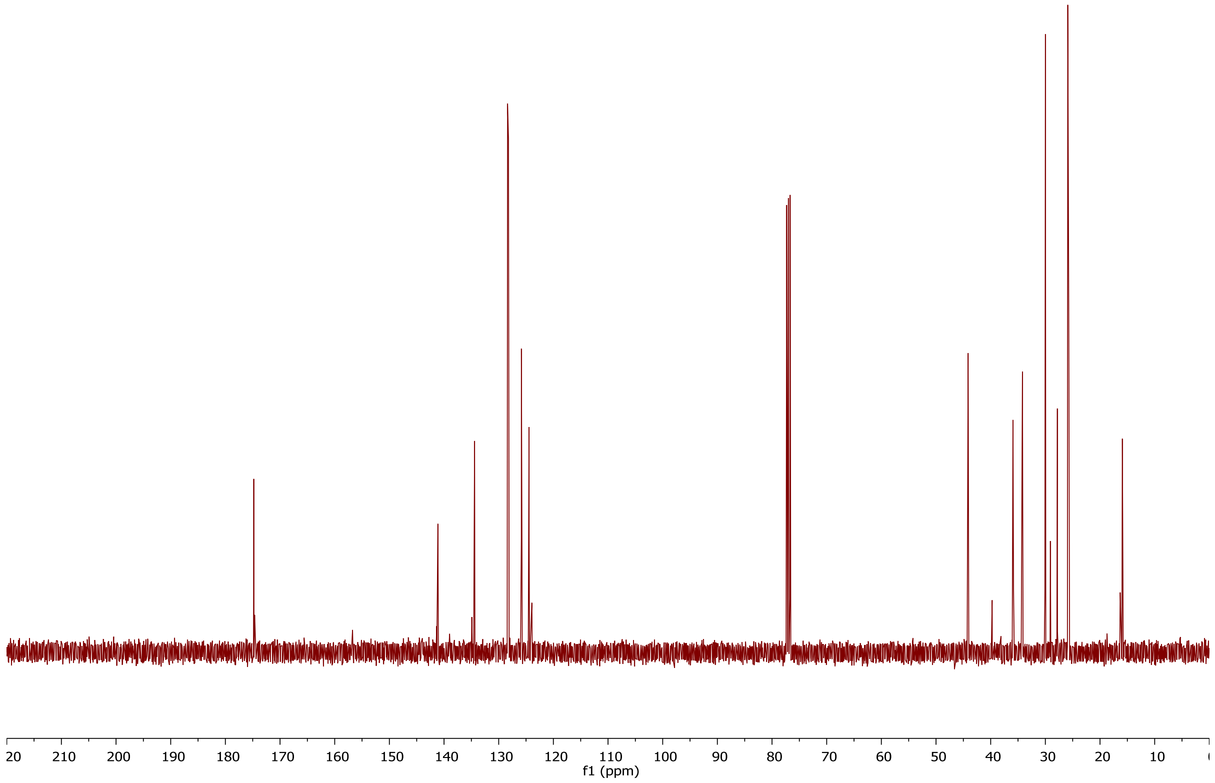
210 200 190 180 170 160 150 140 130 120 110 100 90 80 70 60 50 40 30 20 10
f1 (ppm)

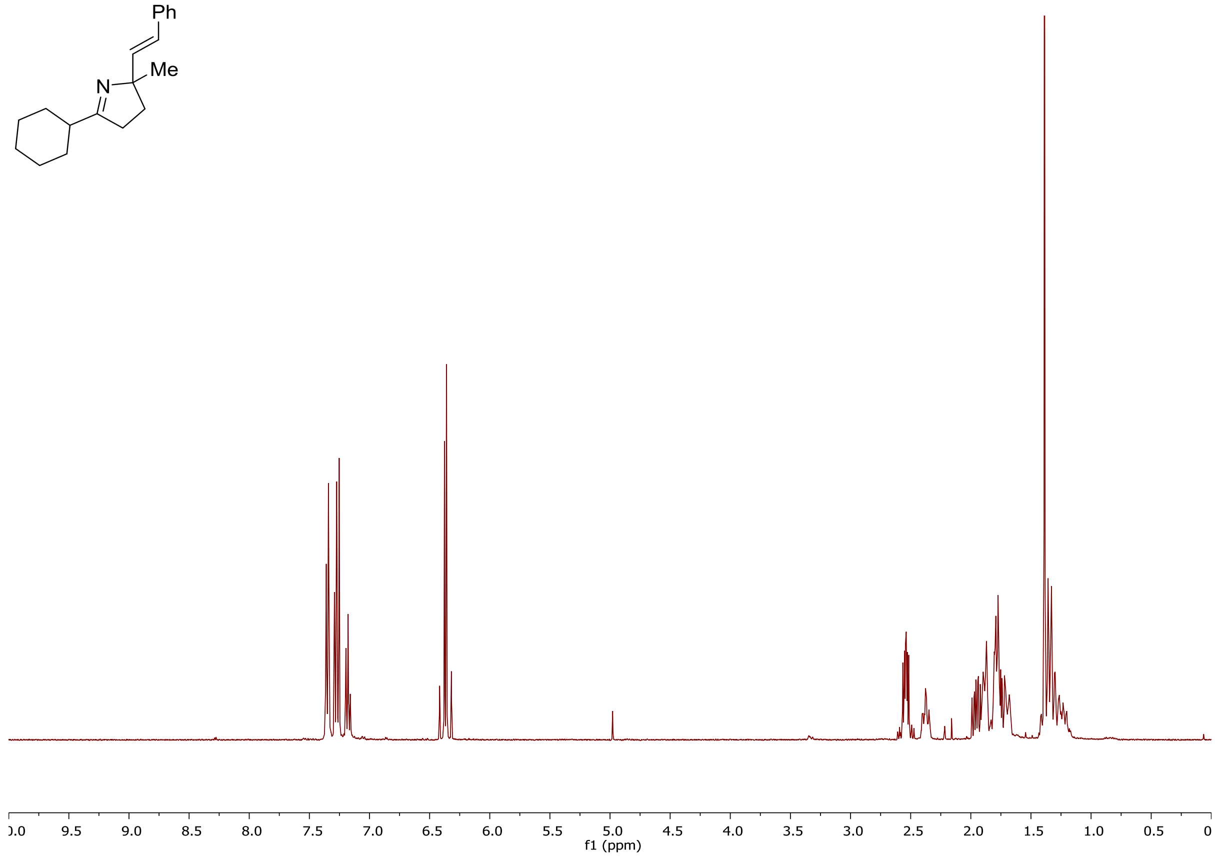
Ph
Me
N
f1 (ppm)

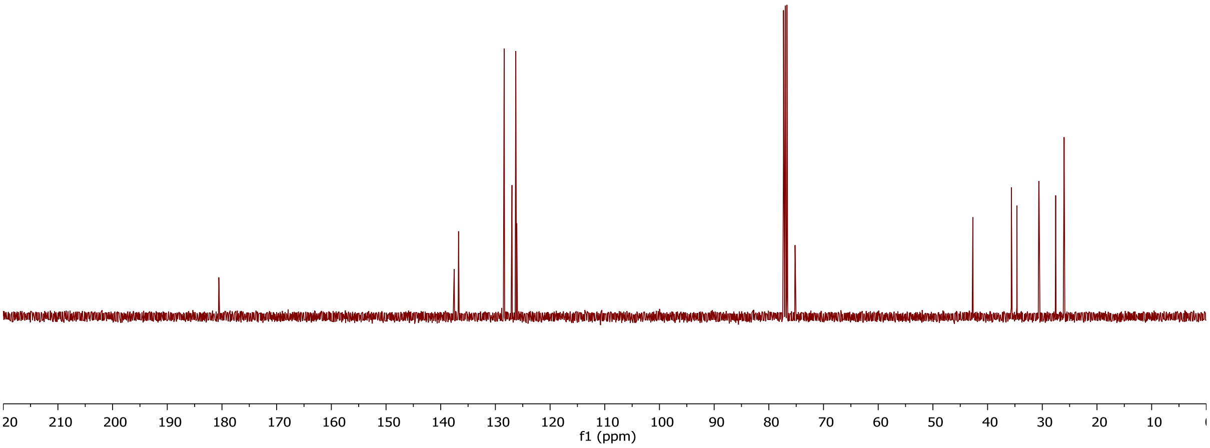
f1 (ppm)

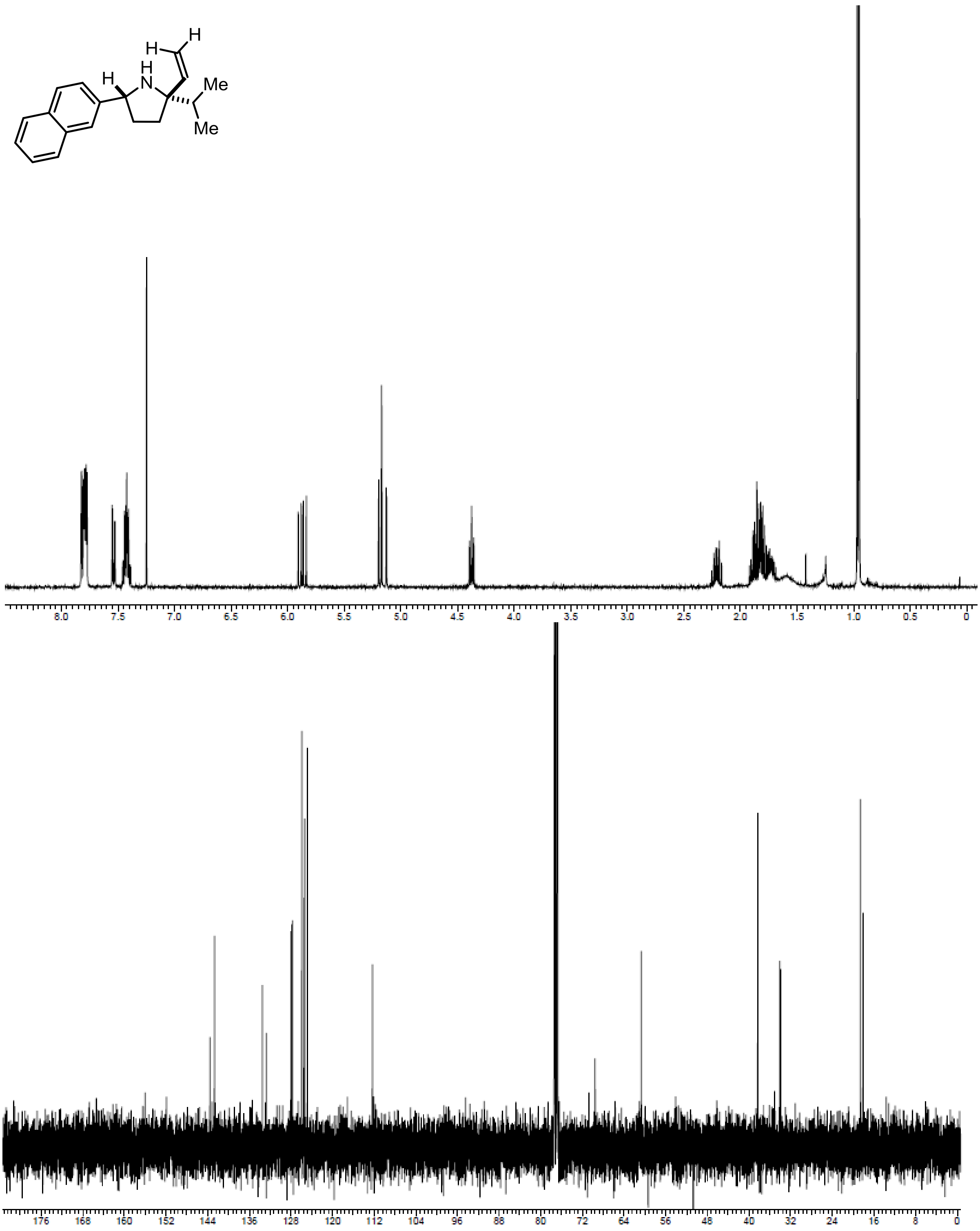

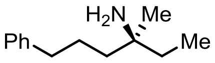
H2N
Me
Ph
Me

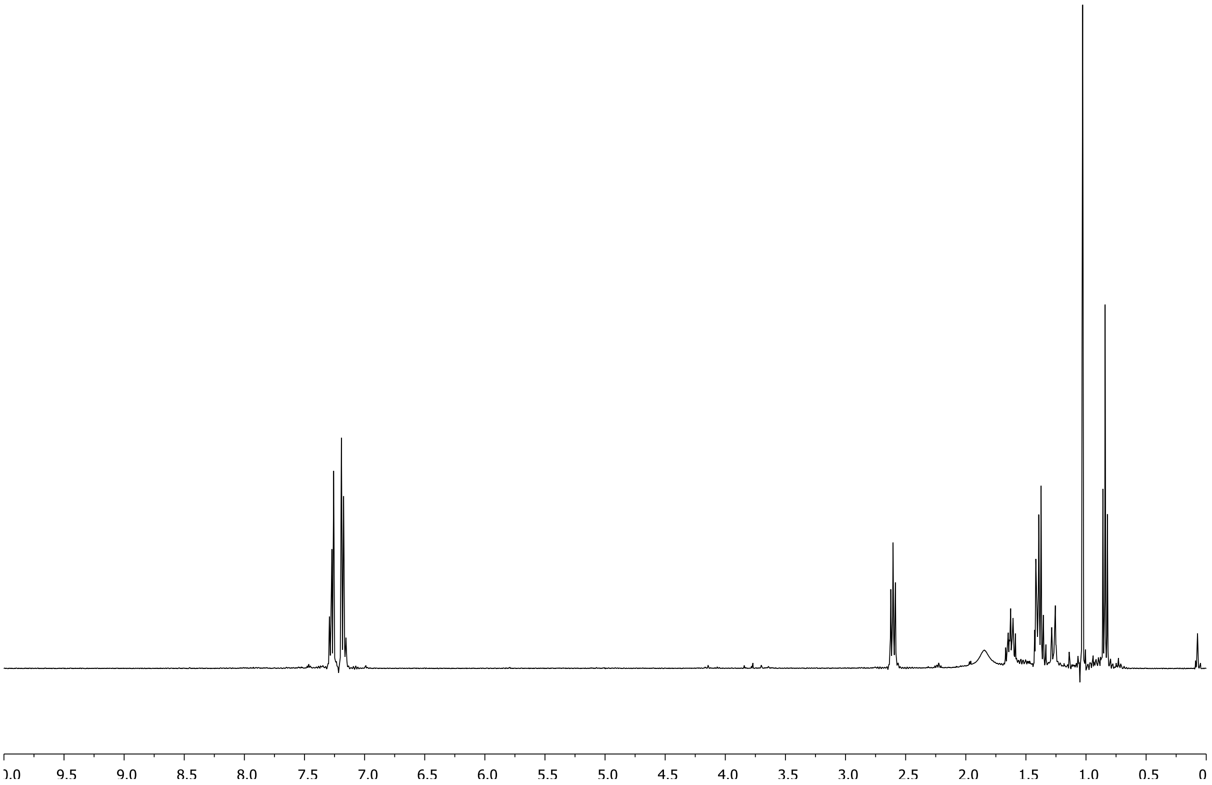

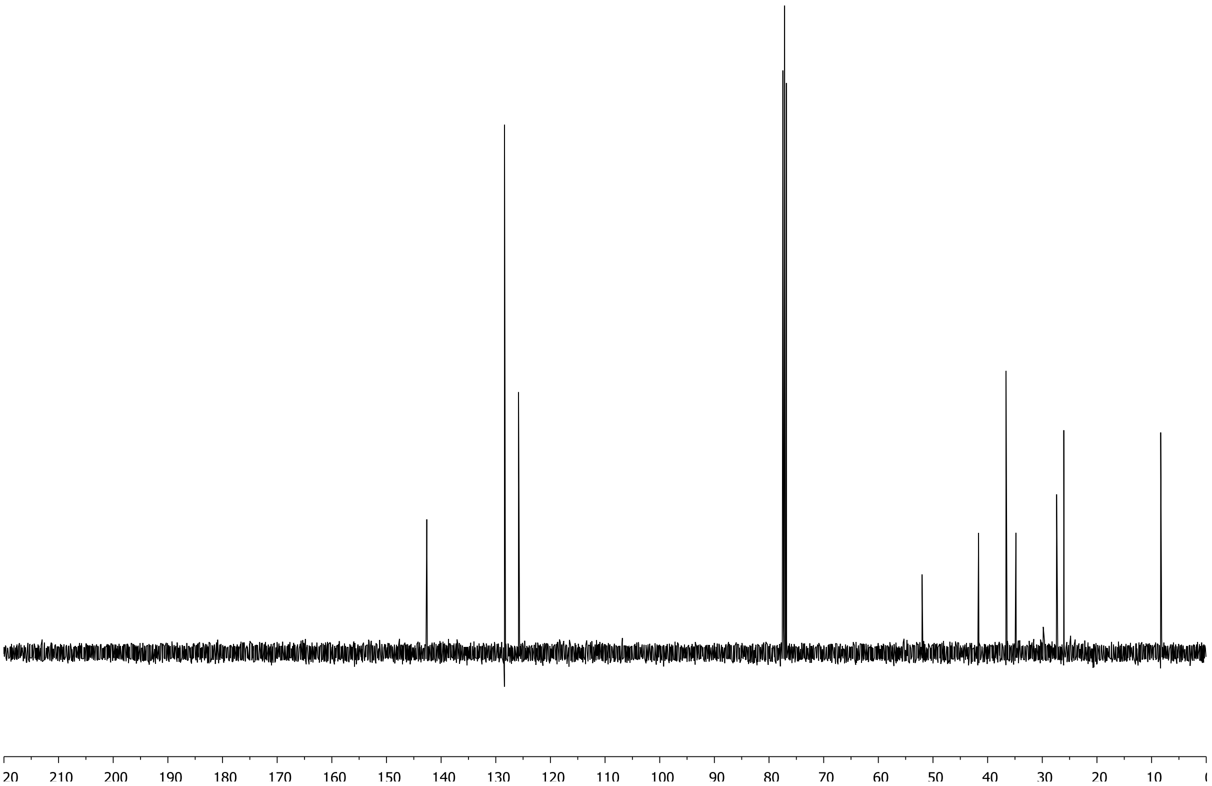

Supplement: Supplementary file 1 [file SC-008-C6SC04466B-s001.pdf]
